# ECEM 2017: Abstracts, August, 20^th^ to 24^th^, 2017, University of Wuppertal, Germany, Edited by Ralph Radach, Heiner Deubel, Christian Vorstius & Markus J. Hofmann

**DOI:** 10.16910/jemr.10.6.1

**Published:** 2017-12-23

## Contents

Keynote Speakers .......................................................................................... 4

Conference schedule ...................................................................................10

Keynotes ..................................................................................................10

Talks .........................................................................................................11 

Poster ......................................................................................................31

Abstracts ......................................................................................................53

Keynotes ......................................................................................................54

Talks .............................................................................................................60

Monday, August 21st, 10.30 - 12.30 ........................................................60

Symposium: Developmental eye tracking: Problems, solutions and applications of screen and head-mounted eye tracking ............................60

Thematic Session: Saccade programming I ..............................................64

Special Thematic Session: Communication by gaze interaction I ...............67

Thematic Session: Reading: Neural basis and binocular coordination ........69

Monday, August 21st 13.30 - 15.30 .........................................................73

Symposium: Using eye-tracking and pupillometry to study rhythmic processing in music and dance................................................................73

Thematic Session: Transsaccadic memory and integration .......................76

Special Thematic Session: Communication by gaze interaction II ..............79

Thematic session: Reading: Spatially distributed processing .....................81

Tuesday, August 22nd, 10.30 - 12.30 .......................................................85

Symposium: Longitudinal research on eye movements in developing readers: What have we learned so far? ...................................................85

Thematic Session: Clinical Research I ......................................................87

Thematic session: Visual interfaces, robotics and virtual reality ................91

Thematic Session: Scanpaths .................................................................94

Tuesday, August 22nd, 13.30 - 15.30 .......................................................97

Symposium: Eye movements during the reading of narrative and poetic text .............................................................................................................97

Thematic Session: Visual search ........................................................... 100

Thematic Session: Interactive and group eye-tracking ........................... 103

Thematic Session: Scene perception .................................................... 106

Wednesday, August 23rd, 10.30 - 12.30................................................ 109

Symposium: The role of eye movements in self-motion perception........ 109

Thematic Session: Attention and memory ............................................ 111

Thematic Session: Innovative methods and technology ......................... 114

Thematic Session: Reading: Predictive and high level processing ........... 117

Wednesday, August 23rd, 13.30 - 15.30................................................ 120

Symposium: Microsaccades: Modeling, Analysis, and Synthesis ..... 120

Thematic Session: Saccade control and fixational activity ...................... 123

Thematic Session: Eye-tracking in the educational context .................... 126

Thematic Session: Reading: Individual differences ................................. 129

Wednesday, August 23rd, 17.00 - 19.00................................................ 133

Symposium: Insights from Eye Movement Research with Immersive Technologies ....................................................................................... 133

Thematic Session: Pupillometry ........................................................... 136

Thematic Session: Learning and cognitive information processing .......... 139

Thematic Session: Reading: Corpus analysis and text processing ............ 142

Thursday, August 24th, 09.00 - 11.00 .................................................... 146

Symposium: Interpreting and using visualizations of eye movements to improve task performance and learning ............................................... 146

Thematic Session: Oculomotor event detection ................................ 149

Thematic Session: Usability and web-based interface design ............ 152

Thematic Session: Reading Basic oculomotor control ............................ 155 Thursday, August 25th, 11.30 - 13.30 .................................................... 158

Symposium: Pharmacological Influences on Voluntary Oculomotor Control .............................................................................................. 158

Thematic seesion: Saccade programming II ..................................... 160

Thematic session: Applied visual cognition ...................................... 164

Thematic session: Reading: Word level processing.......................... 168

Thursday, August 24th, 14.30 - 16.30 ....................................................172

Symposium: Yarbus, eye movements and vision 50 years on ..................172

Thematic session: Clinical Research II ....................................................175

Thematic session: Eye data analysis and evaluation ...............................178

Thematic session: Reading: Across the lifespan......................................180

Posters .......................................................................................................183

Session I - Monday, August 21st, 15.30 - 17:00 .....................................183

Session II - Tuesday, August 22nd, 15:30-17:00 .....................................231

Session III - Wednesday, August 23rd, 15.30 - 17.00 .............................277

Author Index ..............................................................................................321

........................................................................................................................

## Keynote Speakers

We are proud to present the following keynote speakers at ECEM 2017:

•Debra Titone, Canada

•Laure Pisella, France

•Marisa Carrasco, USA

•Ben Tatler, United Kingdom (Scotland)

•James Bisley, USA

•Karl Gegenfurtner, Germany

**Marisa Carrasco **

**Figure:**
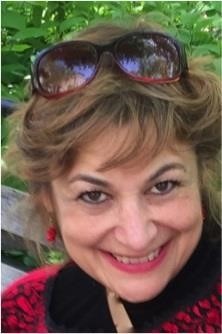


(New York University, United States of America) Keynote Lecture:

On covert attention and presaccadic attention

Marisa Carrasco, Professor of Psychology and Neural Science at New York University, uses human psychophysics, neuroimaging, and computational modeling to investigate the relation between the psychological and neural mechanisms involved in visual perception and attention. She received her Licentiate in Psychology from the National University of Mexico (1984) and her Ph.D. in Psychology from Princeton University (1989). She was an assistant professor at Wesleyan University (1989-1995) before joining NYU as an associate professor (1995), where she became a full professor (2001), served as the Chair of the Psychology Department (2001–2007), and was named a Collegiate Professor (2008). She has been the recipient of multiple awards, including a National Young Investigator Award from the National Science Foundation, an American Association of University Women Fellowship, a Cattell Fellowship and a Guggenheim Fellowship. Her research has been supported by the National Science Foundation and the National Institutes of Health. She has been a senior editor of two premiere journals of vision, *Vision Research* and *Journal of Vision*.

**Laure Pisella **

**Figure:**
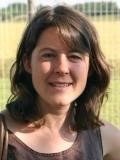


(Lyon Neuroscience Research Center, France)

Keynote Lecture:

An update of the functional role of the dorsal visual stream

Laure Pisella studied biology at the University of Lyon, France. She received a Master of Molecular and Cellular Biology in Ecole Normale Supérieure in 1997 and a PhD in Health Science (Neuropsychology in 2000 with a thesis on « Multiples pathways in interaction for Perception and Action». In 2001 she held a postdoctoral position at the University of Melbourne, funded by the National Health and Medical Research Council of Australia (NH MRC). In 2002, she obtained a full-time research position at the french Center for National Scientific Research (CNRS) and, since then, has been working for the INSERM team called "Espace et Action" in Bron, France. In 2006, she received the Bronze Medal of the CNRS. In 2008, she received her habilitation degree from the University of Lyon 1. In 2011, she joined the Integrative, Multisensory, Perception, Action and Cognition Team (ImpAct) of the Lyon Neuroscience research center (CRNL).

**Ben Tatler **

**Figure:**
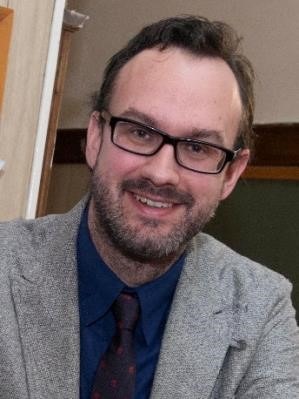


(University of Aberdeen, Scotland)

Keynote Lecture:

Where (and when) next? How people view images of natural scenes

Ben Tatler obtained an undergraduate degree in Natural Sciences from Cambridge University in 1998, and a PhD from the University of Sussex in 2002, under the supervision of Professor Mike Land FRS. After staying with Mike Land for his postdoc, Ben took up a lectureship in Psychology at the University of Dundee in 2004, where he stayed for the next 11 years. Since 2015, Ben has been a Professor of Psychology at the University of Aberdeen. Ben Tatler's Active Vision Lab studies how vision is used to provide the information we need to complete our activities of everyday life. Two key questions if we are to understand how vision supports behaviour are where we look and what we encode and retain from the places that we look at. This work spans domains from static scene viewing to real world settings. A particular emphasis in our lab is the importance of studying vision in the context of natural behaviour in real environments, rather than exclusively in laboratory settings.

**Debra Titone **

**Figure:**
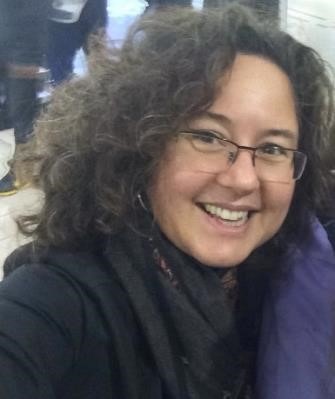


(McGill University, Montreal)

Keynote Lecture:

Eye movement studies of reading in special populations

Debra Titone has a PhD in Experimental Psychology at the State University of New York, Binghamton in 1995, and completed two postdoctoral fellowships - one at the Volen Center for Complex Systems, Brandeis University, and another at McLean Hospital, Harvard Medical School, where she was later appointed as Assistant Professor of Psychology in the Department of Psychiatry. Dr. Titone joined the faculty in the Psychology Department at McGill University in 2002, where she is a Full Professor and Director of the Multilingualism and Language Laboratory. Dr. Titone’s lab has had continuous research funding for the past 15+ years from a combination of sources including NSERC, SSHRC, CIHR, and NIH. Dr. Titone serves on the executive board of the Centre for Research on Brain, Language & Music, McGill University; is an elected Member-at-Large in the Canadian Society for Brain, Behavioral and Cognitive Science; serves as Officer in the NSF-funded Women in Cognitive Science group; and is co-founder of the NSERC-funded Women in Cognitive Science-Canada. Dr. Titone’s research on language and bilingualism has made use of different eye tracking paradigms in a variety of participant populations.

**James Bisley **

**Figure:**
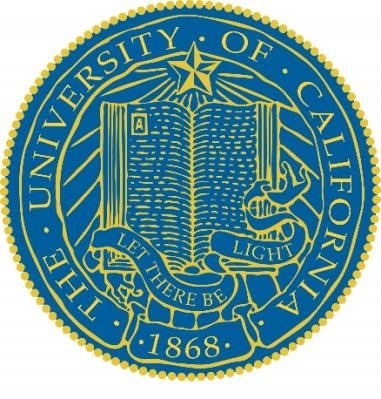


(University of California, United States of America)

Keynote Lecture:

The roles of cortical areas in guiding eye movements during visual search

Dr. Bisley received his Ph.D. from the University of Melbourne, where he studied the peripheral processing of shape information in the somatosensory system. In 1998 he moved to the University of Rochester as a postdoctoral fellow in the lab of Tania Pasternak, where he studied the neural mechanisms underlying short term memory for motion in area MT. He then joined the lab of Mickey Goldberg at the National Eye Institute and Columbia University, where he studied the guidance of visual attention. In 2006, Dr. Bisley joined the faculty in the Department of Neurobiology in the David Geffen School of Medicine at UCLA, where he remains. Dr. Bisley has been a Sloan fellow, a Kingenstein fellow and a McKnight Scholar. He is currently a reviewing editor for the Journal of Neuroscience. Dr. Bisley’s research interests revolve around the cognitive processing of visual information, with particular foci on understanding the neural mechanisms underlying the guidance of visual attention, the guidance of eye movements and spatial stability. An additional aim of his lab is to attempt to identify underlying mechanisms that may explain why neurons within an area seem to play different roles when tested in different tasks and to identify processing steps between areas.

**Karl R. Gegenfurtner **

**Figure:**
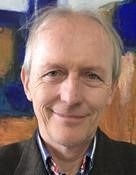


(Giessen University, Germany)

Keynote Lecture:

The interaction between vision and eye movements

Karl Gegenfurtner studied Psychology at Regensburg University. Subsequently he obtained a Ph.D. degree from New York University, where he also spent his first PostDoc. In 1993 he moved to the Max-Planck-Institute for biological cybernetics in Tübingen, where he obtained his Habilitation in 1998 and a HeisenbergFellowship in the same year. In 2000 he moved to the University of Magdeburg and in 2001 to Giessen University, where he since then holds a full professorship for Psychology. The emphasis of Karl Gegenfurtner’s research is on information processing in the visual system. Specifically, he is concerned with the relationship between low level sensory processes, higher level visual cognition, and sensorimotor integration. Karl Gegenfurtner is the head of the DFG Collaborative Research Center TRR 135 on the “Cardinal mechanisms of perception”. He was elected into the National Academy of Science Leopoldina in 2015 and received the Wilhelm-Wundt medal of the German Psychological Association (DGPS) in 2016.

## Conference schedule

Keynotes

**Auditorium Maximum** (HS 33 - K.11.24) 

**Sunday (18.00 - 19.00) **

**On covert attention and presaccadic attention**

Marisa Carrasco 

**Monday (9.00 - 10.00) **

**An update of the functional role of the dorsal visual stream **

Laure Pisella 

**Monday (17.00 - 18.00) **

**Where (and when) next? How people view images of natural scenes**

Ben Tatler 

**Tuesday (9.00 - 10.00) **

**Eye studies of reading in special populations **

Debra Titone 

**Wednesday (9.00 - 10.00) **

**The roles of cortical areas in guiding eye movements during visual search**

James Bisley 

**Thursday (17.30 - 18.30) **

**The Interaction between vision and eye movements** *Karl R. Gegenfurtner *

Talks

**Monday, August 21**^st^**, 10.30 - 12.30 **

Symposium: Developmental eye tracking: Problems, solutions and applications of screen and headmounted eye tracking

**Room 1** (HS 14 - M.10.12) Convenor: Tim J. Smith

10.30 Assessing gaze data quality in a large multi-centre autism developmental cohort 

*Ana M. Portugal, Luke Mason & Tim J. Smith*

10.50 Gazepath: An eye-tracking analysis tool that accounts for individual differences and data quality

*Daan van Renswoude, Maartje Raijmakers & Ingmar Visser*

11.10 Quantifying microdynamics of attention during parent-child interaction: 

 practicalities and insights 

*Nadia Neesgaard, Atsushi Senju & Tim J. Smith*

11.30 Head-mounted eye-tracking for studying infants’ attention during naturalistic activities 

*Heather L. Kirkorian & Seung Heon Yoo *

11.50 Infants’ naturalistic attention dynamics show similar patterns at different spatio temporal scales 

*Samuel V. Wass, Kaili Clackson, Stani Georgieva & Victoria Leong *

12.10 Active Vision: What head-mounted eye tracking reveals about infants' active visual exploration 

 Chen Yu 

Thematic : Saccade programming I

** Room 2** (HS 32 - K.11.23) Chair: Lynn Huestegge

10.30 Fixation-related brain activations: emotional valence interacts with high and lowlevel image properties 

*Michał Kuniecki, Joanna Pilarczyk, Kinga Wołoszyn & Aleksandra Domagalik *

10.50 Learning sequences of eye movements: linking motor processing and cognition in the brain 

*Melanie R. Burke & Claudia C. Gonzalez *

11.10 Oculomotor dominance in dual tasking and the influence of stimulus-response modality mappings 

*Mareike A. Hoffmann, Aleks Pieczykolan & Lynn Huestegge*

11.30 Using Averaging to study Decision Making signals

*Geoffrey Megardon & Petroc Sumner *

11.50 The necessity to choose causes effects of expected value

*Christian Wolf, Anna Heuer, Anna Schubö & Alexander C. Schütz *

12.10 SERIA – A model for antisaccades 

*Eduardo A. Aponte, Dario Schoebi, Klaas E. Stephan & Jakob Heinzle *

12

Special thematic session: Communication by gaze interaction I

**Room 3** (HS 28 - I.13.71) Chair: Andreas Bulling & Carlos H. Morimoto 

10.30 Eye movement as material for interaction design (40 min)

*Hans Gellersen *

11.10 “Here´s looking at you, kid.” Does he see pupil size changes? 

*Anke Huckauf, Christoph Strauch & Jan Ehlers*

11.30 Gaze-contingent for Neurocognitive Therapy: More than Meets the Eye? 

*Leanne Chukoskie, Jacqueline Nguyen & Jeanne Townsend*

11.50 Applicability of smooth-pursuit based gaze interaction for elderly users 

*Sarah-Christin Freytag, Stefan Ruff & Antje C. Venjakob*

12.10 Behavioral Analysis of Smooth Pursuit Eye Movements for Interaction 

* Argenis Ramirez-Gomez & Hans Gellersen*****

Thematic session: Reading: Neural basis and binocular coordination

**Room 4** (HS 26 - I.13.65) : Hazel I. Blythe

10.30 Saccadic eye movements and neural activity associated with letter naming speed task manipulations 

*Noor Z. Al Dahhan, Donald C. Brien, John R. Kirby & Douglas P. Munoz *

10.50 The effects of cloze probability and semantic congruency on brain responses during natural reading: A fixation-related fMRI study

*Sarah Schuster, Nicole A. Himmelstoß, Stefan Hawelka, Fabio Richlan, *

 Martin Kronbichler & Florian Hutzler 

11.10 Reading fluency is associated with fixation related brain responses to reading comprehension in 12-year old typically reading children – findings from co- registered eye-tracking and EEG study

*Otto Loberg, Jarkko Hautala, Jarmo A. Hämäläinen & Paavo H.T. Leppänen *11.30 Changes in overall vergence demands affect binocular coordination during reading 

*Stephanie Jainta *

11.50 Binocular for parafoveal processing in reading

*Hazel I. Blythe, Mirela Nikolova, Stephanie Jainta & Simon P. Liversedge *

12.10 A new understanding of vergence within fixations, based on differences in the reading of Chinese and English 

*Richard Shillcock, Yi-ting Hsiao, Mateo Obregón, Hamutal Kreiner, Matthew A. J. *

Roberts & Scott McDonald

13

**Monday, August 21**^st^**, 13.30 - 15.30 **

Symposium: Using eye-tracking and pupillometry to study rhythmic processing in music and dance

**Room 1** (HS 14 - M.10.12) Convenors: Elke B. Lange & Lauren K. Fink 

 13.30 Eye Can’t Dance; Entraining Saccadic Timing to Musical and Visual Beats 

*Jonathan P. Batten & Tim J. Smith *

 13.50 Pupil dilation indexes the metrical hierarchy of unattended rhythmic violations 

*Atser Damsma & Hedderik van Rijn *

 14.10 Predicting attention to auditory rhythms using a linear oscillator model and pupillometry 

*Lauren K. , Joy J. Geng, Brian K. Hurley & Petr Janata *

 14.30 The Eye-Time Span in Music Reading: Local Effects of Stimulus Complexity on 

 “Looking Ahead” 

*Erkki Huovinen, Anna-Kaisa Ylitalo & Marjaana Puurtinen*

 14.50 Guided eye movements made in response to dance

*Matthew Woolhouse *

 15.10 Eye-movement control and pupillary responses to complex auditory and visual stimuli (Panel discussion) 

Thematic session: Transsaccadic memory and integration

** 2** (HS 32 - K.11.23) Chair: Artem Belopolsky

13.30 Beyond the magic number four: Evidence for high-capacity, trans-saccadic, fragile memory and pre-attentive remapping 

*Paul Zerr, Surya Gayet, Kees Mulder, Ilja Sligte & Stefan Van der Stigchel *

13.50 Unifying the visual world across an eye-movement: Transsaccadic integration is unaffected by saccade landing position

*Martijn Jan Schut, Nathan Van der Stoep & Stefan Van der Stigchel *

14.10 How quickly does the eye movement system register changes across saccades? 

*Jonathan van Leeuwen & Artem V. Belopolsky*

14.30 Trans-saccadic feature integration is contrast dependent

*Lukasz Grzeczkowski, Heiner Deubel & Martin Szinte*

14.50 Task-relevant compete for attention across saccades 

*Christian H. Poth & Werner X. Schneider *

15.10 Remapping of the global effect across saccades

*Kiki Arkesteijn, Jeroen BJ Smeets, Mieke Donk & Artem V. Belopolsky*****

DOI: 10.16910/jemr.10.6 This work is licensed under a

Special thematic session: Communication by gaze interaction II

** Room 3** (HS 28 - I.13.71) Chair: John P. Hansen & Roman Bednarik 

 13.30 Gaze interaction using low-resolution images at 5 FPS

*Carlos E.L. Elmadjian, Antonio Diaz-Tula, Fernando O. Aluani & Carlos H. Morimoto *

 13.50 PSOVIS: An interactive tool for extracting post-saccadic oscillations from eye movement data 

*Diako , Thomas Wilcockson, Baiqiang Xia, Hans Gellersen, Trevor Crawford & Peter Sawyer *

 14.10 GazeBall: Leveraging Natural Gaze Behavior for Continuous Re-calibration in 

 Gameplay 

*Argenis Ramirez-Gomez & Hans Gellersen *

 14.30 Implicit Events in Virtual Reality: A New Concept for Eye-Based Interaction? 

*Teresa Hirzle, Jan Ehlers, Anke Huckauf & Enrico Rukzio*

 14.50 COGAIN Association Meeting (40 min)****

Thematic session: Reading: Spatially distributed processing

**Room 4** (HS 26 - I.13.65) Chair: Sarah Risse 

 13.30 Two routes of parafoveal processing during reading: Eye movements suggest benefits and costs 

 Sarah Risse & Martin R. Vasilev 

 13.50 Late interference by parafoveal difficulty in reading *Stefan Seelig & Sarah Risse *

 14.10 Analyzing Sequential Dependencies between Fixation Durations with Linked 

 Linear Mixed Models 

*Reinhold Kliegl, Sven Hohenstein & Hannes Matuschek*

 14.30 What are the costs of degraded parafoveal previews during silent reading? *Bernhard Angele, Martin R. Vasilev, Timothy J. Slattery & Julie A. Kirkby*

 14.50 Effects of type on parafoveal letter identification in Russian *Svetlana Alexeeva, Aleksandra Dobrego & Alena Konina *

 15.10 Orthographic, Morphological, and Semantic Parafoveal Processing in Arabic 

 Reading: Evidence from the Boundary Paradigm 

*Ehab W. Hermena, Eida J. Juma, Ascensión Pagán, Maryam AlJassmi, Mercedes *

Sheen & Timothy R. Jordan

DOI: 10.16910/jemr.10.6 This work is licensed under a

**Tuesday, August 22**^nd^**, 10.30 - 12.30**

**Symposium: Longitudinal research on eye movements in developing readers: What have we learned so far? **

**Room 1** (HS 14 - M.10.12) Convenors: Johannes Meixner & Christian Vorstius 

 10.30 The development of eye movement control in reading: where do the eyes go? *Ralph Radach, Christian Vorstius & Christopher J. Lonigan *

 10.50 Early development of oculomotor control in reading: a longitudinal eye tracking study from preschool age to fifth grade

*Thomas Günther, Josefine Horbach, Wolfgang Scharke & Ralph Radach *

 11.10 Foveal Processing Difficulty Modulates Perceptual Span Early in Reading 

 Development 

*Johannes M. & Jochen Laubrock *

 11.30 Comprehension in silent and oral sentence reading: Longitudinal evidence from developing readers 

*Christian Vorstius, Young-Suk Grace Kim & Ralph Radach*

**11.50 The development of foveal eye movements in primary school: Findings from the Berlin DevTrack study

*Sascha Schroeder, Simon Tiffin-Richards & Sarah Eilers*

 12.10 General discussion**

Thematic session: Clinical Research I

** 2** (HS 32 - K.11.23) Chair: Andreas Sprenger

10.30 Implicit and explicit oculo-motor learning in Parkinson’s disease and spinocerebellar ataxia 

*Andreas Sprenger, Annika Lasrich & Christoph Helmchen*

10.50 Visual exploration of emotional faces in schizophrenia using masks from the Japanese Noh theatre 

*Teresa Fasshauer, Andreas Sprenger, Karen Silling, Christopher Zeiser, Johanna Elisa Silberg, Anne Vosseler et al.*

11.10 Visual Behavior on Natural Static Images in Patients with Retinitis Pigmentosa *Ricardo R. Gameiro, Kristin Jünemann, Anika Wolff, Anne Herbik, Peter König & Michael Hoffmann*

11.30 Quantifying Traumatic Brain Injury impairments in scanning patterns of complex scenes 

*Nitzan Guy, Oryah Lancry & Yoni Pertzov *

11.50 Smooth pursuit disturbances in schizophrenia during free visual exploration of dynamic natural scenes 

*Johanna Silberg, Ioannis Agtzidis, Mikhail Startsev, Teresa Fasshauer, Karen *

Silling, Andreas Sprenger et al. 

12.10 Eye tracking live social interaction to capture gaze behavior of subclinical autism and social anxiety 

*Roy S. Hessels, Gijs A. Holleman, Tim H. W. Cornelissen, Ignace T. C. Hooge & *

DOI: 10.16910/jemr.10.6 This work is licensed under a

16

* Chantal Kemner*

Thematic session: Visual interfaces, robotics and virtual reality

**Room 3** (HS 28 - I.13.71) Chair: Lucas Paletta

 10.30 Smooth pursuit based mouse replacement: the GazeEverywhere system 

*Simon Schenk, Marc Dreiser, Philipp Tiefenbacher, Gerhard Rigoll & Michael Dorr *

 10.50 Fixation-Related Potentials as a Measure for Cognitive Demand in Visual Tasks on 

 Single Trial Level 

*Dennis , Andrea Finke, Shirley Mey, Dirk Koester, Thomas Schack & Helge Ritter *

 11.10 Gaze Contingent Control of Vergence, Yaw and Pitch of Robotic Eyes for Immersive Telepresence 

*Remi Cambuzat, Frédéric Elisei & Gérard Bailly*

 11.30 Measurement of Situation Awareness in Collaborative Robotics Using Eye Movement Features 

*Lucas Paletta, Cornelia Murko & Amir Dini*

**11.50 Siamese Convolutional Neural Networks for Appearance-Based Gaze Estimation 

*Helen Zhou, David Mayo & Scott Greenwald*

 12.10 Joint visual working memory through implicit collaboration 

*Edwin S. Dalmaijer, Diederick C. Niehorster, Kenneth Holmqvist & Masud Husain *

Thematic session: Scanpaths

**Room 4** (HS 26 - I.13.65) Chair: Ralf Engbert 

 10.30 Disentangling fixation duration and saccadic planning using gaze dependent guided viewing

*Benedikt V. Ehinger, Lilli Kaufhold & Peter König*

 10.50 The early central fixation bias in scene viewing: Experimental manipulation and modeling 

*Lars O. M. Rothkegel, Hans A. Trukenbrod, Heiko H. Schütt, Felix A. Wichmann & Ralf Engbert *

 11.10 Likelihood-based Parameter Estimation and Comparison of Dynamical Eye Movement Models 

*Heiko H. , Lars O. M. Rothkegel, Hans A. Trukenbrod, Sebastian Reich, Felix A. Wichmann & Ralf Engbert*

 11.30 Refixation strategies for memory encoding in free viewing

*Radha N. Meghanathan, Andrey R. Nikolaev & Cees van Leeuwen *

 11.50 Modelling saccade directions with circular distributions

*Ingmar Visser, Maartje Raijmakers & Daan van Renswoude*

 12.10 Considering, rather than restricting eye movement characteristics in Fixation Related Potentials: an application of the rERP framework 

*Tim Cornelissen, Jona Sassenhagen, Dejan Draschkow & Melissa Le-Hoa Vo*

DOI: 10.16910/jemr.10.6 This work is licensed under a

17

**Tuesday, August 22**^nd^**, 13.30 - 15.30**

Symposium: Eye movements during the reading of narrative and poetic text

** Room 1** (HS 14 - M.10.12) Convenors: Arthur M. Jacobs & Jana Lüdtke 

 13.30 Weary with toil, I haste me to my bed: Eye tracking Shakespeare sonnet 

*Shuwei Xue, Daniela Giordano, Jana Lüdtke, Renata Gambino, Grazia Pulvirenti, Concetto Spampinato & Arthur M. Jacobs*

**13.50 Individual differences in eye-movement patterns in response to literary language **

*Emiel den Hoven, Franziska Hartung, Michael Burke & Roel M. Willems *

 14.10 Exploring meaning construction in readers of English-language Haiku: An eye-tracking study****

*Franziska Günther, Hermann J. Müller, Thomas Geyer, Jim Kacian & Stella Pierides *

**14.30 Immersion, Emotion and Eye Movements in Self-paced Reading of passages from Harry Potter 

*Lea Musiolek, Jana Lüdtke & Arthur M. Jacobs*

 14.50**Using eye movements to study comprehension monitoring in beginning readers *Young-Suk Kim, Christian Vorstius & Ralph Radach *

 15.10 General discussion******

Thematic session: Visual search

** 2** (HS 32 - K.11.23) Chair: Rebecca Foerster

13.30 Your Attention seeks Confirmation: Visual confirmation bias overshadows prevalence effects in visual attention 

*Stephen C. Walenchok, Stephen D. Goldinger & Michael C. Hout *

13.50 Humans do not make efficient eye movements during visual search 

*Anna Nowakowska, Alasdair D.F. Clarke & Amelia R. Hunt*

14.10 Time course of brain activity during unrestricted visual search: Co-registering 

 EEG and Eye Movements 

*Juan E. Kamienkowski, Alexander Varatharajah, Mariano Sigman, Rodrigo Quian *

Quiroga & Matias J. Ison 

14.30 Visual -memory biases attention: Evidence for involuntarily object- based top-down control by search-irrelevant features

*Rebecca M. Foerster & Werner X. Schneider*

14.50 Eye Movements and the Label Feedback Effect: Speaking Modulates Visual Search, But Probably Not Visual Perception 

*Katherine P. Hebert, Stephen C. Walenchok & Stephen D. Goldinger *

15.10 Shorter fixation durations in visual search after 24 hours of total sleep deprivation 

DOI: 10.16910/jemr.10.6 This work is licensed under a

18

*Christian & Daniel Aeschbach*

Thematic session: Interactive and group eye-tracking

** Room 3** (HS 28 - I.13.71) Chair: Edwin Dalmaijer

 13.30 Group Eye Tracking (GET) Applications in Gaming and Decision Making * Cengiz Acarturk, Mani Tajaddini & Ozkan Kilic*

 13.50 Mass measurement of eye-movements under the dome - proof of concept studies 

*Maksymilian , Katarzyna Potęga vel Żabik, Michał Gochna & Jacek Mikulski *

 14.10 Using multiple gaze trackers and combining the results *Miika T. Toivanen & Markku Hannula*

 14.30 Joint Attention on the Cartesian Plain: A Dual Eye-Tracking Study 

*Anna Shvarts & Anatoly Krichevets*

**14.50 How Teachers See It: Using Mobile Eyetracking to Explore Professional Vision and Teacher-Student Interactions in the Classroom 

*Irene T. Skuballa & Antje von Suchodoletz*

 15.10 Gaze-assisted remote communication between teacher and students 

*Kari-Jouko Räihä, Oleg Spakov, Howell Istance & Diederick C. Niehorster *****

Thematic : Scene perception

**Room 4** (HS 26 - I.13.65) Chair: Antje Nuthmann

13.30 The relative importance of foveal vision in visual search in 3D dynamic scenes * Adam C. Clayden, Robert B. Fisher & Antje Nuthmann *

13.50 The developmental trajectory of eye movements to object-scene inconsistencies and their relation to language abilities

*Sabine Öhlschläger & Melissa Le-Hoa Vo *

 14.10 Dynamic recipes for oculomotor selection of objects in realistic scenes 

*Sara Spotorno & Ben Tatler *

 14.30 Individual Smooth Pursuit Strategies in Dynamic Natural Scene Perception *Ioannis Agtzidis, Mikhail Startsev & Michael Dorr*

 14.50 The of saccade duration distribution

*Hélène Devillez, Randall C. O’Reilly & Tim Curran*

15.10 Using sound to guide gaze in a ‘split-screen’ film: Mike Figgis’ Timecode as a found experiment 

*Tim J. Smith, Jonathan P. Batten & Jennifer X. Haensel*

**, 10.30 12.30**

Symposium: The role of eye movements in self-motion perception

**Room 1** (HS 14 - M.10.12) Convenors: Paul R. MacNeilage & Jonathan S. Matthis 

 10.30 Gaze the visual control of foot placement when walking over real-world rough terrain 

*Jonathan S. Matthis & Mary Hayhoe *

 10.50 Eye movement cues to self-motion perception 

*Ivar Clemens, Luc Selen, Antonella Pomante, Paul MacNeilage & Pieter Medendorp *

 11.10 Visual-vestibular conflict detection depends on fixation *Isabelle Garzorz & Paul MacNeilage *

 11.30 Heading representations in primates are compressed by saccades****

*Frank Bremmer, Jan Churan & Markus Lappe*

**11.50 Dynamics of eye movements during visual path integration in primates 

*Kaushik J. Lakshminarasimhan, Xaq Pitkow & Dora Angelaki*

 12.10 General discussion 

Thematic session: Attention and memory

** Room 2** (HS 32 - K.11.23) Chair: Daniel Smith

10.30 Attentional selection in averaging saccades 

*Luca Wollenberg, Heiner Deubel & Martin Szinte*

10.50 Vertical gaze paralysis is associated deficits of attention and memory: Evidence from Progressive Supranuclear Palsy

*Daniel Smith & Neil Archibald *

11.10 Nasal-temporal on cueing effect: how cue eccentricity and visual 

 field affect the orienting of visuo-spatial attention

*Soazig Casteau & Daniel T. Smith *

11.30 Presaccadic attention analyzed with a novel dynamic noise paradigm 

*Nina Maria Hanning & Heiner Deubel *

11.50 Detecting concealed memory via eye movements 

*Oryah Lancry, Tal Nahari, Gershon Ben-Shakhar & Yoni Pertzov *

12.10 Spoken words help in retreiving information from visual working memory *Seema Gorur Prasad, Pratik Bhandari & Ramesh Mishra*

Thematic session: Innovative methods and technology

** Room 3** (HS 28 - I.13.71) Chair: Catrin Hasse

 10.30 Improving computerized adaptive testing using eye tracking measures *Benedict C.O.F. Fehringer *

 10.50 Eye movement indicators for successful failure detection

*Catrin Hasse & Carmen Bruder*

 11.10 Individual versus subjective fixation disparity as a function of prism load 

*Wolfgang Jaschinski *

 11.30 3D Eye Tracking in Monocular and Binocular Conditions

*Xi Wang, Marianne Maertens & Marc Alexa*

**11.50 Using Priors to Compensate Geometrical Problems in Head-Mounted Eye Trackers 

*Fabricio B. Narcizo, Zaheer Ahmed & Dan W. Hansen*

 12.10 The development and validation of a high-speed stereoscopic eye tracker *Annemiek D. Barsingerhorn, Nienke Boonstra & Jeroen Goossens *

Thematic session: Reading: Predictive and high level processing

**Room 4** (HS 26 - I.13.65) Chair: Victoria McGowan

10.30 Beyond cloze probability: Semantic and syntactic preview effects in reading 

*Aaron Veldre & Sally Andrews *

10.50 Are older readers “riskier”? Examining adult age differences in reading 

*Victoria A. McGowan, Sarah J. White, Kayleigh L. Warrington & Kevin B. Paterson *11.10 Benchmarking n-gram, topics and recurrent neural network models in predicting 

 word cloze completion and eye movement variance 

*Markus J. Hofmann, Chris Biemann, Steffen Remus & Ralph Radach *

11.30 Predictive processing is key for reading: An evaluation of a visual information 

 optimization with eye movements in reading

*Benjamin Gagl & Christian Fiebach *

11.50 The processing of bounded and unbounded negated representations during reading: An eye-movement investigation 

*Lewis T. Jayes, Hazel I. Blythe, Kevin B. Paterson & Simon P. Liversedge *12.10 Using eye tracking to "figure out" how verb-particle constructions are 

 understood during L1 and L2 reading 

*Mehrgol Tiv, Laura Gonnerman, Veronica Whitford, Deanna Friesen, Debra Jared & *

 Debra Titone 

**, 13.30 15.30**

Symposium: Microsaccades: Modeling, Analysis, and Synthesis

**Room 1** (HS 14 - M.10.12) Convenors: Andrew T. Duchowski, Krzysztof Krejtz & Izabela Krejtz 

 13.30 Dynamic Modeling of Fixational Eye Movements: The Role of Neural Delays *Ralf Engbert, Carl J. J. Hermann & Ralf Metzler*

 13.50 Saliency and Surprise Revealed by Microsaccades

*Yoram S. Bonneh, Uri Polat, Misha Tsodyks & Yael Adini*

 14.10 Evaluating Microsaccades for Cognitive Load Measurement

*Krzysztof Krejtz, Izabela Krejtz, Andrew T. Duchowski, Cezary Biele & Anna Niedzielska *

 14.30 Microsaccades of ADHD Patients during Facial Affect Recognition 

*Nina Gehrer, Michael Schönenberg, Krzysztof Krejtz & Andrew T. Duchowski *

 14.50 Microsaccades Visual Search of Gaussian Terrain

*Justyna Żurawska & Andrew T. Duchowski*

**15.10 Perception of Synthesized Microsaccadic Jitter

*Andrew T. Duchowski, Sophie Jörg & Krzysztof Krejtz*

Thematic session: Saccade control and fixational activity

** Room 2** (HS 32 - K.11.23) Chair: Stefan van der Stigchel

13.30 Rapid of spatial working memory across saccades

*Artem V. Belopolsky, Paul J. Boon, Silvia Zeni & Jan Theeuwes *

13.50 Perceptual continuity across saccades: evidence for rapid spatiotopic updating 

*Jasper Hajo Fabius, Alessio Fracasso & Stefan Van der Stigchel *

14.10 Spatiotemporal dynamics and topological network characteristics of the 

 fixation-related EEG lambda activity 

*Andrey R. Nikolaev, Marcello Giannini, Hossein Seidkhani, Radha Nila Meghanathan, David Alexander & Cees van Leeuwen*

14.30 Microsaccade features and microsaccade-related alpha-synchronization across the life span 

*Ying Gao & Bernhard Sabel *

14.50 Unifying micro and macro-saccades with a space dependent, stochastic 

 threshold 

*Geoffrey Megardon & Aline Bompas *

15.10 The relationship between visual sampling and hippocampal activity in younger and older adults 

*Zhong-Xu Liu, Kelly Shen, Rosanna K. Olsen & Jennifer D. Ryan*

Thematic session: Eye-tracking in the educational context

** Room 3** (HS 28 - I.13.71) Chair: Halszka Jarodzka

 13.30 A tool to assist teachers to determine if learners apply the divisibility rules correctly *Pieter Potgieter & Pieter Blignaut*

 13.50 Using Eye-Tracking to Measure Strategies of Comparing the Numerical Values of 

Fraction 

*Andreas Obersteiner*

 14.10 Adapting instruction to learners’ gaze behavior: Does an adaptive multimedia system support learning? 

*Anne Schueler, Marie-Christin Krebs, Thérése F. Eder & Katharina Scheiter *

 14.30 The effects of conceptual and perceptual difficulty on processing and engagement in text during reading and learning 

*Alexander Strukelj, Marcus Nyström & Kenneth Holmqvist*

 14.50 How are processing strategies reflected in the eyes? Triangulating results from selfreports and eye tracking 

*Leen , David Gijbels & Vincent Donche*

**15.10 Teachers’ perceptions and interpretations of classrooms in the digital age 

*Halszka Jarodzka, Liesbeth Meijer & Sharisse Van Driel*

Thematic session: Reading: Individual differences

**Room 4** (HS 26 - I.13.65) Chair: Mallorie Leinenger

13.30 Effects of individual language skills on phonological coding during skilled reading: Evidence from survival analyses of eye movement data 

 Mallorie 

13.50 Individual differences and context properties affect word learning 

*Victor Kuperman & Bryor Snefjella *

14.10 Using Latent-Growth-Curve-Models to Examine Children’s Eye-movements 

 During Reading as Individual Difference Variables in Development 

*Christopher J. Lonigan, Ralph Radach & Christian Vorstius *

14.30 CompLex: An eye-movement database of individual differences in the 

 recognition of morphologically complex words 

*Daniel Schmidtke & Victor Kuperman *

14.50 An eye movement study of children’s pronoun processing: Individual differences 

 in detection of incongruence during reading *Sarah Eilers, Simon P. Tiffin-Richards & Sascha Schroeder *

15.10 Oculomotor control in visual tasks predicts reading skill regardless of scanning direction 

*Regina Henry, Julie A. Van Dyke & Victor Kuperman*

**, 17.00 19.00**

Symposium: Insights from Eye Movement Research with Immersive Technologies

**Room 1** (HS 14 - M.10.12) Convenors: Gordon Pipa et al.

 17.00 Using Virtual Reality to Assess Ethical Decisions in Road Traffic Scenarios: 

Applicability of Value of Life Based Models and Influences of Time Pressure 

*Gordon *

 17.20 A Virtual reality setup for intensive care unit patients while applying controlled visual and acoustic stimulation 

*Stephan Gerber *

 17.40 The influence of contextual rules on object interactions and spatial representations: a virtual reality investigation 

*Dejan Draschkow & Melissa L.-H. Vo *

 18.00 Advances in the research of anxiety and anxiety disorders using virtual reality 

*Bastian Söhnchen, Mathias Müller & Paul Pauli*

 18.20 Research on cognitive architecture of human motor performance and its application in VR environments 

*Thomas Schack & Kai Essig*

**18.40 Using closed-loop-VR to probe human visuomotor control 

*Constantin A. , Huaiyong Zhao, Julia Frankenstein & David Hoppe *

Thematic session: Pupillometry

** Room 2** (HS 32 - K.11.23) Chair: Sebastiaan Mathôt

17.00 What’s good about big pupils? 

*Sebastiaan Mathôt & Yavor Ivanov *

17.20 Attention in visual periphery: Evidence from pupillometry

*Andreas , Raphael Harbecke & Stefanie Hüttermann *

17.40 Pupil Sizes Scale with Attentional Load and Task Experience in a Multiple Object 

 Tracking Task 

*Basil Wahn, Daniel P. Ferris, W. David Hairston & Peter König *

18.00 Raven revisited: Fixation-related EEG alpha frequency band power and pupil dilation unravel fluctuations in cognitive load during task performance 

*Christian Scharinger & Peter Gerjets *

18.20 Towards pupil-assisted target selection in natural environments 

*Christoph Strauch, Greiter Lukas & Anke Huckauf*

18.40 CHAP: An Open Source Software for Processing and Analyzing Pupillometry Data 

*Ronen Hershman, Noga Cohen & Avishai Henik*

Thematic session: Learning and cognitive information processing

**Room 3** (HS 28 - I.13.71) Chair: Aline Godfroid

 17.00 The use of eye tracker in the discrimination of linguistic and image processing demands in a picture-identification task

*Letícia M. Sicuro Corrêa, Elisângela N. Teixeira & Erica dos Santos Rodrigues *

 17.20 Using eye movements to measure conscious and unconscious linguistic knowledge *Aline Godfroid, Jieun Ahn, Patrick Rebuschat & Zoltan Dienes *

 17.40 Animacy and children’s online processing of restrictive relative clauses 

*Ross G. Macdonald, Ludovica Serratrice, Silke Brandt, Anna Theakston & Elena Lieven *

 18.00 Can Eye-Mind Connection Be Broken in the Visual World Paradigm? 

*Anastasiya Lopukhina & Anna Laurinavichyute*

 18.20 Words and Images: Information Distribution in Comic Panels 

*Clare Kirtley, Benjamin W. Tatler, Christopher Murray & Phillip B. Vaughan *

 18.40 Eye-movements in wordless picture stories: Search for comprehension during bridging inference generation 

*John P. Hutson, Joseph P. Magliano & Lester C. Loschky*

Thematic session: Reading: Corpus analysis and text processing

**Room 4** (HS 26 - I.13.65) : Johanna Kaakinen

 17.00 Russian Sentence Corpus 

 Anna Laurinavichyute, Irina Sekerina, Kristine Bagdasaryan & Svetlana Alexeev 

 17.20 PoCoCo: An eye-movement corpus of graphic novel reading *Jochen Laubrock, Sven Hohenstein & Eike Richter *

 17.40 A Crosslinguistic Investigation of Eye Movements During Reading 

*Denis Drieghe, Jukka Hyönä, Xin Li, Guoli Yan, Xuejun Bai & Simon P. Liversedge *

 18.00 Fluctuations in cognitive engagement during reading: Evidence from concurrent recordings of postural and eye movements

*Johanna K. Kaakinen, Ugo Ballenghein, Geoffrey Tissier & Thierry Baccino *

 18.20 Auditory distraction by meaningful background speech during reading 

*Martin R. , Simon P. Liversedge, Daniel Rowan, Julie A. Kirkby & Bernhard Angele *

 18.40 Eye-tracking data analysis using hidden semi-Markovian models to identify and characterize reading strategies 

*Brice Olivier, Jean-Baptiste Durand, Anne Guérin-Dugué & Marianne Clausel *

^th^**, 9.00 - 11.00 **

Symposium: Interpreting and Using Visualizations of Eye Movements to Improve Task Performance and Learning

**Room 1** (HS 14 - M.10.12) Convenors: Margot van Wermeskerken et al. 

 9.00 Searching with and against each other 

*Tim Cornelissen, Diederick C. Niehorster, Ignace T.C. Hooge & Kenneth Holmqvist *

 9.20 Eye see what you are doing: Inferring task performance from eye movement data *Margot van Wermeskerken, Damien Litchfield & Tamara van Gog*

 9.40 Gaze guidance in number-line tasks 

*Damien Litchfield, Thomas Gallagher-Mitchell & Victoria Simms *

 10.00 Look where eye looked: Eye movement modeling examples enhance learning to solve geometry problems 

*Tim van Marlen, Margot van Wermeskerken, Halszka Jarodzka & Tamara van Gog *

 10.20 Using eye movement modeling examples as an instructional tool for learning with multimedia: The influence of model and learner characteristics 

*Marie-Christin Krebs, Anne Schüler & Katharina Scheiter*

**10.40 If I you where you looked, you still wouldn’t remember 

*Ellen M. Kok, Avi M. Aizenman, Melissa L.-H Võ & Jeremy M. Wolfe *

Thematic session: Oculomotor event detection

** Room 2** (HS 32 - K.11.23) Chair: Ignace Hooge

9.00 Is human classification a gold standard in fixation detection? 

*Ignace T.C. Hooge, Diederick C. Niehorster, Marcus Nyström, Richard Andersson & Roy S. Hessels *

9.20 Looking ? Model-based saccade detection on the position profile 

*David J. Mack & Federico Wadehn *

9.40 Towards Low-Latency Blink Detection Using Event-Based Vision Sensors 

*Florian Hofmann, Arren Glover, Thies Pfeiffer, Chiara Bartolozzi & Elisabetta Chicca *

10.00 Topology for gaze analyses 

*Oliver Hein *

10.20 End-to-end eye-movement event detection using deep neural networks 

*Raimondas Zemblys, Diederick C. Niehorster & Kenneth Holmqvist *

10.40 Comparing Data Evaluation Task Effects on Data Driven Event Detection Models 

*Michael Haass, Matzen Laura & Kristin Divis*

Thematic session: Usability and web-based interface design

** Room 3** (HS 28 - I.13.71) Chair: Gemma Fitzsimmons

 9.00 Fake sites through the customers' eyes *Simone Benedetto & Christian Caldato*

 9.20 Children's management on commercial websites: Effects of task type and advert prominence 

*Nils Holmberg *

 9.40 Reading for Comprehension versus Skim Reading on the Web: The Impact of Hyperlinks and Navigation 

*Gemma Fitzsimmons, Mark J. Weal & Denis Drieghe*

 10.00 Learning 3D layout from 2D views: insights from eye movement behaviour during multiplex screen viewing 

*Kenneth C. Scott-Brown, Matthew J. Stainer & Benjamin W. Tatler*^**^

 10.20 Visual attention and neural co-activation reflect conscious processing during prosthetic hand use, but only during object manipulations 

*Johnny V. V. Parr, Neil Harrison, Sam Vine, Mark Wilson & Greg Wood *

Thematic : Reading: Basic oculomotor control

** Room 4** (HS 26 - I.13.65) Chair: Françoise Vitu

 9.00 Oculomotor adaptations when reading mirror-reversed texts *André Krügel, Johan Chandra & Ralf Engbert *

 9.20 Eye Movement Control for Horizontal and Vertical English Text 

*Sha Li, Maryam A. AlJassmi, Kayleigh L. Warrington, Sarah J. White, Jingxin Wang, Mercedes Sheen et al. *

 9.40 How MASC, a Model of Attention in the Superior Colliculus, pretends to read despite being completely illiterate! 

*Françoise Vitu, Hossein A. Gregory & J. Zelinsky*

 10.00 Eye-Movement Evidence for Object-Based Attention in Reading 

*Yanping & Erik D. Reichle*^**^

 10.20 The impact of forced fixations on word recognition: Dissociation of oculomotor behavior and linguistic processing

*E. R. Schotter, Mallorie Leinenger & Titus von der Malsburg *

 10.40 Word demarcation in reading of newly learned strings: There’s something special about spaces 

*Mengsi Wang, Hazel I. Blythe & Simon P. Liversedge*

^th^**, 11.30 - 13.30 **

Symposium: Influences on Voluntary Oculomotor Control

** Room 1** (HS 14 – M.10.12) Convenor: Jakob Heinzle & Ulrich Ettinger 

11.30 Effects of NMDA antagonists on voluntary control of eye movements in non-human primates 

*Pierre Pouget & Marcus Missal *

11.50 Effects of Ketamine on Brain Function during Smooth Pursuit and Antisaccade Eye Movements in Healthy Humans 

*Maria Steffens, Anna Kasparbauer, Inga Meyhöfer, René Hurlemann & Ulrich Ettinger *

12.10 Neuropharmacology of cognitive control: local manipulations of the dopaminergic 

and cholinergic system in monkey prefrontal cortex during antisaccade performance 

*Susheel , Alex James Major & Stefan Everling*

12.30 Model based analysis of dopaminergic and cholinergic neuromodulation on voluntary control of eye movements in humans

*Jakob Heinzle, Dario Schöbi, Klaas Enno Stephan & Eduardo A. Aponte *

12.50 Cholinergic and Dopaminergic Influences on Eye Movements in Humans 

*Ulrich Ettinger, Anna Kasparbauer, Maria Steffens, Inga Meyhöfer, Eliana Faiola *

 & Nadine Petrovsky 

13.10 General discussion **

Thematic session: Saccade programming II

** Room 2** (HS 32 - K.11.23) Chair: Hans Trukenbrod 

11.30 Dissociating automatic capture, to individual stimuli or the global effect location, from intentional saccade targeting

*David Aagten-Murphy & Paul M. Bays *

11.50 Asymmetries of the saccadic system: A tool to quantify eye dominance strength 

*Jérôme Tagu, Karine Doré-Mazars, Christelle Lemoine-Lardennois, Judith Vergne & Dorine Vergilino-Perez*

12.10 Saccade countermanding reflects automatic inhibition as well as top-down cognitive control 

*Aline Bompas, Annie Campbell & Petroc Sumner*

12.30 Oculomotor gap effect and antisaccade performance in the common marmoset 

*Kevin & Stefan Everling *

12.50 Control of fixation durations in a visually guided task

*Hans A. Trukenbrod & Jan* *Grenzebach *

13.10 Adaptation of post-saccadic drift in reflexive saccades does not transfer to voluntary saccades *Giulia Manca & Heiner Deubel*

Thematic session: Applied visual cognition

** 3** (HS 28 - I.13.71) Chair: Andrew K. Mackenzie

 11.30 Eye movements during lifeguard visual search for a drowning swimmer *Victoria Laxton, David Crundall, Christina Howard & Duncan Guest *

 11.50 Multiple Object Avoidance (MOA): A more sensitive measure of visual attention in the real world 

*Andrew K. Mackenzie, Paul R. Cox, Christina Howard, Duncan Guest & David Crundall*

 12.10 The (Change) Blindingly Obvious: Investigating Fixation Behaviour during CCTV Observation 

*Gemma Graham, James Sauer, Jenny Smith, Lucy Akehurst & James Ost * 12.30 Eye movements during perspective-taking in younger and older adults 

*Victoria E. A. Brunsdon, Elisabeth E. F. Bradford & Heather Ferguson*

 12.50 Using eye-tracking to study how belief-reasoning processes change across the lifespan 

*Elisabeth E. F. Bradford, Victoria E. A. Brunsdon, Heather Ferguson*

 13.10 An -tracking investigation of mindset effects on information search in incentivized decisions under uncertainty

*Jonas Ludwig, Alexander Jaudas & Anja Achtziger*****

Thematic session: Reading: Word level processing

** Room 4** (HS 26 - I.13.65) Chair: Heather Sheridan

 11.30 Raeding transposde etxt: Effects of letter position, word frequency and constraint *Christopher James Hand, Joanne Ingram & Graham Scott *

 11.50 Morphological guidance of eye movements during reading *Jukka Hyönä, Seppo Vainio & Timo Heikkilä*

 12.10 Morphological in sentence reading: Evidence from the fast priming paradigm 

*Betty Mousikou & Sascha Schroeder*

 12.30 Distributional analyses of age of acquisition effects on fixation durations during reading 

*Heather Sheridan & Barbara J. Juhasz*^**^

 12.50 Eye movements during lexical access of a third language

*Pâmela Freitas Pereira Toassi, Mailce B. Mota & Elisângela N. Teixeira *

 13.10 Learning new words when reading: effects of contextual diversity and temporal spacing 

*Ascensión Pagán & Kate Nation*

^th^**, 14.30 - 16.30 **

Symposium: , eye movements and vision 50 years on

** Room 1** (HS 14 – M.10.12) Convenor: Benjamin W. Tatler

14.30 Yarbus on stationary retinal images and moving eyes

*Nicholas Wade *

14.50 The evolution of gaze analysis tools to support complex tasks 

*Jeff B. Pelz *

15.10 Computational modeling of gaze guidance during scene free viewing and daily 

 tasks 

*Ali *

15.30 Eye guidance in scenes: Object-based selection in extrafoveal vision 

*Antje Nuthmann *

15.50 Characterising top-down guidance of fixation in scenes and objects 

*Tom Foulsham *

16.10 The balance between the stimulus and the task in determining the scanpath 

*Iain Gilchrist *

Thematic session: Clinical Research II

** Room 2** (HS 32 - K.11.23) Chair: Valerie Benson

14.30 Processing of Co-Reference in Autism Spectrum Disorder

*Philippa L. Howard, Simon P. Liversedge & Valerie Benson*

14.50 How does the presence or absence of a Title Modulate Processing of Ambiguous 

Passages in Individuals with Autism: An Eye Movement Study

*Valerie Benson, Philippa Howard & Johanna Kaakinen*

15.10 Inhibitory control for emotional and neutral scenes in competition: An eye- tracking study in bipolar disorder 

*Manuel Perea, Ladislao Salmerón & Ana García-Blanco*

15.30 Smooth in Adults with Developmental Dyslexia

*Gillian O'Driscoll, Veronica Whitford, Ashley Chau-Morris & Debra Titone *

15.50 Visual field diagnostics with eye tracking: development and neuropsychological testing of a new diagnostic tool 

*Michael Christian Leitner, Constanze Haslacher, Stefan Hawelka, Lorenzo Vignali, *

 Sarah Schuster & Florian Hutzler 

16.10 Calibrating an eye tracker for blind patients implanted with the Argus II retinal 

 prosthesis using a handheld marker 

*Avi Caspi, Jessy D. Dorn, Arup Roy, Robert J. Greenberg*

Thematic : Eye data analysis and evaluation

** Room 3** (HS 28 - I.13.71) Chair: Laura Matzen

 14.30 SMAC with HMM: a toolbox to model and classify scanpaths with Hidden Markov Models 

*Antoine Coutrot *

 14.50 Gaze Self-similarity Plots as a useful tool for eye movement characteristics analysis 

*Pawel Kasprowski & Katarzyna Harezlak *

 15.10 Towards to an automatic authentication method based on eye movement by using scanpath comparison algorithms**

*Carlos-Alberto Quintana-Nevárez & Francisco López-Orozco* 15.30 Magnitude and Nature of Variability in Eye-tracking Data

*Kenneth , Raimondas Zemblys & Tanya Beelders*

 15.50 Effects of Task on Eye Movements During Comprehension of Abstract Data Visualizations 

*Laura Matzen, Kristin Divis & Michael Haass*****

Thematic session: Reading: Across the lifespan

** Room 4** (HS 26 - I.13.65) Chair: Kevin Paterson

 14.30 Syllables vs. morphemes in early reading of Finnish *Tuomo Häikiö & Seppo Vainio*

 14.50 Words the wizarding world: Reading fictional words in supportive and nonsupportive contexts 

*Joanne Ingram & Christopher J. Hand *

 15.10 Re-Assessing Adult Age Differences in the Perceptual Span during Reading 

*Kevin Paterson, Kayleigh Warrington, Sarah White & Victoria McGowan *

 15.30 Adult Age Differences in Chinese Reading: Effects of Character Complexity 

*Jingxin Wang, Lin Li, Sha Li, Yingying Zhang & Kevin Paterson *

 15.50 Aging and the Misperception of Words during Reading

*Kayleigh L. Warrington, Sarah J. White, Victoria A. McGowan & Kevin B. Paterson *This work is licensed under a

Poster

Session I -Monday, 21^st^, 15.30 - 17.00 

**Attention and visual information processing**

I-1 Gaze-contingent stimulus removal leads to subsequent changes in attentional allocation

 Karin Ludwig, Doris Schmid & Thomas Schenk

I-2 The relationship between subjective time perception and visual attention * Maria Konstantinova, Leonid Tereshchenko, Viktor Anisimov & Alexander Latanov*

I-3 Rapid top-down and bottom-up auditory attention as reflected by (micro-)saccadic inhibition

 Andreas Widmann, Alexandra Bendixen, Susann Duwe, Ralf Engbert, Erich Schröger & Nicole Wetze

I-4 Pre-saccadic remapping of foveal attention

 Meng Fei Ngan, Luca Wollenberg, Heiner Deubel & Martin Szinte

I-5 Saccade deviation and saccadic reaction time: What is the relationship?

 Luke Tudge & Torsten Schubert

I-6 Can you squint on command? No reliable voluntary control and awareness of eye vergence in the absence of an actual target

 Sonja Walcher, Christof Körner & Mathias Benedek

I-7 Maintaining in a fixation task: Are stimuli at all eccentricities equally effective?

 Anna-Katharina Hauperich & Hannah E. Smithson

I-8 Extrafoveal perception of geometric shapes in adults and children

 Anatoly N. Krichevets, Dmitry V. Chumachenko, Anna A. Dreneva & Anna Yu. Shvarts

I-9 What can and what cannot be perceived extrafoveally

 Anna A. Dreneva, Anna Yu Shvarts, Dmitry V. Chumachenko & Anatoly N. Krichevets

I-10 Attention and response speed in pupil old/new effects

 Tim & Andreas Brocher

I-11 Effect of aging on ocular fixation and microsaccades during optic flow

 Marcia Bécu, Guillaume Tatur, Alix de Dieuleveult, Changmin Wu, Silvia Marchesotti, Denis Sheynikhovich & Angelo Arleo

I-12 Saccadic adpatation increases brain excitability: a MEG study

 Judith Nicolas, Aline Bompas, Romain Bouet, Olivier Sillan, Eric Koun, Christian Urquizar, Alessandro Farnè, Aurélie Bidet-Caulet & Denis Pélisson

I-13 Localization of briefly flashed targets across sequential eye-movements

 Janne Aswegen, Stefan Dowiasch & Frank Bremmer

I-14 The influence of threat associated distractors on express saccades

 Jessica Heeman, Stefan Van der Stigchel & Jan Theeuwes

I-15 Stereoacuity in the temporal proximity of vergence movements

 Thomas Eggert

I-16 A Tool-based Process for Generating Attention Distribution Predictions

 Sebastian Feuerstack & Bertram Weutelen 

**Reading: Visual and orthographic processing **

I-17 Statistical Estimation of Oculomotor Processes During Reading *Johan Chandra, André Krügel & Ralf Engbert *

I-18 Contrast change effects reveal time course of parafoveal processing in eye movements during reading 

*Tina Andrea Schlachter & Sarah Risse *

I-19 Gaze-contingent unmasking of filtered text regions during reading of graphic literature 

*Sven Hohenstein, Jochen Laubrock & Eike M. Richter*

I-20 The effect of misspellings on reading of correctly spelled words, across paradigms and languages 

*Victor Kuperman & Sadaf Rahmanian *

I-21 Reading at the speed of speech: Convergence between visual and auditory language perception at 4-5 Hz? 

*Benjamin Gagl, Julius Golch, Stefan Hawelka, Jona Sassenhagen, David Poeppel & *

Christian J. Fiebach 

I-22 Effective visual field of horizontal and vertical reading in Japanese 

*Nobuyuki Jincho *

I-23 The perceptual span of young and older Chinese readers

*Victoria A. McGowan, Kayleigh L. Warrington, Lin Li, Sha Li, Yingying Zhang, Yuxiang Yao, Jingxin Wang, Sarah J. White & Kevin B. Paterson *

I-24 Effects of Aging and Pattern Complexity on the Visual Span of Chinese Readers 

*Kayleigh L. Warrington, Lin Li, Fang Xie, Sha Li, Jingxin Wang, Victoria A. McGowan, *

Sarah J. White & Kevin B. Paterson 

I-25 Adult Age Differences in Eye-Guidance during Chinese Reading *Lin Li, Sha Li, Jingxin Wang, Yuxiang Yao & Kevin B. Paterson *

I-26 Eye Movement Control and Word Identification During Vertical and Horizontal Reading: Evidence from Mongolian 

*Kevin , Juan Su, Guoen Yin, Xuejun Bai, Guoli Yan & Simon P. Liversedge *

I-27 The Perceptual Span during Vertical and Horizontal Reading: Evidence from Mongolan 

*Kevin Paterson, Juan Su, Guoen Yin, Stoyan Kurtev, Simon P. Liversedge, Bai Xuejun & Guoli Yan *

I-28 Investigating word length in Chinese reading: Evidence from eye movements *Chuanli Zang, Ying Fu & Simon P. Liversedge*

I-29 The last, but not the initial character’s positional frequency affects Chinese compound word processing in reading 

Feifei Liang, Qi Gao, Jie Ma, Hao Wu & Xuejun Bai

I-30 The of spaces in segmenting Finnish and Chinese text 

*Raymond Bertram, Liyuan He & Simon P. Liversedge*

I-31 Vertical movement within fixations in the reading of Chinese and English 

*Yi-ting Hsiao, Richard Shillcock, Mateo Obregón, Hamutal Kreiner, Matthew A.J. Roberts & Scott McDonald *

I-32 When readers pay attention to the left: A concurrent eyetracking-fMRI investigation on the neuronal correlates of regressive eye movements during reading 

*Anna F. Weiß, Franziska Kretzschmar, Arne Nagels, Matthias Schlesewsky, Ina Bornkessel-Schlesewsky & Sarah Tune *

**Developmental Eye Movement Research **

I-33 Fetal eye movements in response to a visual stimulus

*Tim Donovan, Kirsty Dunn, Sophie Clarke, Anna Gillies, Olivia Mercer & Vincent Reid *

I-34 Early regulatory problems associated with the affect-biased attention at 8 month of age 

*Eeva Eskola, Riikka Korja, Eeva-Leena Kataja, Linnea Karlsson, Tuomo Häikiö, Henri Pesonen, Jukka Hyönä & Hasse Karlsson*

I-35 Maternal Prenatal stress and Iinfant attention to emotional faces at the age of eight months months in finnbrain birth cohort

*Eeva-Leena Kataja, Linnea Karlsson, Henri Pesonen, Jukka Leppänen, Tuomo Häikiö, Jukka Hyönä, Christine Parsons & Hasse Karlsson *

I-36 Infant -viewing: the role of object knowledge

*Daan van Renswoude, Maartje Raijmakers, Roos Voorvaart & Ingmar Visser *

I-37 Development of oculomotor control from infants to toddlers: temporal and spatial parameters of voluntary saccades 

*Christelle Lemoine-Lardennois, Nadia Alahyane, Mallaury Hamon, Clara Ferrari & Karine Doré-Mazars *

I-38 Individual differences in children’s learning through eye-tracking experiment 

*Dmitry Chumachenko, Anna Shvarts & Anna Dreneva*

I-39 Exploring the development of oculomotor attentional control in emotional and nonemotional contexts 

*Athina Manoli, Simon P. Liversedge, Edmund Sonuga-Barke & Julie A. Hadwin *

I-40 Development of eye movements related to executive functions in elementary school students 

*Suxia Wang, Ralph Radach, Christian Vorstius, Yan Sun & Lizhu Yang *

**Developmental research on eye movements in reading **

I-41 Patterns of 5-6 year old children reading picture book: Evidence from eye movements 

*Yuanyuan Sun, Peng Wan & Guiqin Ren *

I-42 The perceptual span of second graders in Chinese primary school 

*Guoli , Sainan Li, Min Liu & Yali Wang*

I-43 Reading Instructions Influence Cognitive Processes of Illustrated Text Reading for Young Readers: An Eye-Tracking Study

*Yu-Cin Jian *

I-44 The eye-tracking study of reading in Russian primary schoolchildren 

*Aleksei Korneev, Ekaterina Matveeva, Tatyana Akhutina*

I-45 Eye-tracking study of reading the texts of different types: Evidence from russian 

*Tatiana Petrova *

I-46 The Correlation between Eye Movement Data and Three Commonly Used Academic Reading Assessments 

*Alexandra Spichtig, Kristin Gehsmann, Jeffrey Pascoe & John Ferrara *

I-47 Effects of Scaffolded Silent Reading Practice on the Reading Related Eye Movements of US Students in Grades 4 and 5

*Kristin Gehsmann, Alexandra Spichtig, Jeffrey Pascoe & John Ferrara *

I-48 Relationship Between Students’ Stages of Orthographic Knowledge and Reading Efficiency 

*Kristin Gehsmann, Elias Tousley, Alexandra Spichtig, Jeffrey Pascoe & John Ferrara *

I-49 The of Reading Efficiency Measures Obtained by Classroom Educators 

Using a Low-Cost Eye Movement Recording System 

*Alexandra Spichtig, Jeffrey Pascoe & John Ferrara*

I-50 What can we reveal from saccade events of eye movements when EFL high school students read narrative with illustration?

*Grace Ming-Yi Hsieh & Sunny San-Ju Lin *

**Usability, New Media and Visual Communication **

I-51 Situational Modulation of Multimedia Processing Strategies

 Fang , Wolfgang Schnotz, Inga Wagner & Robert Gaschler 

I-52 Extraction of Semantic Saliency on Memory and Remembering during reading/searching information in the context of Web interaction

*Véronique Drai-Zerbib & Thierry Baccino *

I-53 Typography and individual experience in digital reading: Do readers’ eye movements adapt to poor justification? 

*Julian Jarosch, Matthias Schlesewsky, Stephan Füssel & Franziska Kretzschmar *

I-54 A contrastive perception study of popular-scientific texts written by journalists vs. researchers 

*Silvia Hansen-Schirra, Jean Nitzke, Anke Tardel, Christoph Böhmert & Philipp Niemannl *

I-55 Eye Response to Blockiness Artifacts in Video 

*Deepti Pappusett & Hari Kalva *

I-56 Personalization in online advertising: Effects of demographic targeting on visual attention 

*Kai Kaspar, Sarah Lucia Weber & Anne-Kathrin Wilbers*

I-57 Attention to brand logos during the first exposure to advertisements affects the neural correlates of recognition memory: An eye movement – ERP study 

*Jaana Simola *

I-58 Eye Markers in Perceiving of Logos 

*Adel Adiatullin, Marina Koroleva, Victor Anisimov, Alexander Latanov & Natliya Galkina *

I-59 Understanding use of labelling information when preparing infant formula: an eyetracking study 

*Lenka Malek, Hazel Fowler & Gillian Duffy*

I-60 Visual intake of price information of organic food – a shopping task with Eye-Tracking Glasses 

*Manika Rödiger & Ulrich Hamm *

I-61 The ‘objectfiying gaze’ - how it is affected by information on distribution of sexting images 

*Frederike Wenzlaff, Briken Peer & Dekker Arne*

I-62 Speed transformation function as a mean of improvement of gaze-based HCI 

*Dominik Chrząstowski-Wachtel, Cezary Biele, Marek Młodożeniec, Anna Niedzielska, Jarosław Kowalski, Paweł Kobyliński, Krzysztof Krejtz & Andrew T. Duchowski *

I-63 Investigating gaze-controlled input in a cognitive selection test 

*Katja Gayraud, Catrin Hasse, Hinnerk Eißfeldt & Sebastian Pannasch *

I-64 The effect of visual signaling when reading to do

*Michael Meng *

I-65 Eye-Tracking-Based Guidance in Mobile Augmented Reality Assistance Systems 

*Patrick Renner & Thies Pfeiffer *

I-66 Usability Heuristics for Eye-Controlled User Interfaces

*Korok Sengupta, Chandan Kumar & Steffen Staab*

I-67 CrowdPupil: A crowdsourced, pupil-center annotated image dataset 

*David Gil de Gómez Pérez & Roman Bednarik*

I-68 Robust, real-time eye movement classification for gaze interaction using finite state machines 

*Antonio -Tula & Carlos H. Morimoto*

I-69 Supervised Gaze Bias Correction for Gaze Coding in Interactions 

*Rémy Siegfried & Jean-Marc Odobez *

I-70 Schau genau! A Gaze-Controlled 3D Game for Entertainment and Education 

*Raphael Menges, Chandan Kumar, Ulrich Wechselberger, Christoph Schaefer, Tina Walber & Steffen Staab *

**Social Cognition, emotion and cultural factors **

I-71 A for Exploring the Social Gaze Space

*Arne Hartz, Mathis Jording, BjörnGuth, Kai Vogeley & Martin Schulte-Rüther *

I-72 Visual Exploration of Social Stimuli – Comparisons of Patients with ADHD or Autism and Healthy Controls 

*Chara Ioannou, Divya Seernani, Holger Hill, Giuseppe Boccignone, Tom Foulsham, *

Monica Biscaldi-Schäfer, Christopher Saville, Ulrich Ebner-Priemer, Christian Fleischhaker & Christoph Klein

I-73 Eye movement patterns in response to social and non-social cues 

*Claudia Bonmassar, Francesco Pavani & Wieske van Zoest*

I-74 Oculomotor action control in social and non-social information processing contexts 

Eva Riechelmann, Anne Böckler, Tim Raettig & Lynn Huestegge 

I-75 Understanding social interaction and social presence of others using simultaneous eye tracking of two people: Behavioral Data

*Haruka Nakamura, Seiya Kamiya & Takako Yoshida*

I-76 Gender differences in natural viewing behavior?

*Marco Rüth, Anne-Kathrin Wilbers, Daniel Zimmermann & Kai Kaspar *

I-77 Does our native language determine what we pay attention to? A cross-linguistic study of gaze behaviour between Korean and German speakers 

*Florian Goller, Ulrich Ansorge & Soonja Choi*

I-78 Social influence on face perception in different ethnicities – An eye tracking study in a free viewing scenario

*Jonas D. Großekathöfer & Matthias Gamer *

I-79 Psychopaths show a reduced tendency to look at the eyes while categorizing emotional faces 

*Nina A. Gehrer, Jonathan Scheeff, Aiste Jusyte & Michael Schönenberg *

I-80 Perceiver’s sensitivity and lateralization bias in the detection of posed and genuine facial emotions in movie clips: eye tracking study 

*Katerina Lukasova, Yuri Busin, Manish K. Asthana & Elizeu C. Macedo *

I-81 Implicit Affect Predicts Attention for Sad Faces beyond Self-Reported Depression – An Eye Tracking Study 

*Charlott M. Bodenschatz, Marija Skopinceva, Anette Kersting & Thomas Suslow *

I-82 Gender differences in eye movement patterns during facial expression recognition *Elizaveta Luniakova, Natalia Malysheva & Jahan Ganizada *

I-83 Analyzing Emotional Facial Expressions’ Neural Correlates Using Event-Related Potentials and Eye Fixation-Related Potentials

*Emmanuelle Kristensen, Raphaëlle N. Roy, Bertrand Rivet, Anna Tcherkassof & Anne Guérin-Dugué *

I-84 Affective and Cognitive Influences of Aesthetic Appeal of Texts on Oculomotor Parameters 

*Hideyuki Hoshi *

I-85 The eye movement examination on achievement emotion images

*Chia Yueh Chang & Sunny SJ. Lin *

I-86 Space scanning patterns in impulsive and reflective subjects 

*Anna Izmalkova & Irina Blinnikova *

I-87 Correlations between eye movements and personality traits

*Anne-Kathrin Wilbers & Kai Kaspar *

Session II - Tuesday, August 22^nd^, 15.30 - 17.00 

**Smooth pursuit eye movements **

II-1 Saliency coding in superior colliculus during smooth pursuit eye movements *Brian White, Jing Chen, Karl Gegenfurtner & Douglas Munoz *

II-2 Analysis of superior colliculus receptive fields during smooth pursuit eye movements *Jing Chen, Brian White, Karl Gegenfurtner, & Doug Munoz *

II-3 Doing Smooth Pursuit paradigms in Windows 7 

*Inge L. Wilms *

II-4 Predictable motion on a Necker cube leads to micro-pursuit-like eye movements and affects the dynamics of bistability.

*Kevin M. Ashwin Parisot, Alan Chauvin, Anne Guérin, Ronald Phlypo & Steeve Zozor *

II-5 Manual & Automatic Detection of Smooth Pursuit in Dynamic Natural Scenes *Mikhail Startsev, Ioannis Agtzidis & Michael Dorr *

II-6 Spatiotemporal EEG Source Localization during Smooth Pursuit Eye Movement by Use of Equivalent Dipole Source Localization Method 

*Takahiro Yamanoi, Tomoko Yonemura & Hisashi Toyoshima*

II-7 Visual transient onsets decrease initial smooth pursuit velocity and inhibit the triggering of catch-up saccades 

*Antimo & Ziad M. Hafed *

**Visual Search, Scanpaths and Scene Perception **

II-8 Searching for real objects in a natural environment: The role of contextual semantic cues and incidental encoding in older and young viewers 

*Hanane Ramzaoui, Sylvane Faure & Sara Spotorn*

II-9 Dwelling, Rescanning, and Skipping of Distractors Explain Search Efficiency in Difficult Search: Evidence from Large Set Sizes and Unstructured Displays 

*Gernot Horstmann, Stefanie Becker & Daniel Ernst*

II-10 The of changing the item relevance in repeated search 

*Sebastian A. Bauch, Christof Körner, Iain D. Gilchrist & Margit Höfler *

II-11 Target and distractor guidance in repeated visual search: When using memory does not improve search 

Margit Höfler, Iain D. Gilchrist, Anja Ischebeck & Christof Körner 

II-12 Process Analysis of Visual Search in ADHD, Autism and Healthy Controls – Evidence from Intra- Subject Variability in Gaze Control. 

*Divya P. Seernani, Holger Hill, Giuseppe Boccignone, Tom Foulsham, Christian Fleischhaker, Monica Biscaldi,* *Ulrich Ebner-Priemer & Christoph Klein *

II-13 When one target predicts the other: Target guidance in visual search 

*Christof , Jonas Potthoff, Ulrich Ebner-Priemer & Christoph Klein *

II-14 Does context influence the low prevalence effect in visual search? 

*Titus N. Ebersbach, Walter R. Boot & Ralph Radach*

II-15 Simulation of visual hemi-neglect by spatio-topic and retino-topic manipulation of visual search displays 

*Jennifer Winter, Björn Machner, Inga Könemund, Janina von der Gablentz, Christoph Helmchen & Andreas Sprenger *

II-16 Where can I find the Honey, Honey? Using color cues to overwrite syntactic rules in a scene-search paradigm 

*Marian D. Laukamp, Lisa Völker, Sabine Öhlschläger & Melissa Le-Hoa Vo *

II-17 Time course of central and peripheral processing during scene viewing 

*Anke Cajar, Ralf Engbert & Jochen Laubrock*

II-18 Central fixation bias: The role of sudden image onset and early gist extraction *Lisa F. Schwetlick, Lars O. M. Rothkegel, Hans A. Trukenbrod & Ralf Engbert *

II-19 Eye movements in scene perception during quiet standing

*Daniel Backhaus, Hans A. Trukenbrod, Lars O. M. Rothkegel, Ralf Engbert *

II-20 Gaze Paths on a Stochastic Image 

*Miriam & Emiliano Melchiorre *

II-21 Eye movements and saliency for the Hollywood2 action recognition benchmark 

*Michael Dorr & Eleonora Vig *

II-22 Cultural variation in eye movements during scene perception: replication with a Russian sample 

*Anton Gasimov & Artem Kovalev *

II-23 The influence of verbalization on eye movement parameters during complex scene repeated viewing 

*Veronika Prokopenya & Ekaterina Torubarova*

**Clinical Research **

II-24 EyeGrip as a tool for assessing dementia 

*Diako Mardanbegi, Shahram Jalaliniya, Hans Gellersen, Trevor J. Crawford & Peter Sawyer *

II-25 Executive function processes in dementia: Impairments in anti-saccadic eye movements are indicative for first disease stages

*Lucas Paletta, Martin Pszeida & Mariella Panagl*

II-26 Eye movement behavior in MCI and AD:using automatic classification algorithms to identify cognitive decline 

*Marta L. G. F. , Marina von Zuben de Arruda Camargo, Ariella F. Belan, Bernardo dos Santos & Orestes V. Forlenza*

II-27 Eye Movement Parameters while Executing Oculomotor Tasks in Patients with Cerebellum Tumor 

*Marina Shurupova, Viktor Anisimov, Alexander Latanov & Vladimir Kasatkin *

II-28 GENERAIN – a transgenerational eye-tracking study on attention biases in children at risk for depression 

*Anca Sfärlea, Elske Salemink, Gerd Schulte-Körne & Belinda Platt *

II-29 Saccadic inhibition and its interaction with implicit processing of emotion in Bipolar Disorder patients 

*Nathalie Guyader, Alan Chauvin, Lysianne Beynel, Sylvain Harquel, Cécilia Neige & Mircea Polosan *

II-30 Utilizing Eye-Movement Patterns for Improving ADHD Diagnosis and Malingering Detection 

*Michael Wagner, Corinne Berger, Yoram Braw, Tomer Elbaum & Tzur Chohen *

II-31 Parafoveal processing Efficiency in Chinese developmental dyslexia: Evidence from RAN tasks 

*Wen Wang, Ke Tan, Mingzhe Zhang & Xuejun Bai*

II-32 Investigating the effects of orthographic visual complexity on fixations in typical and dyslexic reading of English

*Rea Marmarinou, Jun Bao, Richard Shillcock, Mateo Obregón, Hamutal Kreiner, Matthew A.J. Roberts & Scott McDonald*

II-33 The of eye tracking in the assessment and therapy of acquired dyslexia 

*Irene Ablinger & Ralph Radach *

II-34 A visuomotor analysis of multilevel therapy in pure alexia

*Anne Friede, Irene Ablinger & Ralph Radach*

II-35 Eye movements in text reading in a patient with incomplete Bálint`s syndrome 

*Katja Halm, Ralph Radach & Irene Ablinger*

II-36 Localizing hemianopic visual field defects based on natural viewing behavior while watching movie clips 

*Birte Gestefeld, Alessandro Grillini, Jan-Bernard C. Marsman & Frans W. Cornelissen *

II-37 Visual search behaviours in dementia-related visual impairment in controlled realworld settings 

*Ayako Suzuki, Keir Yong, Ian McCarthy, Tatsuto Suzuki, Dilek Ocal, Nikolaos Papadosifos, Derrick Boampong, Nick Tyler & Sebastian Crutch *

II-38 Playing games with your eyes: using gaze for intervention and outcome assessment in ASD 

*Leanne Chukoskie & Jeanne Townsend *

II-39 Novel steps for online eye-gaze contingent attention training: A mouse-based moving window approach 

*Alvaro Sanchez, Jill Van Put & Ernst Koster*

**Reading: -level processing **

II-40 Sentence to image priming of gender information. Can eyetracking data shed more light on priming effects? 

*Anton Öttl, Ute Gabriel, Dawn Marie Behne, Pascal Gygax & Jukka Hyönä *

II-41 How L2 instruction influences eye-movements during reading: a within-participant study of English learners 

*Daniel Schmidtke , Amy-Beth Warriner, Victor Kuperman & Anna Moro *

II-42 Metaphor comprehension in English as an additional language learner (EALL): evidence from eye movements 

*Annina Kristina Hessel, Victoria Murphy & Kate Nation*

II-43 Using Eye Movements to Investigate Cross-Language Syntactic Activation During Natural Reading 

*Naomi Vingron, Jason Gullifer, Veronica Whitford, Deanna Friesen, Debra Jared & Debra Titone *

II-44 Reading first and second language comprehension texts in Sepedi and English among senior phase learners 

*Pheladi F. Fakude *

II-45 Selective Attention of Second Language Readers

*Caleb Prichard & Andrew Atkins *

II-46 Task reveal cognitive flexibility responding to readers' level and word frequency: Evidence from eye movements for Chinese-English bilinguals during English reading 

*Xin Li, Haichao Li, Jingyao Liu, Yongsheng Wang, Xuejun Bai & Guoli Yan *

II-47 How EFL beginners and intermediate level students read story structures along with illustrations via eye-tracking techniques

*Grace Ming-Yi Hsieh & Sunny San-Ju Lin *

II-48 The influence of location information and word frequency on Chinese poly morphemic word recognition 

*Erjia Xu & Xue Sui *

II-49 Literal and Figurative Language Processing: Evidence from Bilingual Sentence Reading 

*Danielle dos Santos Wisintainer & Mailce B. Mota*

II-50 Reading and topic scanning in English and Chinese: Effects of word frequency and spacing 

*Sarah J. White, Yaqi Wang & Xue Sui *

II-51 Eye movements in reading global and local syntactic ambiguity in Russian 

*Victor Anisimov, Olga Fedorova, Leonid Tereschenko & Alexander Latanov *

II-52 Effects of counterargument construction instruction and viewpoint presentation order on reducing myside bias in reading texts regarding controversial issues 

*Miao-Hsuan Yen & Ying-Tien Wu *

II-53 The of Tasks and Signals on Text Processing for Readers with Different Strategies 

*Shouxin Li, Dexiang Zhang, Zhaoxia Zhu & Yuwei Zheng*

II-54 Eye movement correlates of absorbed literary reading

*Moniek Kuijpers & Sebastian Wallot *

II-55 The role of defaultness and personality factors in sarcasm interpretation: Evidence from eye-tracking during reading 

*Ruth Filik, Hannah Howman, Christina Ralph-Nearman & Rachel Giora *

**Cognition and Learning **

II-56 Lab - Field Comparisons on Intra-Subject Variability of Eye Movements 

*Divya P. Seernani, Holger Hill, Chara Ioannou, Nadine Penkalla, Giuseppe Boccignone, Tom Foulsham,* *Christian Fleischhaker, Monica Biscaldi, Ulrich Ebner-Priemer & Christoph Klein*

II-57 Smart Detection of Driver Distraction Events 

*William David Clifford, Catherine Deegan & Charles Markham *

II-58 The Influence of Light-Induced Dynamics on Attention, Perception, and Driving Behavior: A Real-World Driving Study 

*Markus Grüner, Peter Hartmann, Ulrich Ansorge & Christian Büsel *

II-59 Investigating Processing with Eye Tracking

*Kim Dirkx, Jarodzka Halszka & Desiree Joosten-ten Brinke*

II-60 Sleep deprivation systematically changes eye movement characteristics 

*Justine Winkler, Ricardo Ramos Gameiro, Peter König, Daniel Aeschbach & Christian Mühl *

II-61 Applying head-mounted eye-tracking to investigate cultural differences in real-world face scanning 

*Jennifer X. Haensel, Tim J. Smith & Atsushi Senju*

II-62 Presentation Parameters Affecting Effects in the Visual World Paradigm 

*Marie-Anne & Constanze Vorwerg*

II-63 Predicting Information Context Processing from Eye movements 

*Saurin S. Parikh, Hari Kalva *

II-64 Confidence in perceptual judgments preceding eye movements

*Monique Michl & Wolfgang Einhäuser *

II-65 The relationship between performance in the anti-saccade task and memory for paintings 

*Tobiasz Trawinski, Natalie Mestry, Simon P. Liversedge & Nick Donnelly *

II-66 A closer look at numbers in simultaneous interpreting: An eye-tracking study 

*Pawel Korpal & Katarzyna Stachowiak *

II-67 Is parallel language activation modulated by simultaneous interpreting expertise? 

*Laura Keller *

II-68 Can you see what I’m saying? Eye movements and bilingual spoken language processing in conference interpreting 

*Katarzyna Stachowiak *

II-69 Evidencing emergence of sensorimotor structures underlying proportional reasoning 

*Shakila Shayan, Loes Boven, Arthur Bakker, Marieke van der Schaaf & Dor Abrahamson *

II-70 From lenses movement to cognitive processes: What new insight may eye tracking provide 

*Gustavo Gasaneo, Maria L. Freije, Juan I. Specht, Adrian A. Jimenez Gandica, Claudio A. Delrioux, Borko Stosic & Tatijana Stosic*

**Methods, Software and innovative Technology **

II-71 Statistical analysis of eye movement sequences using spatial point processes 

*Anna-Kaisa Ylitalo *

II-72 Study of and saccades when viewing holograms, stereo images, and 2D images 

*Taina M. Lehtimäki, MikkoNiemelä, Risto Näsänen, Ronan G. Reilly & Thomas J. Naughton *

II-73 Using gaze data to evaluate text readability: a multi task learning approach 

*Ana V. Gonzalez-Garduño *

II-74 Parsing Pupil and Eyeball Movement in Camera-based Eye-tracker Output 

*Jun Bao & Richard Shillcock *

II-75 Extracting Saccade-to-fixation Trajectory From Eye Movement Data in Reading 

 Jun & Richard Shillcock 

II-76 Data-driven Gaze Event Classification for the Analysis of Eye and Head Coordination By Natural Task. 

*Gabriel J. Diaz, Reynold Bailey, Chris Kanan, Mychal Lipson, Jeff Pelz & Rakshit Kothari *

II-77 Assessment of Two Low Cost Eye Trackers 

*Shanmugaraj Madasamy *

II-78 Mobile eye tracking: Reliability in assessing saccadic eye movements in reading 

*Alexander Leube, Katharina Rifai & Siegfried Wahl*

II-79 Is There a “Paperback” Option in the Domain of Eye Trackers? A New Approach for Comparing Devices 

*Agnes Scholz, Johannes Titz & Peter Sedlmeier*

II-80 What to expect from your remote eye-tracker when participants are unrestrained 

*Diederick C. Niehorster, Tim H. W. Cornelissen, Kenneth Holmqvist, Ignace T.C. Hooge & Roy S. Hessels *

II-81 Gaussian Mixture Models for Information Integration: Toward Gaze-Informed Information Foraging Models for Imagery Analysis

*Maximillian Chen, Kristin Divis, Laura McNamara, J. Daniel Morrow & David Perkins *

II-82 Moving low level eye movement data to meaningful content in dynamic environments 

*Kristin M. Divis, Maximillian Chen, Laura McNamara, J. Dan Morrow & David Perkins *

II-83 Measuring dynamic and static vergence using an autostereoscopic display 

*Wolfgang Jaschinski *

II-84 Objective measurement of variability of fixation disparity – is it possible? 

*Dawid Dominiak, Alicja Brenk-Krakowska & Wolfgang Jaschinski *

II-85 Sturmian-Wavelets as a to analyze eye tracking data 

*Jessica A. Del Punta, Gustavo Gasaneo, María L. Freije, Marcos Meo & Lorenzo U. Ancarani *

II-86 Study on eye movement dynamics during the ‘jumping point’ experiment 

*Katarzyna Harezlak & Pawel Kasprowski *

II-87 An Update to the EYE-EEG Toolbox for Combined Eye-Tracking and EEG 

*Olaf Dimigen *

II-88 Accuracy and precision test for a remote visible spectrum eye tracker 

*Chia-Ning , Ming-Da Wu, Yen-Hua Chang, Wen-Chung Kao, Yi-Chin Chiu & YaoTing Sung *

II-89 Study on Directional Eye Movements in Non-frontal Face Images for Eye-controlled Interaction 

*Min Lin *

II-90 Eye-movement in the dark for the exploration of virtual scenes encoded by sounds *Sylvain Huet, Julien Doré, Zélie Buquet, Denis Pellerin & Christian Graff*

II-91 OT Eye: A tool to guide intervention and monitor progress during occupational therapy 

*Pieter Blignaut, Elize Janse van Rensburg & Marsha Oberholzer *

II-92 GazeCode: an -source toolbox for mobile eye-tracking data analysis *Jeroen S. Benjamins, Roy S. Hessels & Ignace T.C. Hooge *

Session III - Wednesday, August 23^rd^, 15.30 - 17.00 

**Visual perception and ocolomotor control **

III-1 Exploring the temporal dynamics of trans-saccadic perceptual re-calibration * Matteo Valsecchi, Carlos R. Cassanello, Arvid Herwig, Martin Rolfs & Karl R. Gegenfurtner *

III-2 Selective facilitation of the luminance visual pathway by postsaccadic target blanking *Kazumichi Matsumiya, Masayuki Sato & Satoshi Shioiri *

III-3 Transsaccadic prediction of real-world objects

 Arvid 

III-4 Visual perception of intrasaccadic information: A response priming experiment * Charlotte Schwedes, Elodie Banse, Lorena Hell & Dirk Wentura *

III-5 Visual working memory aids trans-saccadic integration

 Emma E. Marshall Stewart & Alexander C Schütz

III-6 ERP potentials at the stage of saccadic preparation

 Victoria Moiseeva, Maria Slavutskaya, Natalia Fonsova & Valery Shulgovskiy 

III-7 The of distractor processing on the target-related P300: Evidence from fixation-related potentials

 Hannah Hiebel, Joe Miller, Clemens Brunner, Andrey R. Nikolaev, Margit Höfler, Anja Ischebeck & Christof Körner

III-8 Asymmetrical effects of saccade training on express saccade proportion in the nasal and temporal hemifields 

 Arni Kristjansson, Jay Edelman, Bjarki D. Sigurþorsson & Ómar I. Johannesson 

III-9 Saccade training increases peak velocities and express saccade proportion for both trained and untrained eyes 

 Ómar I. Johannesson, Jay A. Edelman, Bjarki D. Sigurþórsson & Árni Krisjánsson 

III-10 Age-related changes in modulation of saccadic control by salience and value * Jing Huang, Karl R. Gegenfurtner, Alexander C. Schütz & Jutta Billino *

III-11 An age-dependent saccadic saliency model 

 Antoine Coutrot & Olivier Le Meur 

III-12 Can the cortical magnification factor account for the latency increase in the remote distractor effect when the distractor is less eccentric than the target? 

 Soazig Casteau, Françoise Vitu & Robin Walker

III-13 The optokinetic nystagmus dynamic reflects the vection illusion perception 

 Artem Kovalev 

III-14 The of eye tracking in fMRI study: differences in adults and children predictive saccades 

 Katerina Lukasova & Edson Amaro 

III-15 Microsaccade and blink rates index subjective states during audiobook listening 

 Elke B. Lange & Moniek Kuijpers 

III-16 Fixation duration in EOG studies with eyes closed *Tanina Rolf & Niels Galley *

III-17 Separate resource pools for effector systems? Evidence from manual-oculomotor dual tasks 

 Aleks Pieczykolan & Lynn Huestegge 

III-18 Influence of background illumination on horizontal and vertical objective fixation disparity 

 Remo Poffa, Joëlle Joss & Roland Joos 

**Interactive and group eye tracking **

III-19 Explore the effectiveness of online dynamic video-text vs. static image-text multimedia learning on students' science performance: An Eye movement study 

 Ya-Chi Lin & Hsiao-Ching She 

III-20 Using -tracking to provide dynamic assistance on the reading skills of beginner readers on desktop or mobile devices

 Rykie Van der Westhuizen & Pieter Blignaut

III-21 Using Eye Tracking Data to Assist Identifying Wayfinding Strategies in the Virtual Maze 

 Tsuei-Ju Hsieh & Jun-Kai Niu 

III-22 Real-time visualisation of student attention in a computer laboratory 

 Pieter Blignaut 

III-23 Detecting collaboration in a real classroom mathematics problem solving session from visual scan-paths 

 Enrique G. -Esteva, Jessica Salminen-Saari, Miika Toivanen & Markku S. Hannula 

III-24 Preservice teachers’ professional vision of own classroom management: combining mobile eye tracking in the classroom with retrospective reporting 

 Sharisse van Driel, Halskza Jarodzka, Frank Crasborn & Saskia Brand-Gruwel 

III-25 “Look who's reading now!” - Evaluating the benefit of interactive eye tracking in chat * Christian Schlösser, Carsten Friedrich, Linda Cedli & Andrea Kienle *

III-26 Using eye-tracking techniques to explore students’ reading behaviors when using ebooks with different role-playing mechanisms

 Gloria Yi-Ming Kao, Xin-Zhi Chiang & Tom Foulsham

III-27 What simultaneous eye tracking of two people tell us about the social interaction and social presence of others? – A recurrence analysis 

 Seiya Kamiya, Haruka Nakamura & Takako Yoshida

III-28 Teacher monitoring pair and group work in English as a foreign language lessons: insights from an eye-tracking study 

 Eva Minarikova, Zuzana Smidekova & Miroslav Janik

III-29 Detecting collaboration in a real classroom mathematics problem solving session from visual scan-paths 

 Enrique G. Moreno-Esteva, Jessica Salminen-Saari, Miika Toivanen & Markku S. Hannula 

III-30 Facing in groups – An exploratory eye tracking and EDA study 

 Michelle L. Nugteren, Eetu Haataja, Halszka Jarodzka, Jonna Malmberg & Sanna 

Järvelä 

III-31 Infrastructure and Methodology for Group Studies in Multiple Eye Trackers Laboratory 

 Martin Konopka, Robert Moro, Peter Demcak, Patrik Hlavac, Jozef Tvarozek, Jakub Simko, Eduard Kuric, Pavol Navrat & Maria Bielikova 

III-32 Robust Recording of Program Comprehension Studies with Eye Tracking for Repeatable Analysis and Replay 

 Jozef Tvarozek, Martin Konopka, Jakub Hucko, Pavol Navrat & Maria Bielikova 

**Visual in the real world **

III-33 Analyze the gaze behavior of drivers of semi-autonomous vehicles 

 Holger Schmidt & Rahel Milla 

III-34 Adding mirror information to the traditional Hazard perception test discriminates between novice and experienced drivers

 Petya Ventsislavova & David Crundal 

III-35 Age-related changes in gaze dynamics during real-world navigation 

 Marcia Bécu, Guillaume Tatur, Annis-Rayan Bourefis, Luca L. Bologna, Denis Sheynikhovich & Angelo Arleo 

III-36 Potentials of eye-tracker use for wind turbine maintenance workers 

 Berna Ulutas & Stefan Bracke 

III-37 How individual differences in visual learning process are reflected by eye movements 

 Aleksandra Kroll & Monika Mak 

III-38 The challenge of learning histology: a longitudinal observational study with medical freshman students 

 Alan Brecht, Gertrud Klauer & Frank Nürnberger

III-39 The making on radiologists: A joint effect of experience and authority 

 Xuejun Bai & Meixiang Chen 

III-40 An Eye Gaze-Based Approach for Labeling Regions in Fundus Retinal Images 

 Nilima Kulkarni & Joseph Amudha 

III-41 No link between eye movements and reported eating behaviour in a non-clinical population 

 Frouke Hermens & Leanne Caie 

III-42 Using Eye Tracking to Evaluate Survey Questions

 Cornelia E. 

III-43 Identifying problems in translation from scratch and post-editing with keylogging and eyetracking data *Jean Nitzke*

III-44 Evaluating the Comprehensibility of Graphical Business Process Models – An Eye Tracking Study 

 Michael Zimoch, Rüdiger Pryss, Thomas Probst, Winfried Schlee, Georg Layher, Heiko Neumann & Manfred Reichert

III-45 Eye movements while perceiving images of natural and built environments * Jan Petružálek, Denis Šefara, Marek Franěk & Jiří Cabal *

III-46 Eye movements are linked to sexual preference in a real world preferential looking paradigm 

 Frouke Hermens & Oliver Baldry 

**Language and Cognition **

III-47 Gaze-speech coordination when listening to L1 and L2 speech 

 Agnieszka Konopka, Emily Lawrence & Sara Spotorno

III-48 When tones constrain segmental activation-competition in Chinese spoken word recognition: evidence from eye movements 

 Chung-I Erica Su, Guan-Huei Li & Jie-Li Tsai

III-49 Reading Music. How Tonality and Notation Influence Music Reading Experts' Eye Movements and Information Processing. 

 Lucas , Benedict Fehringer & Stefan Münzer

III-50 Characteristics of sight-reading performance of pianists depending on texture of musical pieces 

 Leonid V. Tereshchenko, Lyubov’ A. Boyko, Dar’ya K. Ivanchenko, Galina V. Zadneprovskaya & Alexander V. Latanov

III-51 Eye-movements during the encoding of object locations provide new insights into the processing and integration of spatial information 

 Anne-Kathrin Bestgen, Dennis Edler, Frank Dickmann & Lars Kuchinke 

III-52 Automatic identification of cognitive processes in the context of spatial thinking 

 Anna Klingauf & Benedict C.O.F. Fehringer

III-53 Rotate It! – What eye movements reveal about solution strategies of spatial problems 

 Stefanie Wetzel, Veronika Krauß & Sven Bertel

III-54 Fixation time as a predictor of the improvement of the test performance during a chronometric mental-rotation test 

 Martina Rahe & Claudia Quaiser-Pohl 

III-55 Eye movements during abductive reasoning process

 Li-Yu Huang & Hsiao-Ching She 

III-56 A to visualize the complete problem solving scenario 

 John J. H. Lin & Sunny S. J. Lin 

III-57 The effects of symbolic and social cues on gaze behavior

 Flora Ioannidou, Frouke Hermens & Timothy Hodgson

III-58 Tonic and Phasic Changes in Pupil Size Are Associated with Different Aspects of Cognitive Control 

 Péter Pajkossy, Ágnes Szőllősi, Gyula Demeter & Mihály Racsmány 

III-59 Pupil dilation and conflict processing: probability of occurrence of conflict trials influences pupil size 

 Michael A. , Franca Schwesinger & Jochen Müsseler 

III-60 Location Trumps Color: Determinants Of Free-Choice Eye Movement Control Towards Arbitrary Targets 

 Lynn Huestegge, Oliver Herbort, Nora Gosch, Wilfried Kunde & Aleks Pieczykolan 

III-61 A cross-cultural investigation of the Positive Effect in Older and Younger Adults: An Eye movement study 

 Jingxin Wang, Fang Xie, Liyuan He, Katie L Meadmore & Valerie Benson 

III-62 Time-dependency of the SNARC effect on number words: Evidence from saccadic responses 

 Alexandra Pressigout, Agnès Charvillat, Karima Mersad, Alexandra Fayel & Karine Doré-Mazars 

III-63 Empirical and Perceived Task Difficulty Predict Eye Movements during the Reading of Mathematical Word Problems 

 Anselm R. Strohmaier, Matthias C. Lehner, Jana T. Beitlich & Kristina M. Reiss 

III-64 Cognitive strategies for solving graphically presented chemical tasks 

 Yulia Ishmuratova & Irina Blinnikova 

**Reading: word-level processing **

III-65 The availability of low spatial frequency information affects the effect of word predictability 

 Stefan & Tim Jordan 

III-66 Cross-Frequency Coupling: Correlates of Predictability in Natural Reading 

 Nicole A. Himmelstoß, Sarah Schuster, Lorenzo Vignali, Stefan Hawelka, Florian 

Hutzler & Rosalyn Moran 

III-67 Predictability effects and preview processing for one- and two- character word in Chinese reading 

 Lei Cui, Jue Wang, Huizhong Zhao & Simon Liversedge

III-68 Reading words in context: Effects of predictability in children’s and adults’ eye movements 

 Simon -Richards & Sascha Schroeder

III-69 The two sides of prediction error in reading: on the relationship between eye movements and the N400 in sentence processing

 Franziska Kretzschmar & Phillip M. Alday

III-70 Understanding word predictability using Natural Language Processing algorithms * Bruno Bianchi, Gastón B. Monzón, Diego F. Slezak, Luciana Ferrer, Juan E. Kamienkowski & Diego E. Shalóm*

III-71 Working memory capacity affects eye movement behavior during Chinese reading 

 Xingshan Li &Ya Lou 

III-72 Reading searching in Chinese: The role of lexical processing 

 Sarah J. White, Xiaotong Wang, Li Hua Zhang & Xue Sui

III-73 Orthographic and Root Frequency Effects in Arabic: Evidence from Eye Movements and Lexical Decision 

 Ehab W. Hermena, Simon P. Liversedge, Sana Bouamama & Denis Drieghe 

III-74 Information Acquisition from Left of the Current Fixation: Evidence from Chinese Reading 

 Lin Li, Xue Sui & Ralph Radach 

III-75 Interword spacing effect on Chinese developmental dyslexia: A comparison in oral and silent sentence reading 

 Mingzhe , Ke Tan, Wen Wang & Xuejun Bai

III-76 Transposed Letter Effects in Persian: Evidence from a Semantic Categorization Task 

 Ehab W. Hermena, Hajar Aman-Key-Yekani, Ascensión Pagán, Mercedes Sheen & Timothy R. Jordani 

III-77 Word skipping in Chinese reading: The role of high-frequency preview and syntactic 

felicity 

 Chuanli Zang, Hong Du & Simon P. Liversedge

III-78 Semantic Transparency Modulates the Emotional Words in Chinese Reading: Evidence from Eye Movements 

 Kuo , Jingxin Wang, Lin Li, Shasha Pan & Simon Liversedge 

III-79 General Linear Model to isolate higher-level cognitive components from oculomotor factors in natural reading by using EEG and eye-tracking data coregistration 

 Anne Guérin Dugué, Benoît Lemaire & Aline Frey

III-80 The use of pupillary response as an indicator of reading task complexity in Irish * Patrick M. Hynes, Ronan G. Reilly & Raúl Cabestrero *

III-81 Dynamic properties of return sweep saccades during reading 

 Rostislav Belyaev, Vladimir Kolesov, Galina Menshikova, Alexander Popov & Victor Ryabenkov 

III-82 Taking to experimental testing: On the influence of serifs, fonts and justification on eye movements in text reading 

 Julian Jarosch, Matthias Schlesewsky, Stephan Füssel & Franziska Kretzschmar 

III-83 Translation quality assessment: eye movement evidence

 Alena Konina & Tatiana Chernigovskaya 

III-84 What does the rhino do with the rose? Predicting gaze duration to validate an adult version of the Salzburger Lese-Screening (SLS-B) 

*Jana Lüdtke, Eva Fröhlich & Arthur M. Jacobs*

## Abstracts

**19**^th^** European Conference on Eye Movements **

## Keynotes

**nday, August 20**^th^**, 18.00 - 19.00**

**Auditorium Maximum **(HS 33 - K.11.24) 

On covert attention and presaccadic attention

**Marisa Carrasco **

New University, United States of America marisa.carrasco@nyu.edu 

Endogenous (voluntary) and exogenous (involuntary) covert spatial attention alter performance and appearance in many basic visual tasks mediated by contrast sensitivity and spatial resolution, without accompanying eye movements. Presaccadic attention allocated to the location of the saccade’s target (in the absence of attention cues) also modulates performance. For instance, akin to covert attention, while planning an eye movement presaccadic attention improves performance and increases perceived contrast at the saccade target location. Critically, these modulations change the processing of feature information. Using a psychophysical reverse correlation approach, we found that saccade preparation selectively narrows orientation tuning and enhances the gain of high spatial frequency information at the upcoming saccade location. Moreover, this frequency shift takes place automatically even when it is detrimental to the task at hand. These three studies reveal that these modulations are timelocked to saccade onset, peaking right before the eyes move. Crucially, merely deploying covert spatial attention without preparing a saccade alters neither performance nor appearance within the same temporal interval. We propose that saccade preparation may support transaccadic integration by reshaping the representation of the saccade target to be more fovea-like just before saccade onset. I will discuss similarities and differences among covert–endogenous and exogenous–spatial, feature-based and presaccadic attention, with regard to their temporal dynamics, gain and tuning properties. 

**Monday, August 21**^st^**, 9.00 - 10.00**

**Auditorium Maximum **(HS 33 - K.11.24)

An update of the functional role of the dorsal visual stream

**Laure Pisella **

Lyon Research Center, France laure.pisella@inserm.fr 

While the deficits consecutive to the right temporo-parietal junction damage concern the spatiotemporal integration of the visual information sampled by eye movements, the deficits consecutive to the dorsal posterior parietal cortex (PPC) damage have in common to concern peripheral vision. In particular, bilateral dorsal PPC damage leads to a reduced search window when spatial binding of lines is needed, a specific visual search deficit for symbols which has also been observed in individuals with a visuo-attentional form of developmental dyslexia. One hypothesis we have put forward is that the dorsal PPC actively compensates for the under-representation of peripheral vision that accompanies central magnification. 

**Monday, August 21**^st^**, 17.00 - 18.00**

**Auditorium Maximum **(HS 33 - K.11.24) 

Where (and ) next? How people view images of natural scenes

**Ben Tatler **

University of Aberdeen, Scotland

b.w.tatler@abdn.ac.uk 

We move our eyes two or three times every second, in order to get the right information at the right time for the behaviours we engage in. Where we look is determined by a mixture of low-level information, higher-level understanding, internal goals, and biases to view scenes in particular ways. Computational models are emerging that include factors from the full range of this mixture and do a reasonable job at explaining where people look in scenes. However, a complete understanding of how we gather information from scenes must encompass not only where people look but also when they move their eyes. Studies of temporal allocation of gaze in scene viewing are far fewer in number than studies of spatial allocation and theories assume separate mechanisms for controlling the spatial and temporal allocation of gaze. 

I will review current understanding of spatial and temporal gaze allocation in scene viewing and present our recent decision-based model of scene viewing that encompasses both when and where the eyes move. Our model asserts that each decision to move the eyes is an evaluation of the relative benefit expected from moving the eyes to a new location compared with that expected by continuing to fixate the current target. The eyes move when the evidence that favors moving to a new location sufficiently outweighs that favoring staying at the present location. This single decision process can explain both when and where people look in scenes.

. 

**Tuesday, 22**^nd^**, 9.00 - 10.00**

**Auditorium Maximum **(HS 33 - K.11.24)

Eye movement studies of reading in special populations

**Debra Titone **

McGill University, Montreal debra.titone@mcgill.ca 

Eye movement investigations have long been crucial for building a comprehensive understanding of the cognitive and perceptual processes that support reading and other language processes because of their naturalness and great temporal precision (reviewed in Rayner, Pollatsek, Ashby & Clifton, 2012). Indeed, most of what we know about psycholinguistics has been deeply informed by eye movement reading data, including the fundamentals of word processing, contextual effects, grammatical interpretation, and higher-level aspects of language such as figurative or emotional effects on language. 

Of relevance here, much of this work has historically focused on university-aged monolingual (or presumed monolingual) readers. However, in recent years, eye movement studies of reading have been extended to a variety of “special” populations, many of which are actually quite common. In this talk, I present some of the work from my laboratory that has used eye movement measures to study a variety of psycholinguistic questions about reading in different populations. These populations include healthy bilingual younger and older adults, as well as neuropathic populations, such as people living with schizophrenia or dyslexia. Across these populations, I will focus on the interplay between local wordlevel processing and more global influences of context, such as what arises from variations in sentential constraint or the interpretive demands of figurative language. 

**Wednesday, August 23**^rd^**, 9.00 - 10.00**

**Auditorium Maximum **(HS 33 - K.11.24) 

The roles of cortical areas in guiding eye movements during visual search

**James Bisley **

University of California, United States of America jbisley@mednet.ucla.edu 

Over the past 25 years, it has become clear that the lateral intraparietal area (LIP) and the frontal eye field (FEF) play important roles in guiding saccades. Yet despite this focus, it is still unclear how the two areas differ: neurons in each area often behave quite similarly in standard search or oculomotor tasks. Using a visual foraging task, in which animals are free to move their eyes as they please and from which we can record activity across multiple eye movements within a trial, we have identified numerous differences between the areas. I will discuss the implications of these results and suggest that the areas are part of a recurrent system in which additional processing occurs between LIP and some FEF neurons, while others provide feedback to LIP about upcoming and previous saccades. 

**Thursday, August 24**^th^**, 17.30 - 18.30**

**Auditorium Maximum **(HS 33 - K.11.24)

The between vision and eye Movements

**Karl R. Gegenfurtner**

Giessen University, Germany gegenfurtner@uni-giessen.de 

The existence of a central fovea, the small retinal region with high analytical performance, is arguably the most prominent design feature of the primate visual system. This centralization comes along with the corresponding capability to move the eyes to reposition the fovea continuously. Past research on visual perception was mainly concerned with foveal vision while the observers kept their eyes stationary. Research on the role of eye movements in visual perception emphasized their negative aspects, for example, the active suppression of vision before and during the execution of saccades. But is the only benefit of our precise eye movement system to provide high acuity of the small foveal region, at the cost of retinal blur during their execution? In this review, I will compare human visual perception with and without saccadic and smooth pursuit eye movements to emphasize different aspects and functions of eye movements. I will show that the interaction between eye movements and visual perception is optimized for the active sampling of information across the visual field and for the calibration of different parts of the visual field. The movements of our eyes and visual information uptake are intricately intertwined. The two processes interact to enable an optimal perception of the world, one that we cannot fully grasp by doing experiments where observers are fixating a small spot on a display.

## Talks

Monday, August 21^st^, 10.30 - 12.30 

Symposium: Developmental eye tracking: Problems, solutions and applications of screen and head-mounted eye tracking

**Room 1 **(HS 14 - M.10.12) 

Assessing gaze data quality in a large multi-centre autism developmental cohort

**Ana M. Portugal, Luke Mason, Tim J. Smith **

Center for Brain and Cognitive Development, Birkbeck, University of London, UK, United Kingdom avazpo01@mail.bbk.ac.uk 

Eye tracking data quality is a matter of increasing importance when automatically processing data. This is especially critical when studying gaze behaviour developmentally (infants and children) or clinical populations, who commonly have problems with data recording and for whom the data tends to be systematically poorer. In this project we aimed at processing and analyzing eye tracking data collected as part of a large scale cohort (N=672) of children and adults with Autism Spectrum Disorder (LEAP EUAIMS). Given the size of the dataset, the multi-center nature of the study and the target population, it was expected that the quality of the dataset would differ across participants creating variable processing requirements. We estimated various quality related metrics in the raw gaze data, use principle component analysis to reduce these metrics to 4 quality dimensions (Flicker, Precision, Accuracy and Binocular Disparity); which were then used to cluster the datasets in terms of their relative level of quality. Finally, we automatically estimated fixations using different processing thresholds according to each quality group. This data-driven approach to classify data quality provides a potential way to achieve reliable and valid results that take in consideration the systematic differences found across developmental and clinical groups.

Gazepath: An eye-tracking analysis tool that accounts for individual differences and data quality

**Daan Renswoude**^1^**, Maartje Raijmakers**^2^**, Ingmar Visser**^1^

^1^University of Amsterdam, Netherlands; 

^2^Leiden University, Netherlands 

D.R.vanRenswoude@uva.nl 

Eye-trackers are a popular tool to study cognitive, emotional and attentional processes in different populations (e.g., clinical and typically developing) and participants of all ages, ranging from infants to elderly. This broad range of processes and populations implies there are many inter- and intraindividual differences that need to be taken into account when analyzing eye-tracking data. Standard parsing algorithms supplied by the eye-tracker manufacturers are typically optimized for adults and do not account for these individual differences. In this talk we presents gazepath, an easy-to-use R-package that comes with a graphical user interface (GUI) implemented in Shiny (RStudio, Inc, 2015). The gazepath R-package combines solutions from the adult and infant literature to provide a data-driven eye-tracking parsing method that accounts for individual differences and differences in data quality. Although gazepath is a suitable tool for both adult and infant data, in this talk we highlight its usefulness on infant data. We show that gazepath is able to pick up a developmental pattern of decreasing fixation durations with age, an effect that is obscured when standard parsing algorithms are used.

Quantifying the microdynamics of attention during parent-child interaction: practicalities and insights 

**Nadia Neesgaard, Atsushi Senju, Tim J. Smith**

Birkbeck, University of London, United Kingdom nneesg01@mail.bbk.ac.uk 

Development of joint attention is a fundamental skill for social and cognitive development from early infancy and has been the focus of much research. Evidence, however, shows that different ways of measuring this may not correlate with one another (Navab et al., 2011). Thus, more naturalistic yet still precise measures, such as head-mounted eye-tracking (HMET) during naturalistic parent-child interaction (PCI), are needed to determine how this mechanism develops. While pioneering researchers have successfully used HMET in PCIs (Yu and Smith, 2013), other researchers experience many problems due to the physiological differences and practical challenges of infant HMET. In this talk we will present data from a PCI-study using tabletop free-play (14-monthold infants) where we observed a high attrition rate and experienced great trouble with automated pupil-tracking, accurately tracking pupil center and corneal reflection in less than 50% of frames. Manual override of the tracking was used to salvage data and then hand-label region of interest hits (e.g. head, objects, hands) to measure the microdynamics of joint attention. However, such manual solutions incur a massive time cost (1 minute of data=180 minutes of hand-cleaning). We will discuss the influence data cleaning has on naturalistic measures of joint attention and propose solutions.

Head-mounted eye-tracking for studying infants’ attention during naturalistic activities 

**Heather L. Kirkorian, Seung H. Yoo**

University of Wisconsin-Madison, United States of America kirkorian@wisc.edu 

Eye-tracking is an increasingly popular method for studying perceptual, cognitive, and social development. Such methods are particularly valuable in studying infants and young children who cannot clearly articulate their thoughts or complete repetitive, complex experiments. However, most research with infants relies on simple visual patterns or still pictures of objects and faces, precluding generalizability to infants’ everyday experiences. In the real world, infants encounter complex scenes with people and objects who move through space and with whom infants can interact. Recent advances in head-mounted eye-tracking enables researchers to observe infants’ attention shifts in naturalistic settings, thus overcoming some limitations of prior studies. Nonetheless, this technology also presents new challenges, such as convincing infants to wear (but not touch) the apparatus on their heads. A related issue is the extent to which such compliance varies systematically with other measures (e.g., performance on inhibitory control tasks). This presentation will address some pros and cons associated with remote versus head-mounted eye-tracking to study infants’ visual attention during naturalistic activities (watching videos, interacting with mobile devices, playing with real objects). Emphasis will be given to the practicalities of collecting usable data from infants as well as analyzing data to answer key research questions. ****

Infants’ naturalistic attention dynamics show similar patterns at different spatio-temporal scales 

**Samuel V. **^1^**, Kaili Clackson**^2^**, Stani Georgieva**^2^**, Victoria Leong**^3^

^1^University of East London, United Kingdom; 

^2^University of Cambridge, United Kingdom; ^3^Nanyang Technological University, Singapore 

s.v.wass@uel.ac.uk 

The majority of our understanding of attention comes from studying attention within controlled experimental settings – which, due to their discrete, trial-by-trial nature often bear little resemblance to the requirements placed on attention in ‘real-world’ contexts. In typically developing 12-month-old infants we measured naturalistic attention in four settings. First, to a toy on a tabletop task, in two conditions – solo looking (child playing alone) and joint attention (child playing with mother). Second, to a screen, in two conditions – static (pictures) and dynamic (TV clips). The durations of spontaneous attention episodes were measured at two spatio-temporal scales - look durations and (for the screen stimuli only) fixation durations. For both look durations and fixation durations a similar pattern was observed: dynamic stimuli evoked more very short looks and fixations, but also more very long looks and fixations. A similar pattern was observed, for look durations, during joint attention relative to solo looking: joint attention led to more very short looks, but also more very long looks. We hypothesise that naturalistic attentional behaviours are influenced by two factors: attentional inertia and exogenous attention capture. These two factors combine to create the pattern of results observed. 

Exogenous attention capture is higher for dynamic stimuli, and during joint attention.

**Active Vision: What head-mounted eye tracking reveals about infants' active visual exploration**

**Chen Yu**

Indiana University, United States of America chenyu@indiana.edu 

Visual information plays a critical role in early learning and development, as infants accumulate knowledge by exploring the visual environment. Beyond the earliest stages of infancy, young children are not mere passive lookers, but they are also active doers. One of the first and most vitally informative types of actions infants take involves the self-control of their looking behaviors to visually explore the world. So-called active vision in infancy is key to the goal-directed selection of information to process. Recently, we use a new technology based on head-mounted eye tracking which allows us to collect vast volumes of egocentric video data and also to record infants’ moment-by-moment visual attention when they engage in various tasks in the real world. In this talk, I will present several studies, focusing on examining the structure of children’s dynamic visual experiences during active participation in a physical and social world. I will show how visual information is critical to serve a wide range of tasks in natural environments, from guiding motor action, to learning about visual objects, and to interacting with social partners.

Thematic Session: Saccade programming I

**Room 2** (HS 32 - K.11.23)

**Fixation-related brain activations: emotional valence interacts with high and low-level image properties**

**Michał Kuniecki**^1^**, Joanna Pilarczyk**^1^**, Kinga Wołoszyn**^1^**, Aleksandra Domagalik**^2^

^1^Psychophysiology , Institute of Psychology, Jagiellonian University, Kraków, Poland; 

^2^Neurobiology Department, The Małopolska Centre of Biotechnology, Jagiellonian University, Kraków, Poland 

michal.kuniecki@uj.edu.pl

Temporal and spatial characteristics of fixations are affected by image properties, including high-level scene characteristics and low-level physical characteristics. The influence of these factors is modulated by emotional content of an image. Here, we aimed to establish whether brain correlates of fixations reflect these modulatory effects. We scanned participants and measured their eye movements, while presenting negative and neutral images in various image clarity conditions, with controlled objectbackground composition. The fMRI data were analyzed using novel fixation-based event-related (FIBER) method, which allows tracking brain activity linked to individual fixations. Fixating an emotional object was linked to greater deactivation in the lingual gyrus than fixating the background of an emotional image, while no difference was found for neutral images. Deactivation in the lingual gyrus might be linked to inhibition of saccade execution. This was supported by longer durations of fixations falling on the object than on the background in the negative condition. Furthermore, increasing image clarity was correlated with fixation-related activity within the lateral occipital complex. This correlation was significantly stronger for negative images. Overall, emotional value of an image changes the way low- and high-level scene properties affect characteristics of fixations as well as fixation-related brain activity.

**Learning sequences of eye movements: linking motor processing and cognition in the brain**

**Melanie R. Burke**^1^**, Claudia C. Gonzalez**^2^

^1^University of Leeds, United Kingdom; 

^2^Thomson Rivers University, Department of Psychology, kamloops, BC, Canada.

m.r.burke@leeds.ac.uk 

Many of our daily activities involve the reproduction of series of well-coordinated movements that become well-rehearsed and result in automatic behavior. At some point all of these sequences of movements need to be acquired by the brain, and learnt (generally via repetition) to produce the often seamless result. This study aimed to look at the network of brain areas involved in the acquisition of such sequences and establish if the network differed depending on complexity of the sequence to be acquired. We examined the eye movements and brain activation in 12 healthy individuals to either short (4 component) or long (8 component) sequences and found clear segregation of networks for the shorter versus longer sequences respectively. Shorter sequences activated more pre-frontal brain regions including the dorsolateral prefrontal cortex, whereas in contrast longer sequences revealed activation in more posterior premotor and motor cortices. This supports the current model of parallel processing for motor learning proposed by Hikosaka et al (1999; 2002). The model describes an initial visuo-spatial acquisition of the information on first presentation of the stimulus that can then shift to more implicit (motoric) storage systems depending on sequence complexity.

**Oculomotor dominance in dual tasking and the influence of stimulus-response modality mappings**

**Mareike A. Hoffmann, Aleks Pieczykolan, Lynn Huestegge**

Julius-Maximilians-Universität Würzburg, Germany mareike.hoffmann@uni-wuerzburg.de 

Performing two tasks simultaneously usually yields performance costs. Whenever tasks involve different response modalities (in terms of utilized effector systems), such dual-task costs are typically distributed asymmetrically, a phenomenon that can be interpreted as a marker for response modality prioritization. Specifically, the response modality exhibiting fewer dual-task costs is regarded as being prioritized over the other. Based on this rationale, previous studies examined cross-modal dualresponse compounds triggered by single (auditory) stimuli and demonstrated oculomotor (i.e. saccade) dominance over manual and vocal responses. In order to extend the range of response modalities and to generalize previous findings to a typical dual-task setting, the present study investigated dual-task cost asymmetries in pairwise combinations of saccadic, manual, vocal, and pedal responses triggered by visual or auditory stimuli. Furthermore, by manipulating the mapping of stimulus modality to response modality, which is known to affect dual-task performance, we were able to assess its specific role for response modality prioritization. The resulting dual-task cost asymmetries across all pairings suggest a consistent ordinal prioritization pattern among response modalities including clear oculomotor dominance over all other effector systems. Interestingly, while S-R modality mappings affected dual-task costs in certain response combinations they did not modulate the overall prioritization pattern.

**Using Averaging to study Decision Making signals**

**Geoffrey Megardon, Petroc Sumner**

Cardiff University, United Kingdom geoffrey.megardon@gmail.com 

The Global Effect (GE) traditionally refers to the tendency of eyes to first land in between two nearby stimuli – forming a unimodal distribution. By measuring a shift of this distribution, recent studies used the GE to assess the presence of decision-related inputs on the motor map for eye movements. However, this method cannot distinguish whether one stimulus is inhibited or the other is facilitated and could not detect situations where both stimuli are inhibited or facilitated.Here, we find that 1) a GE is detectable in the bimodal distribution of landing positions for remote stimuli, and 2) this bimodal GE reveals the presence, location and polarity (facilitation or inhibition) of selection-history and goalrelated signals. We tested, for different inter-stimulus distances, the effect of the rarity of doublestimulus trials, and the difference between performing a discrimination task compared to free choice. Our work show that the effect of rarity is symmetric and decreases with inter-stimulus distances, while the effect of goal-directed discrimination is asymmetric -- occurring only on the side of the distractor – and maintained across inter-stimulus distances. These results suggest that the former effect changes the response property of the motor map, while the latter specifically facilitates the target location.

**The necessity to choose causes effects of expected value**

**Christian Wolf, Anna Heuer, Anna Schubö, Alexander C. Schütz**

Philipps-Universität , Germany chr.wolf@uni-marburg.de 

Humans can maximize reward by choosing the option with the highest expected value (probability × magnitude of reward). Expected value is not only used to optimize decisions but also for movement preparation to minimize reaction times to rewarded targets. Here, we asked whether this is only true in contexts in which participants additionally have to choose between different options. We probed eye movement preparation by measuring saccade latencies to differently rewarded single targets (single-trial) appearing left or right from fixation. In choice-trials, both targets were displayed and participants were free to decide for one target to receive the corresponding reward. Single-trial latencies were modulated by expected value only when choice-trials were present. The influence of expected value increased with the proportion and difficulty of choices and decreased when a cue indicated that no choice will be necessary. Choices caused a delay in subsequent responses to the nonchosen option which can be explained by a lowered baseline activity in the decision signal. Taken together, our results suggest that expected value affects saccade preparation only when the outcome is uncertain and depends on the participants’ behavior, for instance when they have to choose between targets differing in expected value.

**SERIA – A model for antisaccades**

**Eduardo A. Aponte**^1^**, Dario Schoebi**^1^**, Klaas E. Stephan**^1,2^**, Jakob Heinzle**^1^

^1^Translational Neuromodeling Unit, University of Zurich/Swiss Institute of Technology, Zurich, Switzerland; 

^2^Wellcome Trust Center for Neuroimaging, University College London, London, UK aeduardo@biomed.ee.ethz.ch 

The task is a paradigm to study voluntary control of eye movements. Participants need to inhibit a prepotent reaction towards a cued location and to produce a saccade in the opposite direction. Here, we introduce the Stochastic Early Reaction, Inhibition, and late Action (SERIA) model, a novel statistical approach to model error rates and reaction times. In contrast to previous methods, we provide a formal statistical formulation as a generative model which can be fitted to individual trials, not only summary statistics. We applied the SERIA model to data (47 subjects) acquired during a mixed pro- and antisaccades design. The two types of responses were randomly interleaved with ratios of 80:20, 50:50 and 20:80, respectively. In total, 27072 trials were analysed and used to infer the parameters of the model. Our results indicate that the SERIA model can explain eye movement behaviour on a subject by subject basis. Moreover, different components of the model are affected by trial probability in different manners: Whereas inhibitory control was highly sensitive to trial type probability, a unit controlling initiation of voluntary action was affected by uncertainty, i.e., Shannon entropy. 

Special Thematic Session: Communication by gaze interaction I

**Room 3 **(HS 28 - I.13.71) 

**Eye movement as material for interaction design **

**Hans Gellersen **

Lancaster University, UK 

h.gellersen@lancaster.ac.uk

Eye movements are central to most of our interactions. We use our eyes to see and guide our actions and they are a natural interface that is reflective of our goals and interests. At the same time, our eyes afford fast and accurate control for directing our attention, selecting targets for interaction, and expressing intent. Even though our eyes play such a central part to interaction, we rarely think about the movement of our eyes and have limited awareness of the diverse ways in which we use our eyes for instance, to examine visual scenes, follow movement, guide our hands, communicate non-verbally, and establish shared attention.This talk will reflect on use of eye movement as input in human-computer interaction. Jacob's seminal work showed over 25 years ago that eye gaze is natural for pointing, albeit marred by problems of Midas Touch and limited accuracy. I will discuss new work on eye gaze as input that looks beyond conventional gaze pointing. This includes work on: gaze and touch, where we use gaze to naturally modulate manual input; gaze and motion, where we introduce a new form of gaze input based on the smooth pursuit movement our eyes perform when they follow a moving object; and gaze and games, where we explore social gaze in interaction with avatars.

**“Here´s looking at you, kid.” Does he see pupil size changes?**

**Anke Huckauf, Christoph Strauch, Jan Ehlers**

Ulm University, Germany

anke.huckauf@uni-ulm.de 

In -human interaction, looking into one’s eyes is an important characteristics. Assuming that pupil-based information is of importance in social interactions, its integration might also be conductive for smooth human-computer-interaction. We review respective hitherto existing approaches with the aim to identify promising per-spectives for future applications. Stimulus-driven pupil-based selection (e.g., Mathot et al., 2013) makes use of the fact that brightness changes produce large and reliable changes of the pupil size. Nevertheless, this methods is slowed down by the require-ment of a binary decision process. Another attempt utilizes actively controlled pupil size changes which can be achieved via biofeedback (e.g., Ehlers et al., 2015, 2016). This produces reliable signals without the need to use binary decisions. The results obtained with active pupil size changes suggest that selection times of about 1s are achievable. Using automatic pupil size changes for input (e.g., Strauch et al., 2017), the signal amplitude is smaller but, pupil size changes can be observed much faster. Although comparative evaluations of all approaches are still missing, the observations so far suggest that pupil-based input provides promising information for target selec-tion. 

**Gaze-contingent Games for Neurocognitive Therapy: More than Meets the Eye?**

**Leanne Chukoskie, Jacqueline Nguyen, Jeanne Townsend**

UC San Diego, United States of America lchukoskie@ucsd.edu 

Attention systems lie at the foundation of a broad range of cognitive skills. Weaknesses in the ability to shift attention can affect higher-level skills such as executive function and social processing. The shared neural circuitry underlying both gaze and attention shifting behavior presented us with an opportunity to train the slow attention shifting observed in individuals with autism spectrum disorders. We designed a suite of gaze-contingent video games incorporating training principles to scaffold basic attention skills. Following the 8 weeks of home-based training with these games, we observed significant change in spatial attention. Our use of gaze as an operant behavior also improved inhibitory control and the ability to maintain focus. Here, we examine additional potential uses of gaze-contingent games including as a supplement to traditional vision therapy. We also report our experience adapting an eye tracking system and our games for an individual with cerebral palsy, who lacks the motor skill to manually control a computer. In summary, this gaze-contingent neurogaming approach shows promise as an effective intervention in autism and has potential as a therapeutic for a broader range of disabilities.

**Applicability of smooth-pursuit based gaze interaction for elderly users**

**Sarah-Christin Freytag, Stefan Ruff, Antje C. Venjakob**

Technische Universität Berlin, Germany freytag.sc@gmail.com 

The applicability of smooth-pursuit based gaze interaction for older users was investigated. In an experiment with 75 older users (55-79 years) three different velocities of object movement were tested regarding two different interfaces: one for entering digits and one for entering letters. Participants were presented with an audio sequence of words or numbers respectively. The sequence was first read in whole and then with each word or number separately to allow for direct input after each chunk of information. The results showed that entering digits was reliable and relatively quick (v1= 1.2 % errors & 6.6 s/number, v2= 1.8% errors & 8.5 s/number, v3= 5.5% errors & 9.8 s/number), while entering sentences was slower and more prone to error (v1= 5.0% errors & 8.1 s/letter, v2= 6.6% errors & 26.9 s/letter, v3= 30.4% errors & 11.8 s/letter) The results suggest that older users are capable of using smooth pursuits for gaze interaction. The number of elements involved in the selection process should be limited to allow for robust selection. Furthermore, the participants stated that the technology was easy to learn and comfortable to use, thus indicating a general acceptance for this interaction technology.

**Behavioral Analysis of Smooth Pursuit Eye Movements for Interaction**

**Argenis Ramirez-Gomez, Hans Gellersen**

Lancaster University, United Kingdom

a.ramirezgomez@lancaster.ac.uk

Gaze has been found challenging to use in dynamic interfaces involving motion. Moving targets are hard to select with state of the art gaze input methods and gaze estimation requires calibration in order to be accurate when offering a successful experience. Smooth Pursuit eye movements broaden opportunities to extend novel interfaces and promise new ways of interaction. However, there is not enough information on the natural behavior of the eyes when performing them. In this work, we tried to understand the relationship between Smooth Pursuits and motion, focusing on movement speed and direction. Results show anticipatory movements when performing pursuits, indicating that the natural behavior of the eyes to predict the displayed movement. Results could help in the design of interfaces and algorithms that use Smooth Pursuit for interaction.

Thematic Session: Reading: Neural basis and binocular coordination

**Room 4** (HS 26 - I.13.65)

**Saccadic eye movements and neural activity associated with letter naming speed task manipulations**

**Noor Z. Al Dahhan, Donald C. Brien, John R. Kirby, Douglas P. Munoz** Queen's University, Canada noor.aldahhan@queensu.ca 

To further understand the processes that are involved during reading, we combined functional magnetic resonance imaging (fMRI) with eye tracking to investigate the neural substrates and cognitive processes underlying performance during letter naming speed (NS) tasks. 19 healthy young adults (ages 21 - 26 years) were recruited. We employed a block design consisting of a letter NS task and three variants that were phonologically and/or visually similar while participants’ eye movements and articulations were recorded. When the stimuli were both visually and phonologically similar, participants had significantly longer naming times and fixation durations, and made more frequent saccades and regressions than in the single manipulation conditions. fMRI results indicate significant activation in regions involved in the reading network and in tasks that require eye movement control and attention in typical adult readers. Activation in the left temporoparietal areas of the reading network increased as stimuli became more visually and phonologically similar to one another indicating differential neural processes that were associated with each task. These findings further our understanding of the neural substrates required for reading, and indicate that NS tasks recruit the same network of neural structures that are involved in reading and target key regions within this network. 

**The effects of cloze probability and semantic congruency on brain responses during natural reading: A fixation-related fMRI study**

**Sarah **^1^**, Nicole A. Himmelstoß**^1^**, Stefan Hawelka**^1^**, Fabio Richlan**^1^**, Martin Kronbichler**^1,2^**, Florian Hutzler**^1^

^1^Centre for Cognitive Neuroscience, University of Salzburg, Austria; 

^2^Neuroscience Institut, Christian-Doppler Klinik, Salzburg, Austria 

Sarah.Schuster@stud.sbg.ac.at

The predictability of a word based on prior sentence context facilitates visual word recognition. Most evidence from neuroimaging – especially from functional magnetic resonance imaging (fMRI) – however, stems from studies presenting semantically legal and illegal sentences which consistently highlight contributions of the left inferior frontal gyrus during semantic processing. Contributions of the left temporal cortex are less consistently reported which is at odds with the notion of this regions’ involvement in storing and retrieving lexico-semantic information. The present study investigated the effects of cloze probability and congruency on eye movements and brain responses during natural reading by means of simultaneous eye-tracking and fMRI. While manipulating the congruency of sentence final words, we also induced different levels of expectations (i.e., cloze probability). In so doing, we observed higher activation within left inferior frontal regions in response to semantic violations compared to legal continuations, whereas left middle temporal regions exhibited higher activation to high-cloze words compared to low-cloze words. Moreover, left occipito-temporal regions, which have been linked to visuo-orthographic processing, exhibited an effect of congruency for highcloze finals, indicating that prediction formation might not be limited to the lexico-semantic level, but also propagates to the orthographic level.

**Reading fluency is associated with fixation related brain responses to reading comprehension in 12-year old typically reading children – findings from coregistered eye-tracking and EEG study**

**Otto Loberg, Jarkko Hautala, Jarmo A. Hämäläinen, Paavo H. T. Leppänen** University of Jyväskylä, Finland loberg.o.h@gmail.com 

We ran an experiment where 67 sixth graders read single sentences that were either semantically plausible, had the last word replaced with a semantically incongruent word or had the last word replaced by a semantically incongruent word neighbor of a plausible last word. The experiment was conducted in Finnish, a highly transparent language, and participants were asked to judge all sentences on their sensibility. We found differentiating pattern of first fixation duration, re-fixation proportion, judgement accuracy, N400 and P600 effects between the congruent, incongruent and word neighbor target words. Also, we analyzed the relationship of reading fluency to eye-movements, fixation related brain potentials and judgement accuracy. Reading fluency was especially associated with the comprehension processes elicited by the word neighbor target words. We suggest that reading fluency modulates the time course of comprehension processes, but is not a strong determinant of comprehension accuracy when the task is simple semantic violation task.

**Changes in overall vergence demands affect binocular coordination during reading**

**Stephanie Jainta**

Fachhochschule Nordwestschweiz - Hochschule für Technik, Switzerland stephanie.jainta@fhnw.ch 

Binocular reading requires fine-tuned vergence eye movements to establish and maintain a single percept of the text. Prismatic glasses (used to relax vergence) induce “forced vergence” conditions, i.e. change absolute disparities while accommodative demands remain unchanged. At present, no data exists describing effects of forced vergence on binocular coordination in reading. We therefore measured binocular eye movements (Eyelink II) while 3 groups of 10 participants read 40 sentences. 20 sentences were always presented with a vergence demand of 6 degrees (individual control). Next, the absolute disparity of the other 20 sentences was either reduced (by -1 degree; EXO-group) or increased (by +1 degree; ESO-group) or remained the same (control-group). While the measured vergence angle decreased (5.3 degrees, EXO-group) or increased (7.2 degrees, ESO-group) relative to controls (6.2 degrees), fixation disparities (i.e. vergence errors relative to the actual demand) increased (to 25 min arc) only for reduced vergence demand (EXO-group). This shows an asymmetry in vergence adjustments and is further supported by a selective increase in convergent drifts (during fixations) for increased demands only (ESO-group). Implications for overall reading performance and optometric treatments are discussed, also accounting for unaltered heterophoria, number of fixations, fixation durations and saccade amplitudes.

**Binocular advantages for parafoveal processing in reading**

**Hazel I. Blythe**^1^**, Mirela Nikolova**^1^**, Stephanie Jainta**^2^**, Simon P. Liversedge**^1^

^1^University of Southampton, United Kingdom; 

^2^Institute of Optometry, University of Applied Sciences and Arts Northwestern, Switzerland hib@soton.ac.uk 

During reading, binocular visual input results in superior performance and is important in parafoveal pre-processing of text. It is not yet clear whether binocular vision in the parafovea primarily facilitates accurate saccadic targeting, efficient cognitive pre-processing of text, or both. We used a dichoptic, gaze-contingent, moving window paradigm (Nikolova et al., 2017) in order to establish: (1) the spatial extent of the region of parafoveal text for which binocular input is necessary for fluent reading; and (2) whether lexical processing efficiency, saccadic targeting accuracy, or both, would show the binocular advantage from parafoveal preview. Reading time measures revealed that cognitive processing of text was disrupted unless word N+1 was entirely binocular in the parafovea, but no additional benefit was observed when word N+2 was also binocular. Additionally, whilst reading times showed a clear binocular advantage to lexical processing from parafoveal preview, saccadic targeting parameters (e.g., accuracy, speed, amplitude and velocity) did not. We conclude that the disruption to reading caused by presenting monocular text to the right of fixation cannot be attributed to difficulties in binocular coordination in saccadic targeting but, instead, results from a decreased efficiency in the cognitive preprocessing of words in the parafovea prior to lexical identification.

**A understanding of vergence within fixations, based on differences in the reading of Chinese and English**

**Richard Shillcock**^1^, **Yi-ting Hsiao**^1^**, Mateo Obregón**^1^**, Hamutal Kreiner**^2^**, Matthew A. J. Roberts**^1^**, Scott McDonald**^3^

^1^University of Edinburgh, United Kingdom; 

^2^Ruppin Academic Centre, Emeque-Hefer, Israel.; 

^3^National Institute for Public Health and the Environment, Bilthoven, Netherlands rcs@inf.ed.ac.uk 

We report analyses from the Edinburgh 5-Language Corpus showing significant differences between the reading of English and Chinese. Chinese readers generate significantly wider (crossed) binocular fixation disparities (FDs) at the beginning of fixation; FDs at end of fixation are closely similar. We present a novel interpretation of the greater divergence in Chinese based on recent demonstrations that apparent size causes increases in visual sensitivity (Arnold & Schindel, 2010) and engages more cortical resource in V1 (Kersten & Murray, 2010). We argue that, when faced with visually complex orthography, the oculomotor system is using size-constancy scaling to enable the cortical visual system to ‘zoom in’ on the text. That is, divergence signals that a more distant object is being inspected, causing size constancy to increase the area of cortex activated, thereby facilitating the orientational processing required by Chinese character recognition. We add this Divergence-Induced Magnification Effect (DIME) to our existing theory of binocular crossed and uncrossed FDs (Shillcock, Roberts, Kreiner, & Obregón, 2010), in which we have argued that crossed FDs reflect unproblematic viewing conditions (or ‘Crossed eyes, No Trouble’, CENT). Together they yield the Divergence Affects Reading (DOLLAR) theory. We discuss the implications for existing theories of binocular fixations.

Monday, 21^st^ 13.30 - 15.30 

Symposium: Using eye-tracking and pupillometry to study rhythmic processing in music and dance

**Room 1 **(HS 14 - M.10.12) 

Eye Can’t Dance; Entraining Saccadic Timing to Musical and Visual Beats 

**Jonathan P. Batten, Tim J. Smith**

Birkbeck, University of London, United Kingdom jonobatten@gmail.com 

Music has been shown to entrain both voluntary and involuntary movements such as walking and heart beats. One of our most frequent movements, saccades are thought to be subject to an internal timer that may also be susceptible to entrainment. To investigate the influence of musical tempi on eye movements we developed a continuous visual search task that minimized extraneous influences on saccadic timing by having participants look clockwise around an ellipse of small circles, in search of a change in the circle’s letter or colour. Target presentation was either gaze contingent, tap-contingent, or externally timed. Across multiple studies we found: 1) explicit control of saccadic timing is limited to a small proportion of saccades and imprecisely synchronised when compared to finger-tap timing; 2) saccadic timing does not show any passive entrainment with musical beats, even when the music is closely aligned in phase; 3) but eye movement timing will synchronise to an isochronous visual sequence. This visual synchrony was not affected by the addition or absence of a corresponding musical beat. These results provide strong evidence that automatic eye movement timing is sensitive to the temporal demands of visual tasks and impervious to the entraining influence of musical beats.

**Pupil dilation indexes the metrical hierarchy of unattended rhythmic violations**

**Atser Damsma, Hedderik van Rijn**

University of Groningen, Netherlands

a.damsma@rug.nl 

When we listen to music, we perceive regularities that drive our expectations. This is reflected in beat perception, in which a listener infers a regular pulse from a rhythm. However, it is still an open question whether attention to the music is necessary to establish the perception of a hierarchy of stronger and weaker beats, or meter. In addition, to what extent beat perception is dependent on musical expertise is still unknown. We addressed these questions by measuring the pupillary response to omissions at different metrical positions in drum rhythms, while participants attended to another task. We found that the omission of the most salient first beat elicited a larger pupil dilation than the omission of the less salient second beat. These results show that participants perceived stronger and weaker beats without explicit attention to the music, suggesting that hierarchical beat perception is an automatic process that requires minimal attentional resources. In addition, we found that this perception of meter was independent of musical expertise. Finally, our results show that pupil dilation reflects surprise without explicit attention, demonstrating that the pupil is an accessible index to unattentive processing.

**Predicting attention to auditory rhythms using a linear oscillator model and pupillometry**

**Lauren K. **^1,2^**, Joy J. Geng**^1,2,3^**, Brian K. Hurley**^1,3^**, Petr Janata**^1,2,3^

^1^Center for Mind & Brain, University of California Davis, United States of America; 

^2^Neuroscience Graduate Group, University of California Davis, United States of America; ^3^Department of Psychology, University of California Davis, United States of America lkfink@ucdavis.edu 

Multiple studies have shown facilitation of auditory and visual responses when targets of either modality are presented simultaneously with a salient beat in musical time. Most of these studies assume their stimuli follow a hierarchical music-theoretic model of time (‘strong’ and ‘weak’ beats), though often music is found pleasurable by virtue of violations of this hierarchy. Here we assess the potential of a stimulus-driven linear oscillator model (Tomic & Janata, 2008) to predict dynamic attention to complex musical rhythms, beyond ‘strong’ and ‘weak’ beats. In addition to calculating participants’ perceptual thresholds for detecting deviants at time points of varying predicted salience, we measured pupil size as an index of attentional state. In our task, participants listened to continuously looping rhythmic patterns and responded anytime they heard a change in volume (200ms deviant; increments/decrements by block). An adaptive thresholding algorithm adjusted the intensity of each deviant at multiple temporal positions throughout each pattern. Interestingly, the pupil dilated to both increment and decrement deviants and was a reliable index for distinguishing detected vs. undetected deviants. A significant negative correlation was found between model-predicted temporal salience and perceptual threshold, highlighting our model’s ability to predict dynamic attention.

**The Eye-Time Span in Music Reading: Local Effects of Stimulus Complexity on **

“Looking Ahead” 

**Erkki Huovinen**^1^**, Anna-Kaisa **^2^**, Marjaana Puurtinen**^3^

^1^Department of Education, Royal College of Music in Stockholm, Sweden;

^2^Department of Music, Art and Culture Studies, University of Jyväskylä, Finland;

^3^Turku Institute for Advanced Studies & Department of Teacher Education, University of Turku, Finland 

erkki.s.huovinen@gmail.com

In reading music, the musician has to “look ahead” from the notes currently played in order to perform fluently. Known as the Eye-Hand Span, this phenomenon has been measured in ways that we divide into the forward projective approach (fixing the “back end” of the span in the musical performance and finding out how far the reader’s gaze extends at that point in time) and the single-item lag approach (pairing a fixation on a score element with the later performance of the same element). Neither of these approaches works for studying stimulus-driven effects on the span, and we thus introduce a backward projective approach, intended as a measure of symbol salience. Starting at a fixation targeting a given note symbol, we work backwards in the score, measuring the distance (in units of musical meter) to “where the musical time was going” at fixation onset. We call this measure the EyeTime Span (ETS). By two experiments of sight-reading musical melodies in tempo, we show that musicians’ visual processing involves local adjustments of the ETS due to music-structural complexity 

(and/or visual salience). Notably, however, the “more difficult” elements may also be handled by early glances to the notes preceding the difficult targets. 

**Guided eye movements made in response to dance**

**Matthew H. Woolhouse**

McMaster University, Canada woolhouse@mcmaster.ca 

Studies involving expert and novice viewers indicate that biological-motion schemas influence eye movements in the observation of dance; some gestures create relatively concentrated fixation clusters amongst participants, whereas others lead to diffuse patterns. These findings suggest that experienced dancers dictate where and how they are observed, guiding viewers’ attention. We investigated the influence of dance gestures on eye movements, and whether these influences are shared between observers. To investigate individual dancer and dancer gaze-direction effects, two females were videoed performing dance-gesture sequences under three gaze-direction conditions: looking (1) at camera, (2) off camera, and (3) at their own gestures. Simultaneously, the dancers’ biological movement data were recorded using an infrared passive-marker motion-captured system, enabling body kinematics and moving ROIs to be included in the analysis. Choreography included 3 anatomical groups (arms, legs, full-body), 2 action locations (peripheral, medial), 2 movement types (staccato, legato), resulting in 12 gestures. Gestures were arranged into 2 random orders prior to being performed and videoed. Videos were presented to 32 males/females, while an optical eye-tracking camera recorded eye movements. Results reveal the significant impact upon observers’ eye movements of dancer gaze-direction, limb type and location, and independent kinematic variables, including peak velocity, periodicity and acceleration. 

Thematic Session: Transsaccadic memory and integration

**Room 2** (HS 32 - K.11.23)

**Beyond the magic number four: Evidence for high-capacity, trans-saccadic, fragile memory and pre-attentive remapping**

**Paul Zerr**^1^**, Surya Gayet**^1^**, Kees Mulder**^2^**, Ilja Sligte**^3^**, Stefan Van der Stigchel**^1^

^1^Experimental Psychology, Helmholtz Institute, Utrecht University, Netherlands;

^2^Methodology and Statistics, Utrecht University, Netherlands; 

^3^Brain and Cognition, Department of Psychology, University of Amsterdam, Netherlands

p.zerr@uu.nl 

Visuospatial short term memory comes in at least two flavors: robust, capacity-limited working memory (WM) and high-capacity, pre-attentive, maskable sensory memory (e.g. fragile memory; FM). Saccades require eye-centered coordinates in memory to be updated (spatial remapping). This process has been considered strictly limited to WM. Can sensory memory also be remapped? We compared transsaccadic WM (tWM) and trans-saccadic FM (tFM) capacity in a change-detection experiment. A predictive retro-cue indicated future targets and protected FM from interference by the memory probe, enabling capacity estimates that include FM items. If only stable, attended memory items (WM) can be remapped, then trans-saccadic capacity should be equal to tWM, even if capacity was high before the saccade (FM). We observed a tFM capacity considerably above that of tWM using Bayesian analysis. This demonstrates that in addition to WM, non-attended sensory memory items were also remapped. Further, we observed that post-saccadic masks disrupted FM in spatiotopic locations, confirming that FM was remapped to world-centered coordinates. We demonstrate remapping of sensory memory and challenge the strongly held belief that trans-saccadic memory is identical to stable WM. This has important implications for the understanding of spatial remapping, which was considered to be intimately linked to spatial attention. 

**Unifying the visual world across an eye-movement: Transsaccadic integration is unaffected by saccade landing position**

**Martijn J. Schut, Nathan Van der Stoep, Stefan Van der Stigchel**

Experimental Psychology, Helmholtz Institute, Utrecht University, Utrecht, Netherlands

m.j.schut@uu.nl 

The subjective experience of our visual surroundings seems continuous, contradicting the erratic nature of visual processing due to saccades. One of the ways the visual system can construct a continuous experience is by integrating pre-saccadic and post-saccadic visual input. However, saccades rarely land exactly at the intended location. Transsaccadic integration would therefore need to be robust against variations in actual saccade execution to facilitate visual continuity. In the current study, we investigated the effect of saccade landing point on transsaccadic integration using a global effect paradigm. In this paradigm participants reported a feature (here color) of the saccade target, which changed slightly during the saccade in half of the trials. In these transsaccadic change-trials, all participants reported a mixture of the pre- and post-saccadic color, indicating transsaccadic integration. In global effect trials, a distractor appeared together with the saccade target, causing most saccades to land in between the saccade target and distractor. Strikingly, there was no effect of saccade landing point on the outcome of transsaccadic integration. Therefore, transsaccadic integration seems robust against variance in saccade landing point, providing further evidence for its role in facilitating visual continuity.

**How quickly does the eye movement system register changes across saccades?** **Jonathan van Leeuwen, Artem V. Belopolsky**

Vrije Universiteit Amsterdam, Netherlands

j.vanleeuwen@vu.nl 

Every time we make a saccade we form a prediction about what we are going to see when the eye lands. While the oculomotor system quickly adjusts to the changes in the visual world, even when occurring during saccades, these changes often go unnoticed. In the current study we investigated how quickly the oculomotor system updates predictions when a distractor is displaced during a saccade. We used saccade curvature to track target-distractor competition and how it is updated when a distractor changes location during a saccade. Participants performed sequences of horizontal and vertical saccades, oculomotor competition was induced by presenting a task-irrelevant distractor before the first saccade. On half of the trials the distractor remained in the same spatial location after the first saccade and on the other half the distractor moved to the opposite hemifield. At short intersaccadic intervals, second saccades curved away from the original distractor location. However, second saccades starting more than 180 ms after the first saccade curved away from the new distractor location. The results show that the oculomotor system initially assumes world stability, but is able to quickly update predictions based on new visual information.

**Trans-saccadic integration is contrast dependent**

**Lukasz Grzeczkowski, Heiner Deubel, Martin Szinte**

Allgemeine und Experimentelle Psychologie, Department Psychologie, Ludwig-Maximilians-Universität 

München, Germany lukasz.grzeczkowski@gmail.com 

Across saccades, the visual system receives two successive static images of the pre- and the postsaccadic retinal projections of the visual field. The existence of a mechanism integrating these images across saccades, and in particular the features they contain, is still nowadays a matter of debate. One way to study trans-saccadic integration is to use the blanking paradigm. Indeed, while a small transsaccadic object shift normally stays unnoticed, blanking the object after the saccade makes the same shift easily noticeable. Recently, it was shown that the blanking effect is reduced when the transsaccadic object is isoluminant relative to the background. Here, using the blanking paradigm, we study the transfer of a visual feature across saccades. Observers saccaded to a grating and discriminated an orientation change occurring during the movement. The post-saccadic grating was either presented with or without a 200 ms blank, and was either non-isoluminant or isoluminant. With non-isoluminant objects we observed an improvement of discrimination with a blank, a blanking effect for orientation. Interestingly, the blanking did not bring benefit to the discrimination of the isoluminant object. We propose that these effects reflect the existence of a trans-saccadic feature integration mechanism that is contrast dependent.

**Task-relevant objects compete for attention across saccades**

**Christian H. Poth, Werner X. Schneider**

Neuro-cognitive and Cluster of Excellence Cognitive Interaction Technology, Bielefeld University, Germany; 

c.poth@uni-bielefeld.de 

Object recognition is limited to a few objects at a time. For being recognized, objects compete for limited attentional processing resources. The more objects compete, the more slowly should each object be processed. We ask whether this competition is restricted to fixations, periods of relatively stable gaze, or whether it extends from one fixation to the next, across intervening saccades. Participants made saccades to a peripheral target-object. They reported a letter shown after the saccade within this saccade target. The letter lasted for different durations (mask-terminated). Processing speed of this letter was assessed by modeling report performance as a function of letter duration. Either no, two, or four additional non-target objects appeared before the saccade. In Experiment 1, presaccadic non-targets were task-irrelevant and had no effects on postsaccadic processing speed of the letter. In Experiment 2, presaccadic non-targets were task-relevant because participants matched them against a probe at trial end. Here, postsaccadic processing speed decreased with increasing number of presaccadic non-targets. These findings show that objects compete for recognition across saccades, but only if they are task-relevant. This reveals an attentional mechanism of task-driven object recognition that is interlaced with active saccade-mediated vision (Schneider, 2013; Poth, Herwig, & Schneider, 2015).

**Remapping of the global effect across saccades**

**Kiki Arkesteijn, Jeroen BJ Smeets, Mieke Donk, Artem V. Belopolsky** VU Amsterdam, Netherlands 

k.arkesteijn@vu.nl 

When a distractor is presented in close spatial proximity to a saccade target, a saccade will land in between the two objects. This is known as the global effect. In the present study we investigated whether the global effect is retained across saccades. Participants performed a sequence of a horizontal and a vertical saccade and the global effect was induced by presenting a distractor next to the second saccade target. Importantly, the distractor was removed before the eyes landed on the first saccade target. On half of the trials the second target was stationary and on the other half it disappeared after the first saccade, resulting in a memory guided saccade. Despite the disappearance of the distractor after the first saccade, there was a global effect of distractor on the landing position of the second saccade. Notably, this was only the case when the second saccade was guided from memory. The results suggest that when programming a sequence of saccades, distractor information that is available before the start of the first saccade is remapped together with the second target. However, when the second target remains present, the planned saccade can be corrected, resulting in the elimination of the global effect.

Special Thematic Session: Communication by gaze interaction II

**Room 3 **(HS 28 - I.13.71)

**Gaze interaction using low-resolution images at 5 FPS**

**Carlos H. Morimoto, Carlos E. L. Elmadjian, Antonio Diaz-Tula, Fernando O. Aluani** University of São Paulo, Brazil hitoshi@ime.usp.br 

With trackers gradually becoming personal wearable devices, gaze-based interaction will become a relevant technique for wearable applications. However, it is a common belief that high-resolution images and high frame rates are desirable to achieve the accuracy and precision required for human interaction. Because of the high computational load, a wearable eye tracker would have their batteries quickly drained out. In this paper we investigate how much processing power can be saved by lowering these requirements, and still maintain the performance adequate for human interaction. We have conducted an experiment using a head-mounted Pupil Labs eye tracker. Our results from 10 participants show that accuracy and precision remain below one degree of error for image resolution of 240 lines, and frame rates as low as 5 frames per second (FPS). Using this minimum setup, we estimate that power consumption can be reduced by 90\% compared to the eye tracker camera regular settings (480 lines and 30 FPS). We also propose an algorithm that successfully detects reading behavior in real-time at 5 FPS in order to demonstrate the usefulness of gaze data at such low rates.

**PSOVIS: An interactive tool for extracting post-saccadic oscillations from eye movement data**

**Diako Mardanbegi, Thomas Wilcockson, Baiqiang Xia, Hans Gellersen, Trevor Crawford, Peter Sawyer**

Lancaster University, United Kingdom

d.mardanbegi@lancaster.ac.uk

Post-microsaccadic eye movements recorded by high frame-rate pupil-based eye trackers reflect movements of different ocular structures such as deformation of the iris and pupil-eyeball relative movement as well as the dynamic overshoot of the eye globe at the end of each saccade. These PostSaccadic Oscillations (PSO) exhibit a high degree of reproducibility across saccades and within participants. Therefore in order to study the characteristics of the post-saccadic eye movements, it is often desirable to extract the post-saccadic parts of the recorded saccades and to look at the ending part of all saccades. In order to ease the studying of PSO eye movements, a simple tool for extracting

PSO signals from the eye movement recordings has been developed. The software application implements functions for extracting, aligning, visualising and finally exporting the PSO signals from eye movement recordings, to be used for post-processing. The code which is written in Python can be download from https://github.com/dmardanbeigi/PSOVIS.git

**GazeBall: Leveraging Natural Gaze Behavior for Continuous Re-calibration in Gameplay**

**Argenis Ramirez-Gomez, Hans Gellersen**

Lancaster University, United Kingdom

a.ramirezgomez@lancaster.ac.uk

Eye tracking offers opportunities to extend conventional game control with gaze input for multimodal game interaction. Gaze, however, has been found challenging to use as it requires re-calibration over time and for different users, in order to maintain an accurate input. In this work, we propose to leverage the natural gaze behavior that users exhibit during gameplay for implicit and continuous re-calibration. We demonstrate this with GazeBall, continually calibrating players' gaze based on their natural ocular pursuit of the game's ball movement. Re-calibration enables the extension of the game with a gazebased `power-up'. In the evaluation of GazeBall, we show that our approach is effective in maintaining highly accurate gaze input over time, while re-calibration remains transparent to the player.

**Implicit Events in Virtual Reality: A New Concept for Eye-Based Interaction?**

**Teresa Hirzle, Jan Ehlers, Anke Huckauf, Enrico Rukzio** Ulm University, Germany teresa.hirzle@uni-ulm.de

Pupil size is a sensitive physiological information channel that reveals internal states of a person. Furthermore, pupil responses are caused by implicit events, which occur usually without noticing it. The current study makes use of these events by presenting an eye-based interaction technique based on attention shifting. As the pupil especially reacts to changes of illumination and environmental noise, the study is conducted using a virtual reality headset with integrated eye tracking. Results show that focusing attention on a target correlates with increased pupil diameters, whereas the simple observation of neutral objects does not provoke a pupil reaction. Therefore, we conclude that using implicit events as interaction technique in eye-based Human-Computer Interaction scenarios is a promising approach.

Thematic : Reading: Spatially distributed processing

**Room 4** (HS 26 - I.13.65)

**Two routes of parafoveal processing during reading: Eye movements suggest benefits and costs**

**Sarah Risse**^1^**, Martin R. Vasilev**^2^

^1^University of Potsdam, Germany; 

^2^Bournemouth University, United Kingdom sarah.risse@uni-potsdam.de

Reading requires the spatiotemporal integration of both foveal and parafoveal vision. Still unresolved is the question as to when parafoveal preview is beneficial and when it is not. The present gazecontingent experiment manipulated the validity and the difficulty of the parafoveal word n+1, while the integration of parafoveal into foveal information was delayed for varying time intervals. Two preview effects were dissociated, and their different time courses indicated two ways of utilizing parafoveal information. First, valid preview of the target word reduced its foveal processing time, but only when the integration process could start within less than 80 ms. Second, previewing a difficult upcoming word had a disruptive effect on target fixations independent of the onset of foveal processing. These results are in agreement with previous findings that the classical parafoveal preview benefit is a mixture of processing benefits and costs. Moreover, a preview difficulty effect across the full range of fixation durations contradicts explanations based on neural delays such as the eye-brainlag or forced fixations. We will discuss extensions of reading models that allow parafoveal information to affect temporal oculomotor decisions via two different routes of parafoveal processing.

**Late interference by parafoveal difficulty in reading**

**Stefan Seelig, Sarah Risse**

Universität Potsdam, Germany sseelig@uni-potsdam.de 

During fixations in sentence reading, useful information is acquired both from foveal and adjacent parafoveal words. Using the gaze contingent boundary paradigm, it is well established, that reducing parafoveally available information about the postboundary target word n+1 during fixations of the preboundary word n prolongs fixations on the target. This is typically interpreted as a consequence of reduced facilitation of foveal processing, whereas interference between preview and target is often dismissed due to inconclusive findings on immediate effects of the preview on pretarget fixation durations. However recent results suggest an interplay between facilitation and interference by preview processing being delayed into the subsequent fixation on the target word. We sought to rule out alternative explanations for these results in a new experiment by varying the previews processing difficulty (via frequency) while keeping target difficulty constant. We found substantial effects of preview difficulty on n+1 fixation durations that can only be explained with delayed or ongoing processing of the preview after crossing the boundary, rather than mere facilitation. We further discuss the impact of the findings on direct vs. indirect control of eye movements and present simulation results for different mechanisms to incorporate the findings in the SWIFT model of reading. 

**Analyzing Sequential Dependencies between Fixation Durations with Linked Linear Mixed Models**

**Reinhold , Sven Hohenstein, Hannes Matuschek**

University of Potsdam, Germany kliegl@uni-potsdam.de 

We address a problem with the current practice of separate analyses of successive fixation durations during reading. The problem is that we often report separate analyses for each of two or more possibly correlated measures--for example, separate analyses of fixation durations of pretarget and target word in gaze-contingent display change experiments (i.e., the boundary paradigm; Rayner, 1975). Classic interpretations of preview benefit (e.g., the difference in fixation durations on target words following a semantically unrelated or related preview) assume that the preview effect is not moderated by the duration of the fixation before the boundary. This assumption was violated in a number of studies, but, of course, these interactions are informative. In such an analysis, we specify durations on target words as dependent variable and treat the duration before the boundary as a covariate (i.e., independent variable). This specification does not adequately capture that words in the perceptual span may affect both fixation durations simultaneously. We propose a variant of linked linear mixed models (LLMMs; Hohenstein, Matuschek, & Kliegl, 2016) as an alternative for modeling more than one dependent variable simultaneously taking into account the order of fixations inherent in the reading process.

**What are the costs of degraded parafoveal previews during silent reading?**

**Bernhard , Martin R. Vasilev, Timothy J. Slattery, Julie A. Kirkby**

Department of Psychology, Bournemouth University, United Kingdom bangele@bournemouth.ac.uk

It has been suggested that the preview benefit effect is actually a combination of preview benefit and preview costs. Marx et al. (2015) proposed that visually degrading the parafoveal preview reduces the costs associated with traditional parafoveal letter masks used in the boundary paradigm (Rayner,1975), thus leading to a more neutral baseline. We report two experiments of skilled adults reading silently. In Experiment 1, we found no compelling evidence that degraded previews reduced processing costs associated with traditional letter masks. Moreover, participants were highly sensitive to detecting degraded display changes. Experiment 2 utilized the boundary detection paradigm (Slattery, Angele, & Rayner, 2011) to explore whether participants were capable of detecting actual letter changes or if they were responding purely to changes in degradation. Half of the participants were instructed to respond to any noticed display changes and the other half to respond only to letter changes. Participants were highly sensitive to degraded changes. In fact, these changes were so apparent that they reduced the sensitivity to letter masks. In summary, degrading parafoveal letter masks did not reduce their processing costs in adults, but both degraded valid and invalid previews introduced additional costs in terms of greater display change awareness.

**Effects of font type on parafoveal letter identification in Russian**

**Svetlana Alexeeva, Aleksandra Dobrego, Alena Konina**

St. Petersburg State University, Russian Federation mail@s-alexeeva.ru 

There is an that in alphabetic writing systems printed words are recognized via their constituent letters. Eye-movement research previously demonstrated that readers start acquiring letter information parafoveally (Lima&Inhoff, 1985). We aimed at measuring legibility of Russian (Cyrillic) letters presented in the parafovea and identifying how the font type mediates the process. In two boundary experiments differing in font (fixed-width Courier New vs. proportional Georgia), after focusing on a fixation cross at the center of the screen, participants saw a letter (*ы*) parafoveally and had to name it. Letters disappeared during the saccade from the cross to the stimulus. Results showed that subjects produced significantly fewer errors when letters were rendered in Georgia than in Courier New (22% vs. 42%). Louvain clustering algorithm based on detection errors revealed 5 letter confusion classes for Courier New font and 7 for Georgia. This supports the idea (Pelli et al. 2006) that letter efficiency varies across fonts. Independent of font, ascenders/descenders and round envelopes were among the most accurate letters to recognize whereas “part-of-the-whole” letters (г-т) deteriorated identification. The results confirm feature-based letter perception. These first confusion matrices of Russian can be used to generate orthographic controls in reading studies. Funded by RSF#14-18-02135. 

**Orthographic, Morphological, and Semantic Parafoveal Processing in Arabic Reading: Evidence from the Boundary Paradigm**

Ehab W. Hermena^1^, Eida J. Juma^1^, Ascensión Pagán^2^, Maryam AlJassmi^1^, Mercedes Sheen^1^, Timothy

**R. Jordan**^1^

^1^Zayed University, United Arab Emirates; ^2^University of Oxford, UK ehab.hermena@zu.ac.ae 

Evidence clearly shows that skilled readers extract information about upcoming words: Parafoveal previews that share relevant information with the target word result in facilitation of target word processing once this target is fixated (e.g., Deutsch et al., 2000; Rayner, 2009; Schotter, 2013; Schotter et al., 2012). Using the boundary paradigm (Rayner, 1975), we investigated native Arabic readers’ processing of orthographic, morphological, and semantic information available parafoveally. Target words were embedded in sentences, with one of the following preview conditions: (a) Identical; (b) Preview sharing root morpheme with the target; (c) Preview sharing form morpheme with the target; (d) Preview sharing all letters with the target, but the root letters were transposed creating a nonsense string with an unknown root; (e) Same as (d) but the root letter transposition created a new word with a known root; (f) Preview that is a synonym with the target; and finally (g) Unrelated previews. Results suggest that previews that share intact target roots (condition a), or all target letters (condition d) produce facilitation. In contrast, previews containing new known roots (condition e) result in increases in target processing time. The effects of pre-processing shared orthographic, morphological, and semantic information parafoveally will be discussed.

Tuesday, 22^nd^, 10.30 - 12.30 

Symposium: Longitudinal research on eye movements in developing readers: What have we learned so far?

**Room1** (HS 14 - M.10.12)

**The development of eye movement control in reading: where do the eyes go?**

**Ralph Radach**^1^**, Christian Vorstius**^1^**, Chris Lonigan**^2^

^1^Bergische Universität Wuppertal, Germany; 

^2^Florida State University, Department of Psychology, United States of America radach@uni-wuppertal.de 

We report data from a large cross-sectional and longitudinal sample, collected with students in grades 1 to 5 as part of the Florida Reading for Understanding project. Eye movements were recorded while children were reading declarative sentences with identical content across grades. Analyses of saccade landing positions and refixation probability curves indicate that beginning readers maintain a primarily sequential, sub-lexical reading strategy, with near-adult behavior emerging at a later point in time. As an example, the positions of fixations made in early reading are less distant but spread across a larger area within words. The word length effect on initial landing position known from adult data emerges over time. We conclude that the principle low-level constraints of eye movement control as described by McConkie et al. (1988) also apply to young children. However, detailed analyses of local fixation patterns suggest an initial dominance of deliberate, strategic viewing behavior in the service of sublexical decoding. There was little influence of orthographic regularity on saccade landing position in very young readers, suggesting lack of effective extrafoveal processing. Apparently, much of the automated oculomotor routines we observe in skilled adult reading are a consequence, rather than a basis of development towards skilled reading.

**Early development of oculomotor control in reading: a longitudinal eye tracking study from preschool age to fifth grade**

**Thomas Günther**^1^**, Josefine Horbach**^1^**, Wolfgang Scharker**^1^**, Ralph Radach**^2^

^1^RWTH Aachen, Germany; 

^2^Bergische Universität Wuppertal, Germany tguenther@ukaachen.de 

The present work examines the development of eye movement control from preschool age to fifth grade in German children. In addition to sentence reading, participants performed the Landolt scanning task (LT). Participants scan lines in which letters are replaced with closed Landolt rings, except for some open rings serving as targets. The present work examined to what extent preschoolers (n=292) are able to perform the LT and how their oculomotor behavior is related to sentence reading at the end of first grade (n=261), second grade (n=230), fourth grade (n=189) and fifth grade (n=130). In contrast to adult readers, beginning readers strongly prefer to place their initial fixations at word beginnings. Interestingly, oculomotor behavior in the LT is becoming more ‘reading like’ with increasing reading experience. In a transparent language like German the default initial landing position is located at the beginning of the word, suggesting a strong preference for a sub-lexical reading strategy. Furthermore, the data suggest an extreme developmental jump between the first and second year and a strong reduction in variability between and within the children. In addition, the LT has proven to be a useful paradigm to examine nonlinguistic processing components in the early development of reading.

**Foveal Processing Difficulty Modulates Perceptual Span Early in Reading Developmemt**

**Johannes M. , Jochen Laubrock**

Universität Potsdam, Germany johannes.meixner@uni-potsdam.de

The perceptual span describes the useful visual field during reading, which contributes to efficient reading. The perceptual span starts to develop from first to second grade, makes a qualitative jump from second to third grade and likely reaches adulthood level at sixth grade. Here, we examine how children’s perceptual span develops for several word-based measures and whether the perceptual span adaptively becomes smaller if foveal processing is difficult. To this end, we applied the moving window paradigm to three cohorts of German young readers in grades one to six at three waves. The wordbased analysis confirmed the qualitative jump from second to third grade, with fixation-duration and first-pass measures saturating earlier than saccade-targeting and second-pass measures. Already for secondgraders, we found that frequency effects of the previous word N on first fixation duration on the upcoming word N+1 increased with larger windows. Fixation durations on N+1 decreased with highfrequency words N, but only when sufficient preview was available. This provides strong support for the foveal load hypothesis that the size of the perceptual span is locally adjusted if foveal processing is difficult. Finally, we show brand-new data of the fourth wave featuring sixth- to eighth-graders and compare these to adults.

**Comprehension in silent and oral sentence reading. Longitudinal evidence from developing readers**

**Christian Vorstius**^1^**, Young-Suk G. Kim**^2^**, Ralph Radach**^1^

^1^University of Wuppertal, Germany;

^2^University of Irvine, California, United States of America vorstius@uni-wuppertal.de 

Data presented in this talk are part of the Florida Fluency Development Project. In this project we collected data from N=400 English native speaking children, who were followed from first through third grade. Eye movements as well as psychometric assessments were collected twice in each grade for a total of six assessment periods. Reading material consisted of single line sentences, taken from the Test of Silent Reading Comprehension (TOSREC). Using a psychometric assessment combined with eye tracking allows illuminating the online processes resulting in reading comprehension. In addition to the overall comprehension score, item level comprehension was used for analyses. Finally, we included a variation in reading mode (silent vs. aloud) to study its effect on comprehension. Results show a steep decrease in reading times for grades one and two and a less pronounced decrease throughout third grade, for both silent and oral reading. Regarding comprehension, the overall standardized scores did not differ between reading modes. For the item level comprehension data, we found that reading times were increased in sentences where children showed poor comprehension, again with no differences between reading modes. The lack of trade-off between reading time and comprehension will be discussed based on various theoretical approaches.

**The development of foveal eye movements in primary school: Findings from the Berlin DevTrack study**

**Sascha Schroeder, Simon Tiffin-Richards, Sarah Eilers**

Max Planck Institute for Human Development, Germany sascha.schroeder@mpib-berlin.mpg.de

In this talk we present data from the Berlin DevTrack study which investigated the development of N = 70 German speaking children’s eye movements from grade 2 to 4 using a longitudinal design. To assess foveal reading, children read single sentences each comprising a target word that was manipulated for length (short vs. long) and frequency (high vs. low). In addition, an extensive set of cognitive tests assessing verbal and non-verbal skills was administered each year. Generally, results indicate that the development of children’s foveal reading is influenced by complex interactions between item- and participant characteristics. Growth curve models show that children’s gaze durations on target words decreased during reading development according to a quadratic function. In addition, length and frequency effects decreased substantially with age with particularly strong changes for long, lowfrequency words. Interindividual differences in children’s growth trajectories were mainly associated with development changes in verbal skills, while non-verbal skills, such as oculomotor control or visual attention, did not strongly affect eye movement development. Results are discussed with regard to current models of the development of children’s eye movements during reading acquisition.

Thematic Session: Clinical Research I

**Room 2** (HS 32 - K.11.23)

Implicit and explicit oculo-motor learning in Parkinson’s disease and

**spinocerebellar ataxia**

**Andreas Sprenger**^1,2^**, Annika Lasrich**^1^**, Christoph Helmchen**^1^

^1^Department of Neurology, University Luebeck, Germany; 

^2^Institute of Psychology II, University Luebeck, Germany andreas.sprenger@neuro.uni-luebeck.de

Motor learning is a fundamental skill in everyday life. Particularly patients with Parkinson’s disease and spinocerebellar ataxia are affected but these patient groups fundamentally differ in their behaviour in motor learning tasks. The aim of the current study was to test differences or similarities in implicit and explicit motor learning tasks. Healthy subjects were able to adapt and stored the adapted gain between the sessions. PD patients were able to adapt to the transient displacement of the target but did not store the information between learning sessions. Patients with SCA did not adapt in the first run but in the second run. In the explicit motor learning task, all participants showed a general motor learning. Patients performed better in the implicit learning trials but needed twice the time of healthy participants. PD patients were not impaired in the implicit motor adaptation task. However, their storage of motor learning is selectively impaired possibly due to reduced functioning of cortico-striatalcerebellar loops and their connections to motor memory systems. SCA patients, in contrast, lack implicit saccade learning because the cerebellum is crucially involved in saccade adaptation. The moderate storage of the adapted saccade gain suggests signal processing by the cortico-striatal-cerebellar network.

**Visual exploration of emotional faces in schizophrenia using masks from the Japanese Noh theatre**

Teresa Fasshauer^1^, Andreas Sprenger^2^, Karen Silling^1^, Christopher Zeiser^3^, Johanna E. Silberg^1^, Anne

**Vosseler**^1^**, Seiko Minoshita**^4^**, Michael Dorr**^3^**, Katja Koelkebeck**^1,5^**, Rebekka Lencer**^1,5^

^1^Department of Psychiatry and Psychotherapy, University of Muenster, Germany;

^2^Department of Neurology, University of Luebeck, Germany; 

^3^Chair of Human-Machine Communication, Technical University of Munich, Germany;

^4^Department of Psychology, Kawamura Gakuen Woman’s University, Abiko, Chiba, Japan;

^5^Otto-Creutzfeldt for Cognitive and Behavioral Neuroscience, University of Muenster, Germany

teresa.fasshauer@arcor.de

We used images of masks from the Japanese Noh-theatre to investigate face and emotion recognition abilities in patients with schizophrenia and their possible deficits in bottom-up information processing. Eye movements were recorded in 25 patients with schizophrenia and 25 age-matched healthy controls while participants explored seven photos of Japanese Noh-masks tilted in seven different angles, seven binary black and white images of these Noh-masks (Mooney images), seven Thatcher images (180° upside-down turned Mooneys), and seven neutral images. Participants indicated either whether they had recognized a face and its emotional expression, or they had to evaluate the brightness of the image (total N=56 trials). We observed a clear effect of inclination angle of Noh-masks on emotional ratings (p<0.001) and visual exploration behavior in both groups. Controls made larger saccades than patients when not being able to recognize a face in Thatcher images (p<0.01). Patients also made smaller saccades when exploring images for brightness (p<0.05). Exploration behavior in patients was related to clinical symptom expression during face/emotion recognition but not during brightness evaluation. Our findings suggest that patients with schizophrenia are not generally but specifically impaired in adjusting their visual exploration behavior to task conditions depending also on clinical symptom expression.

**Visual Behavior on Natural Static Images in Patients with Retinitis Pigmentosa**

**Ricardo R. Gameiro**^1^**, Kristin Jünemann**^1^**, Anika Wolff**^2^**, Anne Herbik**^2^**, Peter König**^1,3^**, Michael Hoffmann**^2^

^1^Institute of Cognitive Science, University of Osnabrück, Germany; 

^2^Visual Processing Lab, Ophthalmic Department, Otto-von-Guericke-University Magdeburg, Germany;

^3^Institute of and Pathophysiology, University Medical Center Hamburg-Eppendorf, 

Germany

rramosga@uni-osnabrueck.de

Retinitis pigmentosa is a disease that causes peripheral visual field loss. Here, we investigated how the loss of peripheral vision affects visual behavior on natural images. Patients with varying degree of visual field loss and control participants freely observed images of three different sizes while eye movements were recorded. We examined whether visual behavior differs when the scene content is shown in varying extends of the visual field, investigating the spatial bias, saccade amplitudes, as well as the amount and duration of fixations. We found that controls and patients with moderate loss of peripheral vision showed a central spatial bias while observing the images. Patients with a severe loss showed individual exploration and systematically scan the whole image area especially on large images. According to saccades amplitudes, controls and patients with moderate loss preferred making short saccades throughout all image sizes. Patients with a severe loss made a higher number of large saccades on large images. The number of fixations increased with an enlarging loss of peripheral vision, while fixation durations decrease. In conclusion, RP patients scan the images more strategically when the observed scene exceeds their visible field. Down-sizing the scene yields an exploration similar to healthy visual behavior.

**Quantifying Traumatic Brain Injury impairments in scanning patterns of complex scenes**

**Nitzan Guy, Oryah Lancry, Yoni Pertzov**

Hebrew University of Jerusalem, Israel guynitzan@gmail.com 

Traumatic brain injury (TBI) may lead to changes in eye movements, which potentially hamper daily tasks. Therefore, it is important to understand how previously reported impairments in basic eye movement tasks are reflected in more ecological settings. We have measured gaze position of TBI patients and matched controls in two different tasks. First we assessed performance in a self-paced saccadic task in which subjects are required to move their gaze as fast as possible between two points. We have used it as a measure for eye movement impairment in controlled, un-ecological environment. Next we assessed a more ecological condition in which subjects scanned images while freely moving their eyes. This enabled us to explore the relationship between eye movement impairments in controlled tasks and more ecological daily tasks. Our results reveal two main findings: TBI patients perform more blinks and move their eyes less than controls in all tasks. The occurrence of this behavior across tasks is an indication of its robustness and provides evidence for abnormalities in TBI patients’ sampling of the world in daily life. We conclude that free viewing tasks may help in assessing the severity of TBI and in quantifying their impairments in daily behavior.

**Smooth pursuit disturbances in schizophrenia during free visual exploration of dynamic natural scenes**

Johanna E. Silberg^1^, Ioannis Agtzidis^2^, Mikhail Startsev^2^, Teresa Fasshauer^1^, Karen Silling^1^, Andreas

**Sprenger**^3^**, Michael Dorr**^2^**, Rebekka Lencer**^1,4^

^1^University of Münster, Germany; 

^2^Chair of Human-Machine Communication, University of Technology, Munich, Germany; 

^3^Department of Neurology and Institute of Psychology II, University of Luebeck, Germany; 

^4^Otto-Creutzfeldt for Cognitive and Behavioral Neuroscience, University of Muenster, Germany j_silb01@uni-muenster.de 

Eye tracking dysfunction (ETD) observed with standard pursuit stimuli represents a well-established biomarker for schizophrenia. Whether ETD manifests during free visual exploration of natural dynamic scenes is unclear. Eye movements were recorded (EyeLink®1000) while 26 schizophrenia patients and 25 healthy age-matched controls freely explored 9 videos and 9 pictures of daily life situations each lasting 20s (http://www.inb.uni-luebeck.de/tools-demos/gaze). Subsequently, participants had to decide whether they had explored still-shots of the stimuli as videos or pictures. Patients made smaller saccades on videos (p=0.002) and pictures (p=0.001) and longer fixations on pictures (p=0.043). Patients’ center bias was stronger and their exploration behavior was less driven by stimulus saliency than in controls on both videos and pictures. Proportions of pursuit tracking differed between groups depending on the individual video (group*video p=0.041, video p<0.0001) and patients showed generally lower motion recall scores (p=0.035). They were also impaired during pursuit of standard triangular wave stimuli (p=0.03) which was not correlated with visual exploration behavior of movies. Our results suggest restricted visual exploration patterns in patients not only on pictures but also on uncutted real life movies, while ETD observed with standard stimuli was not directly related to visual exploration of real life situations. 

**Eye tracking live social interaction to capture gaze behavior of subclinical autism and social anxiety**

Roy S. Hessels^1,2^, Gijs A. Holleman^1^, Tim H. W. Cornelissen^3^, Ignace T. C. Hooge^1^, Chantal Kemner^1,2,4^

^1^Experimental Psychology, Helmholtz Institute, Utrecht University, Netherlands;

^2^Developmental Psychology, Utrecht University, Netherlands; 

^3^Scene Grammar Lab, Department of Cognitive Psychology, Goethe University Frankfurt, Germany; ^4^Brain Center Rudolf Magnus, University Medical Center Utrecht, Netherlands 

r.s.hessels@uu.nl 

In typical face-processing experiments, static representations of faces are used (e.g. pictures or schematic faces). However, in social interaction the content of faces is more dynamic and dependent on the interplay between interaction partners than the content of a non-responsive face. While research using static representations has resulted in a plethora of knowledge on the sub-systems of facial information processing, recent evidence suggests that generalizability of these findings to social situations may be limited. In the present study we used a novel dual eye-tracking setup to investigate whether gaze behavior in interaction is related to (sub)clinical traits of Autism Spectrum Disorder (ASD), and Social Anxiety Disorder (SAD). We report that gaze behavior of individuals scoring high on ASD and SAD to the face of an interaction partner corroborates long-standing findings obtained using static pictures and videos. Moreover, we report that pairs scoring high on ASD or SAD show marked differences in paired gaze states as compared to pairs scoring low on these traits. These findings provide intriguing possibilities for the investigation of gaze behavior in interaction, and attest to the sensitivity of gaze behavior in dyadic interaction to (sub)clinical psychopathology. 

Thematic session: Visual interfaces, robotics and virtual reality

**Room 3** (HS 28 - I.13.71)

**Smooth pursuit based mouse replacement: the GazeEverywhere system**

**Simon Schenk, Marc Dreiser, Philipp Tiefenbacher, Gerhard Rigoll, Michael Dorr**

Technical University Munich, Germany simon.schenk@tum.de 

In gaze-based human computer interaction (HCI), the most commonly used mouse replacement technique is dwell time (DT). This method, however, is prone to the Midas touch problem, i.e. naturally occurring fixations are misclassified as interaction dwells. Here, we present the GazeEverywhere solution, a gaze-only mouse replacement that comprises of two components: i) the SPOCK interaction method which is based on smooth pursuit eye movements and does not suffer from Midas touches; ii) an online recalibration algorithm that continuously improves gaze-tracking accuracy. We conducted four user studies to evaluate different aspects of our solution: In a first experiment, we prove that SPOCK has fewer misclicks than DT (minus 93%), especially under high mental workload. In two followup studies, we show that SPOCK has a higher throughput than DT (14% increase) according to ISO 92419, and that the online recalibration reduces the minimal interaction target ('button') size by about 25%. Finally, a case study shows that users were able browse the internet and successfully run Wikiraces using gaze only. For a broader accessibility, we also present an optional hardware setup, which can be used to superimpose the required visual stimuli purely in hardware and without software modifications.

**Fixation-Related Potentials as a Measure for Cognitive Demand in Visual Tasks on Single Trial Level**

**Dennis Wobrock**^1^**, Andrea Finke**^1^**, Shirley Mey**^2^**, Dirk Koester**^2^**, Thomas Schack**^2^**, Helge Ritter**^1^

^1^Neuroinformatics Group, CITEC, Bielefeld University, Germany; 

^2^Neurocognition and Action Group, CITEC, Bielefeld University, Germany dwobrock@techfak.uni-bielefeld.de 

While technical systems grow in complexity, human-machine interaction (HMI) still requires the user to adapt to the tools. By combining electroencephalography (EEG) and eye-tracking, we aim to create a multi-modal brain machine interface (BMI) to improve system adaptivity and resource efficiency in HMI. We propose to analyze Fixation-Related Potentials (FRP) as indicators for cognitive demand and utilize this measure to automatically adapt a system to the user. To this end, we conducted two complementary studies to investigate variations between different tasks, i.e., an object counting task in cluttered scenes and a subjective choice task. Comparing the FRP grand averages (per task category over all trials and participants), we identified significant amplitude and frequency differences in early fronto-central N100 and P200 components. The dataset combining both studies was linearly classified with 66% (SD: 2.0) accuracy using temporal features, but 93% (SD: 1.8) using frequency features. This suggests that early FRP components vary with task complexity during natural scene exploration. Our results also suggest that cognitive demand may be identified on a single trial level using the proposed multi-modal approach. Thus, EEG data may contain discriminative information that eye-tracking data cannot provide alone, e.g., through fixation duration. 

**Gaze Control of Vergence, Yaw and Pitch of Robotic Eyes for Immersive Telepresence**

**Remi Cambuzat**^12^**, Frédéric Elisei**^1^**, Gérard Bailly**^1^

^1^GIPSA-lab, Univ. Grenoble-Alpes, France; 

^2^CITI-Inria Laboratory, INSA Lyon, France remi.cambuzat@gipsa-lab.grenoble-inp.fr 

Telepresence refers to a set of tools that allows a person to be “present” in a distant environment, by a sufficiently realistic representation of it through a set of multimodal stimuli experienced by the distant devices via its sensors. Immersive Telepresence follows this trend and, thanks to the capabilities given by virtual reality devices, replicates distant sight and sound perception in a more “immersive” way. The use of coherent stereoscopic images displayed in a head mounted display, and natural control of a robotic head collocated with the orientation of the pilot head, help the pilot to feel “embodied” in the distant robotic platform. However even if the actual frameworks have shown increased awareness of the remote scene and enhanced interactivity, no work has currently addressed the challenge of gaze contingent controlled robotic eye in immersive teleoperation (ie. eye vergence) and its impact on scene awareness from the pilot and sense of presence from the remote interlocutors. Based on gaze driven technologies and analysis in related fields of study, we propose and evaluate a set of methods to quantify the impact of the proposed Stereo Gaze Contingent Steering (SGCS) of Robotic Eyes, notably on depth perception and near-field object perception.

**Measurement of Situation Awareness in Collaborative Robotics Using Eye Movement Features**

**Lucas Paletta, Cornelia Murko, Amir Dini**

JOANNEUM Forschungsgesellschaft mbH, Austria lucas.paletta@joanneum.at 

Collaborative robotics requires human factors analysis: human-related variables are essential to evaluate human-robot interaction (HRI) metrics (Steinfeld et al., 2006). Robots will rely on predictions of human worker’s behavior, emotions, and intents to plan actions (Huang & Mutlu, 2016). Particularly, measuring human situation awareness is mandatory for the understanding of delayed action planning (Endsley, 1995). Interrupting questionnaire technologies of SART (Taylor, 1990) and SAGAT (Endsley, 2000) required less invasive technologies, such as, eye tracking (Moore & Gugerty, 2010). However, aposteriori analysis from 2D displays is not applicable to HRI in 3D environments. This work investigated real-time measured eye movements for the correlation with situation awareness (SA) metrics in HRI multiple task switching. Dwell rate and time, turn rate, and fixation distribution analysis by NNI indexing (Camilli et al., 2008) were recovered from probabilistic object localizations by gaze for SA estimation and prediction. NNI was extended to 3D metrics by projection of eye movements on objects of interests. Results enable 83.3% accuracy in SAGAT, 91.7% accuracy in SART and performance classification, respectively. Gaze based metrics enable SA estimation in HRI during real-time tasks. Estimation of human SA is crucial for the elaboration of performance, acceptance, and executive function analysis.

**Siamese Convolutional Neural Networks for Appearance-Based Gaze Estimation** **David Mayo, Helen Zhou, Scott Greenwald**

MIT Media Lab, United States of America dmayo2@mit.edu

With increased availability of low-cost VR displays, the need for more natural, robust, and low-cost input methods has become more salient. In our talk, we present methods of appearance-based gaze estimation which allow us to utilize existing hardware, such as low-cost visible spectrum cameras, contained in mobile devices or VR headsets. We will discuss the performance of state-of-the-art CNNs trained on three distinct datasets: (1) MPIIGaze images (Zhang et. al.), (2) UnityEyes simulated data (Wood et. al.), and (3) our own dataset of eye images captured using a phone in a Google Cardboard. Motivated by traditional calibration techniques, we present a siamese CNN architecture which takes in an image of the eye (whose gaze we want to predict), along with a reference image (of the eye looking straight forward). We also propose a method that takes advantage of the VR display’s reflection onto the eye. This reflection is particularly prominent in VR headsets, and entirely controlled. Following Shih et. al.’s work with ghosting cues, we plan to separate the deformed reflection from the image of the eye. This deformed reflection will then be input along with a reference image of the VR display into a siamese network.

**Joint visual working memory through implicit collaboration**

**Edwin S. Dalmaijer**^1^**, Diederick C. Niehorster**^2,3^**, Kenneth Holmqvist**^4^**, Masud Husain**^1,5^

^1^Department of Experimental Psychology, University of Oxford, United Kingdom;

^2^The Humanities Lab, Lund University, Sweden; 

^3^Department of Psychology, Lund University, Sweden; 

^4^UPSET, North-West University (Vaal Triangle Campus), South Africa; ^5^Nuffield Department of Clinical Neuroscience, University of Oxford, United Kingdom edwin.dalmaijer@psy.ox.ac.uk

Visual memory (VWM) is a limited resource used to temporarily store information on elements in our environment. The total capacity of VWM differs between individuals. Allocation of VWM resources is highly flexible, and can be biased towards reward-associated items. The current study is the first to explore joint visual working memory, using a task where participants collaborate in pairs of two. Specifically, we present the same memory array of several oriented Gabor patches to each pair. Using a group eye tracking setup, participants were shown in real-time which stimuli their partner had fixated. Individuals claimed Gabors by fixating them, and after a pair had fixated all, each individual was probed on one of their claimed elements. Participant pairs were rewarded according to their combined performance (recall errors of the stimulus orientation on a continuous scale). Because individual rewards are shared, optimal behaviour would be to divide the stimuli according to each individual's VWM capacity to maximise reward. This experiment shows to what extent participants can form a shared task representation and distribute VMW between them optimally.

Thematic Session: Scanpaths

**Room 4** (HS 26 - I.13.65)

**Disentangling fixation duration and saccadic planning using gaze dependent guided viewing**

**Benedikt V. Ehinger**^1^**, Lilli Kaufhold**^1^**, Peter König**^1,2^

^1^Osnabrück , Germany; 

^2^Department of Neurophysiology and Pathophysiology, University Medical Center Hamburg-

Eppendorf, Hamburg, Germany behinger@uos.de 

When scanning a scene, we are in a constant decision process whether to further exploit the current fixation or to move on and explore the scene. The balance of these processes determines the distribution of fixation durations. Using a new paradigm, we experimentally interrupt these processes to probe their current state. Here, we developed a guided viewing task where subjects fixated a small bubble (3°) for a fixed period of time. The bubble disappeared and one to five new bubbles emerged at different locations. Subjects performed a gaze-contingent saccade onto one of them. By repeating this procedure, subjects explored the image. We modeled the resulting saccadic reaction times (choicetimes) from bubble offset to saccade onset using a Bayesian Linear Mixed Model. We observe an exponential decay between the fixed period and the choicetimes: Short fixation durations elicit longer choicetimes. This suggests that the sampling and processing of the current stimulus is exhausted for long fixation durations, biasing towards faster exploration. In trials with multiple bubbles, choicetimes increase monotonically, showing that the decision process takes into account processing demands at the current fixation location. We discuss how this paradigm allows for experimental control of fixation duration as well as fixation location.

**The early central fixation bias in scene viewing: Experimental manipulation and modeling**

**Lars O. M. Rothkegel**^1^**, Hans A. Trukenbrod**^1,3^**, Heiko H. Schütt**^1,2^**, Felix A Wichmann**^2,4,5^**, Ralf**

**Engbert**^1,3^

^1^University of , Germany; 

^2^Eberhard Karls University of Tübingen, Germany; ^3^Bernstein Center for Computational Neuroscience 

Berlin, Germany; ^4^Bernstein Center for Computational Neuroscience Tübingen, Germany; ^5^Max Planck Institute for Intelligent Systems, Tübingen, Germany lrothkeg@uni-potsdam.de

When scenes are presented on a computer screen, observers initially fixate close to the center - a systematic tendency known as the central fixation bias. While subsequent fixations continue to follow this tendency, the central fixation bias is most pronounced for the second fixation of the scanpath and, problematic for saliency modeling, masks attentional selection driven by top-down and bottom-up processes. Here we show that saccade target selection can be manipulated experimentally by prolonging initial fixation durations. In four scene-viewing experiments, observers were forced to maintain fixation after image onset on a specific location for a variable amount of time. The early central fixation bias was significantly reduced if the initial fixation was prolonged for 125 ms or more. Post hoc analyses showed that the central fixation bias is stronger if initial fixation durations are shorter than average. We implemented the assumption that the central fixation bias is a default mode of looking under sudden onset conditions in a dynamical model of saccade generation. Our model provides a viable computational mechanism for the interaction of the central fixation bias with bottom-up processing. In conclusion, initial fixations influence gaze patterns substantially and should not be ignored when evaluating models of eye guidance. 

**Likelihood-based Parameter Estimation and Comparison of Dynamical Eye Movement Models**

**Heiko H. Schütt**^1,2^**, Lars O. M. Rothkegel**^1^**, Hans A. Trukenbrod**^1^**, Sebastian Reich**^1^**, Felix A. Wichmann**^2^**, Ralf Engbert**^1^

^1^University of Potsdam, Germany; 

^2^University of Tübingen, Germany 

Heiko.schuett@uni-tuebingen.de

New eye-movement models are predicting full scanpaths in addition to fixation densities. Caused by strong sequential dependencies in scanpaths parameter estimation, model analysis and comparison of such models need improvement. We propose a likelihood-based approach for model analysis in a fully dynamical framework that includes time-ordered experimental data. We developed and tested our approach for the SceneWalk model (Engbert et al., 2015, J Vis). First, we show how to directly compute the likelihood function for experimental scanpaths for any model that predicts a distribution for the next fixation position given the previous fixations. Using this likelihood, we can perform Frequentist and Bayesian parameter estimation. In the Bayesian framework we obtain credible intervals indicating how well each parameter is constrained by the data. Using hierarchical models, inference is even possible for individual observers, which permits the study of individual differences. Furthermore, our likelihood approach can be used to compare different models. In our example, we show a large advantage of dynamical models exploiting dependencies between fixations compared to any fixation density prediction. Additionally, we compare different model variants using our likelihood-based evaluation. Thus the likelihood approach seems to be a promising framework for evaluating dynamical eye movement models.

**Refixation strategies for memory encoding in free viewing**

**Radha N. Meghanathan, Andrey R. Nikolaev, Cees van Leeuwen**

KU Leuven, Belgium

radhanila.meghanathan@kuleuven.be

To understand efficiency of memory encoding across saccades, we considered refixations using traditional and advanced quantification techniques. The latter involve measures based on recurrence plots, which describe the state space trajectory of dynamical systems. Participants were asked to search for 3, 4 or 5 targets for 10 seconds and remember their orientations for a subsequent change detection task, wherein, one target had its orientation changed in 50% of the cases. At the visual search stage we analysed three types of refixations, classified according to their sequence of occurrence on items, separately for targets and distractors. We also analysed recurrence measures regardless of fixations being on targets or distractors. We found that the amount of recurrences, number of repeating fixation patterns and repeated fixations on the same regions increased with the number of targets, indicating memory load. Correct change detection was associated with more refixations on targets and less on distractors, with increased amount of recurrence, and with farther distances between recurrent episodes. Thus, an optimal refixation strategy is essential for encoding and maintenance in visual working memory. 

**Modelling saccade directions with circular distributions**

**Ingmar Visser, Maartje Raijmakers, Daan van Renswoude**

University of Amsterdam, Netherlands

i.visser@uva.nl 

Eye movement patterns can be particularly relevant in participants whom are otherwise hard to test, such as young children and infants. In young infants, eye movement are one of the best sources of information that we have to gain insight into their cognitive processes. Here we study free-viewing data from infants looking at natural scenes. Free-viewing patterns are characterised by a sequence of saccades and here we are interested in modelling the distributions of saccades. Saccades are naturally modelled using circular distributions and here we use mixtures of Von Mises distributions to accurately describe the saccade distributions. An interesting result is that infants, like adults, have a bias for showing more horizontal than vertical or oblique saccades. The mixture distribution approach clearly identifies this bias. Moreover, it quantifies the variance of the saccade distributions accurately and shows a developmental trend towards more accurate eye movements. Modelling saccade directions can provide useful information about cognitive processing by giving an accurate description of the full distribution of these directions and by linking the distribution parameters to age and other individual differences measures.

**Considering, than restricting eye movement characteristics in Fixation Related Potentials: an application of the rERP framework**

**Tim Cornelissen**^1^**, Jona Sassenhagen**^2^**, Dejan Draschkow**^1^**, Melissa Vo**^1^

^1^Scene Grammar Lab, Goethe University Frankfurt, Germany;

^2^FiebachLab, Goethe University Frankfurt, Germany cornelissen@psych.uni-frankfurt.de

When interpreting the relationship between EEG and eye movement data, a researcher faces numerous possible confounds: e.g., temporally overlapping neural responses, the lack of a neutral baseline period due to this overlap, different neural responses related to eye movement parameters such as saccade amplitude, but also the possibility of (remaining) artifacts caused by eyeball rotation. Typically, researchers have avoided these confounds by constraining eye movements in their experiments, e.g. by instruction, stimulus design, or by limiting analysis to similar subsets of eye movements. However, to truly capture the relationship between eye movements and neural activity, it is suboptimal to influence or diminish eye movement effects between conditions before evaluating EEG data. Here, we present a way to address these confounds by applying regression-based estimation of evoked responses (rERPs, Smith & Kutas, 2015a; 2015b). As a proof of concept, we show very similar P300 effects in data recorded in different paradigms with 1) no eye movements, 2) restricted eye movements, and 3) a dual-target visual search task in which participants could freely move their eyes. These rERPs are robust to neural response-overlap and eye movement differences, opening up new venues for investigating neural correlates of visual processing in ecologically valid contexts. 

Tuesday, August 22^nd^, 13.30 - 15.30 

Symposium: Eye movements during the reading of narrative and poetic text

**Room1** (HS 14 - M.10.12)

**Weary with toil, I haste me to my bed: Eye tracking Shakespeare sonnets**

**Shuwei Xue**^1^**, Daniela Giordano**^2^**, Jana Lüdtke**^1^**, Renata Gambino**^2^**, Grazia Pulvirenti**^2^**, Concetto **

**Spampinato**^2^**, Arthur M. Jacobs**^1^

^1^Freie Universität Berlin, Germany; 

^2^University of Catania, Italy shuweixue@gmail.com 

The aesthetically valuable and popular Shakespeare’s sonnets have been the object of countless essays by literary critics and of theoretical but not empirical scientific studies. A fully interdisciplinary team of researchers investigate the reception of a set of sonnets using eye tracking in combination with both qualitative and quantitative narrative/poetic analyses in a model-guided, multilevel, multimethod approach (Jacobs et al., 2016, 2017). We tested to what extent specific eye movement parameters reflect both surface and deep structural as well as back- and foregrounding text features, as exemplified in the 4x4 matrix for neurocognitive poetics studies (Jacobs, 2015). The results from two labs (Catania, Berlin) using the same stimuli, but different subjects (native English speakers) and eye tracking technology provide first eye tracking evidence for the multilevel hypothesis of the Neurocognitive Poetics Model of Literary Reading (NCPM, Jacobs, 2015) submitting that textual foregrounded features detected at four relevant levels of analysis (supralexical, interlexical, lexical, sublexical) differentially affect poetry reception at all three levels of measurement (experiential, peripheral-physiological, behavioral; cf. Jacobs et al. 2016). The results are discussed in comparison to recent eye tracking studies on haiku poems and short narratives (Mueller et al., 2017; van den Hoven et al., 2016). 

**Individual differences in eye-movement patterns in response to literary language**

**Emiel den Hoven**^1,2^**, Franziska Hartung**^2,3^**, Michael Burke**^4^**, Roel Willems**^2,5,6^

^1^Freiburg University, Germany; 

^2^Max Planck Institute for Psycholinguistics, Netherlands; 

^3^University of Pennsylvania, United States of America; 

^4^University College Roosevelt, Netherlands; 

^5^Radboud University, Netherlands; 

^6^Donders Institute for Brain, Cognition and Behaviour, Netherlands emiel.vandenhoven@frequenz.uni-freiburg.de 

In the early 20th century, a group of literature scholars known as the Russian Formalists claimed that stylistically salient (literary) language use increases processing demands, and therefore causes slower reading. We tested this claim by having participants read short literary stories while measuring their eye movements. Our results confirmed that readers indeed showed longer gaze durations and made more regressions towards more literary passages as compared to less literary passages. A closer look, however, revealed significant individual differences in the effect of literariness on eye movements. Some readers in fact did not slow down at all when reading stylistically salient passages. The per-subject slowing down effect for literariness correlated with a per-subject slowing down effect for words that were statistically unexpected given the sentence context (high perplexity words): those readers who slowed down more during literary passages also slowed down more during high perplexity words, even though no correlation between literariness and perplexity existed in the stories. Moreover, readers who slowed down more during literary passages also displayed smaller saccades during these passages than those who slowed down less. We interpret these results with reference to a distinction between more conservative and more proactive, risky readers. 

**Exploring meaning construction in readers of English-language Haiku: An eyetracking study**

**Franziska Günther**^1^**, Hermann J. Müller**^1^**, Thomas Geyer**^1^**, Jim Kacian**^2^**, Stella Pierides**^2^

^1^Ludwig-Maximilians-University Munich (LMU), Germany; 

^2^The Haiku Foundation, Winchester, VA, United States of America franziska.guenther@anglistik.uni-muenchen.de 

The present study – by poets and cognitive scientists – investigated the construction of meaning when reading normative, 3-line English-language haiku (ELH; Müller et al., JEMR, 10(1), 2017). A central design feature of ELH is the presence of a cut (either after line 1 or 2) and the consequent juxtaposition of two images, which relate to each other in terms of either a context–action or a conceptually more abstract association (context–action vs. juxtaposition haiku). Understanding such haiku requires readers to resolve the tension between the two parts of the poem, i.e., to integrate the two parts (images) into a coherent 'meaning Gestalt'. To examine this process, we recorded readers' eye movements. The results indicate that processes of meaning construction are reflected in patterns of eye movements during reading (1st-pass) and re-reading (2nd- and 3rd-pass). From those, the position of the cut (after line 1 vs. line 2) and, to some extent, the type of haiku (context–action vs. juxtaposition) can be 'recovered'. Moreover, results from a recognition memory test indicate that actually resolving the haiku's meaning plays a role for later, explicit memory retrieval. These findings suggest haiku as an apt material for studying processes of meaning construction during poetry reading. 

**Immersion, Emotion and Eye Movements in Self-paced Reading of passages from Harry Potter**

**Lea Musiolek, Jana Lüdtke, Arthur M. Jacobs**

Freie Berlin, Germany leamusiolek@gmail.com 

Immersion in narrative reading and its related constructs have lately attracted increased research (e.g., Hsu et al., 2014; Jacobs & Lüdtke, 2017; van den Hoven et al., 2016). However, so far only a single published study has used eye movements as a possible indicator of immersive reading behaviour. If coherent patterns can be found, this method will prove a valuable asset to ‚objectively’ studying immersive reading and testing/constraining the Neurocognitive Poetics Model of Literary Reading (NCPM, Jacobs, 2015). In this experiment, we manipulated the emotionality of potentially immersive text excerpts from the Harry Potter series across three conditions to study affective effects on various eye movement parameters and ratings. Emotional (i.e., “happy” and “fearful” texts) were more immersive than neutral ones, with happy texts scoring significantly higher than fearful ones only on immersion – sympathy. Immersion generally correlated with faster reading, and fewer but longer fixations. The results are discussed in the light of the NCPM and those of recent related studies, in particular van den Hoven et al.’s (2016) and Mueller et al.’s (2017). 

**Using eye movements to study comprehension monitoring in beginning readers**

**Young-Suk Kim**^1^**, Christian Vorstius**^2^**, Ralph Radach**^2^

^1^University of California, Irvine, United States of America; 

^2^Bergische Universität Wuppertal kim.youngster@gmail.com 

Despite a surge in developmental research we still know little about the dynamics of reading in young children. The present work is focused on comprehension monitoring, a process of tracking and verifying the understanding of written text. More specifically, we sought to determine to what extent beginning readers are sensitive to semantic inconsistencies in short stories and what explains interindividual variation. We examined whether monitoring makes a unique contribution to comprehension over and above word reading and listening comprehension. Second graders from schools in Northern Florida (N=319) were asked to read four line passages that could contain an implausible statement in the last line. A battery of psychometric assessments for component skills of reading was also administered. Results indicate that participants spend considerably greater time fixating inconsistent words and engaged in frequent lookbacks to previous lines of text in search of useful information. These oculomotor manifestations of comprehension monitoring were explained by both word reading and listening comprehension. Although these component skills explained the vast majority of variance in reading comprehension, comprehension monitoring added unique explanatory power. These results contribute to a better understanding of skills underlying comprehension monitoring and its unique role in reading comprehension for beginning readers. 

Thematic Session: Visual search

**Room 2** (HS 32 - K.11.23)

**Your Attention seeks Confirmation: Visual confirmation bias overshadows prevalence effects in visual attention**

**Stephen C. Walenchok**^1^**, Stephen D. Goldinger**^1^**, Michael C. Hout**^2^

^1^Arizona State University, United States of America; 

^2^New Mexico State University, United States of America swalench@asu.edu

Rajsic, Wilson, and Pratt (2015; 2017) recently discovered a visual form of confirmation bias. People searched for a colored target letter in circular displays (e.g. a p among b’s, d’s, and q’s). Crucially, only one color was explicitly cued prior to search (e.g., “Press < z > if the p is [green], press < m > if the p is another color”). In each display, 25%, 50%, or 75% of letters matched the cue color; the remaining were in the uncued color. Optimally, one should restrict search to the minority subset, using inference if necessary. Yet, people preferentially scoured cue-colored letters, even when they comprised the majority, entailing more laborious search. We tested whether this confirmatory bias is exaggerated or mitigated through prevalence learning. Targets either occurred frequently (high prevalence) or rarely (low prevalence) in the cued color. Participants disproportionately inspected cue-colored letters, even when cue-matching targets were exceedingly rare. Additionally, results suggest slower perceptual decisions when confirming (i.e., recognizing and responding to) the rare target. These results suggest that visual confirmation bias arises in attentional guidance and that prevalence effects arise due to differences in perceptual readiness to identify common and rare objects.

**Humans do not make efficient eye movements during visual search**

**Anna Nowakowska**^1^**, Alasdair D. F. Clarke**^1,2^**, Amelia R. Hunt**^1^

^1^University of Aberdeen, United Kingdom; 

^2^University of Essex, United Kingdom

a.nowakowska@abdn.ac.uk 

When searching for an object, to what extent do people move their eyes efficiently? Here we tested whether eye movements are directed to locations that yield the most information, by splitting search arrays into two halves vertically, with homogeneous distractors on one half and heterogeneous distractors on the other. When a target is present on the homogenous side, it can be easily detected using peripheral vision, so observers should only make fixations on the heterogeneous side. However, we find that most participants over-fixate the homogeneous half, at a substantial cost to reaction time. We also find that search in split-screen arrays was slower than predicted based on performance in uniform displays. This suggests a failure to distribute fixations optimally across the two types of search arrays contributed to search inefficiency, in addition to a more general tendency to make unnecessary confirmatory fixations. Finally, we introduced restrictions on viewing time, and found that participants could achieve a similar level of search accuracy with far fewer eye movements. The results demonstrate that we make a large number of inefficient eye movements, suggesting estimates of expected information gain contribute very little to fixation target selection during search.

**Time course of brain activity during unrestricted visual search: Co-registering EEG and Eye Movements**

**Juan E. Kamienkowski**^1,2^**, Alexander Varatharajah**^3^**, Mariano Sigman**^4^**, Rodrigo Q. Quiroga**^3^**, Matias J. Ison**^5^

^1^Laboratory of Applied Artificial Intelligence, CONICET-UBA, Argentina;

^2^Physics Department, FCEyN-UBA, Argentina;

^3^Centre for Systems Neuroscience and Department of Engineering, University of Leicester, United Kingdom;

^4^University Torcuato Di Tella, Argentina;

^5^School of Psychology, University of Nottingham, United Kingdom juank@dc.uba.ar 

When looking for a friend in this conference, we have to inspect several faces before achieving our goal. The processing of visual stimuli within each fixation has been explored in recent EEG and eye movements co-registration experiments (fixation-Related Potentials; fERPs). However, how these individual responses are embedded in more complex behavior has been left relatively aside. Previous experiments with artificial foveated stimuli have shown gradual changes associated with the accumulation of information or expectation, either in the amplitude or the spectral profiles of the EEG signal. This study investigates the brain dynamics throughout the sequence of fixations when searching for a target face within natural images of crowds. FERPs showed target detection effects consistent with previous works. Moreover, the target-related component was significantly modulated by the trial length. The global dynamics of distractor processing within a trial showed gradual changes of fERP’s baseline amplitude, as well as major changes in the spectral profile -particularly in the theta and alpha bands-. This can be interpreted in terms of a growing engagement in the search, with changes in expectation and anticipation. Thus, while fixation-related components account for local processing, baseline activity and oscillations provide information about the global progression of the task. 

**Visual working-memory biases attention: Evidence for involuntarily objectbased top-down control by search-irrelevant features**

**Rebecca M. Foerster**^1,2^**, Werner X. Schneider**^1,2^

^1^Neuro-cognitive Psychology, Bielefeld University, Germany; 

^2^Cognitive Interaction Technology’ Cluster of Excellence CITEC, Bielefeld University, Germany rebecca.foerster@uni-bielefeld.de 

Attentional biasing in visual search for varying targets requires that a target template is stored in visual working memory (VWM). It is unknown whether all or only search-relevant features of a VWM template bias attention during search. Bias signals might be configured to favor task-relevant features so that only search-relevant features bias attention. Alternatively, VWM might maintain objects in the form of bound features rather than individual features. Therefore, all features might bias attention objectbased. We investigated in three experiments which of these two options of attentional biasing by VWM might be valid. A colored cue depicted the target prior to each search. Participants saccaded to the target predefined by its identity opposite a distractor. Saccades went more often and faster to the target when it matched the cue not only in its target-defining identity but also in the irrelevant color. In the third experiment, cue-colored distractors captured the eyes more often than different-colored distractors, even if cue and target were never colored the same. Because participants were informed about the misleading color, this result argues against a strategical and voluntary usage of color. Instead, search-irrelevant template features biased attention obligatorily arguing for involuntary top-down control by object-based VWM templates.

**Eye Movements and the Label Feedback Effect: Speaking Modulates Visual Search, But Probably Not Visual Perception** **Katherine P. Hebert, Stephen C. Walenchok, Stephen D. Goldinger**

Arizona State University, United States of America kpjones7@asu.edu 

The -feedback hypothesis (Lupyan, 2007) proposes that language can modulate low- and high-level visual processing, such as “priming” the perception of visual objects. Lupyan and Swingley (2012) found that repeating target names facilitates visual search (shorter RTs and higher accuracy). However, design limitations made their results challenging to assess and left key questions unanswered, including whether speaking the name simply serves as a task reminder, or whether the verbal process actually modulates visual processing. In this study, we evaluated whether self-directed speech influences target locating (i.e. attentional guidance) or target identification after location (i.e. decision time), testing whether the Label Feedback Effect reflects changes in visual attention or some other mechanism (e.g. template maintenance in working memory). Across three experiments, we analyzed search RTs and eye movements from four within-subject conditions. People spoke target names, nonwords, irrelevant (absent) object names, or irrelevant (present) object names. Speaking target names weakly facilitated visual search, but speaking different names strongly inhibited search. The most parsimonious account is that language affects target template maintenance during search, rather than visual perception.

**Shorter fixation durations in visual search after 24 hours of total sleep deprivation** **Christian Mühl, Daniel Aeschbach**

Institute of Aerospace Medicine, German Aerospace Center (DLR), Germany

Christian.Muehl@dlr.de 

Visual attention is a relevant construct in the domain of aviation, since information for operators is often presented visually. Shift and night work schedules in the aviation industry require an understanding of the susceptibility of visual attention to sleep deprivation (SD). To study the effect of SD on visual attention, we employed two well-established serial search paradigms and analyzed average fixation duration and saccade velocity. We expected SD-induced cognitive slowing to be accompanied by longer fixation durations and slower saccades. We measured search performance and the related oculomotor characteristics before and after 24 hours of SD in 24 subjects, as well as in a well-rested control group. Search performance deteriorated significantly compared to the control group in terms of speed and accuracy. Saccade velocity decreased strongly. Contrary to our expectation, we found a decrease in fixation duration under SD, while the number of fixations did not change significantly. The results show only a partial slowing of oculomotor characteristics in visual search after SD. The decrease of mean fixation duration, however, might indicate a propensity for faster and riskier decision-making, manifesting itself on the level of single fixations. Known as “speed-error trade-off”, such behavior reflects problems to compensate SD-impaired cognitive processes

Thematic Session: Interactive and group eye-tracking

**Room 3** (HS 28 - I.13.71) 

**Group Eye Tracking (GET) Applications in Gaming and Decision Making**

**Cengiz Acarturk, Mani Tajaddini, Ozkan Kilic**

Middle East Technical University, Turkey acarturk@gmail.com 

Eye tracking has been employed as an experimental methodology for individual session recording since the past several decades. It has also been employed as a human computer interaction method for individuals more recently. Multi-user eye tracking is a novel paradigm that has been broadening the scope of both experimental methodology and human computer interaction towards social cognition and interaction. GET (Group Eye Tracking) is a multi-user eye tracking environment for simultaneous recording of eye movement data from multiple eye trackers. We have been employing the GET platform for conducting experiments in two domains: multi-user gaze gaming and group decision making. We will present two studies that have been conducted by using the GET paradigm. The first is a three-player game, where the participants played the game under competitive and collaborative conditions. The second study focuses on a decision-making task, where the participants make risky or conservative monetary choices. Our goal is to share the findings, present the challenges in current research, and discuss the future of multi-user eye tracking paradigms.

**Mass measurement of eye-movements under the dome - proof of concept studies**

**Maksymilian Bielecki**^1^**, Katarzyna Potęga vel Żabik**^1,2^**, Michał Gochna**^2^**, Jacek Mikulski**^2^

^1^SWPS University of Social Sciences and Humanities, Poland; 

^2^Copernicus Science Centre, Poland mbielecki@swps.edu.pl 

Mass measurement of fixation behavior is a challenging task. Richard Shillcock and Cara Wase proposed in their study presented during ECEM in 2015 a novel way allowing to capture eye movements in a large group of students watching a video-recorded lecture. In this study, the lecture recording was interrupted systematically by a grid of dark-on-light letters-and-digits presented across the screen. Participants wrote down the letters-and-digits they saw at that particular moment revealing the fixation locations. We tested the feasibility of using a similar methodology to record gaze locations during planetarium presentations. Two studies were conducted during live presentations under the dome of Copernicus Science Centre in Warsaw (semi-spherical screen with the diameter of 16 meters). Our results prove that proposed methodology allows creating heat maps qualitatively resembling those obtained by using conventional eye-tracking devices. The visual attention of the viewers could be effectively captured revealing not only effects related to the presenter’s narrative but also the effects of participants’ characteristics and seat positions.

**Using gaze trackers and combining the results**

**Miika Tapio Toivanen, Markku Hannula**

University of Helsinki, Finland miika.toivanen@helsinki.fi 

With mobile gaze tracking, the visual attention of a subject can be measured in natural environments. Measuring many subjects simultaneously allows to assess correlations of their gaze patterns which opens new possibilities in studying attentional behavior on a group level. However, the expensiveness of reliable mobile gaze trackers makes this economically challenging or infeasible. An additional problem is combining the gaze points in space and time to infer, e.g., if the subjects have fixated the same target at the same time. We use self-made gaze tracking glasses and advanced algorithms, allowing to conduct group measurements with a reasonable cost. We use visual markers near the expected gaze target locations to map each person's gaze point to same coordinate system and also to alleviate the automatic gaze target classification. The timestamps of the captured video frames are saved on the recording computers which are first synchronized with the universal time. As a result, we have each person's gaze behavior on a same time axis and the gaze targets are automatically classified. We apply the methodology on an educational study and show how four students' and teacher's gaze points are combined, having high temporal and spatial accuracy. 

**Joint Attention on the Cartesian Plain: A Dual Eye-Tracking Study**

**Anna Shvarts, Anatoly Krichevets**

Lomonosov Moscow State University, Russian Federation shvarts.anna@gmail.com 

Joint attention is considered to be a crucial mechanism of language acquisition in early childhood. We suppose that this mechanism plays a significant role in mathematics acquisition as well. In a qualitative study we used dual eye-tracking to describe dynamics of visual joint attention while adults were teaching the first grade students (7 years old) to approach coordinates of points on the Cartesian plane. The dual eye-tracking technical solution allowed a teacher and a student to sit together in front of the same monitor and to involve gestures into their communication; the videos of the overlapping gaze paths from the pairs of the participants were synchronized with the records of their gestures and verbal explanations. Frame by frame analysis revealed that students did not follow the teachers’ guidance, but anticipated it or actively sought how a gesture, an explanation and the diagram corresponded to each other in order to make their perception meaningful. We distinguished two ways of how the joint attention was achieved: a teacher might guide the student or follow the student’s mistaken strategy to adjust the explanations accordingly. Dual eye-tracking appeared to be a promising instrument for the analysis of teaching-learning shared activity. Supported by RFBR, 15-06-06319.

**How Teachers See It: Using Mobile Eyetracking to Explore Professional Vision and Teacher-Student Interactions in the Classroom**

**Irene T. Skuballa, Antje von Suchodoletz**

NYUAD, United Arab Emirates its101@nyu.edu 

Teacher professional vision is the ability to notice and interpret significant features of classroom interactions. It determines how a teacher perceives classroom events and makes instructional decisions in an authentic and demanding environment. Yet, teacher professional vision does not always translate into effective instruction. We explored teachers’ visual attention during classroom interactions and linked teachers’ eye gaze to key events in classroom interactions. We recorded visual attention of 46 kindergarten teachers using mobile eye-tracking glasses. The quality of instruction was assessed with the Classroom Assessment Scoring System (CLASS Pre-K). Results indicated a large variation in teachers’ distribution of visual attention; some teachers focused on only few children, others managed to look at every child for equally long. In general, teachers allocated significantly more visual attention to children than to materials. Visual shifts were positively related to more visual attention on children. In addition, we found a positive association between the number of visual shifts between children and instructional support as measured by the CLASS Pre-K: Higher instructional support correlated with more attentional shifts between children. Together the findings speak to the applicability of mobile eye tracking glasses in naturalistic classroom settings. Implications for teacher education and training will be discussed. 

**Gaze-assisted remote communication between teacher and students**

**Kari-Jouko Räihä**^1^**, Oleg Spakov**^1^**, Howell Istance**^1^**, Diederick C. Niehorster**^2^

^1^University of Tampere, Finland; 

^2^Lund University, Sweden kari-jouko.raiha@uta.fi 

Fluent communication is essential for interaction between teacher and students, and has a significant impact on the learning experience in the classroom. Communication is facilitated by means (like chat boxes, audio channel, or video feed) that do not reveal the point of attention of the student. In other contexts, gaze been found useful to establish joint attention in peer-to-peer communication. We expand on that by facilitating transmission of gaze points between several networked participants. In our setup, students are able to see the teacher's desktop in a window on their own desktop, and the teacher’s active point of gaze superimposed over it. The teacher, in turn, has an additional monitor that shows the desktops of each student, again with their gaze points superimposed. We have run a pilot study with four students and the teacher in a separate room. The screen video was transmitted using the VNC tool, and gaze data was transmitted via in-house software. The teacher and students were also connected by Skype chat; students mostly listened to the teacher (by earphones), but if someone raised a question, it was heard by all participants. We will present the implementation issues and share the experiences from the pilot study.

Thematic Session: Scene perception

**Room 4** (HS 26 - I.13.65)

**The relative importance of foveal vision in visual search in 3D dynamic scenes** **Adam C. Clayden, Robert B. Fisher, Antje Nuthmann**

University of Edinburgh, United Kingdom s1475487@sms.ed.ac.uk 

Search performance when finding targets within static naturalistic-scenes has been demonstrated to be similar with and without foveal vision (Nuthmann, 2014). However, detection of objects during selfmotion has been shown to rely on optical flow (Warren & Rushton, 2008). Participants in our study were required to search for context free targets within 3D, simulated self-motion scenes, made with the Unity Game Engine. As the targets were non-moving, they conformed with the optical flow’s movement, and so blended with the flow field. We investigated: 1) Does the inclusion of optical flow produce costs to search performance with foveal vision loss? 2) If there is a cost, can we improve search performance by manipulating optic flow trajectory and target placement? Results show that 1) localising targets while moving without foveal vision produces a significant cost to performance. 2) Manipulating the path of self-motion and varying target location did not eliminate the cost of foveal impairment. As target motion blended with the flow field, observers needed to fixate near the target for detection, utilising their high acuity vision. We suggest that the optical flow interfered with the target localisation process, which results in an increased reliance on high acuity vision. 

**The trajectory of eye movements to object-scene inconsistencies and their relation to language abilities**

**Sabine Öhlschläger**^1,2^**, Melissa Le-Hoa Vo**^1,2^

^1^Scene Grammar Lab, Goethe University, Frankfurt, Germany; 

^2^Center for Research on Individual Development and Adaptive Education of Children at Risk (IDeA),

Frankfurt, Germany

oehlschlaeger@psych.uni-frankfurt.de

Most of you would be surprised to find a toaster in your bedroom, because it is not what you’d expect in this room. You would also be surprised to find the toaster in the kitchen sink, because it is not where we would expect it to be. Our knowledge about what (semantics) to expect where (syntax) in our world is probably not present from birth. When does this knowledge develop and how does it relate to other meaningful concepts, e.g. in language? To answer these questions we recorded eye movements of two- to four-year old children (n=72) while they were viewing photographs of daily-life scenes with inconsistent semantics or syntax in a paradigm with gaze-contingent stimulus presentation. We found that the difference in first-pass dwell times between inconsistent and consistent conditions increased with age. Concordantly, only 4-year old children showed a positive relation of this eye movement effect with semantic language abilities in categorizing objects (e.g. animals). Here we linked eye movement control to age and language abilities. Interestingly, these relations did not differ between semantic and syntactic inconsistencies, which will be discussed in terms of the sensitivity of the cognitive representations during development and of our eye movement measure.

**Dynamic recipes for oculomotor selection of objects in realistic scenes**

**Sara Spotorno**^1^**, Ben Tatler**^2^

^1^University of Glasgow, United Kingdom; 

^2^University of Aberdeen, United Kingdom sara.spotorno@glasgow.ac.uk

We examined the extent to which semantic informativeness, consistency with expectations and perceptual salience contribute to object prioritisation in scene viewing and representation. In scene viewing (Experiments 1-2), semantic guidance overshadowed perceptual guidance in determining fixation order, with the greatest prioritisation for objects that were diagnostic of the scene’s depicted event; there was some advantage for inconsistent objects, but only relative to consistent but marginally informative objects. Perceptual properties, on the other hand, affected selection of consistent but not of inconsistent objects. Semantic and perceptual properties also interacted in influencing foveal inspection, as inconsistent objects were fixated longer than marginal objects and than low but not high salience diagnostic objects. In change detection (Experiment 3), perceptual guidance overrode semantic guidance, promoting detection of highly salient changes. A residual advantage due to diagnosticity emerged only when selection prioritisation could not be based on low-level features. Overall these findings show that semantic inconsistency is not prioritised within a scene when competing with other relevant information that is essential to scene understanding and respects observers’ expectations. Moreover, they reveal that the relative dominance of semantic or perceptual properties during selection depends on ongoing task requirements. 

**Individual Pursuit Strategies in Dynamic Natural Scene Perception**

**Ioannis Agtzidis, Mikhail Startsev, Michael Dorr**

Technical University of Munich, Germany ioannis.agtzidis@tum.de 

In the presence of dynamic stimuli, observers often perform smooth pursuit (SP) eye movements. Because their characteristics such as speed and durativ are more variable than the more stereotypical fixations and saccades, large-scale analysis of SP properties remains challenging in the absence of highly robust automated classification algorithms. To improve our understanding of SPs when viewing dynamic natural scenes, we manually labelled a large ground truth data set. Three humans annotatedsaccades, fixations, and SPs in the GazeCom dataset, which comprises more than four hours of gaze recordings for naturalistic videos. SP episodes lasted up to 4300ms (median duration 320ms). Overall, SP represented about 11% of the total viewing time. Individually, there was substantial variation between videos: from 0% (videos with very little object motion) up to 23.2% (videos with continuously moving big objects). Subjects also showed great differences with SP rates from 3.7% to 18.4%. These differences likely can be attributed to different tracking techniques for moving objects (many short fixations with small saccades inbetween vs smooth tracking) as well as intrinsic differences in the top-down deployment of attention: while motion onsets widely capture attention and lead to SP initiation, observers differ in how long they sustain SP.

**The bimodality of saccade duration distribution**

**Hélène Devillez, Randall C. O’Reilly, Tim Curran**

University of Boulder, United States of America helene.devillez@colorado.edu

It is well known that there is a positive correlation between saccade amplitude and saccade duration (van Beers, 2007). If saccade amplitude shows an exponential distribution, the distribution of saccade duration is known to be bimodal. This study aims at investigating the saccade duration distribution and discussing its relation with the two modes of viewing during the exploration of scenes. The ambient mode is characterized by large amplitude saccades and short duration fixations, and the focal mode shows shorter saccades and longer fixations (Unema et al 2005). Mode classification relies on the previous saccade amplitudes (Follet et al 2011). We used data from 28 participants freely exploring natural scenes in an object memorization task. Data analysis showed that the bimodality of saccade duration distribution was not present when taking into account only saccades with a small amplitude (< 5°). We clustered fixations according to the duration of the previous saccade. Short duration saccades showed slow speed compared to longer saccades, reminiscent of smooth pursuit eye movements. Interestingly, short duration saccades were preceded by longer and followed by shorter fixations than long saccades, suggesting that the bimodality of the saccade duration distribution is not related to focal/ambient mode. 

**Using sound to guide gaze in a ‘split-screen’ film: Mike Figgis’ Timecode as a found experiment.**

**160, Jonathan P. Batten, Jennifer X. Haensel** Birkbeck, University of London, United Kingdom tj.smith@bbk.ac.uk 

Viewing a dynamic audiovisual scene has inherent challenges for where and when gaze is allocated. Film sound designers believe they have techniques for simplifying this task and guiding viewer attention by modulating the relative audiovisual salience of objects in a scene. However, empirical evidence that such audio manipulations causally influence gaze in dynamic scenes is limited. This study utilised a found experiment, Mike Figgis’s experimental feature film, Timecode (2000) which contains four continuous interrelated perspectives displayed using a 2x2 split-screen, where each quadrant has an isolatable sound mix. We investigated the influence of sound on free-viewing by manipulating the presence of sound across the four quadrants one at a time separated by abrupt sound cuts. Sound presence significantly increased the proportion of gaze to that quadrant but only after the viewer had learnt the audiovisual pairings. Fixation durations to sound regions were significantly longer than those to visual only quadrants. Computational audiovisual salience values are also considered as predictors of gaze between the quadrants. These results confirm Figgis’ belief that he could influence gaze via 

sound design by manipulating entire audio scenes but it is not currently known whether sound has a similar attentional cuing effect in natural scene viewing. 

## Wednesday, 23^rd^, 10.30 - 12.30 

Symposium: The role of eye movements in self-motion perception

**Room1** (HS 14 - M.10.12) 

**Gaze and the visual control of foot placement when walking over real-world rough terrain**

**Jonathan S. Matthis, Mary M. Hayhoe**

University of Texas at Austin, United States of America matthis@utexas.edu 

Walking over rough terrain requires walkers to perform a rapid visual search on the upcoming path to identify stable footholds to ensure safe and stable locomotion. During this behavior, the saccadic eye movements that gather the information necessary for foot placement must occur concurrently with stabilizing reflexes that counteract the characteristic acceleration patterns of the head during locomotion. Using a novel experimental apparatus, we recorded the eye movements and full-body kinematics of subjects walking over three levels of real-world rough terrain – extremely rocky dry creek beds (Rough), moderately rocky trails (Medium), and flat packed-earth trails (Flat). In the Rough and Medium terrains gaze was tightly coupled to the locations of upcoming footholds, with terrain-specific differences in the temporal correlation between gaze and the first, second, and third upcoming steps. In contrast, in the Flat terrain, subjects did not fixate upcoming footholds, but still made occasional fixations on the upcoming path that had a similar temporal look ahead to the patterns of fixations made in the Rough and Medium terrains. In short, subjects showed distinct patterns of gaze behavior that were shaped by the specific task demands inherent to locomotion over the different types of rough terrain.

**Eye movement cues to self-motion perception**

**Pieter W. Medendorp**^1^**, Ivar A. H. Clemens**^1^**, Luc P. J. Selen**^1^**, Antonella Pomante**^1^**, Paul R. MacNeiglage**^2^

^1^Radboud University, Netherlands; 

^2^University of Nevada, United States of America

p.medendorp@donders.ru.nl

Self-motion is typically accompanied by eye movements to maintain gaze on objects of interest. We studied whether these fixation eye movements provide a cue for self-motion perception. Using a two alternative forced choice (2-AFC) task, participants indicated whether the second of two successive passive lateral whole-body translations was longer or shorter than the first. Eye movements were constrained by presenting either a world-fixed or body-fixed fixation point (at two different distances) or no fixation point at all (allowing free gaze) during the motion. Perceived translations were shorter with a body-fixed than world-fixed fixation point, suggesting that eye movement signals contribute to self-motion. Furthermore, perceived translation was smaller when fixating a far compared to a nearby world-centered target, indicating that eye movements are not properly scaled in self-motion perception. Finally, when gaze was free during both translation intervals, the interval with the larger eye movement excursion was judged to be larger more often than chance. We conclude that eye movements provide a rudimentary cue to self-motion, even in the absence of visual stimulation, with a compensation for fixation depth that is partial at best.

**Visual-vestibular conflict detection depends on fixation**

**Isabelle T. Garzorz**^1,2^**, Paul MacNeilage**^1,2,3^

^1^LMU -University of Munich, Germany; 

^2^Graduate School of Systemic Neurosciences, Munich; 

^3^Department of Psychology, Cognitive and Brain Sciences, University of Nevada, Reno isabelle.garzorz@lrz.uni-muenchen.de 

Visual and vestibular signals are the primary sources of sensory information for self-motion. Conflict among these signals can be seriously debilitating, resulting in vertigo, inappropriate postural responses, and motion sickness. Despite this significance, the mechanisms mediating conflict detection are poorly understood. Here we model conflict detection simply as cross-modal discrimination with benchmark performance limited by variabilities on the signals being compared. In a series of psychophysical experiments conducted in a virtual reality motion simulator, we measure these variabilities and assess conflict detection relative to this benchmark as well as visual-vestibular integration performance. We specifically examine the impact of eye movements on these behaviors and observe that there is a tradeoff between integration and conflict detection that is mediated by eye movements. Minimizing eye movements by fixating a head-fixed target leads to optimal integration but highly impaired conflict detection. Minimizing retinal motion by fixating a scene-fixed target improves conflict detection at the cost of impaired integration performance. The common tendency to fixate scene-fixed targets during self-motion may indicate that conflict detection is typically a higher priority than the small increase in precision of self-motion estimation that is obtained through integration.

**Heading representations in primates are compressed by saccades**

**Frank Bremmer**^1^**, Jan Churan**^1^**, Markus Lappe**^2^

^1^Philipps-Universität Marburg, Germany; 

^2^Westfälische -Universität Münster, Germany frank.bremmer@physik.uni-marburg.de 

Perceptual illusions help to understand how sensory signals are decoded in the brain. Here we asked if also the opposite approach is applicable, i.e., if results from decoding neural activity from monkey extrastriate visual cortex could correctly predict a hitherto unknown perceptual illusion in humans. We recorded neural activity from macaque areas MST and VIP during continuous presentation of selfmotion stimuli and concurrent reflexive eye movements. Stimuli simulated self-motion across a ground plane in different directions. A linear heading-decoder performed veridically during fixation and slow eye-movements. During fast eye-movements (saccades), however, the decoder erroneously reported compression of heading towards straight-ahead. Since functional equivalents of macaque areas MST and VIP have been identified in humans, we predicted a perceptual correlate (illusion) of this perisaccadic decoding error. In a second experiment, human subjects performed saccades while we presented visually simulated self-motion in different directions. As predicted, perceived heading was perisaccadically compressed. A behavioral control experiment revealed compression to be directed towards the direction of gaze rather than the head- or body-midline. Our data strongly suggest that response properties of primate areas MST and VIP are the neural substrate of this newly described visual illusion.

**Dynamics of eye movements during visual path integration**

**Dora Angelaki, Kaushik Lakshminarasimhan, Xaq Pitkow**

Baylor College of Medicine, United States of America angelaki@bcm.edu 

The ability to path integrate is well documented in humans and animals. However the behavioural algorithms supporting path integration remain unclear. We studied this in primates by training humans and macaque monkeys to use joystick to catch fireflies in a virtual environment devoid of landmarks. In order to solve this task, subjects had to update their position estimates based solely on optic flow generated by moving through virtual space. Target locations were varied randomly across trials to eliminate the use of time-based strategies. Although each target firefly only appeared briefly at the beginning of the trial, behavioural recordings of eye-movements revealed that subjects tracked the target even after it was long gone, thereby maintaining their gaze at the target location until they reached it. Across trials, variability in subjects’ eye positions mirrored their behavioural variability: subjects were more precise in tracking and reaching nearby than far away targets. Our results suggest that the output of integration might be embedded in the brain’s oculomotor circuit, such that the eye position provides a dynamic readout of one’s distance to target during visual path integration.

Thematic Session: Attention and memory

**Room 2** (HS 32 - K.11.23)

**Attentional selection in averaging saccades**

**Luca Wollenberg**^1,2^**, Heiner Deubel**^2^**, Martin Szinte**^2^

^1^Graduate School of Systemic Neurosciences, Ludwig-Maximilians-Universität München, Munich, Germany; 

^2^Allgemeine und Experimentelle Psychologie, Department Psychologie, Ludwig-Maximilians-

Universität München, Munich, Germany luca.wollenberg@gmx.de 

The premotor theory of attention postulates that spatial attention arises from the activation of saccade areas and that the deployment of attention is the consequence of motor programming. Yet, attentional and oculomotor processes have been shown to be dissociable at the neuronal level in covert attention tasks. To investigate a potential dissociation at the behavioral level, we instructed participants to saccade towards one of two nearby, competing saccade cues. The spatial distribution of visual attention was determined using oriented Gabor stimuli presented either at the cue locations, between them or at several other equidistant locations. Results demonstrate that accurate saccades towards one of the cues were associated with pre-saccadic enhancement of visual sensitivity at the respective saccade endpoint compared to the non-saccaded cue location. In contrast, averaging saccades, landing between the two cues, were not associated with attentional facilitation at the saccade endpoint, ruling out an obligatory coupling of attentional deployment to the oculomotor program. Rather, attention before averaging saccades was equally distributed to the two cued locations. Taken together, our results suggest that the oculomotor program depends on the state of attentional selection before saccade onset, and that saccade averaging arises from unresolved attentional selection.

**Vertical gaze paralysis is associated deficits of attention and memory: Evidence from ProgressiveSupranuclear Palsy**

**Daniel Smith**^1^**, Neil Archibald**^2^

^1^Durham University, United Kingdom; 

^2^The James Cook University Hosptial, South Tees NHS Foundation Trust, UK daniel.smith2@durham.ac.uk 

The that control covert attention and spatial short term memory are tightly coupled with the oculomotor system. We have previously argued for a specific link between the ability to make normal eye-movements and the optimal functioning of exogenous attentional orienting and spatial short-term memory (Pearson, Ball, & Smith, 2014; Smith, Ball, & Ellison, 2014). One key piece of evidence for this link is a selective deficit of exogenous orienting along the vertical axis in Progressive Supranuclear Palsy (PSP), a degenerative neurological disease characterised by vertical paralysis of gaze (Rafal, Posner, Friedman, Inhoff, & Bernstein, 1988). In the current work we used visual search tasks and the Corsi blocks task to test for selective, vertical deficits of covert attention and short-term memory in people with PSP. Patients had shorter memory spans and less efficient covert visual search when stimuli were presented along the vertical axis compared to the horizontal axis. Critically, this effect was not observed in age matched controls, or a group of patients with Parkinsons disease. These data suggest that an intact eye-movement system is required for optimal functioning of covert spatial attention and short-term spatial memory.

**Nasal-temporal differences on cueing effect: how cue eccentricity and visual field affect the orienting of visuo-spatial attention**

**Soazig Casteau, Daniel T. Smith**

Durham University, United Kingdom soazig.casteau@durham.ac.uk 

Premotor theory of attention argues that orienting of attention is the result of the preparation of an eye-movement. Indeed, neuropsychological investigations of patients with defective eye-movements and studies of healthy participants where the range of eye-movements are experimentally manipulated suggest that both covert spatial attention and overt eye-movements are limited to the Effective Oculomotor Range (EOMR). Here, we used the Posner cueing task to examine whether exogenous, covert attentional orienting was limited to the EOMR in neurotypical participants when the eye was in the canonical position. After determining each individual EOMR, we presented a cueing task where we manipulated the eccentricity (below vs. beyond the EOMR) of cues and targets. Overall RT’s were significantly longer in the beyond compared to the below EOMR condition. Contrary to the previous neuropsychological work, we did not observe a any interaction between EOMR and validity. However, looking at cue hemifield separately, our results showed that the cueing effect was absent when stimuli were presented beyond the EOMR, but only in the Nasal visual field. This result offers some support for the idea that exogenous orienting is limited to the EOMR, although the effect was more subtle than that observed in neuropsychological patients.

**Presaccadic attention analyzed with a novel dynamic noise paradigm**

**Nina M. Hanning**^1,2^**, Heiner Deubel**^1^

^1^Ludwig-Maximilians-Universität München, Munich, Germany;

^2^Graduate School of Systemic Neurosciences, Munich, Germany hanning.nina@gmail.com

Discrimination performance has become an important proxy for the analysis of visuospatial attention. In a typical paradigm, test stimuli such as characters or oriented Gabors are briefly presented at various locations in the visual field. One potential problem arising here is that these test stimuli themselves constitute visual objects that may structure the visual field and thus affect what they want to measure, the spatial distribution of attention. We developed a novel full-field stimulus composed of orientationfiltered dynamic pink noise that allows to test the spatio-temporal distribution of attention across the visual field, without the presence of object-like visual structures. As a remarkable property of this stimulus, we demonstrate that local discrimination performance is largely independent of visual eccentricity. This allows to directly compare attentional performance at foveal and peripheral locations. We used this stimulus to analyze the distribution of spatial attention before saccadic eye movements, and to study the effect of the presence or absence of a saccade target object. Results show that saccades are preceded by shifts of attention even if they are directed into an unstructured visual field. This deployment of attention towards the saccade landing position is accompanied by a removal of processing resources from fixation.

**Detecting concealed memory via eye movements**

**Yoni Pertzov, Oryah Lancry, Tal Nahari, Gershon Ben-Shakhar**

The Hebrew University of Jerusalem, Israel pertzov@gmail.com 

Can gaze tracking be used to reveal whether someone is familiar with another person, even when she tries to conceal this familiarity? During visual processing, gaze allocation is influenced not only by features of the visual input, but also by previous exposure to objects, resulting in idiosyncratic scanning patterns. However, the precise dynamic of gaze allocation towards personally familiar objects have not been studied in the context of revealing concealed familiarity. Here we show that when subjects try to encode several faces, gaze is inevitably attracted towards a personally familiar face, followed by a strong repulsion, even when participants were explicitly instructed to conceal their familiarity. Despite attracting overall less fixation time, familiar faces were nevertheless reported more rapidly and accurately. By exploiting these behavioral patterns, a machine learning classification algorithm detected the familiar faces at an accuracy rate exceeding 91%. These results shed new light on the temporal aspects of attention preferences and the efficient way in which existing memory representations are encoded into short term memory. It also provides a highly accurate method of detecting concealed information using eye tracking.

**Spoken words help in retreiving information from visual working memory** **Seema G. Prasad, Pratik Bhandari, Ramesh Mishra**

University of Hyderabad, India gp.seema@uohyd.ac.in 

Visual attention and working memory involve overlapping mechanisms. Retrieving information from visual working memory involves directing visual attention to the location of the information to be retrieved (Theeuwes et al., 2010). Additionally, spoken words have been shown to mediate visual attention to objects that are related phonologically or semantically (Tanenhaus et al., 1995). We examined if spoken word mediated attention helps in retrieving objects from visual working memory. Participants were asked to remember two or four objects. After 3000 ms, participants saw one of the objects and had to judge if its location was same or different compared to the previous display. During the retention interval of 3000 ms, a blank screen was presented along with a spoken word which was a phonological competitor of the object to be remembered (experimental trials). On control trials, no spoken word was presented. Results showed that accuracy on the memory task was higher on experimental trials compared to the control trials. More importantly, the proportion of fixations to the location of the object to be remembered was higher compared to the unrelated objects, only on experimental trials. These findings show that language input can bias visual attention and facilitate in working memory maintenance.

Thematic Session: Innovative methods and technology

**Room 3** (HS 28 - I.13.71)

**Improving computerized adaptive testing using eye tracking measures**

**Benedict C. O. F. Fehringer**

University of Mannheim, Germany

b.fehringer@uni-mannheim.de

Computerized adaptive tests (CATs) adapt the testing procedure to the participants’ ability based on accuracy and reaction times in item subsets. However, the information amount of easy and difficult items is limited because of ceiling or floor effects. Eye tracking measures are promising to gain more information from these items. Gaze movements can show how participants solve an item. The goal of this study was to analyze the potential of eye tracking measures for more efficient adaptive testing. To this end, N = 81 participants conducted a test for spatial thinking. In each task, the participants had to decide whether two presented Rubik’s cubes are equal besides the rotation of single elements. The test is conform to the linear-logistic test model and consists of six difficulty levels. Based on the eye tracking data, entropy values and Hidden Markov Models were computed as well as fixation locations analyzed. Hierarchical regression models with the test score as dependent variable show that the eye tracking measures are able to explain ca. 10% additional variance in the easiest and most difficulty levels given accuracy and reaction time of these levels. The results show the potential of eye tracking measures to make CATs more efficient. 

**Eye movement indicators for successful failure detection** **Catrin Hasse, Carmen Bruder**

German Aerospace Center (D.L.R.), Space and Aviation Psychology, Germany catrin.hasse@dlr.de

It is becoming increasingly important for pilots and air traffic controllers (ATCs) to be able to detect automation failures in a timely manner. In the context of personnel selection, conventional tests based on behavioural indicators could be complemented by integrating eye-tracking methods. The present study focuses on revealing eye movement parameters that reflect adequate scanning behaviour, which, in turn, predicts accurate failure detection. Eye movements were recorded whilst subjects were monitoring an automated system and reporting failures. Based on predefined areas of interest (AOIs), eye movement parameters were analyzed within different time units around the automation failure. The data suggest that there are differences between the eye movements of operators who detected automation failures and those who missed them. Human operators who successfully detect an automation failure demonstrate time-specific monitoring patterns. These patterns are quantified by parameters such as fixations counts, gaze durations, mean fixation durations, and the total time to first fixation. Depending on the time frame, different eye tracking parameters become relevant for failure detection, thus reflecting the interplay of the diverse cognitive processes involved. The findings are discussed in the context of the personnel selection and training of aviation operatives, as well as ATC incident reporting.

**Individual objective versus subjective fixation disparity as a function of prism load**

**Wolfgang Jaschinski**

Leibniz Centre for Working Environment and Human Factors, Germany jaschinski@ifado.de

Inaccuracy in binocular eye movement control is referred to as “objective fixation disparity”, which typically is below 1 deg. This can be measured in research, while clinical optometry uses dichoptic nonius lines for measuring “subjective fixation disparity”. To investigate the relation between these two measures, simultaneous tests were made in far vision when placing prisms in front of the eyes (for a few seconds) to vary the absolute disparity (from 1 deg divergent to 3.4 deg convergent). Frequent repeated measurements in 12 observers allowed individual analyses. Generally, fixation disparity values and effects of prisms were much smaller in the subjective than in the objective measures. Some observers differed systematically in the characteristics of the two types of prism-induced curves. Individual regressions showed that the subjective vs. objective slope was - on the average - 8 % (with largest individual values of 18%). This suggests that sensory fusion shifted the visual direction of the (peripheral) binocular targets by the full amount of objective fixation disparity (since single vision was achieved); however, for the (central) monocular nonius lines this shift was more or less incomplete so that the dichoptic nonius lines indicated an individual percentage of objective fixation disparity.

**3D Eye Tracking in Monocular and Binocular Conditions** **Xi Wang, Marianne Maertens, Marc Alexa**

TU Berlin, Germany xi.wang@tu-berlin.de 

Results of eye tracking experiments on vergence are contradictory: for example, the point of vergence has been found in front of as well as behind the target location. The point of vergence is computed by intersecting two lines associated to pupil positions. This approach requires that a fixed eye position corresponds to a straight line of targets in space. However, as long as the targets in an experiment are distributed on a surface (e.g. a monitor), the straight-line assumption cannot be validated; inconsistencies would be hidden in the model estimated during calibration procedure. We have developed an experimental setup for 3D eye tracking based on fiducial markers, whose positions are estimated using computer vision techniques. This allows us to map points in 3D space to pupil positions and, thus, test the straight-line hypothesis. In the experiment, we test both monocular and binocular viewing conditions. Preliminary results suggest that a) the monocular condition is consistent with the straight-line hypothesis and b) binocular viewing shows disparity under the monocular straight line model. This implies that binocular calibration is unsuitable for experiments about vergence. Further analysis is developing a consistent model of binocular viewing.

**Using Priors to Compensate Geometrical Problems in Head-Mounted Eye Trackers**

**Fabricio B. Narcizo, Zaheer Ahmed, Dan W. Hansen**

IT University of Copenhagen, Denmark narcizo@itu.dk

The use of additional information (a.k.a. priors) to help the eye tracking process is presented as an alternative to compensate classical geometrical problems in head-mounted eye trackers. Priors can be obtained from several distinct sources, such as: sensors to collect information related to distance, location, luminance, movement, speed; information extracted directly from the scene camera; calibration of video capture devices and other components of the eye tracker; information collected from a totally controlled environment; among others. Thus, priors are used to improve the robustness of eye tracking in real applications, for example, (1) If the distance between the subject and the viewed target is known, it is possible to estimate subject’s current point of regard even when target moves in depth and suffers influence of parallax error; and (2) if the tridimensional angular rotation is known, it is possible to compensate the error induced by the head rotations using linear regression. Experiments with simulated eye tracking data and in real scenarios of elite sports have been showing that the use of priors to support the eye tracking systems help produce more accurate and precise gaze estimation specially for uncalibrated head-mounted setups.

**The development and validation of a high-speed stereoscopic eye tracker**

**Annemiek D. **^1^**, Nienke Boonstra**^1,2^**, Jeroen Goossens**^1^

^1^Radboud University Medical Centre Nijmegen, Donders Institute for Brain, Cognition and Behaviour,

Department of Cognitive Neuroscience, biophysics section, Nijmegen, Netherlands; 

^2^Bartiméus, Institute for the Visually Impaired, Zeist, Netherlands

A.Barsingerhorn@donders.ru.nl

Traditional video-based eye trackers require subjects to perform an individual calibration procedure, which involves the fixation of multiple points on a screen. However, certain participants (e.g., people with oculomotor and/or visual problems or infants) are unable to perform this task reliably. Previous work has shown that with two cameras one can measure the orientation of the eye’s optical axis directly. Consequently, only one calibration point is needed to determine the deviation between the eye’s optical and visual axis. We developed such a stereo eye-tracker which can track both eyes at ~350 Hz for eccentricities up to 20° with two USB 3.0 cameras and two infrared light sources. A user interface allows online monitoring and threshold adjustments of the pupil and corneal reflections. We validated the tracker by collecting eye movement data from healthy subjects, and compared this data to eye movement records obtained simultaneously with an Eyelink 1000 plus. The results demonstrate that the two-dimensional accuracy of our system is better than 1°, allowing for at least ±5 cm head motion. The average discrepancy with the Eyelink was <1°. We conclude that our stereo eye tracker is a valid instrument, especially in settings where individual calibration is challenging.

Thematic Session: Reading: Predictive and high level processing

**Room 4** (HS 26 - I.13.65)

**Beyond cloze probability: Semantic and syntactic preview effects in reading** **Aaron Veldre, Sally Andrews**

University of Sydney, Australia aaron.veldre@sydney.edu.au 

Theories of eye movement control in reading assume that early oculomotor decisions are determined by a word’s frequency and cloze probability. This assumption is challenged by evidence that readers are sensitive to the contextual plausibility of an upcoming word: First-pass fixation probability and duration are reduced when the parafoveal preview is a plausible, but unpredictable, word relative to an implausible word. The present study sought to establish whether the source of this effect is sensitivity to violations of syntactic rules. The gaze-contingent boundary paradigm was used to compare plausible previews to semantically anomalous previews that either matched or mismatched the word class of the target. Results showed that semantic plausibility was the primary driver of the plausibility preview effect. However, there was an additional benefit from previewing a syntactically valid word that emerged later in the time course, providing direct evidence of parafoveal syntactic processing in reading. These results highlight the limitations of relying on cloze probability as an index of contextual predictability. It is argued that the data are consistent with recent probabilistic accounts of language comprehension.

Are older readers “riskier”? Examining adult age differences in reading 

**Victoria A. , Sarah J. White, Kayleigh L. Warrington, Kevin B. Paterson**

University of Leicester, United Kingdom vm88@le.ac.uk 

Older adults (aged 65+ years) are typically poorer readers than their younger counterparts (aged 18-30 years) and so read more slowly, make more and longer fixations, and make more regressions. But older readers are also more likely to skip past words and so generally move their eyes further forward in the text. Consequently, it has been argued that older readers adopt a “risky” strategy and so guess the identities of upcoming words using partial word information (Rayner et al., 2006), but this has yet to be directly examined. Accordingly, three experiments are presented. Experiments 1 and 2 manipulated target word predictability to examine whether older adults use contextual information to inform these risky decisions. For older adults in both experiments predictability modulated both first pass reading times and the likelihood of skipping. Experiment 3 examined whether older readers use partial word information to guess the identities of upcoming words by using the boundary paradigm to manipulate parafoveal preview information. The results indicate that older adults make risky decisions to skip words even when little useful parafoveal information is available. Implications for understanding how the oculomotor processes underlying reading are affected by older age will be discussed.

**Benchmarking n-gram, topics and recurrent neural network models in predicting word cloze completion and eye movement variance**

**Markus J. Hofmann**^1^**, Chris Biemann**^2^**, Steffen Remus**^2^**, Ralph Radach**^1^

^1^General and Biological Psychology, University of Wuppertal, Germany; ^2^Language Technology, University of Hamburg, Germany mhofmann@uni-wuppertal.de

To several computational linguistics models of language in predicting cloze completion probabilities (CCPs), single-fixation durations (SFDs) and N400 amplitudes, we used item-level regressions on the Potsdam Sentence Corpus (Hofmann, Biemann, & Remus, in press*). We found that the syntactic and short-range semantic processes of n-gram models and recurrent neural networks performed about equally well when directly accounting for CCPs, N400s and SFDs. In contrast, a topics model accounted for a relatively low amount of variance on CCPs and the N400. For SFDs, however, topic models accounted for more variance, suggesting that long-range semantics may play a somewhat greater role in this early and successful word recognition process. When comparing all three language models together against a classic CCP-based approach to SFDs, fisher’s r-to-z tests revealed that the language models outperform CCPs. In ongoing research we are extending this work to word-level analyses applying linear mixed effects models to a variety of oculomotor measures such as fixation probabilities, first fixation and gaze durations, as well as on total viewing times. Results shed light on mechanisms mediating short and long range influences of linguistic processing on eye movement control in reading.

**Predictive processing is key for reading: An evaluation of a visual information optimization model with eye movements in reading**

**Benjamin Gagl**^1,2^**, Christian Fiebach**^1,2,3^

^1^Goethe Universtiy Frankfurt, Germany; 

^2^Center for Individual Development and Adaptive Education of Children at Risk (IDeA), Frankfurt am Main, Germany;

^3^Max Planck Institute for Empirical Aesthetics, Germany

gagl@psych.uni-frankfurt.de

How do we visual information in reading? We propose a visual information optimization process that "explains away", in the sense of predictive coding, redundant visual information of script. This is realized by an image-based subtraction of a prediction, including the redundant visual information based on a lexicon from a presented word. The result of this computation is a prediction error (PE) that represents the specific visual information of a word. By now we could show, in single word presentations, that the PE relates to lexical-decision behavior and early brain activation in occipital regions. Here we evaluate the PE in natural sentence reading (N=82) using eye-movements. PE showed an effect on skipping rates, first fixation and gaze durations (low-to-high PE: increase in skipping and decrease of reading times). Furthermore, PE interacted with word frequency but not with context predictability. This evidence indicates that information optimization is also implemented in natural reading, possibly already in parafoveal vision (i.e. skipping effect). The interaction pattern (higher PE effect for seldom words) reflects that the PE information is relevant especially for lexical access of seldom words. Thus, we conclude that visual information optimization is central not only in artificial but also natural reading contexts.

**The processing of bounded and unbounded negated representations during reading: An eye-movement investigation**

**Lewis T. Jayes**^1^**, Hazel I. Blythe**^1^**, Kevin B. Paterson**^2^**, Simon P. Liversedge**^1^

^1^University of Southampton, United Kingdom; 

^2^University of Leicester, United Kingdom

L.Jayes@soton.ac.uk 

Measures of eye movements during reading have been shown to be sensitive to factors affecting the semantic interpretation of sentences during reading. We investigated the influence of bounded and unbounded expressions on eye movements during reading. Bounded expressions, when negated, must be interpreted as their antonym (not dead=alive). By comparison unbounded expressions possess a scalar ontology so, when negated, are ambiguous (not wide does not equal narrow). Participants read passages with two statements from characters describing bounded/unbounded entities (Experiment 1). The two accounts were either: repetition (not dead-not dead), contradiction (not dead–not alive) or complementary (alive–not dead). The unbounded contradictory condition disrupted reading less than bounded equivalents. Furthermore, unbounded complementary passages were more difficult to interpret than bounded equivalents. In Experiment 2, we found the addition of congruent connectives facilitated the integration of unbounded negation later than it facilitated bounded negation. In Experiment 3, we explored the nature of unbounded representations. The findings show eye movements and reading can be used to detect subtle semantic effects, such as boundedness. The findings provide the first demonstration of boundedness effects on eye movements in reading, suggesting representations of bounded entities are categorically discrete, whilst representations of unbounded entities are continuous.

**Using eye tracking to "figure out" how verb-particle constructions are understood during L1 and L2 reading**

**Mehrgol **^1^**, Laura Gonnerman**^1^**, Veronica Whitford**^2,3^**, Deanna Friesen**^4^**, Debra Jared**^4^**, Debra Titone**^1^

^1^McGill University, Canada; 

^2^Massachusetts Institute of Technology, United States of America;

^3^Harvard University, United States of America; 

^4^Western University of Ontario, Canada mehrgoltiv@gmail.com

Verb-particle constructions (VPCs) vary in form (chew out the boss, chew the boss out) and semantic transparency of their component words (chew out vs. eat up). Thus, like idioms (Titone et al., 2015), VPCs are difficult for second-language (L2) speakers (Blais & Gonnerman, 2013). We used eye-tracking to investigate adult bilingual reading of sentences having adjacent VPCs (chew out the boss), split VPCs (chew the boss out), or jumbled VPCs (out the boss chew), in the first language (L1) or L2. In L1 and L2 reading, gaze durations (GDs) in the VPC region were comparable for adjacent and split VPCs, but longer for jumbled VPCs. However, L1 readers had shorter post-VPC GDs for adjacent vs. split VPCs as their form frequency increased, suggesting that comprehension was facilitated by L1 memory retrieval. In contrast, for L2 readers, increased VPC frequency led to longer post-VPC GDs for adjacent vs. split VPCs, particularly when the verb was semantically related to the VPC (eat up), suggesting that slower semantic integration processes were necessary for L2 comprehension. Thus, like idioms, L1 readers have robust VPC representations that are directly retrieved during comprehension, whereas L2 readers use on-demand semantic integration processes to overcome weaker VPC representations. 

## Wednesday, August 23^rd^, 13.30 - 15.30 

Symposium: : Modeling, Analysis, and Synthesis

**Room1** (HS 14 - M.10.12) 

**Dynamic Modeling of Fixational Eye Movements: The Role of Neural Delays**

**Ralf Engbert, Carl J. J. Herrmann, Ralf Metzler**

Universität Potsdam, Germany ralf.engbert@uni-potsdam.de 

Fixational eye movements (FEM) serve an inherent tradeoff by (i) shifting the retinal image across photoreceptors to prevent visual fading and, at the same time, by (ii) keeping the gaze in a confined area. In our re-analysis of FEM data we found oscillations in the mean square displacement of experimental eye-position data. These oscillations clearly manifest in the displacement autocorrelation function and are almost not affected by a removal of microsaccades (i.e., the fastest component of FEM). These results are compatible with the view that the slow component of FEM (physiological drift) is controlled by a time delayed-feedback loop. Motivated by these experimental findings, we discuss different physiologically plausible mechanisms of a time delay within the theoretical framework of an existing integrative model of FEM. It turns out that time-delayed updating of fixation position is essential to generate oscillations in the correlation functions of simulated data.

**Saliency Surprise Revealed by Microsaccades**

**Yoram S. Bonneh**^1^**, Uri Polat**^1^**, Misha Tsodyks**^2^**, Yael Adini**^3^

^1^Bar-Ilan University, Israel; 

^2^The Weizmann Inst. of Science, Israel; 

^3^Institute for Vision Research, Kiron, Israel

yoram.bonneh@gmail.com 

Although microsaccades and eye-blinks appear stochastic and arbitrary, they have been extensively linked with cognitive processes and attention. In this talk, I will present evidence that links microsaccades and spontaneous eye-blinks to a general "oculomotor inhibition" mechanism that presumably turns-off oculomotor events while processing previous stimuli. I will show that the timecourse of this inhibition could be used as a proxy for the time-course of processing sensory events, providing precise measures for perceptual saliency and surprise without explicit behavior. This allowed us to measure the effect of sensory saliency (visual contrast and spatialfrequency) as well as contrast sensitivity in passive viewing, by just looking at the onset times of microsaccades and eye-blinks, with faster release of inhibition found for more salient stimuli (Bonneh et al. JOV 2015,2016). In contrast, we find longer inhibition for "surprise" in the identity as well as time of items in sequences presented in passive viewing. I will show that the time-course of this oculomotor inhibition depends on the history of preceding events in a precise manner that could be explained by a simple quantitative model that computes the likelihood of future events based on the recent past, assuming longer inhibition for higher prediction error (surprise).

**Evaluating for Cognitive Load Measurement**

**Krzysztof Krejtz**^1^**, Izabela Krejtz**^1^**, Andrew Duchowski**^2^**, Cezary Biele**^3^**, Anna Niedzielska**^3^

^1^SWPS University of Social Sciences and Humanities, Poland; 

^2^Clemson University; United States of America;

^3^National Information Processing Institute, Poland kkrejtz@swps.edu.pl 

We compare and contrast eye tracking metrics for suitability as indicators of cognitive load. Three metrics are tested, thought to be influenced by task difficulty: (1) the change in pupil diameter with respect to inter- or intra-trial baseline, (2) the frequency of pupil diameter oscillation, and (3) the rate and magnitude of microsaccades. Replicating Siegenthaler et al.’s (2014) experiment, participants performed easy and difficult mental arithmetic tasks while fixating a central target (a requirement for replication of prior work). The pupillometric indicator based on the frequency of pupil diameter oscillation implements a revised version of Marshall’s (2000) Index of Cognitive Activity (ICA). Microsaccade detection follows Engbert and Kliegl’s (2003) algorithm with some modifications. An SR Research EyeLink 1000 eye tracker was used for eye movement recording at 500 Hz. Inter-trial change in pupil diameter and microsaccade magnitude appear to adequately discriminate task difficulty. Results corroborate previous work concerning microsaccade magnitude and extend this work by comparing microsaccade metrics with pupillometric measures.

**Microsaccades of Patients during Facial Affect Recognition**

**Nina A. Gehrer**^1^**, Michael Schönenberg**^1^**, Krzysztof Krejtz**^2^**, Andrew T. Duchowski**^3^

^1^University of Tuebingen, Germany; 

^2^SWPS University of Social Sciences & Humanities, Poland; 

^3^Clemson University, United States of America nina.gehrer@uni-tuebingen.de 

Key symptoms of attention deficit hyperactivity disorder (ADHD) include marked difficulty to sustain attention, enhanced distractibility, impulsive and hyperactive behavior. Thus far, only one study has explored microsaccades in adult ADHD patients while performing a continuous performance test (Fried et al., 2014). The aim of the present study was to replicate and extend their finding of deviations in microsaccade parameters linked to attention deficit disorders while viewing emotional facial stimuli. We recorded the eye movements of 21 ADHD patients and 21 matched healthy controls with an EyeLink 1000 eye tracker from SR Research at a sampling rate of 500 Hz. We examined microsaccade magnitude and rate while participants visually inspected the emotional face stimuli.ADHD patients showed a higher microsaccade rate according to a deficit in inhibitory oculomotor control, supporting findings of Fried et al. (2014). Results also suggest an increase in microsaccade magnitude with cognitive load while judging emotional faces, extending the results of Siegenthaler et al. (2014).

**Microsaccades during Visual Search of Gaussian Terrain**

**Justyna J. **^1^**, Andrew T. Duchowski**^2^

^1^SWPS University of Social Sciences & Humanities, Poland; 

^2^Clemson University, United States of America jzurawska@st.swps.edu.pl 

We analyze microsaccades during visual search, which we assume follows Just and Carpenter’s (1976) three-stage model of cognitive processing: search → decision → confirmation. We expect higher cognitive load during the decision-making aspect of the task. To find decision-making periods, we apply our metric (Krejtz et al. 2016) used to distinguish focal and ambient fixations. We compare and contrast microsaccade magnitude and rate with during visual search of Gaussian terrain. Participants were asked to locate an elevated terrain feature embedded in a (Gaussian) surface. The experimental design was a repeated measures factorial design with terrain feature serving as the fixed factor at three levels: low, mid, and high elevation. Our assumption was that these elevations would result in high, mid, and low levels of search difficulty, respectively. Analysis of suggests a greater proportion of focal fixations in the high elevation conditions versus the control condition, especially during the latter stages of inspection. In all conditions, ’s zero-crossing likely suggests transition from search to decision-making. We discuss microsaccade characteristics within this experimental paradigm.

**Perception of Synthesized Microsaccadic Jitter**

**Andrew Duchowski**^1^**, Sophie Jörg**^1^**, Krzysztof Krejtz**^2^

^1^Clemson , United States of America; 

^2^SWPS University of Social Sciences & Humanities, Poland duchowski@clemson.edu 

Eye movements are an essential part of non-verbal behavior, especially when depicting the eyes of avatars or synthetic actors, e.g., in games or film. We have developed a procedural (stochastic) model designed to synthesize the subtleties of eye motion. The main sequence gives a plausible range of durations and corresponding eyeball rotations that are intuitively understood: the larger the eye rotation, the more time required to rotate the eye. The main sequence only describes the duration of movement between fixation points. Fixations can be specified artificially as look points, sequenced by an animator, or captured by an eye tracker. Given a sequence of fixations, the next task is to simulate realistic motion by modeling microsaccadic eye gaze jitter via pink noise. In a series of perceptual twoalternative forced-choice (2AFC) experiments we explored the perceived naturalness of different parameters of pink noise by comparing synthesized motions to the rendered motion of recorded eye movements. Our results showed that, on average, animations based on a procedural model with pink noise were perceived and evaluated as highly natural.

Thematic Session: Saccade control and fixational activity

**Room 2** (HS 32 - K.11.23) 

**Rapid updating of spatial working memory across saccades**

**Artem V. **^1^**, Paul J. Boon**^1^**, Silvia Zeni**^2^**, Jan Theeuwes**^1^

^1^Vrije Universiteit Amsterdam, Netherlands; 

^2^University of Nottingham artem.belopolsky@gmail.com 

Each time we make an eye movement positions of objects on the retina change. In order to keep track of relevant objects their positions have to be updated. The situation becomes even more complex if the object is no longer present in the world and has to be held in memory. In the present study we used saccadic curvature to investigate the time-course of updating of the memorized location across saccades. Previous studies have shown that a memorized location competes with a saccade target for selection on the oculomotor map, which leads to saccades curving away from it. In our study participants performed a sequence of two saccades while keeping a location in memory. The trajectory of the second saccade was used to measure when the memorized location was updated after the first saccade. The results showed that the memorized location was rapidly updated with the eyes curving away from its spatial coordinates within 150 ms after the first eye movement. The time-course of updating was comparable to the updating of an exogenously attended location (Jonikaitis & Belopolsky, 2014), but depended on how well the location was memorized.

**Perceptual continuity across saccades: evidence for rapid spatiotopic updating **

**Jasper H. Fabius**^1^**, Alessio Fracasso**^1,2,3^**, Stefan Van der Stigchel**^1^

^1^Utrecht , Netherlands; ^2^University Medical Center Utrecht, Netherlands; ^3^University of Amsterdam, Netherlands, 

j.h.fabius@uu.nl 

The retinotopic organization of visual information is shifted with each saccade. Yet, we experience a continuous stream of visual information. The discrepancy between the disrupted retinotopic organization and apparent perceptual continuity of visual information has been studied for centuries. It is still debated whether perceptual continuity across saccades is illusory, or whether retinotopic information is updated across saccades. Recent studies provided considerable evidence in favour of spatiotopic updating. Importantly, these studies showed that the build-up of spatiotopic coding takes up to 500 ms, plus saccade latency. Here, we challenge this view by showing that spatiotopic updating occurs within saccade latency. In our experiments, we used the High Phi illusion, where the random texture of a slowly rotating annulus is replaced with four different random textures. Even though the textures are not correlated, the slow rotation induces a strong percept of a large backward jump upon changing the textures. We showed that the illusory backward jump can be induced spatiotopically, and crucially that this updating can be detected even when the presaccadic inducer interval is as short as the saccade latency. These results provide evidence for rapid spatiotopic updating across saccades in much shorter regime than previously assumed. 

**Spatiotemporal dynamics and topological network characteristics of the fixation-related EEG lambda activity**

**Andrey R. Nikolaev, Marcello Giannini, Hossein Seidkhani, Radha N. Meghanathan, David Alexander, Cees van Leeuwen**

KU Leuven - University of Leuven, Belgium

Andrey.Nikolaev@kuleuven.be

Lambda activity is the most prominent and robust brain response occurring at each eye fixation. Known from the early 1950s, it is a widely used indicator of early visual processing in perceptual and cognitive research. In a series of studies we explored the whole-brain dynamical properties of the lambda activity, as well as the functional connectivity networks arising in the lambda interval. We simultaneously recorded EEG and eye movement while participants engaged in unrestricted visual exploration of a display. Analyzing the spatially-smooth phase gradient over the scalp we found that the lambda activity has the spatiotemporal properties of a travelling wave. Next, we compared the functional connectivity networks in the lambda interval between encoding and retrieval stages in a combined visual searchchange detection task. In the frequency range of the lambda activity, we observed differences between the two stages for several network-topological measures, such as mean path length, radius, diameter, closeness and eccentricity, indicating that encoding involves a more segregated mode of operation than retrieval. We concluded that lambda activity, representing early visual processing at fixation, is organized globally, and configured according to perceptual task requirements.

**Microsaccade features and microsaccade-related alpha-synchronization across the life span**

**Ying Gao, Bernhard Sabel**

Otto von Guericke University of Magdeburg, Germany ying.gao@med.ovgu.de 

Microsaccades a significant role in normal vision and are altered in different ophthalmological and neurological diseases. Since these diseases often occur in the elderly population, it is crucial to know if microsaccades are age-dependent. Yet, no study of microsaccades features across the life span is available. The present study aims to fill in this blank with a thorough description of microsaccades and microsaccade-related cortical synchronization in different age groups. High-resolution eye-tracking data were recorded from 19 young subjects (18-29 years), 17 middle-aged subjects (31-55 years) and 18 elderly subjects (56-77 years) during a fixation task. We assessed the microsaccade features, microsaccadic lambda response (MLR) and microsaccade induced alpha band synchronization with dense array EEG. We discovered that in all three age groups, binocular microsaccade percentage, microsaccade rate, amplitude, velocity, duration, horizontal and vertical binocular disconjugacy, the latency and amplitude of MLR were comparable. Alpha waves resynchronized in occipital region with the microsaccades where the microsaccade-related spectral perturbation and inter-trial coherence within alpha band were similar among three groups. Our findings suggest well-preserved microsaccade generation in aging and provide reference for future studies. 

**Unifying micro and macro-saccades with a space dependent, stochastic threshold**

**Geoffrey Megardon, Aline Bompas**

CARDIFF UNIVERSITY, United Kingdom geoffrey.megardon@gmail.com 

It was long thought that peripheral stimuli generate a competition between a saccadic system (i.e., go) and a fixational system (i.e., no go), that were identified, respectively, as the caudal and the rostral part of Superior Colliculus (SC). This strong dichotomy was challenged by the study of fixational eye movements. These contain micro-saccades that have similar properties to “macro-saccades” and are controlled by the rostral part of the SC. Hence, the rostro-caudal axis would code for a continuum of saccade amplitudes. However, computational models fail in finding a mechanism that can initiate both micro and macro-saccades from the same motor map. Paradoxically, micro-saccades are mostly spontaneous and rare events while macro-saccades can be triggered quickly and reflexively by stimulus onset. Using a dynamic neural field, we introduced variability with a stochastic threshold – rather than a noisy input with fixed threshold. That allowed us to control precisely the probability of saccade initiation against the neural activity. Furthermore, our initiation threshold decreased exponentially with saccade eccentricity. These additions created a spatially inhomogeneous variability in saccade initiation across the motor map. The rostral part could trigger rarely small saccades while the caudal part could still generate fast saccadic responses to peripheral stimuli.

**The relationship between visual sampling and hippocampal activity in younger and older adults**

**Jennifer D. Ryan**^1,2^**,Zhong-Xu Liu**^1^**, Kelly Shen**^1^**, Rosanna K. Olsen**^1,2^****

^1^Rotman Research Institute, Canada; ^2^Department of Psychology, University of Toronto jryan@research.baycrest.org

Visual information is accumulated through eye movements, and incorporated into coherent memory representations via function of the medial temporal lobe system, including the hippocampus. The hippocampus and the oculomotor network are anatomically connected through an extensive set of polysynaptic pathways. However, whether visual sampling behaviour is related to hippocampal responses during encoding has not been directly studied in human neuroimaging. Also unknown is whether such a relationship changes during aging, presumably due to age-related declines in the medial temporal lobe structure/function. Here, younger and older adults engaged in a face processing task while brain responses (fMRI) and eye movements were simultaneously monitored. In younger adults, increased numbers of gaze fixations were significantly correlated with stronger hippocampal activation during viewing of novel, but not repeated, faces. Increases in fixations during viewing of novel faces led to larger repetition-related suppression in the hippocampus, suggestive of the ongoing development of lasting representations. By contrast, older adults made more gaze fixations than younger adults, but showed only weak modulations of hippocampal activation by gaze fixations. These results provide novel empirical support for the idea that visual exploration and hippocampal binding processes are inherently linked, and that such an exploration-binding link is altered with aging. 

Thematic Session: Eye-tracking in the educational context

**Room 3** (HS 28 - I.13.71) 

**A tool to assist teachers to determine if learners apply the divisibility rules correctly**

**Pieter Potgieter**^1^**, Pieter Blignaut**^2^

^1^Central University of Technology, South Africa; 

^2^University of the Free State, South Africa blignautpj@ufs.ac.za

Divisibility rules make it easy to determine if a multi-digit number is divisible by a divisor by inspecting only the relevant digits of the dividend. Knowing the divisibility rules will assist learners to simplify mathematical operations such as factorisation, addition of fractions and identification of prime numbers. Learners’ gaze behaviour were investigated to determine if eye-tracking can indicate whether they applied the divisibility rules correctly when they correctly indicated if a dividend is divisible by a specific single digit divisor. A pre-post experiment design was used to investigate the effect of revision on gaze behaviour before and after revision of divisibility rules. The study suggests that if teachers have access to learners’ answers, motivations for the answers as well as gaze behaviour, they can determine if learners (i) guessed the answers, (ii) applied the divisibility rules correctly, (iii) applied the divisibility rules correctly but made mental calculation errors, or (iv) applied the divisibility rules incorrectly. It was also found that revision did not have a significant impact on the percentage of fixation time per digit for learners who provided the correct answer and motivation before or after revision but that the divisor affected gaze behaviour significantly.

**Using Eye-Tracking to Measure Strategies of Comparing the Numerical Values of Fractions**

**Andreas **

University of Education Freiburg, Germany andreas.obersteiner@ph-freiburg.de 

Research suggests that educated adults adapt their strategies for comparing the numerical values of fractions to the type of comparison problem. They use component strategies that do not rely on the fraction magnitudes (e.g., 5/7>2/7 because 5>2), or holistic strategies that do rely on the fraction magnitudes. These results were largely based on verbal self-reports and reaction times. Because these methods are not always reliable measures of strategy use, we used eye-tracking to investigate mathematically skilled adults’ strategies in fraction comparison. To extend previous research on simple fraction comparison, we used a highly controlled set of more complex fractions with two-digit components. We were interested in how often the participants fixated on and alternated between specific fraction components. In line with previous studies, our data suggest that the participants preferred componential over holistic strategies for fraction pairs with common numerators or common denominators. Conversely, they preferred holistic over componential strategies for fraction pairs without common components. Our results support the assumption that mathematically skilled adults adapt their strategies to the type of fraction pair even in complex fraction comparison. This study also suggests that eye-tracking is a promising method for measuring strategy use in solving fraction problems.

**Adapting instruction to learners’ gaze behavior: Does an adaptive multimedia system support learning?**

**Anne Schueler**^1^**, Marie-Christin Krebs**^1^**, Thérése F. Eder**^1^**, Katharina Scheiter**^1,2^

^1^Leibniz-Institut für Wissensmedien, Germany; 

^2^Universität Tübingen, Germany

a.schueler@iwm-tuebingen.de

It is a well-established finding that presenting multimedia materials (i.e., text-picture combinations) can support learning. However, regardless of the effectiveness of multimedia instructions in general, some learners have difficulties to adequately process multimedia materials. To support these learners, an adaptive multimedia system was developed, which provides personalized, just-in-time instructional support. To do so, the system monitors and analyses online the learners’ processing behavior based on the learners’ eye movements. Pursuant to these analyses learners with inadequate processing behavior receive instructional support. In the reported experiment (N = 58) we investigated whether the adaptive multimedia system supports learners in processing multimedia material and whether it is beneficial for learning. We compared two groups: Learners in the experimental group received adaptive instructional support based on their individual gaze behavior, whereas the control group received no instructional support. After learning all participants completed a posttest. Results indicated an interaction between prior knowledge and experimental condition: Learners with higher prior knowledge showed better performance when learning with the adaptive system. Learners with lower prior knowledge, however, performed significantly worse when learning with the adaptive system compared to the control group. Implications for the use of an adaptive multimedia system for individual support are discussed. 

**The effects of conceptual and perceptual difficulty on processing and engagement in text during reading and learning**

**Alexander Strukelj**^1^**, Marcus Nyström**^2^**, Kenneth Holmqvist**^2^

^1^Centre for Languages and Literature, Lund University, Sweden; 

^2^Humanities Lab, Lund University, Sweden alexander.strukelj@englund.lu.se 

To how conceptual and perceptual difficulty affects reading and learning, three eye-tracking experiments were conducted. Subtle low-pass filtering was used as the perceptual difficulty manipulation, learning was measured after 25 minutes, and working memory capacity (WMC) was assessed. When comparing the perceptually difficult text with the control condition, appropriate conceptual difficulty resulted in a shift from shorter to longer total reading times on words (Experiment 1), high conceptual difficulty resulted in shorter total reading times during the entire text (Experiment 2), and low conceptual difficulty resulted in longer total reading times during the entire text (Experiment 3). This suggests that conceptual difficulty interacts with perceptual difficulty and affects processing. Learning outcomes were unaffected by the perceptual manipulation in all experiments, but WMC predicted learning outcomes in Experiment 1, agreeing with previous research. In Experiment 2, participants with lower WMC performed significantly worse compared to participants with higher WMC for the perceptually difficult text only, with longer first fixation durations also observed. This suggests that the high cognitive load from the perceptual and conceptual difficulties was too large to counteract. In Experiment 3, WMC did not predict learning outcomes, likely because the conceptual difficulty of the text was inappropriately low. 

**How are processing strategies reflected in the eyes? Triangulating results from self-reports and eye tracking** **Leen Catrysse, David Gijbels, Vincent Donche** University of Antwerp, Belgium leen.catrysse@uantwerpen.be 

This paper starts from the observation that research in which online process tracking measures, that do not include self-reports, are adopted to uncover differences in students' processing strategies is currently lacking within the Student Approaches to Learning (SAL) field. In this study, we therefore used eye tracking in combination with self-report measures to operationalize and triangulate processing strategies. Forty-two volunteers, with different general preferences towards processing strategies, were purposeful selected for the eye-tracking experiment. Students were asked to study three short expository texts (± 400 words) on positive psychology. Generalized linear mixed effects models were applied with random effects for students and sentences and fixed effects for scores on processing strategies and the type of sentence (key, facts and other sentences). Results indicated that scoring higher on surface processing resulted in a longer first pass fixation duration. In addition, an interaction effect was found between surface processing and type of sentence. Scoring higher on surface processing resulted in a lower first pass fixation duration of key sentences and other sentences in comparison with factual sentences. With regard to the second pass fixation duration and look back fixation duration, no effects of processing strategy or sentence type were found.

Teachers’ perceptions and interpretations of classrooms in the digital age 

**Halszka Jarodzka**^1,2^**, Liesbeth Meijer**^1^**, Sharisse Van Driel**^1^

^1^Open of Netherlands, Netherlands; 

^2^The Humanities Lab, Lund University, Sweden

Halszka.Jarodzka@OU.nl 

Classrooms are complex, information-rich and dynamic environments that require plenty cognitive and attentional resources from teachers to manage them. Nowadays, pupils often bring mobile devices to school, which complicates classroom management even more. With this study, we investigated how experienced teachers perceive and interpret such scenarios by means of eye tracking and verbal protocols. 14 teachers (7 female; 35 - 56 years old; 6 - 30 years of experience) watched four videos of classroom lessons. In two videos pupils were allowed to use mobile devices and in two other videos, this was forbidden. Preliminary results from verbal protocols indicate that teachers mainly focused on whether pupils were paying attention and participating in the lesson. Interestingly, in the ‘mobile device’ videos teachers criticised the use of cell phones but were less critical about laptops and their influence on pupils. Preliminary eye tracking analyses show that for the ‘mobile device’ videos teachers monitor more the pupils’ tables and learning material, in contrast to when mobile devices are not allowed (revisits) and spent less time looking at pupils’ faces (total dwell times). These preliminary findings show how the use of mobile devices by pupils influences teachers’ perception and interpretations of classrooms.

Thematic Session: Reading: Individual differences

**Room 4** (HS 26 - I.13.65)

**Effects of language skills on phonological coding during skilled reading: Evidence from survival analyses of eye movement data**

**Mallorie Leinenger**

Denison University, United States of America leinengerm@denison.edu 

When reading, the meanings of words can be “looked up” directly based on written forms, or written forms can be recoded into phonological codes that are used to access meaning. According to PDP models (Harm & Seidenberg, 2004), these routes work in parallel to mutually inform semantic activation, but the relative contributions of each route can vary. The current study investigated whether differential reliance on these routes varies as a function of a given reader’s individual language skills. Subjects completed language assessments and read sentences containing correct (sensible) target words or anomalous words (phonologically related or orthographically-matched controls) while their eye movements were recorded. Survival analyses of first fixation durations on the phonologically related and control words were conducted to determine how early each individual reader generated phonological codes. Results revealed that readers with better phonemic decoding skills generated phonological codes more rapidly. Furthermore, the rapidity with which a given reader generated phonological codes was more predictive of word identification speed among two groups: skilled phonemic decoders and readers with lower general reading skill, suggesting that the processes associated with word identification can be adjusted to a given reader’s individual set of language skills, maximizing the efficiency of word identification.

**Individual differences and context properties affect word learning** **Victor Kuperman, Bryor Snefjella**

McMaster University, Canada vickup@mcmaster.ca 

Literature that eye-movements to novel words become more efficient with every exposure to those words (Joseph et al., 2014), and the quality of context can modulate the facilitation. We asked how individual variability in statistical learning and other abilities affects both the online process of word learning (e.g., eye-movements) and its outcome (measured through orthographic choice and definition tests). Snefjella and Kuperman (2016) further predicted that novel words are learned better in positive rather than neutral or negative contexts. A sample of (currently) 22 readers were eye-tracked while they each read 9 novel words appearing in 5 contexts each: novel words and context emotionality were counterbalanced. Participants completed a battery of tasks measuring memory retention with a one-week interval. Mixed-effects models showed that better spellers have a strong learning advantage (shorter fixation durations) at initial (1-3) but not later exposures (4-5). We also found that positive contexts and novel words in them are read with significantly less effort, but this advantage does not lead to a long-term memorization benefit. We discuss our findings in light of the Lexical Quality hypothesis, and identify strategies of word learning as a function of both context properties and individual abilities of the reader. 

**Using Latent-Growth-Curve-Models to Examine Children’s Eye-movements During Reading as Individual Difference Variables in Development**

**Christopher J. Lonigan**^1,3^**, Ralph Radach**^2^**, Christian Vorstius**^2^

^1^Florida State University, United States of America; 

^2^University of Wuppertal, Germany; 

^3^Florida Center for Reading Research, United States of America lonigan@psy.fsu.edu 

This study examined intra- and inter-individual differences in age-related development in children’s eye-movements during reading. The sample for this study included 369 children (mean age=106.77 months, SD=19.62) initially recruited when they were in the first, third, and fifth grades. Children participated in eye-movement recording using an EyeLink1000 while they read 48 declarative sentences in both silent- and oral-reading conditions in their initial recruitment year and in each of the subsequent two years (e.g., children initially recruited in 1st grade completed assessments in 1st, 2nd, and 3rd grades). Children also completed standardized measures of word-decoding and vocabulary at the time of recruitment. Latent-growth-curve-models were computed for three time-based metrics (i.e., initial fixation duration, refixation duration, rereading time) on target words (all nouns) in the sentences. All growth models provided good to excellent fit to the data. As expected, there were mean changes in all time-based metrics across time, but there was relatively little reliable individual difference in the rate of change over time. Children’s ages and scores on the psychometric measures predicted intercepts, but, except for refixation duration, slopes were generally accounted for by intercepts (e.g., children with longer initial fixation durations decreased more over time) and age.

**CompLex: An eye-movement database of individual differences in the recognition of morphologically complex words**

**Daniel Schmidtke**^1^**, Victor Kuperman**^2^

^1^University of Alberta, Canada; 

^2^McMaster University, Canada schmiddf@mcmaster.ca 

Massive online databases containing behavioural responses to visual word comprehension tasks provide an important proving ground for theories of lexical processing (e.g., Balota et al., 2007, Kennedy, 2003, Keuleers et al., 2010, Kliegl et al. 2006). For example, centralized and comprehensive visual lexical decision databases are used to advance research on morphological processing. However, there is an absence of such datasets for morphological research during naturalistic reading. With the Complex Word Database (CompLex), we present a large-scale eye-movement study that collected data on individual differences in English complex word processing. A total of 138 students were recruited from McMaster University and 45 adult non-college bound individuals were recruited from the local community in Hamilton, ON, Canada. Participants from both population samples completed a series of eye tracking experiments in which they read complex words embedded in sentence contexts. We present a database for 813 English compound words (e.g., snowman) and 617 English derived words (e.g., snowy), comprising eye-movement data, lexical characteristics for all stimuli, and the results of a battery of skill tests that were administered to participants. The present report describes our motivation for this project, outlines the methods of data collection, and reports initial analyses of the results. 

**An eye movement study of children’s pronoun processing: Individual differences in the detection of incongruence during reading**

**Sarah Eilers, Simon P. Tiffin-Richards, Sascha Schroeder**

Max Planck Institute for Human Development, Germany eilers@mpib-berlin.mpg.de 

Eye tracking is increasingly employed as an approach to investigate children’s online reading of text. The present study aims to better understand the automatic and strategic processes in children’s pronoun resolution. In two eye tracking experiments, we tested fourth graders’ sensitivity to gender feature mismatches during pronoun processing. In our first experiment, we showed children and adults two-phrase sentences like “Max(m) / Mia(f) fed the mouse and then he(m) scrubbed the dirty cage”. Eye tracking measures showed no qualitative differences between children’s and adult’s pronoun processing. For example, both groups showed longer gaze durations on mismatching than matching pronouns. However, in contrast to the adults, not all fourth graders reported the gender mismatch. In a second experiment with a sample of 76 children, we replicated earlier results, and found that about half of the fourth graders detected the gender mismatch. Successful detection was associated with shorter overall gaze durations, but increased total reading times. Moreover, children who detected the mismatch were more likely to make regressions early at the pronoun. We conclude that children who read more fluently use the available resources to immediately repair inconsistencies in text. We discuss our findings with respect to individual differences in beginning readers.

**Oculomotor control in visual tasks predicts reading skill regardless of scanning direction**

**Regina Henry**^1^**, Julie A. Van Dyke**^2^**, Victor Kuperman**^1,2^

^1^McMaster , Canada; 

^2^Haskins Laboratories, CT, United States of America henryr@mcmaster.ca 

The current study investigates 1) the visual scanning hypothesis, which posits that control of eyemovement is part of the underlying link between rapid automatized naming (RAN) and reading; and 2) the effect that interfering with overlearned features of oculomotor control has on this relationship. We recorded eye-movements of 86 undergraduates and 64 non-college-bound young English speaking adults during the reading of text passages and performance of RAN variations. These variations were designed to isolate RAN task components including oculomotor control. To investigate 2, participants were required to perform RAN in the habitual direction of reading (forward RAN) and also backwards: from right to left and top to bottom. The change in scanning direction did not result in significant differences in timing, accuracy or variability of saccades. A small increase in viewing times during backwards RAN in comparison to forwards RAN indicated spatial bias, but only when the grids contained alphanumeric symbols. This difference did not occur during purely oculomotor RAN conditions, indicating that it was likely due to a loss of parafoveal preview advantage rather than oculomotor control. Crucially, participants who were better at oculomotor control were better readers regardless of task type or scanning direction.

## Wednesday, August 23^rd^, 17.00 - 19.00 

Symposium: Insights from Eye Movement Research with Immersive Technologies

**Room1** (HS 14 - M.10.12)

**Using Virtual Reality to Assess Ethical Decisions in Road Traffic Scenarios: Applicability of ValueofLifeBased Models and Influences of Time Pressure**

**Gordon Pipa **

Neuroinformatics, Institute of Cognitive Science, University of Osnabrück, Germany gpipa@uos.de

Selfdriving cars are posing a new challenge to our ethics. Previous research has determined a large variety of factors influencing judgment and behavior in moral dilemmas, evidencing that there is no ground truth for ethical decisions. We, therefore, used immersive virtual reality to assess ethical behavior in simulated road traffic scenarios. Participants controlled a virtual car and had to choose which of two given obstacles they would sacrifice in order to spare the other. We randomly drew objects from a variety of inanimate objects, animals and humans. Utilizing logistic regression, we show that simple models based on onedimensional value of life scales are suited to describe human ethical behavior in these situations. Furthermore, we examined the influence of severe time pressure on the decisionmaking process. We found that it decreases consistency in the decision patterns, thus providing an argument for algorithmic decisionmaking in road traffic. This study demonstrates the suitability of virtual reality for the assessment of ethical behavior in humans, delivering consistent results across subjects, while closely matching the experimental settings to the real world scenarios in question.

**A Virtual reality setup for intensive care unit patients while applying controlled visual and acoustic stimulation**

**Stephan Gerber**^1^

Gerontechnology & Group, University of Bern, Switzerland stephan.gerber@artorg.unibe.ch 

Around 70% of patients in the intensive care unit (ICU) suffer long-term functional deficits after prolonged stay in the ICU, resulting in a reduction of quality of life after discharge. It is assumed that the noisy and stressful ICU environment leads to both stimulus habituation and deprivation in patients which in turn causes cognitive impairment. The aim of the study was to measure the effect of audiovisual virtual reality (VR) stimulation on eye movement and physiological data in healthy subjects in an ICU setting. The VR setting consisted of a head-mounted display in combination with an eye tracker to measure eye movements and sensors to assess physiological parameters. The VR stimulation featured three nature videos and was tested on 37 healthy participants in the ICU. Heart rate, blood pressure and respiratory rate significantly decreased during the audio-visual stimulation. However, the decrease in eye movement data over time was very small and not significant. Fixation/saccade ratio was decreased when no visual target was presented, reflecting enhanced visual search and reduced visual 

processing. Overall stimulation had a strong relaxing and calming effect and the visual search activity was reduced when given attention to a target. 

**The influence of contextual rules on object interactions and spatial representations: a virtual reality investigation** **Dejan Draschkow, Melissa L.-H. Võ**

Scene Grammar Lab, Goethe University Frankfurt, Germany draschkow@psych.uni-frankfurt.de

We investigated the influence of general scene knowledge and episodic memory on participants’ interactions with objects, as well as the detail of spatial memory representations formed during these interactions. In Experiment 1, participants arranged virtual objects consecutively in sixteen rooms. In half of the rooms participants arranged objects in a meaningful way (e.g. placing a pot onto a stove), whereas in the other rooms the objects had to be arranged chaotically. In a subsequent, unannounced, free recall task location memory was assessed by asking participants to rebuild these rooms. Explicit location memory was better for syntactically consistent compared to inconsistently placed objects. The instruction to place objects chaotically lead to longer interaction with objects – measured as object grabbing time. In Experiment 2, participants had to build eight rooms in the same fashion as in Experiment 1, yet this time a surprise search task followed. Participants either searched for objects within the rooms they had built, or within rooms arranged by participants from Experiment 1. Search was speeded for consistently placed objects, especially for objects placed by participants themselves. Our results suggest that contextual violations, even when self-inflicted, lead to differential objectinteraction behavior, as well as a decrease in memory performance.

**Advances in research of anxiety and anxiety disorders using virtual reality**

**Bastian Söhnchen**^1,2^**, Mathias Müller**^1,2^**, Paul Pauli**^1^

^1^Department of Psychology I, Biological Psychology, Clinical Psycholoy, Psychotherapy; University Würzburg, Germany 

^2^VTplus GmbH, Würzburg 

Virtual reality (VR) is an effective and ecologically valid tool for psychological research. By means of computer generated interactive environments, users can be immersed into virtual worlds. These environments are under full experimental control and therefore offer unique means to investigate human behavior in well controlled studies. In addition, VR setups allow for the assessment of multiple behavioural and psychophysiological responses, such as tracking of body- and eye movements, and measurements of skin conductance, electromyography and the cardiovascular system. Next to the application on fundamental research, the investigation of treatment methods, for instance mental disorders can highly benefit from VR as a research tool. In the field of anxiety and anxiety disorders, VR has been successfully used in the framework of exposure therapy and to study contextual conditioning as a model for sustained anxiety. Both approaches will be discussed.

**Research on cognitive architecture of human motor performance and its application in VR environments**

**Thomas Schack**^1,2^**, Kai Essig**^1,2^

^1^Neurocognition Action - Biomechanics- Research Group, Faculty of Psychology and Sport Sciences, Bielefeld University, Germany; 

^2^Center of Excellence "Cognitive Interaction Technology" (CITEC), Bielefeld University, Germany thomas.schack@uni-bielefeld.de

First I will examine the cognitive architecture of human motor performance and show that Basic Action Concepts (BACs) have been identified as major building blocks on a representation level. These BACs are cognitive tools for mastering the functional demands of movement tasks. Research showed that not only the structure formation of mental representations in long-term memory but also chunk formation in working memory are built up on BACs and relate systematically to movement structures and gaze behavior. Then I will discuss challenges and issues that arise when we try to replicate complex movement abilities in the context of interactive technical systems like virtual reality. The research results on mental motor representation combined with the measurement of eye movements cannot only help to understand the cognitive background of motor performance, they also provide a basis for building intuitive interfaces for artificial cognitive systems that are able to learn from the user. This knowledge of how mental representation structures are formed, stabilized and adapted in daily actions enables a coach or technical system (e.g. intelligent glasses and virtual coaches) to address individual users concerning their current level of learning and performance, and to shape instructions to optimize learning processes and maximize performance.

**Using closed-loop-VR to probe human visuomotor control**

**Constantin A. Rothkopf, Huaiyong Zhao, Julia Frankenstein, David Hoppe**

TU Darmstadt, Germany rothkopf@psychologie.tu-darmstadt.de 

Virtual allows generating interactive environments for studying visuomotor control strategies. The statistical relationships connecting task relevant variables, visual display, and the consequences of actions are under the control of the experimenter. This allows a tight control of the variables relevant for human visuomotor control and it also allows manipulating these relationships leading to contingencies that may have never been experienced by participants before. We will present a number of studies that are all targeted at elucidating the visuomotor control policies employed by human subjects in the context of optimal control under uncertainty. E.g., we address the three strategies that have been proposed for locomotor interception. While the pursuit strategy keeps target-heading constant at zero, the constant target-heading strategy keeps target-heading constant at a certain value and the constant bearing strategy keeps the target at a constant bearing angle relative to an allocentric reference axis. Testing these strategies in tasks where participants controlled only locomotion speed along a fixed straight path makes it impossible for subjects to use the pursuit strategy, and it does not allow discriminating between some strategies. We show how to use VR to test which strategy humans may use by systematically manipulating perceptual and control uncertainties. 

Thematic Session: Pupillometry

**Room 2** (HS 32 - K.11.23)

What’s good about big pupils? Sebastiaan Mathôt, Yavor Ivanov 

University of Groningen, Netherlands

s.mathot@cogsci.nl 

The pupil light response is believed to reflect a trade-off between visual acuity (small pupils see sharper) and sensitivity (large pupils are better able to see faint stimuli); that is, pupils take on the smallest size that still allows sufficient light to enter the eye. But why then do pupils dilate when we get aroused, apparently perturbing this delicate trade-off? We hypothesized that the optimal pupil size depends on the situation; specifically, we hypothesized that small pupils are best for calm, focused behavior, whereas large pupils are best for vigilance. To test this, we asked participants to perform one of two tasks: discrimination of a fine tilted grating in central vision (a model of calm, focused behavior); or detection of a faint stimulus in peripheral vision (a model of vigilance). We manipulated pupil size by varying ambient luminance, while keeping the luminance of the task-relevant stimuli constant. We found that discrimination performance did not systematically depend on pupil size; however, detection performance was much better when pupils were large. This suggests that pupil dilation in response to arousal is not, as is often suggested, a nonfunctional epiphenomenon; rather, it optimizes vision for vigilance.

**Attention in visual periphery: Evidence from pupillometry**

**Andreas Brocher**^1^**, Raphael Harbecke**^2^**, Stefanie Hüttermann**^2^

^1^University of Cologne, Germany; 

^2^German Sport University Cologne, Germany abrocher@uni-koeln.de

We used an Attention Window Paradigm with pupil size as dependent measure to study attention in visual periphery. In Experiment 1, trials included cue stimuli that were briefly (300 ms) presented at one of five different angles left and right to eye fixation (12.5°, 20°, 27.5°, 35°, 42.5°). Cues consisted of zero to four black or white triangles and circles. At each trial, participants counted the white triangles in the cues simultaneously presented to their periphery. Response accuracy decreased with increasing angle, p < .001. More importantly, however, pupil size increased with increasing angle, p = .001. In Experiment 2, using the same design as in Experiment 1, but only the angles 12.5°, 27.5°, and 42.5°, participants either counted the white triangles (attention) or reported whether or not stimuli appeared in their periphery (detection). The attention condition replicated Experiment 1, and, crucially, the increase in pupil size was much larger in the attention than the detection condition, p = 027. Our findings open the possibility of testing attention in visual periphery in the absence of an explicit task related to the periphery. Such a paradigm might be appealing to any researcher working on breadth of visual attention.

**Pupil Sizes Scale with Attentional Load and Task Experience in a Multiple Object Tracking Task**

**Basil Wahn**^1^**, Daniel P. Ferris**^2^**, David W. Hairston**^3^**, Peter König**^1,4^

^1^Institute of Science, University of Osnabrück, Osnabrück, Germany, Germany; 

^2^Human Neuromechanics Laboratory, School of Kinesiology, University of Michigan – Ann Arbor, MI, United States of America; 

^3^Human Research and Engineering Directorate, U.S. Army Research Laboratory, Aberdeen, MD, 

United States of America;

^4^Department of Neurophysiology and Pathophysiology, Center of Experimental Medicine, University Medical Center Hamburg-Eppendorf, Hamburg, Germany bwahn@uos.de 

Previous studies demonstrated that attention-demanding tasks modulate pupil sizes. However, to date, researchers have not investigated how attentional load, task experience, and task performance relate to pupil sizes. Here, we investigated how these factors affect pupil sizes in a visuospatial task. To manipulate attentional load, participants covertly tracked between zero and five objects among several randomly moving objects. To investigate effects of task experience, participants performed the experiment on three consecutive days. We found that pupil sizes increased with each increment in attentional load. With increasing task experience, we found systematic pupil size reductions. We compared the model fit for predicting pupil size modulations using attentional load, task experience, and task performance as predictors. We found that a model which included attentional load and task experience as predictors had in terms of the Bayesian information criterion the best model fit. Notably, adding task performance as a predictor reduced the model fit. Overall, these results suggest that pupillometry provides a viable metric for precisely assessing attentional load and task experience in visuospatial tasks.

**Raven revisited: Fixation-related EEG alpha frequency band power and pupil dilation unravel fluctuations in cognitive load during task performance**

**Christian **^1,2^**, Peter Gerjets**^1,2^

^1^Leibniz-Institut für Wissensmedien Tübingen, Germany; 

^2^Fachbereich Psychologie, Universität Tübingen, Germany

c.scharinger@iwm-tuebingen.de

The Raven Matrices test consists of sets of abstract visual figures each missing a specific part. For each figure, participants have to identify the one pattern out of a set of possible solutions shown on the same screen that correctly completes the figure. In the present study using combined EEG and eyetracking we were interested in a) a stimulus-locked data analysis, examining the overall fluctuation of cognitive load (CL) over the course of processing Raven figures, and b) a fixation-related data analysis, comparing CL for fixating different areas of interest (AOIs). The EEG alpha frequency band power and pupil dilation served as measures of CL. The stimulus-locked data analysis revealed increased CL (i.e., increased pupil dilation and decreased EEG alpha power) during the course of working on the Raven figures. The fixation-related EEG data analysis revealed that viewing the correct solution led to increased CL already during initial viewing. CL was low when initially viewing the AOI of the missing part of the figure (i.e., where the integration process has to be performed) and then increased. Pupil dilation 

data partly supported the outcomes of the EEG data. We will discuss these results with a specific focus on methodological challenges.****

**Towards pupil-assisted target selection in natural environments** **Christoph Strauch, Greiter Lukas, Anke Huckauf**

Ulm University, Germany christoph.strauch@uni-ulm.de 

Assisting commands via pupil dilation has been demonstrated in preliminary reports. However, practical usage of such input is still debatable, especially due to the low specificity of signal variations, e.g. through changes in brightness. To critically examine usage and usability of pupils assisting in selection, we implemented and evaluated an onscreen keyboard. Letters were to be selected via a 1.5 s dwell-time, which could be lowered to 730 ms, if a dilation of 0.04 mm within 360 ms and a subsequent constriction of 0.7 mm within 360 ms had been detected during the key-fixation. The screen and the eye tracker were situated next to a window in a user study. 21 users were able to type using pupil-assisted target selection (PATS). Words per minute were slightly lower than those reported for dwell-time. Over 90% of selections were speeded up via pupil. Diameter changes throughout the spelling operation showed an interindividually consistent dilation and constriction, which was however shifted in temporal phase and amplitude. Data suggest that improving the selection algorithm is still possible which might further enhance PATS. Implications of the current findings, e.g. for variations revealing user intent, are discussed, indicating a huge potential for pupil assisted interaction.

**CHAP: An Open Source Software for Processing and Analyzing Pupillometry Data**

**Ronen Hershman**^1,2^**, Noga Cohen**^3^**, Avishai Henik**^2,4^

^1^Department of Cognitive and Brain Sciences, Ben-Gurion University of the Negev, Beer-Sheva, Israel;

^2^Zlotowski Center for Neuroscience, Ben-Gurion University of the Negev, Beer-Sheva, Israel; 

^3^Department of Psychology, Columbia University, New York, United States of America; ^4^Department of Psychology, Ben-Gurion University of the Negev, Beer-Sheva, Israel ronenhe@post.bgu.ac.il 

Pupil dilation is an effective indicator of cognitive load. There are many available eye tracker systems in the market that provide effective solutions for pupil dilation measurement, which can be used to assess different cognitive and affective processes. However, there is a lack of tools for processing and analyzing the data provided by these systems. For this reason, we developed CHAP - an open source software written in Matlab. This software provides a user-friendly interface (graphical user interface) for processing and analyzing pupillometry data. The software receives input of a standard output file from the Eyelink (EDF file) and the Eyetribe (CSV file) eye trackers and provides both pre-processing and initial analysis of the data. Our software creates uniform conventions for building and analyzing pupillometry experiments, and provides a quick and easy-to-implement solution for researchers interested in pupillometry.

Thematic Session: Learning and cognitive information processing

**Room 3** (HS 28 - I.13.71) 

**The use of eye tracker in the discrimination of linguistic and image processing demands in a picture-identification task**

**Erica dos Santos Rodrigues**^1^**, Letícia M. S. Corrêa**^1^**, Elisângela N. Teixeira**^2^

^1^Pontifical Catholic University of Rio de Janeiro, Brazil; ^2^Federal University of Ceará, Brazil ericasr@puc-rio.br 

The processing of object relative clauses (RCs) is particularly hard for language impaired, ADHD children and agrammatic patients. The assessment of linguistic abilities is generally conducted by means of picture-identification tasks involving images describing reversible actions. It is not clear the extent to which image processing contributes to the asymmetry between subject and object RCs as revealed in these tasks. An eye-tracking experiment was conducted aiming to distinguish linguistic and image processing demands in a three-alternative, forced-choice sentence-picture matching task. The task consisted in inspecting a scene while listening to a sentence and clicking on the referent of the complex subject/object. 41 adult speakers of Portuguese were tested. The independent variables were image complexity (complex vs. simple) and type of sentences (subject vs. object RCs) in a 2x2 design. Both independent variables provided significant main effects (p<.05) for all dependent variables (fixation count, total fixation duration and time to first mouse click) and no significant interaction was obtained. The asymmetry between RCs is maintained in the simple image condition but complex images presenting reversible actor-action-object relations add to the overall processing cost. Methodology implications for the assessment of the comprehension abilities of language impaired and ADHD subjects are discussed. 

**Using eye movements to measure conscious and unconscious linguistic knowledge**

**Aline Godfroid**^1^**, Jieun {Irene} Ahn**^1^**, Patrick Rebuschat**^2^**, Zoltan Dienes**^3^

^1^Michigan State University, United States of America; 

^2^Lancaster University, United Kingdom; 

^3^University of Sussex, United Kingdom godfroid@msu.edu 

An important question in the language sciences is whether adults, like children, can develop unconscious or implicit linguistic knowledge (Rebuschat, 2015). Research in this area has relied largely on offline measures, leaving real-time cognitive processes mostly unexplored. To address this issue, we triangulate real-time eye-movement data with two offline awareness measures (retrospective verbal reports and source attributions) and answer the question of whether eye movements during reading foreshadow the type of linguistic knowledge that develops later. Eighty-six English speakers were exposed to an artificial language with English words and German syntax (Rebuschat and Williams, 2012). Participants completed a training phase and a surprise grammaticality judgment test. Their eye movements were recorded throughout the study. Of interest was whether they would acquire conscious and/or unconscious syntactic knowledge. Training data showed all groups learned over time (decreasing sentence reading times and fixation counts); however, only participants who developed conscious knowledge had elevated regression rates (compare Godfroid et al., 2015). Next in this ongoing project, we will analyze eye-movement data from the grammaticality judgment test. We will discuss the benefits of triangulating offline and online measures, including eye-movement recordings, to study grammar acquisition and identify possible eye-movement markers of implicit knowledge. 

Animacy and children’s online processing of restrictive relative clauses 

**Ross G. Macdonald**^1^**, Ludovica Serratrice**^2^**, Silke Brandt**^3^**, Anna Theakston**^1^**, Elena Lieven**^1^

^1^University of Manchester, United Kingdom; 

^2^University of Reading, United Kingdom;

^3^University of Lancaster, United Kingdom ross.macdonald@manchester.ac.uk 

Subject-relative clauses (SRCs, “the dog that chased the cat”) are typically processed more easily than object-relative clauses (ORCs, “the dog that the cat chased”), but this difference is diminished by the presence of an inanimate head-noun. We investigated the influence of animacy on children’s online processing of SRC and ORC sentences. Forty-eight children (aged 4;5–6;5) listened to sentences that varied in the animacy of the head-noun (Animate/Inanimate) and the type of relative clause used (SRC/ORC). Concurrently, while eye movements were monitored, participants saw two images depicting the same two agents, carrying out reversed actions (e.g. dog chasing cat/cat chasing dog) and were asked to choose the picture matching the sentence using a game-pad. As expected, children were significantly more accurate with ORCs with an inanimate head-noun rather than an animate head-noun. However, surprisingly, for SRCs, after the onset of the relative clause (“that...”) participants made more looks more quickly to the target in the inanimate rather than animate condition, suggesting greater anticipation for a SRC with inanimate head-nouns. This may be due to surprisal at inanimate objects acting on animates. Regardless of the cause, our results show children’s anticipatory fixations at relative clause-onset do not predict performance. 

**Can the Eye-Mind Connection Be Broken in the Visual World Paradigm?**

**Anastasiya **^1^**, Anna Laurinavichyute**^2^

^1^National Research University Higher School of Economics, Russian Federation;

^2^National Research University Higher School of Economics, Russian Federation; University of Potsdam

alaurinavichute@hse.ru 

Visual world studies demonstrate that auditory linguistic cues trigger saccades to the referent (Huettig et al., 2011; Knoeferle and Guerra, 2016). Interestingly, eye movement experiments in maintained fixation show that participants can effectively suppress their saccades (Kowler, 2011). We investigated to what extent referential relationships (nouns and pronouns) determine eye movements. We conducted two visual world experiments with the same set of sentences and pictures: in the first, the participants were implicitly allowed free inspection of the visual scene while listening to a story. In the second, they were asked to not look at the picture that the narrator was speaking about. The second group of participants lessened saccades to referent pictures (Est.=-2.55, SE=0.17, p=0.001). Additionally, in the second experiment the probability of fixating an object referred to with a pronoun did not decrease as much as the probability of fixating an object referred to with a noun (Est.=-0.89, SE=0.28, p=0.01): the participants were less able to control their eye movements when hearing a pronoun. Therefore, processing indirect nominations is more effortful, people are searching for more information and use visual context to determine a referent.

**Words and Images: Information Distribution in Comic Panels**

**Clare Kirtley**^1^**, Benjamin W. Tatler**^1^**, Christopher Murray**^2^**, Phillip B. Vaughan**^2^

^1^University of , United Kingdom; 

^2^University of Dundee, United Kingdom clare.kirtley@abdn.ac.uk

While we encounter information presented by both words and images everyday, there is very little research into how readers prioritise and acquire the information simultaneously from the two modalities. Previous work has found that readers prioritise text over images, and do not make frequent movements between the text and image regions. However, this earlier work does not consider how the distribution of information between the word and image regions might affect how regions are prioritized. Using McCloud’s (1995) six categories of word-image combination for comic panels, we presented participants with different versions of single panels in which the text had been adjusted to create the required relationship with the image. Experiment 1 showed that the number of words in the panel, along with comic reading expertise, were the strongest influences on fixations and exploration strategies. In Experiment 2, where words per panel was controlled, the informational relationship between text and image influenced time spent on both text and image, and how readers explored the two regions. Furthermore, words and images were not processed separately: each region influenced 

inspection of the other. Text directed readers to necessary regions of the image, while the image enhanced the meaning of the text.

**Eye-movements in wordless picture stories: Search for comprehension during bridging inference generation**

**John P. Hutson**^1^**, Joseph P. Magliano**^2^**, Lester C. Loschky**^1^

^1^Kansas State University, United States of America;

^2^Northern Illinois University, United States of America jphutson@ksu.edu 

Reading have shown a wide range of comprehension effects on eye-movements, but film and picture story studies have shown only modest effects. This study investigated eye-movements in wordless picture stories during bridging inference generation. We induced bridging inference generation by manipulating ellipses in 3-image target episodes embedded within narratives. In those episodes, half the participants saw the full 3-image episode, while the other half missed the middle image showing a highly inferable action. Magliano et al. (2016) showed that participants in the ellipsis condition inferred the missing action when viewing the third image, and produced longer viewing times. The current study added eye-tracking to test two competing hypotheses to explain the longer viewing times: 1) Computational Load: Inference generation increases fixation durations due to computational load. 2) Visual Search: Inference generation drives eye-movements through search for inferencerelevant information, producing more fixations. Results: Ellipsis trial participants made more fixations, but fixation durations were similar to non-ellipsis trials. We compared fixation heat-maps to inferenceinformativeness heat-maps developed in a separate experiment. Ellipsis trial participants fixated more inference-informative locations. Thus, results supported the Visual Search Hypothesis. During bridging inference generation, participants made more eye-movements to search for information to aid drawing inferences.

Thematic Session: Reading: Corpus analysis and text processing

**Room 4** (HS 26 - I.13.65)

**Russian Sentence Corpus**

**Anna Laurinavichyute**^1^**, Irina Sekerina**^2^**, Kristine Bagdasaryan**^1^**, Svetlana Alexeeva**^3^

^1^National Research University Higher School of Economics / University of Potsdam, Germany; 

^2^City University of New York, United States of America; 

^3^St. Petersburg University, Russia annlaurin@gmail.com

We a corpus of eye-tracking data from 96 individuals reading 144 Russian sentences, analogous in design and structure to the Potsdam sentence corpus (Kliegl et al. 2004). Russian language utilizes an alphabetic script and has rich inflectional morphology. We expected the eye-movement measures to pattern with those reported for other alphabetic languages, as well as to find morphology-related effects. We replicated the main effects found in other languages: reading times in Russian corpus decrease with increase in frequency and predictability, and increase with increase in word length. In addition, increase in the upcoming word's length decreases reading times on the current word. With respect to morphological influence on the eye-movements, we found that, as in Finnish (Hyönä et al. 1995), inflected word forms take longer to read than 'base' word forms. Research on lexical processing has established that verbs are more difficult to process than nouns (Bassano 2000; Szekely et al. 2005; Crepaldi et al. 2011), and we found that gaze durations and total reading times were significantly longer for the verbs than for the nouns. No difference was found in reading morphosyntactically ambiguous and unambigouos words, perhaps while this type of ambiguity is effectively eliminated by the context. 

**PoCoCo: An eye-movement corpus of graphic novel reading**

**Jochen Laubrock, Sven Hohenstein, Eike Richter**

University of Potsdam, Germany laubrock@uni-potsdam.de 

Much of eye tracking research has been devoted to reading and scene perception, but little is known about how these tasks interact. Comics and graphic novels present an ideal testbed for theories of information integration. Here we present a corpus of eye movements while reading comics. The first edition of this corpus, PoCoCo-1, is based on eye movements collected from 100 readers reading passages from six graphic novels. The material is annotated with respect to several variables such as panel location, location of speech bubbles, captions, and text; a more detailed description of the material in terms of various visual features extracted from computer vision methods is underway. First analyses suggest that by far the largest share of time is spent on reading text. Attention appears to be allocated towards the image content in quite a top-down fashion: main characters and story-relevant items are selected first, and little information is devoted to the background. Peripheral vision appears to be used to select information in upcoming panels, and effectively guide the gaze to interesting regions. A planned second edition, PoCoCo-2, will represent eye movements from a smaller number of readers on a much wider selection of material. 

**A Crosslinguistic Investigation of Eye Movements During Reading**

**Denis Drieghe**^1^**, Jukka Hyönä**^2^**, Xin Li**^3^**, Guoli Yan**^3^**, Xuejun Bai**^3^**, Simon Liversedge**^1^

^1^University of , United Kingdom; 

^2^University of Turku, Finland; 

^3^Tianjin Normal University, China

d.drieghe@soton.ac.uk 

Reading is a complex, visually mediated psychological process, and eye movements are the behavioural means by which we encode the visual information required for linguistic processing. Recently, Frost (2012) has argued that establishing universals of process is critical to the development of meaningful, theoretically motivated, cross-linguistic models of reading. To investigate universality of representation and process across languages we examined eye movement behaviour during reading of very comparable stimuli in three languages, Chinese, English and Finnish. These languages differ in numerous respects (character based vs. alphabetic, visual density, informational density, word spacing, orthographic depth, agglutination, etc.). Despite fundamental visual and linguistic differences in the orthographies, statistical models of global reading behaviour (e.g. total sentence reading times) were strikingly similar, and thus, we argue that their composition might reflect some universality of representation and process in reading (Liversedge, Drieghe, Li, Yan, Bai & Hyönä, 2016). In this talk, I will discuss findings from analyses of local eye movement behaviour on specific target words, which show patterns that differ considerably across languages reflecting differences in terms of linguistic and visual density.

**Fluctuations in cognitive engagement during reading: Evidence from concurrent recordings of postural and eye movements**

**Johanna K. Kaakinen**^1^**, Ugo Ballenghein**^2^**, Geoffrey Tissier**^3^**, Thierry Baccino**^2^

^1^University of Turku, Finland; 

^2^University of Paris, France; 

^3^LUTIN , France johkaa@utu.fi

In the present study, thirty-three participants read an expository text with a specific task in mind while their eye and postural movements were concurrently recorded. After reading, readers were asked to recall the text. The results showed that readers spent longer total fixation time and had better memory for task-relevant than irrelevant text information. Individual fixation durations, head-to-screen distance and the speed of head motion decreased more for relevant than irrelevant text segments during the course of reading. The results support the dynamic engagement hypothesis: there is task-induced fluctuation in cognitive engagement during reading. Moreover, the results suggest two types of engagement processes: transient and sustained engagement. The former refers to fast, momentary changes, whereas the latter refers to slower changes in the level of engagement observed across the reading task. The novel combination of eye and postural movement recordings proved to be useful in studying cognitive engagement during reading. 

**Auditory distraction by meaningful background speech during reading**

**Martin R. Vasilev**^1^**, Simon P. Liversedge**^2^**, Daniel Rowan**^3^**, Julie A. Kirkby**^1^**, Bernhard Angele**^1^

^1^Department of Psychology, Bournemouth University, United Kingdom; 

^2^Department of Psychology, University of Southampton, United Kingdom; 

^3^Institute of Sound and Vibration Research, University of Southampton, United Kingdom mvasilev@bournemouth.ac.uk 

Most of reading research has been conducted in a quiet and well-controlled environment. However, everyday reading rarely occurs in such conditions, as readers are often exposed to different noise and speech sounds in the background. Previous behavioural studies have suggested that reading and proofreading performance may be negatively affected by meaningful background speech, but the evidence is mixed. In the present study, we recorded participants’ eye-movements while they were reading single sentences in four background sound conditions (presented at 60 dBA): silence, pink noise, Mandarin speech and English speech. Additionally, in each sentence, there was a target word whose lexical frequency was manipulated. Meaningful (i.e., English) speech prolonged the total reading time of the sentences compared to silence. This was mostly due to making more re-reading fixations. Additionally, English speech resulted in significantly more re-reading fixations and greater regression probability compared to Mandarin speech, thus suggesting that auditory distraction by background speech is mostly semantic in nature (Martin et al., 1988). There were no significant interactions with lexical frequency, which shows that meaningful speech did not interfere with the lexical access of words. These findings suggest that distraction by meaningful speech occurs mostly in the later stages of sentence integration.

**Eye-tracking data analysis using hidden semi-Markovian models to identify and characterize reading strategies**

**Brice Olivier**^1,2^**, Jean-Baptiste Durand**^1,2^**, Anne Guérin-Dugué**^3^**, Marianne Clausel**^2^ ^1^Inria, France;

^2^Laboratoire Jean Kuntzmann, France;

^3^Gipsa-lab, France briceolivier1409@gmail.com 

Textual information search is not a homogeneous process in time, neither from a cognitive perspective nor in terms of eye-movement patterns (Simola, 2008). The research objective is to analyze eyetracking signals acquired through participants achieving a reading task and simultaneously aiming at making a binary decision: whether a text is related or not to some theme given a priori. This activity is expected to involve several phases with contrasted oculometric characteristics, such as normal reading, scanning, careful reading, associated with different cognitive strategies, such as creation and rejection of hypotheses, confirmation and decision. We propose an analytical data-driven method based on hidden semi-Markov models (Yu, 2010), composed of two stochastic processes. The former is observed, and corresponds to eye-movement features over time, while the latter is a latent semi-Markov chain, which preconditions the first process, and is used to uncover the information acquisition strategies. Four interpretable strategies were highlighted: normal reading, fast reading, careful reading, and decision making. This interpretation was derived using the model properties such as dwell times, interphase transition probabilities, and emission probabilities, which characterize the observed process. More importantly, model selection was performed using both, information theory criterion and some covariates, used to reinforce the interpretation. 

## Thursday, August 24^th^, 09.00 - 11.00 

Symposium: Interpreting and using visualizations of eye movements to improve task performance and learning

**Room 1 **(HS 14 - M.10.12) 

**Searching with and against each other**

**Diederick C. Niehorster**^1^**, Tim H.W. Cornelissen**^1,2^**, Ignace T.C. Hooge**^3^**, Kenneth Holmquist**^4^

^1^Lund University, Lund, Sweden; 

^2^Goethe University Frankfurt, Frankfurt, Germany;

^3^Helmholtz Institute, Utrecht University, Netherlands; 

^4^North-West University, South Africa diederick_c.niehorster@humlab.lu.se 

Although in real life people frequently perform visual search together, in lab experiments this social dimension is typically left out. Collaborative search with visualization of partners’ gaze has been shown to be highly efficient (Brennan et al. 2008). Here we aim to extend prior findings to competitive search. Participants were instructed to search a grid of Gabors for a target while being eye-tracked. Participants completed three conditions: individual, collaborative and competitive search. For collaboration and competition, searchers were shown in real-time at which element another searcher was looking. To promote collaboration or competition, points were rewarded or deducted for correct or incorrect answers. Early in collaboration trials searchers rarely looked at the same elements. RTs were roughly halved compared to individual search, although error rates did not increase. This indicates searchers formed an efficient collaboration strategy. During competition overlap increased earlier, indicating that competitors divided space less efficiently. Participants also increased their rate of inspecting search elements and found targets faster than during the collaboration condition, without making more errors. We conclude that participants can efficiently search together when provided only with information about their partner’s gaze position. Competing searchers found the target even faster, but without a clear strategy. 

**Eye What You Are Doing: Inferring Task Performance from Eye Movement Data**

**Margot van Wermeskerken**^1^**, Damien Litchfield**^2^**, Tamara van Gog**^1^

^1^Utrecht University, Netherlands;

^2^Edge Hill University, United Kingdom

m.m.vanwermeskerken@uu.nl

Eye movements provide a window into the mind: fixations show what is at the center of people’s visual attention, which is usually what they are thinking about. However, inferring from a display of someone’s eye movements what they must be thinking, requires substantial interpretation, and little is known about how people make sense of visualizations of other people’s eye movements. Recently, we found that observers were able to judge which relatively simple task instruction was reflected in static or dynamic displays of eye movements. In the present study we used more complex tasks to investigate whether observers are able to infer the (in)accuracy of other people’s task performance from their eye movement patterns. Observers were presented with dynamic and static eye movement displays of another person solving relational reasoning tasks. They were to judge, based on this display, which answer option was chosen. Findings suggest that observers were able to judge above chance whether another person chose the right or a wrong answer. However, judgment accuracy was affected by the distinctiveness of the eye movement pattern: more distinctive patterns resulted in accuracy than less distinctive patterns, with dynamic displays yielding accuracy for less distinctive patterns than static displays. 

**Gaze guidance in number-line tasks**

**Damien **^1^**, Thomas Gallagher-Mitchell**^2^**, Victoria Simms**^3^

^1^Edge Hill University, United Kingdom;

^2^Liverpool Hope University, United Kingdom;

^3^Ulster University, United Kingdom damien.litchfield@edgehill.ac.uk

In this paper we present an investigation into the use of visual cues during number-line estimation, and their influence on cognitive processes for reducing number-line estimation error. Participants completed a 0-1000 number-line estimation task pre and post a brief intervention in which they observed static-visual or dynamic-visual cues (control, anchor, gaze cursor, mouse cursor) and also made estimation marks to test effective number-target estimation. Results indicated that a significant pre-test to post-test reduction in estimation error was present for dynamic visual cues of modelled eye-gaze or mouse movement. However, there was no significant performance difference between pre and posttest for the control condition or static anchor intervention condition. Findings are discussed in relation to the extent to which anchor points alone are meaningful in promoting successful segmentation of the number-line, and whether dynamic cues promote the utility of these locations in reducing error through attentional guidance. More broadly, we highlight the application of dynamic intervention cues to 

improve behavioural responses and the potential for this paradigm to provide a baseline measure of accuracy in following and interpreting gaze cursors. 

**Look Where Eye Looked: Eye Movement Modeling Examples Enhance Learning to Solve Geometry Problems**

**Tim van Marlen**^1^**, Margot van Wermeskerken**^1^**, Halszka Jarodzka**^2^**, Tamara van Gog**^1^

^1^Utrecht University, Netherlands;

^2^Open , Netherlands

T.V.A.vanMarlen@uu.nl 

Eye movement modeling examples (EMME) show students a demonstration of a task by another person (the model), with the model’s eye movements superimposed on the task to guide students’ attention. Earlier research has shown mixed results regarding the effectiveness of EMME compared to regular modeling examples (ME). We hypothesize that this might be related to the ambiguity of the model’s verbal explanation, with EMME presumably being more effective than ME when verbal instructions are ambiguous (i.e., not immediately clear what the model is referring to). To investigate this hypothesis, 108 secondary education students (Mage=12.05, Sd=.46) observed modeling examples on solving geometry problems in a 2 (EMME vs. ME) x 2 (ambiguous vs. unambiguous verbal instructions) betweensubjects design. Results revealed that participants in the EMME conditions outperformed participants in the ME conditions at a problem-solving posttest. Contrary to our hypothesis, the effectiveness of EMME was not affected by the ambiguity of the verbal instructions. These findings suggest that EMME can foster integration of the visual and auditory information. The current results will be discussed in comparison with earlier studies with older participants that only found beneficial effects of EMME on guiding visual attention, without enhancing learning of problem-solving tasks.

**Using Eye Movement Modeling Examples as an instructional tool for learning with multimedia: The influence of model and learner characteristics**

**Marie-Christin Krebs**^1^**, Anne Schueler**^1^**, Katharina Scheiter**^1,2^

^1^Leibniz-Institut für Wissensmedien, Germany;

^2^Universität Tübingen, Germany

m.krebs@iwm-tuebingen.de 

In two experiments, we investigated whether the effectiveness of Eye Movement Modeling Examples (EMME) as an instructional tool is influenced by learner and/or model characteristics. EMME are recorded eye movements of a model while s/he is performing a task, which are superimposed onto the to-be-processed material. In Experiment 1 (n=118), two groups received EMME showing effective multimedia processing strategies by visualizing a skilled model’s eye movements. They were informed that the model was either a successful learner (competent model) or another participant (neutral model). A third group received no EMME. Results indicated that only learners with less prior knowledge benefited from EMME, but only when receiving a neutral model. There was no effect for learners with more prior knowledge. The procedure of Experiment 2 was similar to Experiment 1 except for the fact that participants’ prior knowledge was experimentally manipulated (domain related information vs. domain non-related information before the learning phase). Contrary to our findings in Experiment 1, results indicated that all learners benefited from EMME irrespective of model and learner 

characteristics. Further research is needed to investigate the influence of learner and model characteristics on the effectiveness of EMME in more detail. 

If I showed you where you looked, you still wouldn’t remember

**Ellen M. Kok**^1^**, Avi M. Aizenmann**^23^**, Melissa L.-H. Vö**^4^**, Jeremy M. Wolfe**^2^

^1^Maastricht University, School of Health Professions Education, Maastricht, Netherlands;

^2^Brigham and Women’s Hospital/Harvard Medical School, Cambridge;

^3^University of California, Berkeley, United States of America; ^4^Scene Grammar Lab, Goethe University, Frankfurt, Germany

e.kok@maastrichtuniversity.nl

Prior research shows that observers have poor introspection about their own eye movements. We investigated whether providing observers with online information about where they looked during search would help them recall their own fixations immediately afterwards. Seventeen observers searched for objects in “Where’s Waldo” images for 3s. On 1/3th of the trials, they were asked to click twelve locations in the scene where they thought they had fixated. Half of the scenes were presented normally (control). In the other half, we employed a gaze-contingent window that gave the effect of a 7.5 deg “spotlight” that illuminated everything fixated, while the rest of the display was still visible but darker. To measure performance, we calculated the overlap of circular regions placed over each actual fixation and each click. Ceiling performance was modeled by placing theoretical clicks at each fixation with some added spatial noise. This produced 66% overlap with circles of 2.6 deg. average diameter. Chance performance, modeled by randomly generated ‘clicks’, yielded 21% overlap. Control condition results were 26%, just slightly better than chance. With the gaze-contingent spotlight, performance was 28%, somewhat better than control (p=0.02). Online information about fixations improved memory for those fixations, but only very modestly.

Thematic Session: Oculomotor event detection

**Room 2 **(HS 28 - K.11.23)

**Is human classification a gold standard in fixation detection?**

**Ignace T.C. Hooge**^1^**, Diederick C. Niehorster**^2^**, Marcus Nyström**^2^**, Richard Andersson**^2,3^**, Roy S. Hessels**^1^ ^1^Utrecht University, Netherlands;

^2^Lund University, Sweden;

^3^IT of Copenhagen, Denmark

i.hooge@uu.nl 

Manual classification is a common method to test event detection algorithms. The procedure is often as follows: two or three human coders and the algorithm classify a significant quantity of data. The gold standard approach implies that deviations from the human classification are due to the mistakes of the algorithm. The gold standard approach assumes that humans agree and deliver perfect classifications. This is the first investigation of human coding in eye tracking. Twelve human coders classified fixations in 350s of adult and infant eye tracking data. According to Cohen’s K and F1-scores, the classifications of the humans agreed near perfectly. However, fixation durations and number of fixations differed substantially between the different coders. Merging the classified fixations being spatially close, removed these differences. From that we conclude that human coders may have applied different (implicit) thresholds and selection rules. Another analysis showed that some coders change criteria over time. Based on our results we conclude that human fixation classification is not a gold standard. However, with clever coding instructions, a good coding interface, human classifications can be very useful in testing and building event classifiers for eye tracking.

**Looking sparse? Model-based saccade detection on the position profile**

**David J. Mack**^1^**, Federico Wadehn**^2^

^1^University Hospital Zurich, Switzerland;

^2^ETH Zurich, Switzerland david-jule.mack@iis.ee.ethz.ch

Saccades one of the most commonly tracked events in eye movement recordings. Their main characteristics are short durations and high velocities. Thus, most detection methods operate on velocity or acceleration profiles. However, this is problematic, since digital differentiation amplifies measurement noise. Consequently, strong filtering is required. This however, might distort the signal. The step-like nature of saccades would suggest to use change detection methods on the position profiles to overcome this issue. However, many of these methods require either prior knowledge on the number of saccades or are computationally heavy. Here, we propose a new approach to saccade detection on the position profile based on modelling eye position via a state-space model driven by Gaussian noise (drift) and sparse inputs (saccades). The model parameters can be estimated by Expectation Maximization. Being based on Kalman smoothing, our method is computationally efficient and achieves a similar detection accuracy as simple threshold-based approaches on clean data, but performs significantly better on noisy data. In addition, it has only one tuning parameter which can be used to set detection sensitivity. Finally, the model is easily expandable to account for binocular tracking or include horizontal and vertical channels to further increase its accuracy.

**Towards Low-Latency Blink Detection Using Event-Based Vision Sensors**

**Florian Hofmann**^1^**, Arren Glover**^2^**, Thies Pfeiffer**^2^**, Chiara Bartolozzi**^1^**, Elisabetta Chicca**^2^

^1^Bielefeld University, Germany; ^2^Italian Institute of Technology, Italy fhofmann@techfak.uni-bielefeld.de

Using conventional frame-based cameras in eye-tracking systems requires developers to compromise high frame rates due to limited processing resources. Shifting the paradigm from discrete frame-based to continuous event-based vision supports low-latency eye-tracking with low power consumption towards real-time, closed-loop, embedded eye-tracking solutions. To establish the usability of such sensors, we developed a blink detection algorithm based on event-based optical flow. To that end we introduce motion magnitude as an efficient alternative to other event-based optical flow algorithms. Motion magnitude measures the average moving-edge angle characteristic of event-based vision data, without relying on plane- fitting or PCA. In contrast to established algorithms, it handles the highbandwidth output of current generation event-based vision sensors with less resource requirements. While only approximating the optical flow, it produces a good indicator of the eyelids up and down motion and therefore is well suited for event-based blink detection. Verifying the results against manually annotated data we achieve near perfect (kappa = 0.82, raw agreement = 0.90) inter-annotator reliability even in difficult, changing lighting conditions. This research shows that event-based vision 

sensors are well suited to be used for blink detection. Building upon this we hope to develop resourceefficient, full featured event-based eye-tracking systems. 

**Topology for gaze analyses**

**Oliver **

Universität Hamburg, Germany oliver.hein.home@web.de 

The talk sets out how the application of topological arguments can improve the evaluation of eyetracking data. The task of separating raw, noisy eye tracking data into distinct events (i.e., fixations, saccades, smooth pursuits, and post-saccadic oscillations) on the basis of a single, simple as well as intuitive argument, described as coherence of spacetime, is discussed, and the hierarchical ordering of the data shown. The method, namely identification by topological characteristics (ITop), is parameterfree and requires no pre-processing and post-processing of the raw data whatsoever. The general and robust topological argument is easy to implement and to expand into complex settings of higher visual tasks, making it a powerful tool by which to identify visual strategies.

**End-to-end eye-movement event detection using deep neural networks**

**Raimondas Zemblys**^1^**, Diederick C Niehorster**^2^**, Kenneth Holmqvist**^3^

Research Institute, Siauliai University, Lithuania;

Humanities Lab & Department of Psychology Lund University, Lund, Sweden; UPSET, North-West University (Vaal Triangle Campus), South Africa

r.zemblys@tf.su.lt 

Existing event detection algorithms for eye-movement data almost exclusively rely on thresholding one or more hand-crafted signal features, each computed from the stream of raw gaze data. Moreover, this thresholding is usually left for the end user. Zemblys et al (2017) present an event detector based on Random Forests, where they show how to train a computationally inexpensive classifier to produce oculomotor events, without the need for a user to set any parameters. This approach outperformed conventional event detection algorithms, approaching the accuracy of expert human coders. However, Random Forests and other traditional machine learning algorithms still need a collection of hand-crafted data descriptors and signal processing features. In this paper, we take one step further and use an endto-end deep learning approach to classify raw gaze data into fixations, saccades and PSOs. Our method challenges an established tacit assumption that hand-crafted features are necessary in the design of event detection algorithms. Using manually or algorithmically coded examples, we train a LSTM neural network that produces meaningful eye-movement event classification from raw eye-movement data without any need for pre- and post-processing steps. Its accuracy is also at the level of expert human coders. 

**Comparing Data Evaluation Task Effects on Data Driven Event Detection Models**

**Michael Haass, Matzen Laura, Kristin Divis **

Sandia National Laboratories, United States of America mjhaass@sandia.gov 

Eye movement characteristics, such as fixation duration and saccade velocity, are known to change with different types of viewed content and tasks. Numerous studies report differences in eye movement characteristics across reading and free viewing tasks such as artworks or natural scenes, but few studies have examined comprehension of data visualizations. Furthermore, these assessments are often made using predefined velocity, acceleration and/or dispersion thresholds to separate raw eye tracking data into oculomotor events such as fixations and saccades. This use of predefined thresholds can inherently influence results by skewing the distribution of event types. Higher thresholds can cause distinct fixations to be assigned to a single fixation event whereas lower thresholds can artificially divide a single fixation event into multiple events. In this study, we compare two data driven methods for identifying oculomotor events; mixture models and Nystrom & Holmqvist’s adaptive algorithm (2010), and examine model parameter variations based on task and stimulus conditions. The results showed substantial differences in parameters, and subsequently eye movement characteristics, across certain task goals and minimal differences across other task goals. This approach demonstrates the feasibility, and challenges, for incorporating eye movement features into user models for adaptive visualization and information analysis systems.

Thematic : Usability and web-based interface design

**Room 3 **(HS 28 - I.13.21) 

**Fake sites through the customers' eyes**

**Simone Benedetto, Christian Caldato** TSW Experience Lab, Italy simone.benedetto@tsw.it 

The goal of the present study was to see whether there are differences between expert and novice online shopping users with respect to their navigation behavior on search engine research pages (SERP) and fake websites. Fifteen experts and fifteen novices, were asked to complete three consecutive tasks on a pc while their eyes were tracked. In the first task participant were required to look for a specific garment on a tailormade SERP, and buy it. In the second and third tasks participants were asked to purchase a specific item on two randomly assigned fake clothing websites. As to the behavior on SERP, while experts never go on fake website, novices often fall into the trap: their goal is just looking for the best deal, regardless if it takes to a fake website or not. As to the behavior on fake websites, only the 30% of experts verified the correctness of the url, whereas just the 20% of them noticed the lack of a secure connection (https). Novices never verified neither of them. Overall look and usability seem to 

influence the perceived reliability of a website, rather than the correctness of the url and the presence of a secure connection.

**Children's attention management on commercial websites: Effects of task type and advert prominence**

**Nils Holmberg**

Lund , Sweden nils.holmberg@gmail.com 

This experiment was designed to investigate how children cope with salient online advertising while engaging in task-oriented website interaction. 57 children in 3rd grade (9-year-olds) participated in the experiment. Each participant was introduced to a mock-up website and was instructed to solve two types of online tasks: reading for comprehension and information search. The web pages used by the children contained both task-relevant textual information as well as task-irrelevant online display advertising. The adverts were presented in two saliency conditions: static and animated. Eye movement data were used to differentiate task types in terms of cognitive load, and to construct an advert distraction measure. Pupil dilation data were used to measure children's cognitive load and fixation location data were used to measure attentional advert distraction. The results of the study showed that animated online adverts caused increases in both task-related cognitive load and advert-related fixations compared to static adverts. However, the results also showed that children's level of advert distraction differed between task types, such that advert distraction was higher during task types associated with lower cognitive load (reading for comprehension). The results are discussed in relation to existing cognitive load theory, as well as current media and communication research.

**Reading for Comprehension versus Skim Reading on the Web: The Impact of Hyperlinks and Navigation**

**Gemma Fitzsimmons, Mark J. Weal, Denis Drieghe**

University of Southampton, United Kingdom gemma.fitzsimmons@soton.ac.uk 

Studies of reading have focused on reading behaviour when participants read a single, mono-coloured sentence for comprehension. However, everyday reading behaviour, such as reading on the Web often entails people skim reading passages of text containing coloured links. We ran an experiment where participants simply read Webpages presented to them and another where participants could click and navigate through the Webpages. We recorded participants’ eye movements while they read modified pages from Wikipedia (that contained target words) and asked them to read for comprehension or skim read. Target words were either hyperlinked/unlinked, and either high/low-frequency. Linked words were skipped less often than unlinked words when skimming, revealing that participants used the coloured words as ‘anchor’ points for scanning strategies. In both experiments, frequency effects were observed during reading for comprehension but not during the skimming task, except when the words were hyperlinked. This indicated more advanced lexical processing for the linked target words. Comprehension was reduced when clicking and navigating suggesting this extra task of navigating impacts on comprehension of the text. Results are discussed in terms of task effects on eye movements during reading and the necessity to also study reading behaviour in more realistic settings. 

**Learning 3D from 2D views: insights from eye movement behaviour during multiplex screen viewing**

**Kenneth C. Scott-Brown**^1^**, Matthew J. Stainer**^2^**, Benjamin W. Tatler**^3^

^1^University of Abertay, United Kingdom;

^2^Griffith University, Australia;

^3^University of Aberdeen, United Kingdom

k.scott-brown@abertay.ac.uk

Multiplex video displays are increasingly used by security operatives to patrol and secure complex areas of the built environment. A key component of CCTV surveillance is learning 2D views of the 3D observed space, yet little is known about how expertise in CCTV surveillance operation is achieved. We recorded eye movements while untrained observers watched a 6-camera multiplex video array showing actors walking through an environment. Participants viewed the actors moving through the environment repeatedly, allowing us to chart the changes in oculomotor behaviour that occurred as the participants learnt the mapping between scenes in the display. Over repeated viewings, participants spent more time looking at the scene containing the target actor, and were sooner to move their eyes to the next scene when the actor transitioned between scenes. Anticipation was evident, with participants moving their eyes to the screen that the actor would appear on before the actor left the previous screen. This decreasing inspection of irrelevant scenes, together with a visual anticipation of the actor’s future location provide quantitative and continuous measures of observers’ understanding of the relationship between the 2D screens and the observed events in the 3D environment.

**Visual and neural co-activation reflect conscious processing during prosthetic hand use, but only during object manipulations**

**Johnny V. V. Parr**^1^**, Neil Harrison**^1^**, Sam Vine**^2^**, Mark Wilson**^2^**, Greg Wood**^3^

^1^Liverpool Hope University, United Kingdom;

^2^Exeter University, United Kingdom;

^3^Manchester Metropolitan University, United Kingdom parrj@hope.ac.uk

Prosthetic hand devices are often poorly utilised and frequently rejected. High rejection rates have been attributed to a high cognitive burden imposed on users. We investigated the nature of this burden by simultaneously examining gaze behaviour and EEG coherence between the verbal-analytical (T7) and motor planning (Fz) regions in able-bodied participants using a prosthetic hand simulator. Twenty participants were required to perform 30 trials of the “lifting a heavy object” task from the Southampton Hand Assessment Procedure (SHAP) using their anatomical hand and the prosthesis. During performance, recorded gaze behaviour determined spatial and temporal characteristics of visual attention. EEG was recorded to compute high-alpha (10-12Hz) T7-Fz coherence to determine conscious movement control during the reaching and grasping phases. Participants were significantly slower, used more hand-focused gaze and took longer to disengage vision from hand movements when using the prosthesis. Disruptions were multiplied during manipulation of the jar. The dependence on vision during the manipulation phase coincided with increased T7-Fz coherence, suggesting conscious movement control during this movement phase. Findings suggest a link between increased visual attention and verbal-analytical processing is related to the cognitive burden associated with prosthetic hand rejection. Highlighted metrics could test rehabilitation strategies and inform prosthesis design.

Thematic Session: Reading Basic oculomotor control

**Room 4 **(HS 26 - I.13.65)

**Oculomotor adaptations when reading mirror-reversed texts**

**André Krügel, Johan Chandra, Ralf Engbert**

University of Potsdam, Germany kruegel@uni-potsdam.de 

The control of eye movements during reading is an important example that demonstrates how sensory processes and prior knowledge are integrated for optimal motor behavior. The launch-site contingent shift of saccades’ mean landing position within words is a remarkably robust signature for using prior knowledge for saccade planning during reading (McConkie et al., 1988; Engbert & Krügel, 2010). However, while prior knowledge ensures stability under sensory uncertainty, optimal saccadic behavior requires flexibility for adaptation to changing reading conditions. Here we present results of an extensive reading experiment with different conditions of mirror-reversed texts, some of which invert the normal left-to-right reading direction within words. Most importantly, we found substantial changes in the launch-site effect when reading inverted texts. Interestingly, an inverted within-word reading direction leads to a reduction of the launch-site effect, but a letter-wise mirror-reversed text with maintained within-word reading direction leads to an increase of the effect. The results are compatible with the view that readers flexibly adapt the weighting of prior knowledge against sensory processing during saccade planning when faced with new reading conditions. 

**Eye Movement Control for Horizontal and Vertical English Text**

**Sha Li**^1^**, A. AlJassmi**^2^**, Kayleigh L. Warrington**^3^**, Sarah J. White**^3^**, Jingxin Wang**^1^**, Mercedes **

**Sheen**^2^**, Timothy R. Jordan**^2^**, Kevin B. Paterson**^3^

^1^Tianjin Normal University, China;

^2^Zayed University Dubai, UAE;

^3^University of Leicester, UK llesq@outlook.com 

Text in English usually is read horizontally from left-to-right, and mechanisms of eye movement control for this conventional reading direction are relatively well understood. However, text sometimes is displayed in unconventional formats and findings show that reading is slower for vertical than horizontal reading directions (Yu, Park, Gerold, & Legge, 2010). Whether this slower reading results from impaired word identification or poorer saccade-targeting for unconventional reading directions is unclear. Accordingly, we assessed effects of reading direction on eye movements in two experiments that manipulated either the length (4-letter vs. 10-letter) or lexical frequency (low vs. high) of a target word in sentences, while controlling for other factors. Sentences were displayed normally, rotated 90° clockwise or counter-clockwise, or in a marquee format in which upright letters were arranged vertically. Reading was slower for vertical than horizontal displays. Moreover, while standard effects of word length and lexical frequency were obtained for all displays, these effects were greater for vertical displays, indicating that word identification was disrupted during vertical reading. Text format did not affect the location of initial fixations in target words, however, indicating that saccade-targeting was unimpaired. We discuss these findings in relation to models of eye movement control during reading. 

**How MASC, a Model of Attention in the Superior Colliculus, pretends to read despite being completely illiterate!**

**Françoise Vitu**^1^**, Hossein Adeli**^2^**, Gregory, J. Zelinsky**^2^

^1^CNRS, Aix-Marseille Université, France;

^2^Stony University, NY, United States of America

Francoise.Vitu-Thibault@univ-amu.fr

Existing models of eye-movement control during reading lack neurobiological plausibility and often overstate the role of language-related processes. They also use many free parameters, obscuring the nature of the processes that generate a given eye-movement pattern. In contrast, MASC, our Model of Attention in the Superior Colliculus (SC), predicts well-established properties of eye guidance during reading (e.g., the preferred-viewing-location effect), while being completely illiterate. MASC is grounded in core principles of saccade programming in the SC, and uses a minimal number of parameters taken directly from neurophysiology. It generates sequences of saccades by (1) extracting luminance contrast over a sentence's image, (2) projecting a computed saliency map into SC space, where space closer to the fovea is over-represented, (3) averaging activity over translation-invariant neuronal populations in visual and motor maps, (4) programming a saccade to the location in space corresponding to the center of the maximally active population, and (5) inhibiting the fixated location in the saliency map before repeating the cycle. We will report a "dissection" of MASC, and show that its success stems from population averaging in the distorted space of the SC, a fundamental principle that should be central to any model of eye-movement control. 

**Eye-Movement Evidence for Object-Based Attention in Reading**

**Yanping Liu**^1^**, Erik D. Reichle**^2^

^1^Sun Yat-sen University, People's Republic of China;

^2^University of Southampton, United Kingdom liuyp33@mail.sysu.edu.cn

Is attention allocated to only one word or multiple words at any given time during reading? The experiments reported here address this question using a novel paradigm inspired by Duncan’s (1984) classic findings of object-based attention. In Experiment 1, participants made lexical decisions about one of two spatially co-located words, with the key result being that only the attended word’s frequency influenced response times and accuracy. In Experiment 2, participants read target words embedded in two spatially co-located sentences, with the key finding being that the target words’ frequencies had a larger, more rapid influence on looking times than did (unattended) distractor words. These results provide evidence consistent with the hypothesis that words are attended in a strictly serial (and perhaps object-based) manner during reading. The theoretical implications of this conclusion are discussed in relation to models of eye-movement control during reading and the conceptualization of words as visual 

“objects“. 

**The impact of forced fixations on word recognition: Dissociation of oculomotor behavior and linguistic processing**

**E. R. Schotter**^1^**, Mallorie Leinenger**^2^**, Titus von der Malsburg**^3^

^1^University of South Florida, United States of America;

^2^Denison University, United States of America;

^3^Potsdam University, Germany eschotter@usf.edu 

Easy parafoveal processing not only causes word skipping, but also forced fixations on words, i.e., short single fixations due to pre-initiated forward saccades (Schotter & Leinenger; 2016), which can explain standard preview benefit effects (cf. linguistic integration accounts: Rayner, 2009) and reversed preview benefit effects—longer fixations following identical than higher frequency unrelated previews. An open question is whether, following forced fixations, linguistic processing proceeds from parafoveal information that initiated the saccade or higher fidelity foveal information. Twenty-four subjects read 150 sentences in the boundary paradigm (Rayner, 1975) with orthogonally crossed high- and lowfrequency preview and target words that were plausible at the point of the target region and intervening buffer region and then neither/one/both became implausible at a sentence-final critical region. We replicated Schotter and Leinenger (2016) and found that regressions out of the buffer region showed only an effect of the display change while regressions out of the critical region showed only an effect of target word plausibility. These data suggest a dissociation in the reading system: immediate oculomotor behavior is based on “hedged bets” initiated by low-acuity parafoveal information, emerging linguistic processing occurs mostly based on high-acuity foveal vision, and display changes are sometimes immediately detected. 

**Word demarcation in reading of newly learned strings: There’s something special about spaces.**

**Mengsi Wang, Hazel I. Blythe, Simon P. Liversedge**

University of Southampton, United Kingdom mw2u14@soton.ac.uk 

Removal of word demarcation causes interference for word identification and saccadic targeting during reading. Here, we explored how word exposure (frequency) and word demarcation affected eye movements in a Landolt-C learning and reading paradigm. During learning, participants learnt LandoltC triplets with high or low exposure frequency. Post-learning recognition was assessed in a lexical decision task. In the reading phase, participants “read” sentence-like Landolt-C strings with different formats (unspaced, highlighted, spaced) to decide whether a target word was present. During learning, accuracy increased and processing time decreased across blocks. Exposure frequency moderated learning. During reading, saccadic targeting and word processing time were affected by word demarcation; shorter times on spaced strings and highlighted strings. We also replicated findings of initial landing position distributions with quadratic PVL curves for spaced strings and negative PVL curves for unspaced/highlighted strings. Frequency effects increased with block during learning but did not maintain to reading indicating exposure frequency did not accelerate string identification in reading. Word spacing and highlighting facilitated word identification due to disambiguation of word boundaries. Also, spacing did, but highlighting did not benefit saccadic targeting probably due to lateral masking, indicating that there is something special about spaces for saccadic targeting. 

## Thursday, August 25^th^, 11.30 - 13.30 

Symposium: Pharmacological Influences on Voluntary Oculomotor Control

**Room1 (**HS 14 - M.10.12**) **

**Effects of NMDA antagonists on voluntary control of eye movements in nonhuman primates **

**Pierre Pouget**^1^**, Marcus Missal**^2^

^1^Institute of Brain and Spinal Cord (ICM) Paris France; 

^2^Institute of Neuroscience (IONS) Brussels Belgium pierre.pouget@upmc.fr 

The neuropharmacology of time perception is complex and still poorly understood. Subanesthetic doses of ketamine can induce distortions of time perception suggesting that glutamatergic transmission at NMDAr synapses is essential. Often, humans under light ketamine influence report that "time slows down". It has been shown that the effect of ketamine in healthy humans is specific to timing. In the oculomotor domain, the precise timing of anticipatory and visually-guided saccades rest on an implicit estimate of elapsed time. Particularly, if there is a random delay drawn from a uniform distribution between the disappearance of the fixation target and the appearance of an eccentric one, saccadic latency of voluntary movement decreases as time elapses during the delay period. This is often referred to as the foreperiod effect. In a series of experiments, we will show that a subanesthetic dose of ketamine or memantine suppresses anticipatory saccades and alters the foreperiod effect. We suggest that NMDA antagonists could alter neural processes of implicit timing and modify the decision threshold at which a voluntary movement is produced. 

**Effects of Ketamine on Brain Function during Smooth Pursuit and Antisaccade Eye Movements in Healthy Humans **

**Maria , Anna Kasparbauer, Inga Meyhöfer, René Hurlemann, Ulrich Ettinger****

University of Bonn, Germany maria.steffens@uni-bonn.de 

Ketamine has been proposed to model symptoms of psychosis. Impairments in smooth pursuit eye movements (SPEM) and antisaccades (AS) are established biomarkers and endophenotypes of schizophrenia spectrum disorders. SPEM impairments have also been demonstrated during ketamine administration in healthy volunteers. However, the neural mechanisms of ketamine on eye movements in healthy humans have not been characterized. Here, twenty-seven healthy participants received racemic ketamine (100 ng/ml target plasma concentration) in a within-subjects, double-blind, placebocontrolled study. Participants performed a SPEM task and an AS task during functional magnetic resonance imaging (fMRI). Self-ratings of psychosis-like experiences were obtained using the Psychotomimetic States Inventory (PSI). Ketamine administration induced psychosis-like symptoms and led to robust deficits in SPEM performance, accompanied by reduced blood oxygen level dependent (BOLD) signal in the SPEM network compared to placebo. These results are similar to the deviations found in schizophrenia patients. In contrast, AS error rate and BOLD response to the AS task were not affected by ketamine. Overall, our findings support the role of glutamate in SPEM and provide partial support for the use of ketamine as a pharmacological model of psychosis. ****

**Neuropharmacology of cognitive control: local manipulations of the dopaminergic and cholinergic system in monkey prefrontal cortex during antisaccade performance**

**Susheel Vijayraghavan**^1^**, Alex James Major**^2^**, Stefan Everling**^3^****

^1^Robarts Research Institute,The University of Western Ontario, London, Canada;

^2^Graduate Program in Neuroscience, The University of Western Ontario, London,Canada; ^3^Department of Physiology and Pharmacology, The University of Western Ontario, London, Canada svijayra@uwo.ca 

The prefrontal cortex (PFC) is a critical locus in circuitry mediating cognitive control and inhibition of reflexive responses. Ascending neuromodulatory systems have a profound influence on PFC cognitive functions. Dopaminergic and cholinergic influence has been extensively studied in oculomotor spatial working memory paradigms in PFC. Here, we present our recent investigations of the influence of these systems on representations of rules in PFC during performance of pro- and antisaccades. We found that local dopamine D1 receptor stimulation suppresses PFC physiology and disrupt rule representation of PFC neurons, while D2 receptors modulate the strength of PFC saccade-related activity for reflexive saccades, while sparing antisaccade selectivity and rule representation. We have previously reported that cholinergic muscarinic receptor blockade strongly disrupts all aspects of PFC activity during this task, thus suggesting an excitatory role for muscarinic receptors in PFC. Based on anatomical and physiological evidence and abundance of M1 muscarinic receptors in cortex, we hypothesized that M1 receptor stimulation would facilitate rule representation in PFC. Here we report that, contrary to expectations, M1 receptor stimulation has a significant inhibitory influence on PFC neurons engaged in the antisaccade task. Our results have interesting implications for pharmacological interventions in ameliorating PFC-dependent cognitive dysfunction. 

**Model based analysis of dopaminergic and cholinergic neuromodulation on voluntary control of eye movements in humans **

**Jakob Heinzle**^1^**, Dario Schöbi**^1^**, Klaas Enno Stephan**^1,2^**, Eduardo A. Aponte**^1^****

^1^University of Zürich and ETH Zürich, Switzerland; 

^2^Wellcome Trust Centre for Neuroimaging, UCL, London, UK 

Cholinergic and dopaminergic neuromodulation influence voluntary control of eye movements. Here, we present the results of two single dose, within subject placebo controlled drug studies. A novel computational model was used to explain reaction times and error rates in a paradigm with varying probabilities of pro- and antisaccades. In the first study, participants received a single dose of levodopa or placebo in two separate sessions. Levodopa increases the availability of dopamine within the brain which led to a reduction in error rates in prosaccade trials, without significant effects on reaction times. Dopamine slowed down the timing of the model unit that controlled reaction times of voluntary saccades. In addition, dopamine increased the number of voluntary, relatively slow prosaccades. The second study used galantamine which increases availability of acetylcholine. This led to faster reaction times for antisaccades and made the voluntary saccade unit faster. A drug by weight interaction affected the antisaccade error rate suggesting the amount of available acetylcholine influenced the probability of making errors. In summary, a model based analysis showed opposite effect for dopamine and acetylcholine on voluntary saccades: Dopamine increased the timing and changed the saccade goal of voluntary saccades, while acetylcholine reduced reaction times of voluntary saccades. 

**Cholinergic and Dopaminergic Influences on Eye Movements in Humans **

**Ulrich Ettinger, Anna Kasparbauer, Maria Steffens, Inga Meyhöfer, Eliana Faiola, Nadine Petrovsky **Department of Psychology, University of Bonn, Germany ****ulrich.ettinger@uni-bonn.de 

In this talk, I will present evidence from studies of the effects of pro-cholinergic and pro-dopaminergic substances on eye movements in healthy humans. Nicotine is an agonist at the nicotinic acetylcholine receptor (nAChR) that is widely consumed via smoking of tobacco. In a number of studies, we have found that nicotine improves performance on the antisaccade task. Effects on antisaccades may depend on baseline performance in healthy subjects but are observed across schizophrenia spectrum samples (patients, highly schizotypal subjects, controls). The neural effects of nicotine during antisaccades involve reduced frontal eye field activation. Beneficial effects of nicotine are also observed on smooth pursuit, under conditions of increased demands on the pursuit system. Methylphenidate is a dopamine transporter blocker that is used in the treatment of ADHD. Administration of methylphenidate improves smooth pursuit and increases the timing of saccades under conditions of temporal predictability of the stimulus. At the level of brain function, nicotine and methylphenidate show opposing effects in frontal eye field during smooth pursuit, with nicotine leading to a decrease and methylphenidate to an increase in activation. Overall, these findings suggest that these putative cognitive enhancers may have beneficial effects on different aspects of eye movement control.

Thematic seesion: Saccade programming II

**Room 2 **(HS 32 - K.11.23) 

**Dissociating automatic capture, to individual stimuli or the global effect location, from intentional saccade targeting **

**David Aagten-Murphy, Paul M. Bays**

Cambridge University, United Kingdom ****david.aagtenmurphy@gmail.com 

In the presence of multiple objects, eye-movements may be “captured” to the location of a distractor object, or biased towards the intermediate position between objects ("global effect"). We examined how the relative strengths of the global effect and visual object capture changed with saccade latency, the separation between visual items and stimulus contrast. Importantly, while many previous studies have omitted giving observers explicit instructions, we instructed participants to either saccade to a specified target object or to the midpoint between two stimuli. By implementing a novel, probabilistic mixture model analysis we quantified the probability of saccades landing at either the target, distractor, or intermediate locations at different saccade latencies. Comparing model weights across the tasks then allowed us to distinguish between automatic, unavoidable capture to either the global effect or stimulus location (most prevalent for rapid saccades with the likelihood of each depending on spatial separation) and the intentional, goal-directed targeting of saccades towards the current task goal (increasing influence as latency increases). Overall, these results suggest that previous studies may have overestimated the global effect by confounding the influences of stimulus capture, global effect, and goal-directed processes on saccade landing distributions. 

**Asymmetries of the saccadic system: A tool to quantify eye dominance strength **

**Jérôme Tagu**^1^**, Karine Doré-Mazars**^1^**, Christelle Lemoine-Lardennois**^1^**, Judith Vergne**^1^** & Dorine **

**Vergilino-Perez**^12^****

^1^Laboratoire Vision Action Cognition (EA7326), Institut de Psychologie, Institut Neurosciences et 

Cognition, Paris Descartes University, Sorbonne Paris Cité, France; 

^2^Institut Universitaire de France, Paris, France jerome.tagu@parisdescartes.fr 

The system presents multiple asymmetries. Notably, peak velocities are higher for temporal than for nasal saccades and for centripetal than for centrifugal saccades. We have already shown with binocular recordings (Vergilino-Perez et al., 2012) that participants with weak eye dominance exhibit the classical naso-temporal asymmetry while participants with strong eye dominance exhibit higher peak velocities for a given saccade direction whichever the recorded eye. This categorization of eye dominance strength however remains binary. The current study tests the naso-temporal asymmetry over different conditions so as to provide a finer quantification of eye dominance strength. We ask participants to make centripetal and centrifugal saccades from five different locations. Analyses on the saccadic peak velocities of 48 participants show that the presence of the naso-temporal asymmetry (signing a weak eye dominance) depends on the centripetal or centrifugal nature of the saccade. We propose for the first time a graduated measure of eye dominance strength on a continuum from no eye dominance to very strong eye dominance. Indeed, by testing the naso-temporal asymmetry over different conditions, we assign to each participant a percentage of eye dominance. Potential physiological origins of the asymmetries found in the saccadic system will be discussed. 

**Saccade countermanding reflects automatic inhibition as well as top-down cognitive control **

**Aline Bompas, Annie Campbell & Petroc Sumner **

Cardiff University, United Kingdom ****bompasae@cardiff.ac.uk 

Saccade countermanding is commonly employed for investigating cognitive control, as typically modelled by competing go and (top-down) stop processes. However, saccade initiation can also be interrupted automatically by visual stimulus-evoked activity in motor programming circuits, causing dips in latency distributions (a phenomenon called ‘saccadic inhibition’). We hypothesised that this low level effect may account for a large proportion of the saccade countermanding process when visual signals are used. Here, we used the same stimuli and same participants but different instructions, in order to compare the latency distributions for failed countermanding with the latency distributions for distractor-induced dips. We find dips in both contexts time-locked to the onset of the visual signal, both beginning ~100 ms following signal onset. We further use a biologically-inspired model of saccade generation (DINASAUR) to illustrate that distributions following both instructions can be captured by assuming a common automatic mechanism for saccadic inhibition and initial countermanding. We propose that top-down inhibition acts later in the distribution suppressing the post-dip recovery period, piggy-backing on the more rapid automatic saccadic inhibition. We conclude that SSRTs calculated from these experiments do not represent top-down inhibition alone, but rather the interaction of top down and bottom-up inhibition effects. 

**Oculomotor gap effect and antisaccade performance in the common marmoset **

**Kevin & Stefan Everling****

University of Western Ontario, Canada********kjohnst9@uwo.ca 

The common marmoset (Callithrix jacchus) is a New World primate that shows promise as a model animal for oculomotor research. To date however, a limited number of studies have evaluated the oculomotor performance of this primate species. Here, we investigated the saccadic behaviour of the common marmoset on a suite a suite of tasks, performed for liquid reward. Consistent with previous observations in humans and macaques, we observed a prominent “gap effect” – a reduction in SRTs in a “gap” as compared to a “step” saccade condition. We further investigated the ability of this animal to perform an antisaccade task in which saccades made in the direction opposite a suddenly appearing salient visual stimulus were rewarded. Performance was accurate under conditions in which a small dim placeholder was present at the opposite location, but not when no placeholder was present – i.e. when saccades to an internally-generated representation of the mirror location were required. Taken together, these data show that conserved oculomotor circuits mediate the gap effect in marmosets and Old World primates, and that this species can be trained on more sophisticated tasks. Further, this suggests that the common marmoset is a suitable model for neurophysiological investigations of oculomotor control. 

**Control of fixation durations in a visually guided task **

**Hans A. Trukenbrod & Grenzebach Jan **

University of Potsdam, Germany ****

Hans.Trukenbrod@uni-potsdam.de

Eye movements are a moment-to-moment correlate of cognition. The exact link between fixation durations and underlying cognitive processes, however, remains an open research question. While some theories suggest that specific processing events trigger saccades, other theories suggest weaker links. Here, we investigate the control of fixation durations in a visually guided task, where stimulus n indicates the position of the next stimulus n+1. The task maximizes the chances to find evidence for a direct link between cognitive processing and fixation durations. We manipulated processing demands by modulating visibility of items over time. Presentation of displays was gaze contingent with all but the fixated symbol (Landolt-C) masked by rings without a gap. In two experiments, we observed that fixation durations immediately adjusted to new processing demands. However, large changes in processing demands as well as changes during fixation led to an asymmetry in the control of fixation durations. Fixation durations lengthened immediately when processing demands increased and shortened maximally only on later fixations when processing demands decreased. Our results lend support to theories that suggest a weaker link between fixation durations and cognitive processing. We discuss our results within the framework of a dynamical model.

**Adaptation of post-saccadic drift in reflexive saccades does not transfer to voluntary saccades **

**Giulia Manca**^1,2^**, Heiner Deubel**^1^****

^1^Graduate School of Systemic Neurosciences, Ludwig-Maximilians-Universität München, Munich, Germany; 

^2^Allgemeine Experimentelle Psychologie, Department Psychologie, Ludwig-MaximiliansUniversität München, Munich, Germany manca.giuli@gmail.com 

Post-saccadic eye stabilization is essential for ensuring high visual acuity during fixations. Previous monkey studies demonstrated a cerebellum-dependent adaptive mechanism that is able to minimize post-saccadic drift (PSD) resulting from lesions of the peripheral oculomotor system. It has been proposed that this adaptation occurs by an adjustment of the pulse/step innervation ratio at the level of the brainstem. The present study aimed at investigating plasticity of post-saccadic eye stabilization in healthy humans. In particular, we asked whether PSD adaptation would be specific to the type of saccade for which it was elicited. To answer this question we attempted to induce PSD by consistently presenting a small, exponential onward target drift immediately following reflexive saccades. We analyzed the amount of transfer of the resulting adaptation to interleaved open-loop reflexive and voluntary saccades. 

Our post-saccadic target drifts indeed resulted in the induction of PSD following reflexive saccades. However, voluntary saccades were not affected, suggesting separate adaptive mechanisms for reflexive and voluntary saccades. The results confirm that PSD can be induced by consistent post-saccadic target drifts. They argue against an exclusive brainstem involvement in adaptive control of PSD, as a simple adjustment of the pulse/step ratio would affect both saccade types. 

Thematic session: Applied visual cognition

**Room 3 **(HS 28 - I.13.71) 

**Eye movements during lifeguard visual search for a drowning swimmer **

**Victoria , David Crundall, Christina Howard & Duncan Guest **Nottingham Trent University, United Kingdom ****n0261371@ntu.ac.uk 

The effects of expertise in visual search have been demonstrated in applied domains (driving, CCTV monitoring, airport security screening). However, one domain that remains under researched is that of the lifeguard. This current study aimed to investigate differences in lifeguard and non-lifeguard responses in a visual search task for an active or passive ‘drowning’ swimmer. The study used naturalistic and dynamic stimuli depicting differing number of people swimming in a pool. In an eye tracking study, accuracy and reaction times were measured through push button responses. Whilst there was no difference in accuracy of responses between the levels of experience, on drowning trials lifeguards’ superiority was shown in faster responses than controls. Generally, eye movement data showed that passive drownings were being looked at earlier than active drownings and more often. Response times to passive drownings were faster than active drownings and active drownings showed marginally longer dwell times than passive drownings. This shows that active drownings are potentially not as salient as passive drownings, and require a longer decision process once detected. These results could offer possible insight to training search methods for the different drowning types. ****

**Multiple Object Avoidance (MOA): A more sensitive measure of visual attention in the real world **

**Andrew K. Mackenzie, Paul R. Cox, Christina Howard, Duncan Guest, David Crundall **Nottingham Trent University, United Kingdom ****andrew.mackenzie@ntu.ac.uk 

Visual attention in the ‘real world’ is often more complex than what is represented in standard visual attention assessment tasks. We therefore developed an interactive tracking task called Multiple Object Avoidance (MOA) that may better capture the visual attentional properties involved in complex tasks. The aim was to explore how well attentional function, as measured by the MOA, predicts eye movements and behaviour in driving tasks. In Experiment 1, we found MOA performance predicted both driving performance and effective eye movements (e.g. road scanning) whereas a standard MOT task did not. This may be due to the active nature of the task where the link between vision and action is represented. In Experiment 2, predictive performance of the MOA was compared to a more commonly used FFOV task and incorporated more hazardous situations to establish a link between effective eye movements and safe driving. MOA performance predicted driving and eye movement behaviour, and a correlation between effective eye movements and hazard perception performance was established. We conclude that the MOA task may be a useful tool for assessing visual attention in the real world. We wish to promote this type of task to researchers in other domains of visual attention.

**The (Change) Blindingly Obvious: Investigating Fixation Behaviour during CCTV Observation **

**Gemma Graham**^1^**, James Sauer**^2^**, Jenny Smith**^3^**, Lucy Akehurst**^4^**, James Ost**^4^****

^1^University of Brighton, United Kingdom; 

^2^University of Tasmania, Australia; 

^3^University of Chichester, United Kingdom; 

^4^University of Portsmouth, United Kingdom 

Although CCTV footage is used for both crime prevention and police investigations, relatively little is known about the strategies that observers use when monitoring and interpreting (criminal) events observed in such footage. The complexity of the constantly changing scenes often encountered when viewing CCTV footage highlights an important and applicable context in which real-world, fast moving environments need to be visually understood. Using an applied change blindness task and recording eye movements, a set of experiments explored how task instructions and central and marginal information influence fixation behaviour during CCTV observation. We also investigated whether verbalisation and repeated viewing of CCTV footage would improve change detection rates and influence fixation behaviour. Results demonstrated that change detectors and non-detectors display different fixation behaviours during the observation of CCTV footage. Change detectors fixate more often and for longer on visually important targets (i.e. the criminal) and central information in the footage compared to nondetectors. Repeatedly watching CCTV footage also improved change detection and influenced visual search strategies. We will discuss these findings in relation to both live and post-event CCTV observation and the role of video-based paradigms in applied eye tracking research. 

**Eye during perspective-taking in younger and older adults **

**Victoria E. A. Brunsdon, Elisabeth E. F. Bradford & Heather Ferguson **University of Kent, United Kingdom ****

v.e.a.brunsdon@kent.ac.uk

Healthy adults can rapidly compute their own and another’s perspective, yet they have difficulties when another person’s point of view conflicts with their own. This study investigated how perspective-taking abilities change across the lifespan using eye-tracking to examine the cognitive mechanisms that underlie visual perspective-taking. Younger (18-30 years-old) and older (65-80 years-old) adults completed a version of Samson et al.’s (2010) visual perspective-taking task. Participants’ behavioural responses were complemented by eye movement analysis. The behavioural responses of the younger adults indicated that they are influenced by what they can see when judging another’s perspective (egocentric intrusions) and influenced by what someone else can see when judging their own perspective (altercentric intrusions), replicating previous findings. However, older adults had specific impairments when there was a conflict between their own and another’s perspective. This pattern was also examined in gaze behaviour and pupillometry analysis. We examined the location of participants’ eye movements around the visual scene. There were distinct fixation patterns for self and other perspective-taking that did not differ between the younger and older age groups. Eye movement analyses indicated that both younger and older adults were using similar processing strategies during visual perspective-taking. 

**Using eye-tracking to study how belief-reasoning processes change across the lifespan **

**Elisabeth E. F. Bradford, Victoria E. A. Brunsdon, Heather Ferguson **

University of Kent, United Kingdom

E.E.Bradford@kent.ac.uk 

This study explored how efficiently younger (18-30 years) and older (65-80 years) adults compute beliefstates of the ‘Self’ and ‘Other’. Using a computerised false-belief task, participants were shown a container with expected (e.g., sugar in a sugar jar) or unexpected (e.g., marbles in a sugar jar) contents inside. Following contents revelation, participants heard an audio question asking them to consider what either they themselves (‘Self’) or another person (‘Other’) had believed to be within the container, before seeing inside. Three images then appeared on screen: the correct answer (‘sugar’), a distracter (‘marbles’), and a novel filler item. Eye-tracking analysis revealed that, compared to younger adults, older adults took longer to disengage from the ‘distracter’ object (i.e., the object they know to actually be held in the container), in order to focus on the correct belief-state object, suggesting egocentric processing of the scenario. Behavioural results reflected this: older adults were slower and less accurate than younger adults when attributing beliefs to other people. Results suggest that different strategies are utilized across the lifespan when considering the perspectives of the ‘Self’ versus ‘Other’, and indicate that reductions in the ability to inhibit the knowledgeable egocentric viewpoint may influence social communication skills. 

**An eye-tracking investigation of mindset effects on information search in incentivized decisions under uncertainty **

**Jonas Ludwig, Alexander Jaudas & Anja Achtziger **

Zeppelin Universität Friedrichshafen, Germany jonas.ludwig@zu.de 

An eye-tracking study investigated the effects of deliberative and implemental mindsets on information search and decision making in risky choices. Building on previous work (Rahn, Jaudas, & Achtziger, 2016a), we explored how achievement motivation and self-efficacy were affected by the induction of mindsets, as well as potential effects of monetary incentives in a well established lottery task paradigm adopted from Glöckner and Herbold (2011). In addition to previous applications of that paradigm, a combined yield of all gambles was calculated based on participants‘ choices and determined the monetary compensation for participation in this study. Participants‘ personal yield, and thus, incentive, was directly linked to individual decisions. While main effects of prior studies were replicated, the results suggest that incentivization contingency on decision behavior has notable impact on information search, but little to no effect on choice. Likewise, mindsets affect decision processes, but not choices. These results emphasize a high robustness of choice preferences in economic decision making. However, they also stress that decision processes such as information search, unlike choices, are highly sensitive to variations of motivational states and monetary incentive. 

Thematic session: Reading: Word level processing

**Room 4 **(HS 26 - I.13.65) 

**Raeding transposde etxt: Effects of letter position, word frequency and constraint **

**Christopher James Hand**^1^**, Joanne Ingram**^2^**, Graham Scott**^2^******

^1^Glasgow Caledonian University, United Kingdom; 

^2^University of the West of Scotland christopher.hand@gcu.ac.uk 

We investigated the effects of letter transposition position and Frequency and Word-Initial Letter Constraint (WILC) of the orthographically correct word form on lexical processing via a lexical decision task (LD; n1=40) and an eye movement reading task (n2=50). We hypothesised that External-Beginning transpositions would be most disruptive – in LD, these transpositions would facilitate a ‘non-word’ response (i.e., faster reaction times; RTs), whereas in normal reading, we expected that the less wordlike a target, the more difficult processing would be (i.e., longer fixation durations; FDs). A 2 (Frequency: HF, LF) × 2 (WILC: HC, LC) × 5 (Transposition: Normal, External-Beginning, Internal-Beginning, InternalEnding; External-Ending) design was used. All 120 target words were 5 letters long. Experiment 1 analyses revealed a significant effect of transposition on RTs. External-Beginning transpositions yielded faster RTs than other transposed conditions. Analyses revealed a three-way interaction between Frequency, WILC and transposition. In Experiment 2, target words were placed in single-line sentences, which were contextually neutral. Non-target words were transposed according to target condition. Experiment 2 analyses revealed External-Beginning transpositions yielded longer FDs than other transposed conditions. Analyses revealed interactions between Frequency, WILC and transposition. Implications for models of word identification and of eye movements in reading are discussed. 

**Morphological guidance of eye movements during reading **

**Jukka Hyönä, Seppo Vainio & Timo Heikkilä **

University of Turku, Finland ******hyona@utu.fi 

Yan et al. (2014) showed that in a morphologically rich language (Uighur) the morphological status of a word can influence where in the word the initial fixation lands. The initial landing position was closer to the word beginning when it hosts morphological suffixes, in comparison to a monomorphemic word. Hyönä et al. (in press) replicated the effect in another morphologically rich language, Finnish. A possible explanation for the effect is that readers parafoveally recognize the suffixes and direct the eyes toward the center of word stem, which is shorter in multimorphemic than monomorphemic words when word length is equated. This explanation was tested in a gaze-contingent display change experiment, where for the half of the multimorphemic target words the suffixes were initially replaced with pronounceable letter clusters not constituting morphemes. We replicated the morphological effect in initial landing position: it was closer to the word beginning in suffixed than monomorphemic words. However, the effect remained even when the suffixes in the multimorphemic word conditions were unavailable parafoveally. It is concluded that this effect is not due to readers detecting suffixes at the word end. Instead, it reflects parafoveal access to word stems that are shorter in multimorphemic than monomorphemic words. 

**Morphological processing in sentence reading: Evidence from the fast priming paradigm **

**Betty Mousikou & Sascha Schroeder **

Reading and Development, Max Planck Institute for Human Development, Berlin, Germany ****mousikou@mpib-berlin.mpg.de 

We investigated morphological processing of prefixed and suffixed words in single-word reading using masked priming, and in sentence reading using fast priming in eye tracking. In Exp.1, target words (KIND) were preceded by five types of primes: (1) words comprising the target as a stem and an affix (kindlich); (2) nonwords comprising the target as a stem and an affix (kindhaft); (3) nonwords comprising the target as a stem and a non-affix (kindpern); (4) unrelated words (holzhaft); (5) nonwords comprising a stem and a non-affix (holzpern). In Exp.2, target words (kindlich) were masked by random letter strings until the eyes crossed an invisible boundary located before the target. At boundary crossing, the mask was replaced by a briefly-presented prime (kindpern) before the target appeared. Results from both experiments indicated embedded stem activation for both suffixed and prefixed words, independently of the presence of an affix in the prime. The eye movement data further revealed that this effect was driven by the duration of the first fixation on the target and by differences in the probability to refixate it, thus indicating embedded stem processing during sentence reading. We interpret our findings within extant models of morphological processing and eye movement control. 

**Distributional analyses of age of acquisition effects on fixation durations during reading **

**Heather Sheridan**^1^**, Barbara J. Juhasz**^2^****

^1^Department of Psychology, University at Albany, State University of New York, Albany, NY, United States of America; 

^2^Department of Psychology, Wesleyan University, Middletown, CT, United States of America hsheridan@albany.edu 

The study examined the time course of age of acquisition (AoA) effects on fixation durations during reading. Participants’ eye movements were monitored in an experiment that manipulated the age-of-acquisition of target words (early vs. late), which were matched across conditions for a variety of variables including word frequency, imageability, familiarity, length and OLD-20. Mean fixation durations were significantly longer in the late than the early condition, and distributional analyses revealed that this AoA effect had a rapid impact on distributions of first-fixation durations. Specifically, survival analyses revealed that the earliest discernible effect of AoA on the distributions emerged at 158 ms from the start of fixation. In addition, Vincentile plots showed that AoA effects were relatively constant in magnitude across the distribution, indicating that both short and long fixations were impacted by the manipulation. These results are consistent with prior findings that a wide range of lexical variables have a fast-acting effect on distributions of fixation durations during reading, including word frequency, lexical ambiguity and predictability. Implications for models of eye-movement control are discussed.

**Eye movements during lexical access of a third language **

**Pâmela Freitas Pereira Toassi**^1^**, Mailce B. Mota**^2^**, Elisângela N. Teixeira**^1^

^1^Federal Universtiy of Ceara, Brazil; 

^2^Federal University of Santa Catarina, Brazil pam.toassi@gmail.com 

Investigating lexical access of trilingual speakers may give hints regarding the organization and processing of multiple languages. Among the factors that may interfere with lexical access of bilingual and multilingual speakers, it can be mentioned the similarity among the languages and the linguistic knowledge of the interlocutor (De Bot & Jaensch, 2015). We conducted an eye tracking study to investigate the effect of triple cognates in the lexical access of speakers of English (L3), German (L2), and Brazilian Portuguese (L1). The participants performed a sentence comprehension task, containing 60 experimental sentences with the following critical words: triple cognates, double cognates between Brazilian Portuguese and English, and double cognates between German and English. The first fixation and the first and second reading pass times were analyzed. The results suggested that triple cognates were processed faster than their respective controls in first fixation (M: 264/311ms (cognate/control); p=0,03) and first pass (M: 407/448ms (cognate/control); p=0,05). We conclude that our results could contribute to the literature of lexical access of multilinguals, favoring the view that all the languages of a multilingual are active even when the speaker intends to use only one language. 

**Learning new words when reading: effects of contextual diversity and temporal spacing **

**Ascensión Pagán & Kate Nation**

University of Oxford, United Kingdom ****ascension.pagancamacho@psy.ox.ac.uk 

We examined whether contextual diversity and spacing during reading experience influence new word learning as adults read sentences silently. Eye movements were recorded as adults read new words embedded in either neutral (testing phase) or meaningful sentences (exposure phase). Words were presented either in the same sentence repeated four times (low diversity) or in four different sentences (high diversity). Spacing was manipulated by presenting the sentences in a distributed or nondistributed episodes. During the exposure phase, words experienced in low diversity contexts had shorter fixation and reading times than words in experienced in varying contexts. Similarly, words experienced in a non-distributed manner received shorter fixation and reading times than words seen in distributed contexts. At test, fixation times on the new words were reduced relative to baseline for both early and late measures of processing. The interaction between diversity and spacing was significant for total time, such that words experienced in the low diversity condition and in a nondistributed manner resulted in longer total times compared to words experienced in varying and distributed contexts. These findings suggest that diversity and spacing promote word learning. 

Thursday, August 24^th^, 14.30 - 16.30 

Symposium: Yarbus, eye movements and vision 50 years on

**Room 1 (HS 14 – M.10.12) **

**Yarbus on stationary retinal images and moving eyes**

**Nicholas Wade **

University of Dundee, United Kingdom

n.j.wade@dundee.ac.uk****

It is an of history that the impetus for examining how the eyes move derived from experiments that kept stimuli stationary on the retina. Nowhere is this more apparent than in Yarbus’s 'Eye Movements and Vision'. The seven chapters were concerned with methods, perception of retinal stationary objects, eye movements during fixation, saccades, scanning, pursuit, and eye movements with complex objects. Yarbus brought a new dimension of precision to recording how the eyes moved, either when attempts were made to keep them stationary or when scanning pictures. The most venerable technique for examining ocular stability involved comparing relative motion between an afterimage and a real image. This was applied initially for studying post-rotational nystagmus and ocular instability when attempting to fixate steadily. Photographic records of eye movements during reading stimulated research using pictures. Attention shifted back to the stability of the eyes during fixation, with the emphasis on involuntary movements. The contact lens methods developed by Yarbus were initially applied to recording the perceptual effects of retinal image stabilization. The major impact of Eye Movements and Vision is now seen as demonstrating the influence of instructions on scanning eye movements.****

**The evolution of gaze analysis tools to support complex tasks**

**Jeff B. Pelz**

Rochester Institute of Technology, United States of America**** pelz@cis.rit.edu****

Yarbus’ classic experiments, described in Chapter VII of Eye Movements and Vision (and earlier in Eye Movements on Looking at Complex Objects [Biofizika 6: No.2, 207-212, 1961]), were powerful in part because of the simplicity of the data analysis. In the earlier paper, Yarbus briefly outlined his conclusions that fixation patterns were dependent both on the task and on the information available in different image regions. Analysis, however, was left to the readers’ intuition. The single page of text ended with the sentence, “As an illustration of these remarks we give some records of movements of the eyes and invite the reader himself on the basis of figures to judge the claims of the author.” [P 229] The four pages of figures indeed provided powerful support for Yarbus’ claims, and have been an inspiration for many in the intervening decades. While the development of instrumentation has supported gaze recording during more complex and interactive tasks, data analysis tools have not kept up, requiring laborious, and perhaps error-prone manual analysis methods. Recent advances in machine learning and computer vision, however, have provided the basis for new analysis tools that show great promise.****

**Computational modeling of gaze guidance during scene free viewing and daily tasks **

**Ali Borji**

University of Florida, United States of America aborji@crcv.ucf.edu 

In the past two decades, much progress has been made in developing theories and computational models for exogenous guidance of attention towards salient stimuli. Despite a large body of research, there still exists a gap between the accuracy of current models and the human inter-observer in predicting fixations during scene free viewing, visual search and daily tasks. In this talk, I intend to give a snapshot of biological findings on visual attention, theoretical background on saliency concepts and models, illustrating successful applications of saliency models, as well as my research findings in mind state decoding from eye movements. I will cover three topics in more detail: 1) bottom-up, stimulusdriven attention, 2) top-down, task-driven attention, and 3) task decoding from eye movements effectively replicating Yarbus' original experiment. In each case, I will first present some psychological studies followed by some computational models. Concentration will be on computational modeling, current state in eye movement prediction, model benchmarking, and future directions towards reaching human level accuracy in fixation prediction.

**Eye guidance in scenes: Object-based selection in extrafoveal vision**

**Antje Nuthmann**

School of Philosophy, Psychology and Language Sciences, University of Edinburgh, United Kingdom Antje.Nuthmann@ed.ac.uk 

Based on his seminal case studies, Yarbus assumed some form of object-based attentional selection in naturalistic scenes – in stark contrast to today’s predominance of image-based saliency models of visual attention. I begin by presenting evidence for an alternative role for image salience: rather than prioritizing locations, salience aids object prioritization. I go on to present an analysis framework that allows us to assess the independent contributions of low- and high-level object- and image-based variables to early and late measures of extrafoveal attentional selection in scenes. Object-scene congruence was experimentally manipulated, while statistically controlling for other variables in images of real-world scenes. The very first saccade was guided by object size and object salience, but not by global image clutter or object-scene semantics. Importantly, as scene processing went on, the eyes took less time to travel to incongruent than congruent objects, providing evidence for the much-disputed inconsistent object advantage. By-item random effects in linear mixed models suggested that scene items varied in the extent to which they showed the effect. Fifty years after Yarbus’s classic work, we now have better analytical tools to systematically investigate the control of attention and fixation in scenes.

**Characterising top-down guidance of fixation in scenes and objects** **Tom Foulsham**

University of Essex, United Kingdom foulsham@essex.ac.uk 

Fixations on complex images are “determined by the nature of the object and the problem facing the observer at the moment of perception” (Yarbus, 1967, p. 196). Despite this simple statement, characterising the different sorts of top-down processing which affect eye guidance remains a challenge. I will describe three recent findings showing how the knowledge and actions required in particular realworld tasks affect fixation placement. First, when searching for objects in pictures, knowledge about likely object location has an immediate effect on where people look. Prior expectations of object position provide a quantitative measure of knowledge and how it is combined with visual input over multiple fixations. Second, there are reliable differences between gaze when walking through a real environment and fixations on an image of the same environment. These indicate the richness of the implicit tasks being carried out, as well as knowledge of the structure of the world. Third, with real objects, participants’ fixation placement depends on action and affordance. I will discuss how to represent the “problem facing the observer” in each of these tasks, as well as their prior information, in order to predict top-down attention. 

**The balance between the stimulus and the task in determining the scanpath**

**Iain Gilchrist**

University of Bristol, United Kingdom

i.d.gilchrist@bristol.ac.uk

In his now famous experiment, Yarbus gave participants different instructions while viewing Ilya Repin’s picture ‘They did not expect to see him’. What Yarbus showed was that the scanpaths while participants viewed the painting depended on the viewing instructions. This remains a clear and striking demonstration that fixation behaviour is not solely determined by the stimulus characteristics but rather is, in part, shaped by the task of the participant. A central research question that stems directly for this demonstration is: what is the balance between stimulus characteristics and the task in determining fixation behaviour? I will review some of our own work and that of others to illustrate that we are still a long way from being able to answer this question, and illustrate that some of the approached adopted in this field are not logically able to distinguish between these two possible determinates. I will conclude with the disturbing suggestion that this question may not be experimentally tractable. 

Thematic session: Clinical Research II

**Room 2 (HS 32 - K.11.23) **

**Processing of Co-Reference in Autism Spectrum Disorder **

**Philippa L Howard**^1,2^**, Simon P Liversedge**^2^**, Valerie Benson**^2^

^1^Southampton Solent University, United Kingdom; 

^2^University of Southampton, United Kingdom****philippa.howard@solent.ac.uk****

Individuals autism spectrum disorder (ASD) are often reported to have reduced performance accuracy for reading comprehension, relative to typically developing (TD) controls. This study examined whether reduced comprehension accuracy may be underpinned by differences in the efficiency with which co-referential links are formed. Adults with ASD and TD controls read mini discourses comprised of two sentences, as their eye movements were monitored. The second sentence contained a category noun (e.g., bird) that was preceded by and co-referred to either a typical (e.g., pigeon) or atypical (e.g., penguin) instance of the category. An effect of typicality was found for gaze duration upon the category noun, with longer times being observed when the instance was atypical, in comparison to typical. No group differences or interactions were detected for target processing, and verbal language proficiency was found to predict general reading efficiency and referential processing skill. However, individuals with ASD were more likely to re-read the text, in comparison to TD controls. These data suggest that readers with ASD do not differ in the efficiency with which they compute anaphoric links on-line during reading, but readers with ASD may adopt a qualitatively different reading strategy to TD controls. 

**How does the presence or absence of a Title Modulate Processing of Ambiguous Passages in Individuals with Autism: An Eye Movement Study**

**Valerie Benson**^1^**, Philippa Howard**^1^**, Johanna Kaakinen**^2 ^

^1^University of Southampton, United Kingdom; 

^2^University of Turku, Finland vb1@soton.ac.uk 

Weak Central Coherence (WCC) theory proposes that individuals with Autism Spectrum Disorders (ASD) fail to use contextual information to facilitate their global processing and understanding of ambiguous text. This study investigated behavioural and eye movement measures of typically developing (TD) and ASD adult participants when reading ambiguous passages of text with and without titles. Individuals with ASD showed no differences in comprehension accuracy, gaze duration on target words, or total time spent fixating target words for passages presented with or without a title, indicating that the presence of titles did not facilitate processing, at least at the lexical level, in the ASD group. There was however a difference in total time on target words for TD readers between the titles and no titles conditions. These results suggest that ASD individuals fail to use the contextual information provided by a title to facilitate their reading of ambiguous passages, and provide some support for WCC theory. 

**Inhibitory control for emotional and neutral scenes in competition: An eye-tracking study in bipolar disorder**

**Manuel Perea**^1^**, Ladislao Salmerón**^1^**, Ana García-Blanco**^1,2^

^1^Universitat de València, Valencia, Spain; 

^2^Health Research Institute La Fe, Valencia, Spain mperea@uv.es 

The present study examined the inhibitory control of attention to social scenes in manic, depressive, and euthymic episodes of Bipolar Disorder. Two scenes were simultaneously presented (happy/threatening [emotional] vs. neutral). Participants were asked either to look at the emotional pictures (i.e. attend-to-emotional block) or to avoid looking at the emotional pictures (i.e. attend-toneutral block) while their eye movements were recorded. The initial orienting (first-fixation) and subsequent attentional engagement (first-pass fixation duration) were computed. In the attend-toemotional block, attention was captured equally by the two emotional images. In the attend-to-neutral block, whereas manic patients showed a higher number of initial fixations for happy scenes, their firstpass fixation duration was longer for threatening scenes. Inhibitory control was not modulated by the scene emotional salience in the other groups. Thus, manic patients had difficulties voluntarily ignoring emotional information, which was characterized by a happy-related bias during initial orienting, but a threat-related bias during attentional engagement. 

**Smooth in Adults with Developmental Dyslexia**

**Gillian O'Driscoll**^1^**, Veronica Whitford**^2^**, Ashley Chau-Morris**^1^**, Debra Titone**^1 ^

^1^McGill University, Canada; 

^2^Harvard University gillian.odriscoll@mcgill.ca 

Schizophrenia and dyslexia both involve deficits in language processes and saccade control. A common neurodevelopmental basis for these disorders has been suggested by genetic and pathophysiological overlap. Abnormal smooth pursuit eye movements are a marker of risk for schizophrenia. Studies of pursuit maintenance in dyslexia have been few. Here, we report the first study of pursuit maintenance in adults with dyslexia. METHODS: 17 adults with dyslexia and 12 matched controls tracked a target moving with a sinusoidal velocity profile at 0.4Hz across 20 degrees. Eye movements were recorded with an Eyelink 2. RESULTS: There was a significant Group by Direction interaction on pursuit gain, with dyslexia participants having significantly lower gain to the left than controls, and a trend to make more saccades when pursuing to the left than controls. The amplitude of saccades during pursuit were larger in dyslexia (p=.036) driven by more anticipatory saccades (>3 degrees) and larger amplitude squarewave jerks. CONCLUSION: Adults with dyslexia showed impairments in pursuit gain that implicate left hemisphere pursuit structures. Bilateral deficits in saccade control were also apparent, with large amplitude intrusive saccades superimposed on pursuit. Our data provide some support for a pathophysiological overlap between dyslexia and schizophrenia, but some differences too in specific impairments.

**Visual field diagnostics with eye tracking: development and neuropsychological testing of a new diagnostic tool**

**Michael Christian Leitner, Constanze Haslacher, Stefan Hawelka, Lorenzo Vignali, Sarah Schuster, Florian Hutzler**

Centre Cognitive Neuroscience, Universität Salzburg, Austria 

Whether visual restitution trainings improve visual field loss after stroke or traumatic brain injury is – as yet – uncertain. Several studies reported evidence for neuroplasticity in the visual cortex, while other studies suggest that these findings reflect methodological shortcomings rather than actual improvement. Among these shortcomings are inadequate fixation control and susceptibility to compensation strategies. We developed a new paradigm for visual field diagnosis which incorporates a stringent fixation control and adaptive stimulus presentation (see Fig. 1 in the SI). These features provide superior accuracy and markedly reduce the susceptibility to compensation strategies. In a first step, we diagnosed the blind spot in normally sighted participants (repeatedly) in order to assess the accuracy and reliability of the paradigm (see Fig. 2 in the SI). Currently we compare our new paradigm with established diagnostic tools in order to assess its validity. Ultimately, the tool will be implemented on VR-hardware and will then be used to evaluate visual restitution trainings and the issue of the potential plasticity of the human visual cortex. 

**Calibrating an eye tracker for blind patients implanted with the Argus II retinal prosthesis using a handheld marker**

**Avi Caspi**^1,2^**, Jessy D. Dorn**^2^**, Arup Roy**^2^**, Robert J. Greenberg**^2^****

^1^Jerusalem College of Technology, Jerusalem, Israel; 

^2^Second Sight Medical Products, Inc., Sylmar, California, USA 

Blind can gain some useful sight by electrical stimulation of the retina. The Argus® II visual prosthesis has been implanted in more than 200 blind individuals worldwide. In the Argus II, the camera is mounted on the glasses and eye movements do not affect the implant’s visual information, though the location of the percepts depends on eye position. Users are instructed to keep their eyes aligned with their head while scanning with head movements. 

Integrating an eye tracker in the visual prosthesis enables scanning using eye movements. Eye position can set the region of interest within a wide head mounted camera. However, traditional eye tracker calibration methods requiring looking at points in space and cannot be used with blind people. In the presented research, epi-retinal electrodes were directly stimulated and patients reported the precept’s location by placing a trackable handheld marker in space. The correlation of pupil location at the onset of the stimulation with the head-centered percept location was used to calibrate and align the eye tracker on Argus II users. Our experimental results show that integrating a calibrated eye tracker reduces the amount of head motion and improves visual stability in Argus II users. 

Thematic session: Eye data analysis and evaluation

**Room 3 (HS 28 - I.13.71) **

**SMAC with HMM: a toolbox to model and classify scanpaths with Hidden Markov Models**

**Antoine Coutrot**

University College London, United Kingdom 

a.coutrot@ucl.ac.uk 

How look at visual scenes contains fundamental information about them and their state of mind; like the task at hand, their level of expertise, their mental workload, their personality or even their state of health. Eye movements are highly dynamic, complex signals, which makes the information they convey hard to capture. Here, we provide a turnkey method for data-driven scanpath modelling and classification. This method relies on variational Hidden Markov Models (HMMs) and Discriminant Analysis (DA). HMMs capture the dynamic and idiosyncratic dimensions of gaze behaviour, allowing DA to capture systematic patterns diagnostic of a given class of observers and/or stimuli. We validate our method on different datasets available online. First, we use fixations recorded while viewing 800 static images, and infer an observer-related characteristic: the task at hand. Second, we use eye positions recorded while viewing 15 videos of people having a conversation, and infer a stimulus-related characteristic: the presence or absence of original soundtrack. This synergistic approach between eyetracking and machine learning will open new avenues for simple quantification of gaze behaviour. We release SMAC with HMM, a Matlab toolbox freely available to the community under an open-source license agreement. 

**Gaze Self-Similarity Plots as a useful tool for eye movement characteristics analysis**

**Pawel Kasprowski, Katarzyna Harezlak**

Silesian University of Technology, Poland kasprowski@polsl.pl 

Eye tracking has become an important way to analyze human behavior. However, a proper analysis of data obtained from an eye tracker is a challenging task. Traditional visualization techniques such as scanpaths or heat maps may reveal interesting information, nonetheless much of useful information is still not visible, especially when the temporal characteristics of eye movement is taken into account. This presentation introduces a technique called gaze self-similarity plot (GSSP) that may be applied to visualize both spatial and temporal eye movement features on the one two dimensional plot. The technique is an extension of the idea of recurrence plots, commonly used in time series analysis. The main advantages of the GSSP are that it does not depend on any adjustable parameters or thresholds which have to be tuned and it is very easy to plot. It may visually reveal many eye movement properties like outliers, ambient/focal processing, search strategy, recurrence and so on. Moreover, GSSP may be used to calculate different gaze pattern related metrics. The basic concepts of the proposed approach (three types of GSSP) complemented with some examples explaining what kind of information may be disclosed and areas of the GSSP applications will be presented. 

**Towards to an authentication method based on eye movement by using scanpath comparison algorithms**

**Carlos-Alberto Quintana-Nevárez, Francisco López-Orozco**

LabTEC2, División Multidisciplinaria de la UACJ en Ciudad Universitaria, Cd. Juárez, Chihuahua, México. al131608@alumnos.uacj.mx

Nowadays, automatic authentication is still an important issue. This paper presents a relatively new approach towards a construction of a secure method to authenticate people by using their eye movements. Our method is based on a simple scanpath comparison. Ten volunteers were asked to evaluate our proposal. People’s eye movements were recorded by using an eyetracker when they were drawing a numeric personal identification number (PIN) in a screen numeric pad. Scanpaths were compared using the simple linear correlation algorithm proposed by Liu & Gao, et. al. (2015). We also used an eye analysis which consists of measuring the similarity of scanpaths by calculating and normalizing the distance in pixels for each point in the scanpaths proposed by Mathot & Cristino, et. al. (2012). Preliminary results are promising. We got an average acceptance rate of 70% with our second approach and a low false acceptance rate under 25%. However, the study should be continue in order to generalize our results toward the construction of a complete method to be follow as an automatic authentication approach. 

**Magnitude Nature of Variability in Eye-tracking Data**

**Kenneth Holmqvist**^1,2^**, Raimondas Zemblys**^3^**, Tanya Beelders**^4^

^1^Lund, Sweden; 

^2^UPSET, North-West University (Vaal Triangle Campus), South Africa; 

^3^Siauliai University, Lithuania; 

^4^Universiteit van die Vrystaat, Bloemfontein, South Africa kenneth.holmqvist@ownit.nu

Existing precision measures do not adequately describe the magnitude of variability (e.g. noise) in eyemovement data, because they are also affected by the nature of the variability in the signal. For instance, sample-to-sample RMS of an “ant trailing” signal would be low, indicating good precision, while the signal could spread over a large spatial extent. Conversely, for sawtooth signal artifacts that are for instance produced by a spurious corneal reflection, the spatial standard deviation (STD) can be small while RMS can be very large. We have developed two new complementary measures of variability that unambiguously indicate the magnitude of variability independent of its nature, and orthogonally the nature of the variability independent of its magnitude. We hypothesize that the nature of the variability is a constant property of an eye-tracker, while the magnitude varies with many factors specific to the situation when data are recorded (such as gaze position on the screen and pupil size). Data quality studies benefit from such a distinction. Our measure could further be employed to test how robust event detection algorithms are to increases in the magnitude of variability in the eye-movement signal, and which algorithms are most suitable for which type of signal variability. 

**Effects of Task on Eye Movements During Comprehension of Abstract Data Visualizations**

**Laura Matzen, Kristin Divis, Michael Haass**

Sandia Laboratories, United States of America lematze@sandia.gov 

Data visualizations are widely used to convey information, but it is difficult to evaluate their utility. Developing a better understanding of how the user’s task influences their path through an abstract representation of data would help visualization designers to assess the effectiveness of their designs from the perspective of human cognition. In this study, eye movement data was recorded from participants who completed different tasks using scatter plots. We investigated the influence of the viewer’s task on their attention to different regions of interest. In one set of tasks, participants viewed scatter plots depicting a trend and outliers. They were asked to describe the trend for half of the stimuli and the outliers for half of the stimuli (counterbalanced across participants). In the second set of tasks, participants viewed scatter plots containing two clusters. They were asked to indicate which cluster had the highest average value or to determine the cluster membership of a reference point. The results showed that the task had a substantial influence on the participants’ patterns of eye movements. This mirrors findings for natural scenes (c.f. Mills et al., 2011) and lays the foundation for developing new heuristics for evaluating the design of abstract data visualizations. 

Thematic session: Reading: Across the lifespan

**Room 4 (HS 26 - I.13.65) **

**Syllables vs. morphemes in early reading of Finnish **

**Tuomo Häikiö, Seppo Vainio**

University of , Finland****tuilha@utu.fi

To facilitate the use of syllables, syllable boundaries are signaled by hyphens (e.g., ta-lo, house) in early Finnish reading instruction. However, hyphenation is detrimental to reading speed by virtue of dividing words into smaller units than preferred by young readers (Häikiö et al., 2015, 2016). As readers become more skilled, they start utilizing larger units such as morphemes. Since Finnish is an agglutinative language with rich morphology, morphemes may be more important than syllables even for early readers. To assess this, 7-9-year-old Finnish children read sentences with embedded inflected target words while their eye movements were registered. The target words were either in essive or inessive/adessive (i.e., locative) case. The target words were either non-hyphenated, or had legal or illegal syllabic hyphenation. In Finnish, the syllable and morpheme boundaries overlap for essives but not for locatives. This was utilized to disentangle syllables from morphemes; for locatives, illegal hyphenation was congruent with the morpheme boundary whereas for essives it was incongruent. In gaze duration, there was a hyphenation by case interaction. Illegal hyphenation did not affect the locatives even though it slowed down reading the essives. We interpret this finding as early Finnish readers processing words via morphemes rather than syllables. 

**Words from the wizarding world: Reading fictional words in supportive and nonsupportive contexts **

**Joanne Ingram**^1^**, Christopher James Hand**^2 ^

^1^University of the West of Scotland, United Kingdom; 

^2^Glasgow Caledonian University joanne.ingram@uws.ac.uk 

This study examined the reading of fictional words from the Harry Potter (HP) series of books and movies to establish if, when presented in a supporting context, participants familiar with HP read these words in a similar manner to standard words. Words from this series, representing concepts that do not exist outside related publications (e.g., muggle), were presented to readers in addition to high and low frequency words, in supportive or non-supportive contexts. Participants’ eye movements were recorded as they read two sentence passages: the initial sentence contained contextual information; the second sentence contained the target word. Words from HP could either be familiar or novel to participants dependent on their level of engagement with the HP series. Results showed significant typical main effects of frequency. High frequency words were processed faster than low frequency words. Processing of HP words was facilitated by a supportive context for those who had engaged with the series. Results suggests that those familiar with HP have integrated these words into their lexicon to the extent that they are read as easily as low frequency words when supported by context. Future investigation of words from fiction may wish to examine familiarity in addition to frequency. 

**Re-Assessing Adult Age Differences in the Perceptual Span during Reading**

**Kevin Paterson, Kayleigh Warrington, Sarah White, Victoria McGowan ** University of Leicester, United Kingdom kbp3@le.ac.uk 

Readers acquire linguistic information from a narrow region of text on each fixational pause (called the perceptual span). The perceptual span also appears to change with age so that, compared to young adults, older adults (aged 65+ years) acquire linguistic information from a narrower and more symmetrical area on each fixation. This change in the perceptual span with age could be an important component of the greater reading difficulty that older adults experience. However, recent findings suggest that adult age differences in the perceptual span may be over-stated. Accordingly, we conducted two experiments to more comprehensively examine the perceptual span of young and older adult readers. In these experiments, sentences were shown normally or in a gaze-contingent paradigm in which text was normal within windows that extended either symmetrically (Experiment 1) or asymmetrically (Experiment 2) around fixation and letters in words outside these windows were replaced by visually-similar letters. In addition, spaces between words were either retained or filled by a letter in Experiment 1. No age differences in the perceptual span were observed. We argue that these and related findings suggest that adult age changes in the perceptual span may not be an important component of age-related reading difficulty.

**Adult Age Differences in Chinese Reading: Effects of Character Complexity**

**Jingxin Wang**^1^**, Lin Li**^1^**, Sha Li**^1^**, Yingying Zhang**^1^**, Kevin Paterson**^2^

^1^Academy of Psychology and Behavior, Tianjin Normal University, Tianjin, China;

^2^College of Medicine, Biological Sciences and Psychology, University of Leicester, Leicester, UK wjxpsy@126.com 

Older adults produce characteristic patterns of age-related reading difficulty for both alphabetic and non-alphabetic languages. But while this reading difficulty is attributable tovisual and cognitive declines in older age, itsprecise nature is yet to be fully understood.With the present research, we focused on the role of visual complexity, which is a likely source of reading difficulty (Wang, He, &Legge, 2014). Chinese is ideally suited to investigating such effects, as Chinese characters are formed from differing numbers of individual brush-strokes but always occupy the same square area of space, and so effects of visual complexity are not confounded with word length.In our experiment, young (18-28 years) and older (65+ years) adults read text containing interchangeable high-complexity (>9 strokes) or lowcomplexity (<=7 strokes)two-characters words matched for lexical frequency and predictability. Typical patterns of age-related reading difficulty were observed. But, in addition, an effect of visual complexity in first-pass fixation probabilities and gaze durations for the target words was greater for the older adults. The indication, therefore, is that the older readers have particular difficulty processing words that contain more visually-complex Chinese characters. We discuss these findings in relation to the specific visual demands of Chinese reading. 

**Aging and the Misperception of Words during Reading**

**Kayleigh L. Warrington, Sarah J. White, Victoria A. McGowan, Kevin B. Paterson ** University of Leicester, United Kingdom 

klw53@le.ac.uk 

Research with lexical neighbors (words that differ by a single letter while the number and order of letters is preserved, e.g., stork & story) indicates that readers frequently misperceive a word as its higher frequency neighbor (HFN) even during normal reading (Slattery, 2009). Previous research has not examined age differences in this neighbor frequency effect but if older readers make riskier decisions about the identities of words (Rayner et al., 2006) they may be more susceptible to such effects, especially when the neighbor word is consistent with prior sentence context. Two experiments addressed this issue. In both, young and older adults read sentences containing target words with and without a HFN, where the HFN was congruent with prior sentence context or not. Further, Experiment 2 considered only visually-similar neighbours (e.g., branch & brunch). Consistent with previous findings for young adults, eye movements were disrupted more for words with than without an HFN when the HFN was congruent with prior context. However, age differences in this effect were found only in Experiment 2, when target words and HFNs were visually as well as orthographically similar. We discuss these findings in relation to the nature of word misperception effects in older age.

## Posters

Session I - , August 21^st^, 15.30 - 17:00 

Attention and visual information processing

**Gaze-contingent stimulus removal leads to subsequent changes in attentional allocation **

**Karin Ludwig, Doris Schmid, Thomas Schenk **

Clinical Neuropsychology, Department of Psychology, Ludwig-Maximilians-Universität München, Germany karin.ludwig@psy.lmu.de 

According to the premotor theory of attention, brain circuits that prepare and control (eye) movements also serve to shift or maintain spatial attention. The aim of this study was to determine whether reducing eye movements to one visual hemifield over the course of several hundreds of trials led to a subsequent decrease in deployment of attention to this hemifield. The participants carried out a visual feature search during which the stimuli in the left visual field were removed whenever the participants made eye movements to the left. Indeed, this led to a steady decrease in left-sided fixations over the course of the intervention. The performance in four spatial attention paradigms was measured before and after this intervention. In two visual search paradigms (feature and conjunction search) the proportion of leftsided fixations significantly decreased from pre to post measurement, which was also true for the first fixation. In a Posner task with exogenous cues, a partial effect of the intervention was found. Performance in a line bisection paradigm was not significantly influenced by the intervention. To conclude, transfer effects of the gaze-contingent removal of left-sided stimuli were found in three out of four spatial attention paradigms. 

**The relationship between subjective time perception and visual attention **

**Maria Konstantinova**^1,2^**, Leonid Tereshchenko**^1^**, Viktor Anisimov**^1^**, Alexander Latanov**^1^****

^1^ Lomonosov Moscow State University, Russian Federation; 

^2^Neurodatalab, Russian Federation konstantinova@neurobiology.ru 

The aim of this study was to investigate whether there is a relationship between subjective time perception and visual attention. We have studied the engagement of focal and ambient vision during visual tasks performance. Our participants were athletes with different skill level. They performed Go/No go task and Go/No go task with stimuli relevance change after signal. After the task performance athletes gave self-report about time perception. We analyzed the averaged fixation duration (FD) and saccade amplitude (SA) in different conditions of subjective time perception (subjective time longer than physical time – “long condition - LC” and subjective time shorter than physical time – “short condition - SC”). We used ANOVA and Student’s t-test. Subjective time perception coupled with attention processes (ANOVA for FD F2,5863=14.264, p<0.01; for SA F2,6064=46.175, p<0.01). “LC” is accompanied by greater engagement of focal vision than ambient vision in comparison with “SC” in both groups of athletes. FD in “LC” is significantly longer than in “SC” - 402.1±5.6 vs. 379.5±4.1 ms respectively (t=3.28, df=3748, p<0.01). SA is significantly shorter in “LC” than in “SC” - 19.1±0.2 vs. 22.4±0.2 deg. respectively (t=-10.39, df=4602, p<0.01). Thus longer subjective time perception accompanied by greater engagement of focal vision than ambient vision.

**Rapid top-down and bottom-up auditory attention as reflected by **

**(micro-)saccadic inhibition**

**Andreas **^1^**, Alexandra Bendixen**^2^**, Susann Duwe**^1^**, Ralf Engbert**^3^**, Erich Schröger**^1^**, Nicole Wetzel1 **

^1^University of Leipzig, Germany; 

^2^Chemnitz University of Technology, Germany; 

^3^University of Potsdam, Germany widmann@uni-leipzig.de 

Rare target sounds embedded in a stream of frequent non-target and rare distractor sounds elicit a sustained inhibition of microsaccades, indicating fast discrimination of target sounds. We previously suggested that microsaccadic inhibition reflects the top-down allocation of auditory attention towards targets. Here we aimed at testing this hypothesis in two combined EEG-eyetracking experiments. In an auditory distraction paradigm designed to concurrently manipulate top-down and bottom-up auditory attention, we replicated our finding of sustained microsaccadic inhibition in response to target sounds. Moreover, we additionally observed a transient inhibition of microsaccades in response to distractor sounds. The onset of this response was 125 ms earlier than the corresponding event-related potential (ERP) component P3a that is assumed to reflect bottom-up orienting of attention. In a second study, we measured saccade rates in response to task-irrelevant sounds that were either frequent (standards) or rare (novels). Again, we observed a transient inhibition of saccades selectively in response to novels as early as 50 ms after sound onset and preceding the P3a by 125 ms. We conclude that sustained and transient (micro-)saccadic inhibition reflects the allocation of auditory attention and that attentional orienting is faster than assumed previously on the basis of ERPs. 

**Pre-saccadic remapping of foveal attention **

**Meng Fei Ngan**^1^**, Luca Wollenberg**^1,2^**, Heiner Deubel**^1^**, Martin Szinte**^1^****

^1^Allgemeine Experimentelle Psychologie, Department Psychologie, Ludwig-MaximiliansUniversität München, Munich, Germany; 

^2^Graduate School of Systemic Neurosciences, Ludwig-Maximilians-Universität München, Munich, Germany. 

bethngan92@gmail.com 

Across saccades, the retinal projections of the visual scene shift with the eyes. To counteract this shift, neuronal receptive fields in oculomotor areas are remapped before the saccade. We recently demonstrated a behavioral correlate of this phenomenon showing that pre-saccadic sensitivity is enhanced at the remapped location of an attended target, opposite to the direction of the saccade. In the present study we asked whether attention to a cue appearing at fixation would also be remapped before the saccade. Participants saccaded either towards one of two peripheral exogenous cues, or towards one of two peripheral colored targets matching with a color cue at fixation. Pre-saccadic visual sensitivity was determined using oriented Gabor stimuli presented either at the cued locations, in the opposite direction of the cues, or at several other equidistant locations. As expected, highest sensitivity was found at the saccade target. Importantly, however, sensitivity was also enhanced at the location opposite to the saccade target, but only when participants prepared an eye movement instructed by the attended foveal color cue. This demonstrates that the remapping of foveal attention occurs if participants attend to the fixation target in order to solve the saccade task. 

**Saccade deviation and saccadic reaction time: What is the relationship?**

**Luke Tudge**^1,2,3^**, Torsten Schubert**^3^****

^1^Humboldt-Universität zu Berlin, Germany; 

^2^Berlin School of Mind and Brain; 

^3^Martin-Luther-Universität -Wittenberg luke.tudge@hu-berlin.de 

The trajectories of saccadic eye movements can reveal the impact of an irrelevant distractor on visual attention and selection. The impact of distractors on saccade trajectories is approximately negatively related to saccadic reaction time; early saccades tend to be directed towards a distractor, whereas later saccades tend to be directed away from it. In the present study, we asked how best to describe this function, for example as linear, quadratic, or as a composite of different functions. This question is important theoretically, in order to test conjectures about the underlying processes that generate saccade trajectories and reaction times, but we focus on its equally important methodological impact. Due to the increasingly popularity of mixed effects models, it is often necessary for researchers studying saccade trajectories to select a suitable random effects model of the relationship between saccade trajectory and saccadic reaction time. Using a large data set from several previous experiments, we applied cross-validation methods to test the fit of several plausible models, and also report the effects that using these different models can have on both the computational tractability of the analyses and the substantive conclusions drawn. 

**Can you squint on command? **

No reliable voluntary control and awareness of eye vergence in the absence of an actual target

**Sonja Walcher, Christof Körner, Mathias Benedek **

University of Graz, Austria sonja.walcher@uni-graz.at

Eye as a measure of attentional processes is gaining popularity in eye movement research. It is therefore important to know if eye vergence can be considered an objective parameter or if participants can control it at will. We asked 48 participants to focus on a fixation cross on a screen (baseline), look through it (diverge) or look in front of it (converge) for 10 seconds each. After each task, participants rated their performance. A quarter of our participants were able to make both convergence and divergence eye movements on command. Almost two thirds succeeded to make one vergence movement on cue but not the other; one sixth could not induce either. Some participants did not only fail to make the asked vergence movement, they even made the opposite one. Given these results it is not surprising that the performance judgements were not related to the actual degree of eye vergence. Our results demonstrate that some people are able to voluntarily control eye vergence in the absence of a stimulus but most participants lack awareness of their eye vergence movements. Thus, to ensure objectivity of this measure, participants’ ability to control vergence should be assessed in future studies.

**Maintaining stability in a fixation task: Are stimuli at all eccentricities equally effective?**

**Anna-Katharina Hauperich, Hannah E. Smithson **

University of Oxford, United Kingdom anna-katharina.hauperich@pmb.ox.ac.uk 

Fixation targets are typically small stimuli that participants are asked to foveate. In central vision loss, fixation stability is poor, but many ophthalmic procedures require steady fixation. We tested how peripheral stimuli contribute to a participant’s ability to maintain stable eye position during a fixation task. Stimuli were Gabor-like patches presented to the left and right of the desired fixation location in an otherwise dark environment. We compared retinal eccentricities of 3 and 6 degrees. To equate the detectability of stimuli at these eccentricities, we established the scaling factors required to match participants’ detection thresholds for low-contrast versions of the stimuli. For the fixation task, we used higher contrast versions of the scaled stimuli, and measured eye-movements (Eyelink1000, 2kHz sampling rate) whilst participants held their gaze in the centre of the stimulus pair for 3 seconds. Between trials, a bright noise-field maintained the retina in a light-adapted state. When assessing stability, we computed deviation from the overall mean fixation during each trial. Our results show that for perceptually matched visual information, fixational stability does not depend on stimulus eccentricity, suggesting that the visual input to eye stability reflects similar scaling to the m-scaling that equates the detectability of grating stimuli. 

**Extrafoveal perception of geometric shapes in adults and children **

**Anatoly N. Krichevets, Dmitry V. Chumachenko, Anna A. Dreneva, Anna Y. Shvarts **Lomonosov MSU, Russian Federation ankrich@mail.ru 

Numerous researches show that reading process includes extrafoveal perception of a text, and its role increases when reading skills develop. The purpose of the investigation was to determine variations of foveal and extrafoveal perception of geometrical objects depending on the competence level. University students and 7-8-year-old children were asked to find an object with a target shape as quick as possible. Each of the 96 trials presented four geometrical objects located at the same distance from the central fixation point. The target shapes, the target and the distractors similarity, and the habitualness of their orientation were varied. The results indicate that a target shape was recognized by the first saccade or without any saccades by the adults in the case of mild contrast of a target and the distractors. Children demonstrated certain extrafoveal perception only in the case of very high contrast. Note that the adults showed greater variety of perceptual strategies in comparison with the children. The results suggest that greater competence is accompanied by folding of searching saccades and enrichment of perception strategies. However, these strategies are strongly individual and their dependence on the subject’s personal and cognitive features needs further investigation. Supported by RFBR, grant No. 15-06-06319. 

**What can and what cannot be perceived extrafoveally **

**Anna A. Dreneva, Anna Y. Shvarts, Dmitry V. Chumachenko, Anatoly N. Krichevets **Lomonosov Moscow State University, Russian Federation annadrenyova@mail.ru 

Continuing research programme on extrafoveal perception of geometric shapes (Krichevets et. al., in press), we explored the ability to distinguish a target polyhedron among three other polyhedrons. We also studied how well extrafoveal processes could be trained. In the first experiment, university students were asked to find a pyramid or a prism among other pyramids or prisms with 3-, 4-, 5-, and 6-angular base. The objects were located at the same distance from the fixation point. The number of saccades preceding purpose fixation was calculated. All participants demonstrated significantly less number of saccades than there would be in case of random fixations on the shapes, while searching for a prism among pyramids. Nobody showed extrafoveal perception when searching for a pyramid among pyramids. Note, that pyramids’ bases weren't visible while prisms' bases were. The second experiment contained a sequence of 128 trials on search for a pyramid among pyramids. It was shown that, despite the fact that originally nobody could perform the task extrafoveally, at the end of the training the target could be found without any saccade. Thus, extrafoveal processing of spatial shapes can be substantially trained even under such a difficult condition. 

**Attention and response speed in pupil old/new effects **

**Tim Graf, Andreas Brocher**

University of Cologne, Germany tim.graf@uni-koeln.de 

We tested the impact of attention allocation on pupil old/new effects, with words and pseudowords. In Expt1, we used a typical old/new paradigm: 40 items at study phase, 40 studied and 40 unstudied items at subsequent recall phase, asking Have you studied the letter string onscreen?. Old/new effects, i.e. larger pupils for studied than unstudied items, obtained for words and pseudowords. In Expt2, participants engaged in word/pseudoword discriminations rather than old/new discriminations, focused on word-likeness (Is the item a legal word?), and had ample time to respond. Old/new effects only obtained for words. Requiring speeded responses (Expt3) eliminated old/new effects altogether, as did word/pseudoword discriminations with participants’ focus on nonword-likeness (Is the item a pseudoword?) irrespective of response speed (Expt4: ample time; Expt5: speeded responses). Our data indicate that pupil old/new effects crucially require attention. When discriminating studied and unstudied items, attention is distributed equally across stimuli. In contrast, word/pseudoword discriminations involve more attention to words than pseudowords, through presence vs. absence of long-term memory representations. Increasing task difficulty through the need for speeded responses or the shift of attention to the pseudowords reduces - but does not eliminate - attention to the words.

**Effect of on ocular fixation and microsaccades during optic flow **

**Marcia Bécu, Guillaume Tatur, Alix de Dieuleveult, Changmin Wu, Silvia Marchesotti, Denis Sheynikhovich, Angelo Arleo **

Sorbonne Universités, UPMC Univ Paris 06, INSERM, CNRS, Institut de la Vision, Paris, France marcia.becu@inserm.fr 

Optic flow can influence the ocular fixation and statistics of fixational eye movements (FEM), therefore affecting the quality of visual information accessible to the brain. While aging is known to alter the processing of dynamic visual cues, the influence of optic flow on FEM remains poorly characterized. This study assessed ocular fixation and FEM under no-flow (control), radial and tangential optic flow conditions in 66 subjects (21–83 y/o). Microsaccades were analyzed using a novel unsupervised clustering method that permits reliable detection in the presence of high-frequency pupil detection noise. We found that fixation areas were larger in old adults. All optic flow conditions reduced the fixation area to a similar extent in all age groups. Moreover, tangential optic flow significantly affected the ocular fixation drift slope, and amplified the extent of drift significantly more in aged compared to young subjects. Our microsaccade analysis extends previous data, showing that healthy aging significantly increased microsaccade frequency, amplitude, and peak velocity. Moreover, optic flow influenced all microsaccade characteristics, by reducing frequency, amplitude, and peak velocity. Tangential optic flow significantly triggered microsaccades in the opposite direction to the flow. 

Importantly, this directional bias tended to be stronger in old adults. 

**Saccadic adpatation increases brain excitability: a MEG study**

**Judith Nicolas**^1^**, Aline Bompas**^2^**, Romain Bouet**^1^**, Olivier Sillan**^1^**, Eric Koun**^1^**, Christian Urquizar**^1^

**Alessandro **^1^**, Aurélie Bidet-Caulet**^1^**, Denis Pélisson**^1^

^1^Lyon Neuroscience Research Center, INSERM, Unit 1028, Lyon, France.; ^2^School of Psychology, Cardiff University, Cardiff, Wales, UK judith.nicolas@inserm.fr 

Attention and saccadic adaptation are critical components of visual perception, the former enhancing sensory processing of objects of interest, the latter maintaining the accuracy of saccadic eye movements toward these objects. Recent studies propelled the hypothesis of a tight functional coupling between these two mechanisms. Indeed adaptation of reactive saccades towards the left hemifield increases the processing speed of unpredictable stimuli (Habchi et al., 2015), conversely attentional load boosts saccadic adaptation (Gerardin et al. 2015) and finally, their neural substrates (Gerardin et al. 2012, Corbetta and Shulman 2002) partially overlap. Here, we used magnetoencephalography to gain understanding of the neurophysiological bases of this coupling. We compared visual discrimination performance of 12 healthy subjects before and after an adaptation or control task involving reactive saccades. Eye movements and magnetic signals were recorded continuously. The neurophysiological analysis focused on gamma band power during the pre-target period of the saccadic adaptation and the discrimination tasks. Although attentional modulations by saccadic adaptation failed to impact behavioral performance in our paradigm, they could be demonstrated at the electrophysiological level as an increase of gamma band power within an extended brain network. These results suggests that gamma oscillations mediate the coupling between attention and saccadic adaptation

**Localization of briefly flashed targets across sequential eye-movements **

**Janne van Aswegen, Stefan Dowiasch, Frank Bremmer **

Department of Neurophysics, Philipps- Universität Marburg, Germany Janne.Aswegen@physik.uni-marburg.de

Eye-movements induce characteristic visual spatial localization errors of briefly flashed stimuli. It is unknown, how localization errors emerge, when different types of eye movements are performed in sequential order. We performed a psychophysical localization study with eight human subjects during a sequence of fixations, saccades and smooth pursuit eye-movements. Localization targets (diameter: 0.5°) were flashed for 8.3 ms at one of four positions equally spaced around the fovea (gaze contingent) and localized with a mouse-pointer. After central fixation, the oculomotor target stepped 10° into the periphery and started moving at 10°/s either centripetally (i.e. opposite to saccade direction) or perpendicular to saccade direction. The transition from fixation to saccade induced the mislocalization to merge from a centripetal bias (fixation) to a shift in the direction of the saccade. Steady state pursuit revealed the previously described localization shift in direction of the pursuit. During the transition from saccade to pursuit, however, the localization pattern associated with the saccade appeared truncated and taken over by an early pursuit-related component. Our findings imply an interference between localization patterns of saccades and pursuit. In future experiments, we will aim to determine the neural basis of this phenomenon by recordings in the macaque monkey. 

**The influence of threat associated distractors on express saccades **

**Jessica Heeman**^1,2^**, Stefan Van der Stigchel**^2^**, Jan Theeuwes**^1^>

^1^Vrije Universiteit Amsterdam, Netherlands; 

^2^Utrecht University, Netherlands 

j.heeman@vu.nl 

Previous studies have shown that regular saccades curve away from stimuli associated with threat. It is unknown whether threat also affects so called express saccades, saccades with a latency below 130 ms. It is generally believed that these express saccades --which have latency distributions that are different from that of regular saccades-- represent a different type of saccades, which should be less vulnerable to “higher” cognitive processes such as emotion and threat. Alternatively, express saccade may be nothing else than very fast regular saccades and as such should also be malleable by threat. Our previous research have shown that both express and regular target-driven saccades are affected by the presence of close or remote distractors. We designed a task in which participants had to make a saccade to a target in the presence of a colored close (20°) or remote (50°) distractor. Subsequently, we associated one color with the possibility of receiving an electric shock. Analysis showed that the threat association did not affect saccade averaging for express saccades but increased saccade averaging for regular saccades. This result suggests that unlike regular saccades, express saccades are not vulnerable to the cognitive influences such as threat or emotions. 

**Stereoacuity in the temporal proximity of vergence movements **

**Thomas Eggert**

Ludwig-Maximilians Universität, Germany eggert@lrz.uni-muenchen.de

Stereoscopic depth perception is challenged by natural vergence movements inducing large global mean values of absolute disparity (Da) and global disconjugate image velocity (DV). The current study investigates the effects and possible aftereffects of these variables on the perceptual threshold of relative disparity. Thresholds were measured during and shortly after symmetrical divergence movements to investigate the effects of both Da and DV on stereoacuity. During the vergence movement, thresholds were about 3.5 times (0.55 decades) larger than during fixation. Between these two viewing conditions, the Da of the test stimulus differed by 0.86 deg, and the DV by 3.85 deg/s. Thresholds measured immediately after movement end and 2 s later did not differ. Thus, no aftereffects of Da or DV were observed. In a control experiment the effect of Da on the disparity threshold was measured in the absence of DV. In this experiment, the threshold increased by 1.68 decades per degree change of Da from its optimal value. Thus, this effect alone was more than sufficient to explain the threshold increase during the divergence movement (1.68 > 0.55/0.86=0.64). This result suggests that Da and not DV is the main factor limiting stereoacuity during slow symmetrical vergence movements. 

**A Tool-based Process for Generating Attention Distribution Predictions **

**Sebastian **^1^**, Bertram Wortelen**^2^

^1^Human centered-Design, OFFIS Institute for Information Technology, Germany;

^2^Cognitive Psychology, C.v.O. University Oldenburg, Germany 

Sebastian@feuerstack.org 

Monitoring is one of the most important tasks for an operator driving a vehicle. Designing Human Machine Interfaces (HMI) for vehicles requires optimizing what is presented to the most limited resource: the driver’s visual attention. But how a car driver divides attention is hard to anticipate for a human factors (HF) expert. Eye tracking studies therefore are performed to measure the impact of design changes to the drivers’ visual attention. Cognitive models can complement these studies specifically in an early design phase as they do not require functional HMI prototypes to measure visual attention but instead generate valid predictions by simulating human visual attention based on psychological and physiological plausible models. We present a software tool that can efficiently capture operator domain knowledge, simulate human visual attention, and generate valid visual attention predictions. In an experiment we collected with the software tool knowledge from 20 car drivers in parallel session. Their aggregated attention prediction was high (R=0.719) and better than the average prediction of an individual HF expert (of a group of 8) compared to the drivers’ monitoring behavior that we measured using an eye tracking device in a car simulator. 

Reading: Visual and orthographic processing 

**Statistical of Oculomotor Processes During Reading**

**Johan Chandra, André Krügel, Ralf Engbert **

University of Potsdam, Germany jochandr@uni-potsdam.de 

Knowing where the eyes land is crucial for our understanding of oculomotor processes during reading. Within-word landing positions, however, are surprisingly noisy, making it difficult to directly infer the intended landing positions from eye movement data. To obtain estimates for the intended landing position we analyse three different methods: (1) The mean value of all observations (“naive” mean method), (2) the peak of truncated Gauss distribution (i.e., based on a Gaussian fitting method), and (3) the maximum a posteriori estimator from Bayesian inference. The effectiveness of the three methods, indicated by mean absolute estimation error and mean estimation bias, in estimating the mean (?) and standard deviation (?) of landing positions and the slope parameter (?) of landing-position function are tested based on different simulation models. As a result, the Bayesian approach is most reliable, even for small sample size (e.g., N=20), in reconstructing the parameters. The “naive” computation of the mean landing position shows the strongest systematic bias and should not be used for the analysis of eye-movement control during reading. 

**Contrast change effects reveal time course of parafoveal processing in eye movements during reading**

**Tina Schlachter, Sarah Risse**

University of Potsdam, Germany schlachter@uni-potsdam.de

In the context of reading, parafoveal information of not-yet-fixated words contributes to the timely movements of the eyes. While much has been learned about the type of information acquired in parafoveal vision, little is known about when this information is available to the oculomotor system to plan its next move.Manipulating the temporal availability of the parafoveal preview gaze-contingently, we varied the contrast of a target word n+1 from high to low (or low to high) at six different time points after fixation onset on the pretarget word n (i.e., after 0, 40, 80, 120, 160, and 200 ms). End-of-fixation visibility of the preview was overall more beneficial as compared to beginning-of-fixation visibility. Asymptotic benefits suggested that the first 80-120 ms of preview were not crucial to undisturbed reading. However, presenting high-contrast preview during the first 40 ms of pretarget fixation, we observed small benefits as compared to the full low-contrast condition. The present results support findings from Inhoff, Eiter, and Radach (2005, JEP:HPP) and confirm the superiority of end-of-fixation preview. However, they indicate gradual accumulation of preview starting early during the beginning of fixation. We will discuss the results with respect to spatio-temporal properties of attention during reading. 

**Gaze-contingent unmasking of filtered text regions during reading of graphic literature **

**Sven Hohenstein, Jochen Laubrock, Eike M. Richter **

University of Potsdam, Germany sven.hohenstein@uni-potsdam.de 

Graphic is characterized by a combination of visual and textual information. In order to understand the narrative, readers have to integrate both types of information. Although the proportion of visual elements is dominant in comics, readers spent most of their time reading text in speech bubbles and captions. Sometimes picture content is not fixated at all. We assume that visual information is processed in parafoveal vision. We conducted a study, in which we masked text in graphic literature with low-pass filtering rendering word identification impossible. Text was unmasked only after the reader looked at visual elements and made a saccade to a speech bubble or caption. In contrast to a control condition without masking, this experimental condition was assumed to increase the probability of picture fixations. In the masking condition we observed more first fixations and fixations in general on visual elements. Furthermore, fixations outside text regions were longer, and therefore picture context was viewed for a longer time in the masked condition. Interestingly, the probability for first fixations on characters is higher when text is masked initially, indicating that readers are able to process visual information of the upcoming panel and guide their gaze towards regions of interest. 

**The effect of misspellings on reading of correctly spelled words, across paradigms and languages **

**Victor Kuperman, Sadaf Rahmanian**

McMaster University, Canada vickup@mcmaster.ca 

Spelling errors can be seen as an effect of a word’s weak orthographic representation in an individual mind. What if spelling errors are a partial cause of effortful word recognition? We selected words that had homophonous substandard spelling variants, which varied in their frequency (innocent and inocent occur in 69% and 31% of occurrences of the word). Conventional forms of English words were presented for recognition either in context (Experiment 1, eye-tracking sentence reading, N = 35) or in isolation (Experiment 2, lexical decision megastudy data). The critical predictor was spelling entropy, i.e. a measure of uncertainty regarding which of spelling variants is a preferred one: Entropy is lower when one variant is clearly dominant and higher when available variants are similar in probability. Generalized additive models showed that higher-entropy words elicited reliably longer total fixation durations and higher regression-in rates, as well as longer lexical decision latencies. The effects were particularly strong in higher-frequency words, and did not depend on individual reading or spelling skill. Pilot eyetracking and lexical-decision data in Hebrew confirm these trends. Readers pay a price for spelling errors even if their own spelling skill is excellent and even when reading conventionally spelled words. 

**Reading at the speed of speech: Convergence between visual and auditory language perception at 4-5 Hz?**

**Benjamin Gagl**^1,2^**, Julius Golch**^1^**, Stefan Hawelka**^3^**, Jona Sassenhagen**^1^**, David Poeppel**^4,5^**, Christian J. **

**Fiebach1,2,4 **

^1^Goethe Universtiy Frankfurt, Germany; 

^2^Center for Individual Development and Adaptive Education of Children at Risk (IDeA) Frankfurt am Main, Germany; 

^3^University of Salzburg, Austria; 

^4^Max Planck Institute for Empirical Aesthetics, Germany; 

^5^New York University, USA gagl@psych.uni-frankfurt.de 

Across languages, the speech signal is characterized by a 4-5 Hz rhythm in the amplitude modulation spectrum. It is suggested that during comprehension, this temporal structure drives brain activity in the language system, reflecting the processing of linguistic information chunks every 200-250 ms. Interestingly, this is the typical eye-fixation duration in reading. To investigate this observation systematically, we first realized a meta-analysis (36 studies; N=273 FDs; Figure 1a). The analysis demonstrates that the predominant fixation-based ‘sampling frequency’ across different languages is between 4-5 Hz, with systematic differences between languages reflecting the difficulty of the writing systems. In a second investigation (N=50; Figure 1b) the individual sampling frequencies for sentence reading were around 5 Hz (~200 ms) with a low standard deviation (0.6 Hz). This is consistent with the German meta-analytic data. In z-string-scanning, a non-linguistic control task, the sampling frequency was significantly lower (4 Hz; Wilcoxon-rank test) with a significantly higher variance (0.8 Hz). The observed range suggests a remarkable temporal alignment of reading and speech processing. This invites the hypothesis that the language system drives voluntary eye-movements in reading, presumably to supply linguistic information in chunks at an optimal rate, 4-5 Hz, reflecting a common uptake for linguistic information. 

**Effective visual field of horizontal and vertical reading in Japanese **

**Nobuyuki Jincho**

Waseda University, Japan jinchod@aoni.waseda.jp

This study compared size of effective visual field (known as perceptual span and word identification span) for horizontal and vertical Japanese text. In a moving window paradigm experiment, Japanese adults read two novel stories with one horizontal and one vertical reading. A gaze contingent moving window controlled the number of presented characters: 1, 2, 4, 6, 8, or 10 characters of a current fixated character in horizontal text (left and right) or vertical text (above and below), respectively. The preliminary results of the generalized linear models on forward fixation duration (FFD) and forward saccade length (FSL) revealed that FFD increased if the below 4 characters in vertical text and the left 2 characters in horizontal text were perturbed than they were not. In addition, FSL was shorter when the right 8 characters in horizontal text and the below 6 characters in vertical text were perturbed than they were not. These results suggest that the word identification span is wider for vertical text than for horizontal text, while the perceptual span is wider for horizontal text than for vertical text. It also suggests that the saccade planning strategy may be different for horizontal and vertical reading in Japanese. 

**The perceptual span of young and older Chinese readers **

**Victoria A. **^1^**, Kayleigh L. Warrington**^1^**, Lin Li**^2^**, Sha Li**^1,2^**, Yingying Zhang**^2^**, Yuxiang Yao**^2^

**Jingxin Wang**^2^**, Sarah J. White**^1^**, Kevin B. Paterson**^1^

^1^University of Leicester, United Kingdom; 

^2^Tianjin Normal University, China vm88@le.ac.uk 

Older Chinese adults (aged 65+) read more slowly than their younger counterparts (aged 18-30), and adopt a cautious reading strategy in which they make more and longer fixations, more regressions, and are less likely to skip past words. These reading difficulties are poorly understood. In particular, it has yet to be determined how young and older Chinese readers may differ in their perceptual span, i.e. the region within which information can be acquired from a text during a single fixation. Accordingly, young and older Chinese adults read sentences in which a gaze-contingent moving window was used to obscure text to the left and/or right of the fixated word. In Experiment 1 characters outside the window were replaced with a uniform pattern mask, and in Experiment 2 characters were replaced with pseudocharacters. The results indicate that young and older adults processed text within a similar sized region, but the reading of the older adults was disrupted to a greater extent when characters to the right of the fixated word were replaced with pseudocharacters. The implications of these results for understanding how the perceptual span changes across the lifespan will be discussed.

Effects of and Pattern Complexity on the Visual Span of Chinese Readers

**Kayleigh L. Warrington**^1^**, Lin Li**^2^**, Fang Xie**^2^**, Sha Li**^2^**, Jingxin Wang**^2^**, Victoria A. McGowan**^1^**, Sarah J. White**^1^**, Kevin B. Paterson**^1^

^1^University of Leicester, United Kingdom; 

^2^Tianjin Normal University, China klw53@le.ac.uk 

Research with young adult Chinese readers suggests that pattern complexity (i.e., number of character strokes) limits the visual span (i.e., number of characters that can be recognized accurately on a single glance; Wang, He, & Legge, 2014). This is attributed to greater visual crowding for more complex characters. Older adults read Chinese more slowly than younger adults (Wang et al., 2016). Moreover, they experience sensory declines that may limit their ability to recognize complex Chinese characters, including increased effects of visual crowding. Whether these age-related visual changes produce smaller visual spans, and therefore slower reading, is unclear. Accordingly, we assessed the visual spans of young and older Chinese readers using low, medium and high complexity characters. An eye-tracker ensured participants fixated a designated fixation point accurately and brief displays of character triplets were presented at different horizontal eccentricities. Span size differed as a function of character complexity for both age groups, but older adults had a smaller visual span than younger adults for high complexity characters. The indication is that older Chinese readers acquire more limited character information when that information is high in pattern complexity, and this may be an important factor underlying adult age differences in Chinese reading. 

**Adult Age Differences in Eye-Guidance during Chinese Reading **

**Jingxin Wang**^1^**, Lin Li**^1^**, Sha Li**^1^**, Yingying Zhang**^1^**, Kevin Paterson**^2^

^1^Academy of and Behavior,Tianjin Normal University, Tianjin,China;

^2^College of Medicine, Biological Sciences and Psychology, University of Leicester, Leicester,UK wjxpsy@126.com 

Evidence indicates that young adults use parafoveal information about upcoming character information to guide their eye movements when reading Chinese (Li, Liu, & Rayner, 2011; Yan, Kliegl, Richter, Nuthmann, & Shu, 2010). However, it is unclear if the greater reading difficulty experienced by older readers is associated with less effective use of these parafoveal cues. To address this issue, we recorded the eye movements of young adult (18-21 years) and older adult (65+ years) native Chinese readers presented with sentences containing either a 2- or 4-character target word. Target words of each length were matched for the complexity of their first character, lexical frequency and predictability in sentences. Typical patterns of age-related reading difficulty were observed. In addition, word length effects were observed for the young but not the older adults for words receiving only one first-pass fixation, and for neither age-group for words receiving multiple first-pass fixations. These results add to the evidence that parafoveal character information guides saccade-targeting during Chinese, but reveal that the effectiveness of these parafoveal cues declines with age. We consider these findings in relation to current theories of eye-guidance during Chinese reading and implication for understanding changes in Chinese reading behavior in older age. 

**Eye Movement Control and Word Identification During Vertical and Horizontal Reading: Evidence from Mongolian **

**Kevin Paterson**^1^**, Juan Su**^2^**, Guoen Yin**^2^**, Stoyan Kurtev**^3^**, Simon Liversedge**^4^**, Bai Xuejun**^2^**, Guoli Yan**^2^****^1^University of Leicester, United Kingdom; 

^2^Tianjin Normal University, China;

^3^University of , UK; 

^4^University of Southampton, UK kbp3@le.ac.uk 

Mongolian is a cursive alphabetic language that is conventionally printed vertically (so that sentences are effectively rotated 90° from horizontal) and so naturally read from top to bottom, but can also be printed horizontally. This language is therefore ideal for assessing the versatility of word identification and oculomotor control when reading text in different directions. Two experiments addressed this issue by examining the influence of reading direction and both word frequency (Experiment 1) and word length (Experiment 2) on eye movement control. In both experiments, horizontal reading was slower than vertical reading, although effects of word frequency and word length were similar for the two reading directions. Crucially, the initial landing positions of fixations on words were also broadly similar for the two reading directions, and in Experiment 2 were closer to the beginnings of longer words. Thus, while reading is generally slower for the less familiar reading direction, this did not disrupt normal processes of word identification or saccade-targeting (e.g., Rayner, 1979). The findings therefore reveal that processes of word identification and eye movement control are highly adaptable to these changes in reading direction.

**The Perceptual Span during Vertical and Horizontal Reading: Evidence from Mongolan **

**Kevin Paterson**^1^**, Juan Su**^2^**, Guoen Yin**^2^**, Stoyan Kurtev**^3^**, Simon Liversedge**^4^**, Bai Xuejun**^2^**, Guoli Yan**^2^****^1^University of Leicester, United Kingdom; 

^2^Tianjin Normal University, China; 

^3^University of , UK; 

^4^University of Southampton, UK kbp3@le.ac.uk 

Research shows that the perceptual span adjusts flexibly to the direction of reading for alphabetic languages read from left-to-right (e.g., English) or right-to-left (e.g., Arabic, Hebrew). The indication, therefore, is that asymmetry in the perceptual span reflects the allocation of attention in the direction of reading. However, little is known about the perceptual span for alphabetic languages like Mongolian that can be read vertically, from top-to-bottom, or horizontally, from left-to-right. Accordingly, we investigated the perceptual span during vertical and horizontal Mongolian reading. Text was presented entirely as normal or in a gaze-contingent paradigm in which a window of text was displayed as normal at the point of fixation and text outside this region was blurred to obscure letter identities. The windows of normal text extended either symmetrically about fixation, or asymmetrically above and below or to the left and right of fixation. Reading rates for Mongolian readers were closest to normal when the windows of normal text extended asymmetrically either below or to the right of fixation. These findings provide further evidence that the perceptual span is determined by the allocation of attention in the direction of reading, and novel evidence that such effects are observed during vertical alphabetic reading.

Investigating word length in Chinese reading: Evidence from eye movements

**Chuanli Zang**^1^**, Ying Fu**^1^**, Simon P. Liversedge**^2^

^1^Tianjin Normal University, China;

^2^University of , UK 

 c.zang@soton.ac.uk 

A word’s length in English is fundamental in determining whether readers fixate it, and how long they spend processing it during reading. Unlike English, Chinese is unspaced without interword spaces marking word boundaries, and most words are one or two characters long, resulting in less variability in word length. This raises the question of whether word length is an important cue in Chinese reading? Readers’ eye movements were monitored as they read sentences containing a one-, two-, or threecharacter word of similar frequency. When only the target word region was analyzed (with its stroke number as a covariate), the results showed that the longer a word was, the longer it took to process. This effect was mainly driven by refixations (including gaze and total fixations) rather than first or single fixations. Furthermore, increased word length resulted in less skips, landing positions further to the right of words, and longer outgoing saccades. However when a three-character region (with perfectly matched stroke number) was analyzed, there was an incremental processing cost when the additional character(s) belonged to a different word rather than the same word. These results demonstrate that word length affects both lexical identification and saccade target selection in Chinese reading. 

The last, but not the initial character’s positional frequency affects Chinese compound word processing in reading

**Feifei Liang, Qi Gao, Jie Ma, Hao Wu, Xuejun Bai **

Tianjin Normal University, China, People's Republic of feifeiliang_329@126.com 

Recent studies have demonstrated that the positional frequencies of word’s constituent characters contribute to the process of Chinese compound word during reading (Liang et al., 2015). Since the morphemes in different within-word positions play different roles when processing compound word of Chinese, two experiments were conducted to examine the effects of initial and final character’s positional frequency on compound word’s identification in reading. In experiment 1, on the basis of each character’s positional frequency, the initial character of two-character compound word was manipulated to be at word beginning with high- or low- probability while the final character was controlled as equally to occur at word beginning and ending. In experiment 2, similar manipulation was made for the final character while the initial character was controlled. We found that reading time was remarkably reduced when reading compound words where the final character occurred at word ending with high-probability compared to that with low-probability, such pattern did not occur for our manipulation of initial character. It appears that only the final character’s within-word positional information played important role on segmenting and identifying words during Chinese reading. These data will be discussed within the context of word segmentation and recognition model in Chinese. 

**The role of spaces in segmenting Finnish and Chinese text **

**Raymond Bertram**^1^**, Liyuan He**^2^**, Simon Liversedge**^3^

^1^University of Turku, Finland; 

^2^Tianjin Normal University; 

^3^University of Southampton rayber@utu.fi 

In alphabetic languages like English or Finnish, word boundaries are clearly indicated by interword spaces and presenting these languages without spaces slows down reading to a great extent (Rayner et al, 1998). In the current eye movement study we first investigated the role of spaces in reading Finnish sentences including target word compounds (e.g., vuorileijona ’mountain lion’) of which the first part 

(mountain) either was compatible with the preceding context (as in 

'hesawthemountainlionfromadistance’) or not (as in ’heheardthemountainlionfromadistance’). The main finding was that especially for the unspaced condition, reading proceeded more smoothly in the latter case. Similar decisions have to be made in Chinese constantly, as subsequent characters often but not always need to be unified to form compound words. To investigate this issue further, we conducted an eye movement experiment in Chinese in which 3-character clusters (ABC) were included that could either be segmented into an AB-C or A-BC 2-word combination. Here spacing - consistent with the preceding context interpretation - facilitated ambiguity resolution in comparison to the unspaced condition. However, the text before the ambiguity was read faster in the unspaced condition. Both experiments show that spacing may be facilitative, but mostly in case of local ambiguity. 

**Vertical movement within fixations in the reading of Chinese and English **

**Yi-ting Hsiao**^1^**, Richard Shillcock**^1^**, Mateo Obregón**^1^**, Hamutal Kreiner**^2^**, Matthew A.J. Roberts**^1^**, Scott **

**McDonald**^3^

^1^University of Edinburgh, United Kingdom; 

^2^Ruppin Academic Centre, Emeque-Hefer, Israel; 

^3^National Institute for Public Health and the Environment, Bilthoven, Netherlands nyhsiao@yuntech.edu.tw 

We report analyses from the Edinburgh 5-Language Corpus showing comparable behaviour in the reading of Chinese and English, in the set of binocular fixations that begin and end synchronously (about 50% of all fixations; >100k fixations in each language). Both sets of readers tended to make upward movements within fixations. The calculations do not refer to precise registration on the text, but all fixations occurred during reading for meaning. In reading, the earlier part of a fixation tends to be more associated with visual recognition, the later part with executive action (i.e. the next saccade). In the absence of an alternative explanation for the upward movement, we suggest that the tendency to move upwards represents an overall tendency for the earlier part of a fixation to involve the ventral pathway and the later part to involve the dorsal pathway, with their respective processing specializations (cf. Milner & Goodale, 1995). In addition, this direction of movement means that the more informative upper part of words/characters is available to the ventral pathway longer (cf. Blais, Fiset, Arguin, Jolicoeur, Bub, & Gosselin, 2009; Chi, Yan, Meng, Zang, & Liversedge, 2015).

**When readers pay attention to the left: A concurrent eyetracking-fMRI investigation on the neuronal correlates of regressive eye movements during reading **

**Anna F. Weiß**^1^**, Franziska Kretzschmar**^2^**, Arne Nagels**^1,3^**, Matthias Schlesewsky**^4^**, Ina Bornkessel-**

**Schlesewsky**^4^**, Sarah Tune**^5^

^1^Department of Germanic Linguistics, Philipps-University of Marburg, Germany;

^2^Institute of German Language and Literature I, University of Cologne, Germany;

^3^Department of Psychiatry and Psychotherapy, Philipps-University of Marburg, Germany;

^4^School of , Social Work & Social Policy, University of South Australia, Adelaide, Australia; ^5^Department of Psychology, University of Lübeck, Germany fiona.weiss@staff.uni-marburg.de 

Predictive coding postulates that saccades are used to actively test hypotheses about the causes of sensory input (Friston et al., 2012). Accordingly, refixations in sentence reading may be triggered by prediction errors as indicators of the need to update one's internal model of the world. Refixations follow regressive inter-word saccades after changes in attentional re-orientation (Apel et al. 2012). The presence/absence of prediction errors and leftward attention shifts predict qualitatively different activation patterns for regressive and progressive saccades in brain regions involved in reading. We tested this hypothesis using concurrent fMRI-eyetracking.Twenty-three native German speakers read semantically anomalous and non-anomalous sentences. Progressions and regressions were identified via eye-movements and temporally correlated to BOLD signal changes. Onsets and durations were modeled separately per saccade type. At the group level, we contrasted regressive and progressive saccades and examined amplitude modulation differences by saccade length. Activation patterns differed substantially between saccade types. Progressions reveal bihemispheric deactivation especially in frontal regions with only sporadic activations at temporal sites (e.g. left MTG). Regressions engender broad bihemispheric activations within a fronto-parietal-temporal network, including regions of attention control. These findings suggest that neuronal activation for regressions correlates with resolution of prediction error and changes in attention direction. 

**Developmental Eye Movement Research **

**Fetal eye movements in response to a visual stimulus **

**Tim Donovan**^1,2^**, Kirsty Dunn**^2^**, Sophie Clarke**^2^**, Anna Gillies**^2^**, Olivia Mercer**^2^**, Vincent Reid**^2^

^1^University of Cumbria, United Kingdom; 

^2^Lancaster , United Kingdom tim.donovan@cumbria.ac.uk 

In 2D ultrasound the lens of the fetal eye can be distinguished as white circles within the hypoechoic eyeball, and eye movements can be visualised by movements of the lens from about 15 weeks’ gestation. In 4D ultrasound it is possible to view face and head movements, but determining eye movements can be problematic. However, as the 4D image is produced by selecting an ideal 2D image within the region of interest, 2D data is available for review. It has previously been shown that in the last 2 months of pregnancy the fetal sensory system is capable of directed vision if enough light is available (Del Giudice, 2011). We have developed a light source for delivering visual stimuli to be seen by the fetal eye, using laser dot diodes emitting at 650 nm. The 2D component of 60 fetal scans (mean gestational age 240 days), where the light stimulus was presented and moved across the maternal abdomen, was then reviewed and coded to determine whether the eyes moved in response to the stimulus irrespective of any head movement. Initial results indicate that more eye movements than head movements can be determined after the stimulus has been presented, suggesting fetal awareness.

**Early regulatory problems associated with the affect-biased attention at 8 month of age **

**Eeva Eskola**^1,2^**, Riikka Korja**^1,2^**, Eeva-Leena Kataja**^1,2^**, Linnea Karlsson**^2,3^**, Tuomo Häikiö**^1^**, Henri**

**Pesonen**^2^**, Jukka Hyönä**^1^**, Hasse Karlsson**^2,4^

^1^Department of Psychology, University of Turku, Finland; 

^2^FinnBrain Birth Cohort Study, Turku Brain and Mind Center, Institute of Clinical Medicine, University of Turku, Finland; 

^3^Department of Psychiatry, Turku University Hospital and University of Turku, Finland; 

^4^Department of Psychiatry, University of Turku, Finland Eeva epesko@utu.fi

Attentional biases have been associated with emotional dysregulation like anxiety and depressive symptoms in adults. However, little is known of the regulation problems and the attentional biases in infants. We examined whether the very early behavioural regulatory problems, known as risk factors of later emotional dysregulation, associate with affect-biased attention. This longitudinal data comprised 359 infants. The overlap paradigm was used in an eye-tracking experiment to measure the infant’s tendency to disengage attention from fearful, happy and neutral facial expressions and from a non-face picture at 8 months. Parents’ report was used to assess infants’ problems in sleeping, feeding and calming down at 3 months of age. The sum-variable of regulatory problems was formed based on the questions. A General Linear Model of the probability of disengagement with facial expression and regulatory problems as factors, revealed main effects of facial expression (F(3,635)=30.67, p<0.001, etasquared=0.13) and regulatory problems (F(1, 635)=25.59, p<0.001, eta-squared=0.039). The probability of disengagement from happy and neutral faces was lower in the group of infants with higher regulatory problems. According to these preliminary results, infants’ regulatory problems at 3 months seems to predict the heightened attention to happy and neutral facial expressions at 8 months.

**MATERNAL PRENATAL STRESS AND INFANT ATTENTION TO **

**EMOTIONAL FACES AT THE AGE OF EIGHT MONTHS IN FINNBRAIN BIRTH COHORT **

**Eeva-Leena Kataja**^1,2^**, Linnea Karlsson**^2,3^**, Henri Pesonen**^2^**, Jukka Leppänen**^4^**, Tuomo Häikiö**^1^**, Jukka**

**Hyönä**^1^**, Christine Parsons**^5,6^**, Hasse Karlsson**^7^

^1^Department of Psychology, University of Turku, Finland; ^2^FinnBrain Birth Cohort Study, Turku Brain and Mind Center, Institute of Clinical Medicine, University of Turku, Finland; ^3^Department of Child 

Psychiatry, Turku University Hospital and University of Turku, Finland; ^4^Infant Cognition Laboratory, 

Center Child Health Research, School of Medicine, University of Tampere, Finland; ^5^Department of 

Psychiatry, University of Oxford, UK; ^6^Interacting Minds Center, Department of Clinical Medicine, Aarhus University, Denmark; ^7^Department of Psychiatry, University of Turku, Finland elpelt@utu.fi 

Maternal prenatal stress (PS) may have programming effects on the neurocognitive development of the fetal brain with long-lasting consequences. To date, no study has investigated whether PNS has effects on affect-biased attention, moreover attention bias to threat, already in infancy. Eight month-old infants (N=318) exposed to either high (n=131) or low (n=187) levels of mother reported PS (depressive/anxiety symptoms) were compared for their affect-biased attention with eye tracking (EyeLink1000). The Overlap –paradigm with neutral, happy, fearful, and phase-scrambled face and a lateral distractor was used. High and low PS groups did not differ in terms of missing attention shifts from faces to distractor (ps, 0.23 – 0.81). All infants, irrespective of PS grouping or sex showed an age-typical bias to fearful face. However, high PS group girls tended to show heightened fear-bias (a median-split fear contrast measure), p=0.053. Finally, PS did not remain as a significant predictor of fear-bias (p=0.301), after controlling for maternal postnatal depression (p=0.002) and anxiety (p=0.013). Maternal stress may affect affect-biased attention in infants for instance by lowering the overall responsiveness to facial expressions or enhancing the processing of fear-related stimuli. The specific roles of prenatal and postnatal depressive and anxiety symptoms needs further investigation. 

**Infant free-viewing: the role of object knowledge**

**Daan van Renswoude, Maartje Raijmakers, Roos Voorvaart, Ingmar Visser **

University of Amsterdam, Netherlands

D.R.vanRenswoude@uva.nl 

What factors drive infants’ eye-movements over complex real-world scenes? In adults, both objects and perceptual salience influence gaze behavior. For younger infants, perceptual salience is likely to have a larger influence than objects, as many objects are unknown. Whereas gaze behavior of older infants is likely also influenced by objects. In this study we examine the role of object knowledge on infants’ eyemovements. Forty infants (6 - 12-month-olds) will free-view 29 scenes from the OSIE (Object and Semantic Images and Eye-tracking) dataset in which objects are tagged. Parents are asked to what extend they think their infant knows the objects displayed in the scenes on a scale from ‘never seen’ to ‘can name the object’. We fit a GLMM (Generalized Linear Mixed Model) to the data and control for the influence of the central bias, perceptual salience and the size of objects to assess the role of object knowledge on gaze behavior. More specifically, we expect that known objects are more frequently fixated than unknown objects. In addition we explore the role object knowledge has on fixation durations, fixation order, and number of fixations. Data collection is ongoing and we gladly present the results at ECEM. 

**Development of oculomotor control from infants to toddlers: **

**temporal and spatial parameters of voluntary saccades**

**Christelle Lemoine-Lardennois, Nadia Alahyane, Mallaury Hamon, Clara Ferrari, Karine Doré-Mazars **Laboratoire Vision, Action, Cognition, EA 7326, Université Paris Descartes, France christelle.lemoine@parisdescartes.fr 

During first years of life, eye movements represent a vital means to interact with the environment but the development of oculomotor control is still poorly known. We developed a novel paradigm to investigate reactive saccade performance in infants and toddlers (Alahyane et al., 2016). Our results revealed that saccade reaction time decreases with age and that saccade accuracy improves over the 160 trial session. Here, we adapted this paradigm to elicit voluntary saccades, based on an overlap procedure. In some trials (‘double target’), while the participant is fixating a stimulus, two remote peripheral stimuli appear simultaneously at a 10° eccentricity, at unpredictable locations. When one of the two stimuli is selected as the saccade target, the other stimuli disappear. The saccade target becomes then the fixation point of the following trial. In some other trials (‘single-target’), only one peripheral stimulus is displayed to examine the “remote distractor effect” (e.g., Walker et al., 1997). Voluntary saccade performance (amplitude, reaction time) in young participants (6-42 months-old) will be compared to a group of adults. Performance in single target trials will also be compared to our previous reactive saccade data. 

**Individual differences in children’s learning through eye-tracking experiment **

**Dmitry Chumachenko, Anna Shvarts, Anna Dreneva**

Moscow State University, Russian Federation dmitry.chumachenko@gmail.com 

Cognitive scientists often include repeated similar tasks in order to have more data for statistical analysis. However, participants (especially children) tend to learn new perceptual strategies during such an experiment. We investigated the influence of training on extrafoveal perception of geometric shapes. In our pilot study children (7-8 years-old, n=8) had to find one target object (a square or a rectangle) between the distractors as quick as possible in 64 trials. Three dependent variables were selected as learning indicators: time of task solving, the amount of fixations before finding the target (“necessary fixations”) and after it (“additional fixations”). We compared first and second half of trials by ANOVA within each child. Some children were getting to perform better (n=2; the amount of “necessary” and “additional” fixations significantly decreased, p<0,05) in the second part, other children were getting to performed worse (n=2; time of solving significantly increased, p<0,05). For “bad learners” the time and the amount of “necessary fixations” significantly correlate. Thus, we assume “bad learners” spent time for the search of the target. 

The results suggest strong individual differences in children’s perceptual strategies. Supported by RFBR, grant No. 15-06-06319

**Exploring the development of oculomotor attentional control in emotional and non-emotional contexts**

**Athina Manoli**^1,2^**, Simon P. Liversedge**^2^**, Edmund Sonuga-Barke**^3^**, Julie A. Hadwin**^1^****

^1^Centre for Innovation in Mental Health – Developmental Laboratory, University of Southampton, United Kingdom; 

^2^Centre for Vision and Cognition, University of Southampton, United Kingdom; ^3^Department of Child and Adolescent Psychiatry, Institute of Psychiatry, Psychology and Neuroscience, King's College 

London, United Kingdom A.Manoli@soton.ac.uk 

Evidence shows that oculomotor attentional and inhibitory control improves with age. That is, adults show fewer saccadic errors in their ability to suppress prepotent reflexive saccades over voluntary saccades and reduced saccade latencies for both reflexive and voluntary saccades compared to children. Although a large body of research has further examined how attentional control can be modulated by the presence of emotional stimuli, current understanding of such processing in children is still limited. In this study, we utilised eye-movement measurements in the Go/No-Go and Spatial Cueing paradigms to examine oculomotor inhibitory control (suppression of reflexive saccades) and attentional orienting in response to directional cue distractors, arrows and eye gaze, in both emotional and non-emotional contexts in adults and children. We also considered the influence of individual differences in anxious affect in emotion-modulated oculomotor attentional control. Children showed poorer attentional and inhibitory control, as indicated by the failure to execute voluntary saccades and increased saccade onset latencies following emotional contexts compared to adults. The findings from the current study extend our understanding on the developmental improvements in the interaction between cognitive control and emotional processing.

**Development of movements related to executive functions in elementary school students**

**Suxia Wang**^1^**, Ralph Radach**^2^**, Christian Vorstius**^2^**, Yan Sun**^1^**, Lizhu Yang**^1^****

^1^Liaoning Normal University, China; ^2^University of Wuppertal , Germany yanglizhu126@126.com 

Despite recent progress in this area, we still have no complete understanding of developmental changes in the control of eye movements associated with executive functions. The present work examined two visuomotor tasks with a total sample of 96 normally developing Chinese children in grades 2, 4, and 6, aged 7-12 years. In the variable cue-to-target interval saccade task, participants were asked to maintain fixation for 250, 1000, or 4000 ms, until a prosaccade target appeared. Results showed similar saccadic accuracy and gain in all three grades. For latencies, there was an interaction between grade and interval, indicating somewhat shorter latencies for older children. In the mixed pro/antisaccade task, the color of a fixation cross served as the cue for whether the required saccade was to be a prosaccade or antisaccade. As expected, performance (accuracy and latency) for antisaccades was inferior in all three grades. There was no grade difference in prosaccade latency, while antisaccade latency gradually decreased. The latency gap between pro- and antisaccades narrowed over time. The largest gain in antisaccade performance occurred between grade 4 and 6, suggesting late development of voluntary control. Further analyses will focus on the cost of switching between pro- and antisaccade responses. 

**Developmental research on eye movements in reading**

**Patterns of 5-6 year old children reading picture book: Evidence from eye movements**

**Yuanyuan , Peng Wan, Guiqin Ren **

Liaoning Normal University of China, China ofrenguiqin@126.com 

In this study, we used eye movement technique to explore the effect of reading style on children's picture book reading. Specifically, a total of two experiments were conducted to explore how reading styles(autonomous reading, shared reading), and the types of story (common sense, emotional expression) influence 5~6 years old children's reading. During the experiments, participants were asked to look at the pictures and Chinese characters on the screen and then answer three questions. Respectively, the area of text, picture and the whole page were taken as three areas of interest. Eye movement measures such as first fixation duration, total fixation duration, and number of fixations were recored and analyzed. 

The results showed that: (1) in both reading styles, children have shown a preference for the picture area. (2) story types to a certain extent affect the reading results. (3) the reading comprehension score of shared group was significantly higher than that of the independent group.

**The perceptual span of second graders in Chinese primary school**

**Guoli Yan, Sainan Li, Min Liu, Yali Wang **

Tianjin Normal University, China psyygl@163.com 

The perceptual span is the region where one can extract useful information during a single fixation. It varies as a function of age associated with reading skills. The present study mainly focused on exploring the perceptual span in second grade Chinese children(n=30). Using moving window paradigm developed by McConkie and Rayner (1975) to control the visibility of the characters during reading Chinese stories selected from the textbook, we collected children’s and 25 college students’eye movement measures (reading rate, the average fixation duration, average saccade amplitude, forward saccade amplitude). The results showed that the perceptual span of grade 2 is 1-2 characters to the right, while the perceputal span for the adult is 2-3 characters. Furthermore, for second graders, we also analyzed the correlation between the eye movement measures and the reading comprehension test , reading speed test, orthographic test. We found that there are significant correlations between the eye movement measures and the reading comprehension test , reading speed test. In conclusion, the perceptual span for second graders is smaller than adults about 1 character, and the perceptual span is related to reading comprehension and reading speed.

**Reading Instructions Influence Cognitive Processes of Illustrated Text **

** Reading for Young Readers: An Eye-Tracking Study **

**Yu-Cin Jian **

National Taiwan Normal University, Taiwan jianyucin@ntnu.edu.tw 

Young show limited ability to use illustration information and integrate it with the text in illustrated text reading. In the present study, readers were taught illustrated text reading strategies, and we investigated whether strategy instructions influence reading comprehension and learning processes. Sixty-two fourth-grade students read an illustrated science text while their eye movements were recorded, and then completed a reading test. The results showed that the instruction group outperformed the control group on the reading test, especially for illustration recognition and textillustration integration questions. As for the eye-movement data, the results of analysis showed that the instruction group spent a greater proportion of reading time (27%) on illustrations than the control group (16%). This indicates that students in the instruction group learned the illustration reading strategy that the majority of young readers do not develop naturally at that age, and they directed attention to illustration sections because they recognized the importance of the science illustrations. Besides, the instruction group made more saccades between the text and illustrations than the control group, especially between related text and illustration sections. Above findings indicated that reading instructions changed learning processes, helping readers use multiple representations during reading, leading to better learning. 

**The eye-tracking study of reading in Russian primary schoolchildren**

**Aleksei Korneev, Ekaterina Matveeva, Tatyana Akhutina **

Lomonosov Moscow State University, Russian Federation korneeff@gmail.com 

The study is directed to the analysis eye movements during silent reading in Russian on early stages of acquiring this skill. We developed the corpus that consists of 30 sentences with target words with controlled length and frequency. 37 second grade schoolchildren read the sentences and performed the test of reading words with regular and irregular spelling and high and low frequency. We used an EyeLink 1000 eye-tracker (SR Research, Ontario, Canada). The analysis was carried out in comparison with a similar study conducted on the material of the German language (Tiffin-Richards, Schroeder, 2015). Results showed that effects of frequency and length were similar in both languages however, Russian children made more single fixations and skips than German children. Additional analysis of the eye movements in two groups with better reading of regular or irregular words revealed that the difference in number and duration of fixations and fixation count in the groups support the hypothesis that the children of these groups use two different reading strategies – analytic and holistic. 

**Eye-Tracking sSudy of reading the texts of different types: Evidence from russian**

**Tatiana Petrova **

Saint-Petersburg State University, Russian Federationtatiana petrova4386@gmail.com 

The hypothesis is based on the assumption that a text type is among the readability categories and it influences the effect of reading perspective. Three experiments were carried out. In Exp.1 native speakers of Russian read static texts and a dynamic texts. In Exp.2 reading the texts of different functional styles by Russian and students learning Russian as a foreign language was studied. Exp.3 describes the process of reading a complex and a simple text (in a sense of contained propositions) by children with high level of reading and children with low level of reading. The results of the experiments indicate that the type of a text affects the individual patterns of oculomotor behavior during reading. Analysis of the results revealed differences when reading texts of different functional styles, static and dynamic texts, texts of varying difficulty in a large group of children of primary school age. The overall results of the study show that the influence of the type of text affects the text flow as you get closer to the "adult rate": the higher the level of development of reading skills, the greater the influence of the factor "text type". Checking the texts by readability formulas revealed correlations with eye tracking data. 

**The Correlation between Eye Movement Data and Three Commonly Used **

**Academic Reading Assessments **

**Alexandra Spichtig**^1^**, Kristin Gehsmann**^2^**, Jeffrey Pascoe**^1^**, John Ferrara**^1^****

^1^Reading Plus, United States of America; 

^2^Saint Michael's College, United States of America alex@readingplus.com

Slow reading rates may indicate that the process of taking in text has not yet become dynamic or automatic, but instead is labored and burdensome. Eye movement data (fixations, regressions, fixation duration, and reading rate) can be useful to educators for evaluating reading efficiency more thoroughly. This research examined the relationship between reading efficiency and the academic reading achievement of grade 4 and 5 students in the US. Eye-movement data were collected by a team of trained adults (using the Visagraph, a low-cost system that uses goggles fitted with infrared emitters and sensors to measure corneal reflections) while students read standardized 100-word grade level 4 passages, each followed by 10 true/false comprehension questions. Analyses were based on 322 students who completed the efficiency and three academic reading assessments (Group Reading Assessment Diagnostic Evaluation (GRADE), the Smarter Balanced Assessment Consortium (SBAC), and the Reading Plus InSight Assessment). The intercorrelation matrix indicated weak (r < .50) to moderately strong (r > .60) correlations between efficiency and academic reading measures. Apart from fixation duration, these correlations were stronger in grade 5. Apart from reading rate, these correlations were weak in grade 4. These results suggest an increasing role of efficiency in literacy achievement. 

**Effects of Scaffolded Silent Reading Practice on the Reading Related Eye Movements of US Students in Grades 4 and 5**

**Kristin Gehsmann**^1^**, Elias Tousley**^2^**, Alexandra Spichtig**^2^**, Jeffrey Pascoe**^2^**, John Ferrara**^2^****

^1^Saint Michael's College, United States of America; 

^2^Reading Plus, United States of America kgehsmann@smcvt.edu 

This research evaluated the impact of scaffolded silent reading practice (SSRP) on four measures of reading efficiency; reading rate, fixations and regressions per 100 words, and fixation duration. Eye movement recordings were collected from fourth and fifth grade students (~ ages 10 and 11) while they read standardized fourth grade level passages, each followed by a brief comprehension test. Recordings were made at the start and end of the 2015-2016 school year using a low-cost, portable eye movement recording system (Visagraph). Random assignment was used to divide 196 students, initially paired on reading proficiency and demographic factors, into control and treatment groups. Controls received their regular literacy instruction during the intervention block, while the treatment group engaged in SSRP (~100 lessons, totaling ~ 24.5 hours) using a web-based reading program (Reading Plus®). This program presents text through a moving window that travels across lines of text and down the page at a student’s individualized reading rate. Students in both groups increased their reading proficiency during the school year. Significantly larger improvements in three reading efficiency measures (reading rate, fixations, and regressions) were seen in the treatment group. These results suggest that SSRP helps students become more efficient readers. 

Relationship Students’ Stages of Orthographic Knowledge and Reading Efficiency 

**Kristin Gehsmann**^1^**, Elias Tousley**^2^**, Alexandra Spichtig**^2^**, Jeffrey Pascoe**^2^**, John Ferrara**^2^****

^1^Saint Michael's College, United States of America; 

^2^Reading Plus, United States of America kgehsmann@smcvt.edu 

This research evaluated the relationship between students’ orthographic knowledge, reading achievement, and four measures of reading efficiency: reading rate, fixations and regressions per 100 words, and fixation duration. Orthographic knowledge was assessed in 273 fourth and fifth grade students (~ ages 10 and 11) using an online version of the Elementary Spelling Inventory described in Words Their Way (Bear et. all, 2016). This measure enables the classification of students into five distinct stages of spelling and literacy development. Using a low-cost eye movement recording system (Visagraph), eye movement recordings were collected while students read standardized fourth-grade passages. Each 100-word test passage was followed by a brief comprehension check involving 10 true/false questions. All eye-movement measures differed significantly across the stages of development, with the upper stages being associated with faster reading rates, fewer fixations and regressions per word, and shorter fixation durations (p < .001). These results demonstrate a strong relationship among orthographic knowledge, reading efficiency (as measured by eye movement), and reading proficiency. While the reciprocal relationship between orthographic knowledge and reading achievement is well established, this is the first known demonstration of the relationship between these measures and oculomotor efficiency across multiple stages of literacy development. 

**The Reliability of Reading Efficiency Measures Obtained by Classroom **

** Educators Using a Low-Cost Eye Movement Recording System**

**Alexandra , Jeffrey Pascoe, John Ferrara **

Reading Plus, United States of America alex@readingplus.com 

The Visagraph is a portable eye movement recording system that uses goggles fitted with infrared emitters and sensors to measure corneal reflections. Educators use the system to evaluate reading efficiency. This research examined the test-retest reliability of the device. Recordings were collected by educators while students in even grades between 2 and 12 read a practice passage followed by four standardized passages at a student’s grade level, each followed by 10 true/false comprehension questions. Analyses were based on 827 students who completed at least two valid recordings; i.e., recordings were interpretable, line counts matched the text, and the comprehension criterion was met (70%). Using data from the first two valid recordings, test-retest reliability coefficients and coefficients of variation (CV) were calculated for reading rate, fixations, regressions, and fixation durations. Means for each measure closely matched efficiency norms reported in previous research. Reliability was lower in grades 2-4 and stabilized thereafter. In grades 6 and above, reliability coefficients for rate and fixations averaged .83 with CVs between 9% and 11%; measures of fixation duration had lower CVs (7.1%) and regressions had higher CVs (27.6%). These results are useful for guiding the interpretation of eye-movement data in educational and research settings. 

**What can we reveal from saccade events of eye movements when EFL high school students read narrative with illustration?**

**Grace Ming-Yi Hsieh, Sunny San-Ju Lin **

National Chiao Tung University, Taiwan grace.myhsieh@gmail.com 

This -event study qualitatively explored where EFL high-school students with intermediate-level proficiency would visit when they understand and interpret narratives accompanying with illustrations. Three slides including narrative text and accompanying illustrations represented a progress of the story structure from exposition, climax to resolution and the purpose of this study was to mine specific reading behavior from the first and the last ten changing points (five saccades) among 13 EFL readers who successfully comprehended the story. Based on a content analysis of saccade events and meaning units, in the slide one (exposition) the first and last five saccades overlapped at some meaning units on both text and illustrations. For the slides two (climax) and three (resolution), their first and last five saccades revealed that readers had different focuses on the text but with the overlapping areas of the illustration. It suggests when EFL intermediate-level readers interpreted narratives along a story structure, the effortful construction of a foundation for the emergence of a coherent mental representation took place at the early stage and illustration was used in bustling construction. When moving on to the climax and resolution, text information was added onto previous laid coherence foundation and illustrations were used as confirmation. 

**Usability, New Media and Visual Communication **

**Situational Modulation of Multimedia Processing Strategies **

**Fang Zhao**^1^**, Wolfgang Schnotz**^2^**, Inga Wagner**^2^**, Robert Gaschler**^1^****

^1^FernUniversität in Hagen, Germany; 

^2^University of -Landau fang.zhao@fernuni-hagen.de 

Placing questions before or after multimedia learning material constitutes different reading situations that stimulate learners to apply different processing strategies. The present study aims to investigate strategy differences regarding the usage of text and pictures triggered by such different reading situations when students from different school tiers and different grades try to answer questions of varying difficulty. Results of an eye tracking experiment with 144 students reveal that learners use text differently from pictures whereby this difference is moderated by the processing strategy. Learners mainly use text for mental model construction, whereas they mainly use pictures to search for task relevant information. As question difficulty increases, their emphasis on pictures increases more than their emphasis on text. Higher tier students focus more on pictures instead of text than lower tier students. Higher graders outperform lower graders mainly in text processing rather than picture processing. Usage of text and pictures as complementary sources of information occurs in a highly flexible manner according to the processing situation at hand. 

**Extraction of Semantic Saliency on Memory and Remembering during reading/searching information in the context of Web interaction**

**Véronique DRAI-ZERBIB**^1^**, Thierry BACCINO**^2^

^1^LEAD - University of Bourgogne France, France; 

^2^LUTIN - University Paris, France veronique.drai-zerbib@u-bourgogne.fr 

Collecting on combined web sites pages is a highly cognitive demanding task because or many types of information displayed (link or not with the topic we are focused on) and multimodal information (video, picture, text). Therefore, strategies of reading laying on semantic saliency may help the reader to find requested information and to not be disrupted by irrelevant information. This eye tracking study used 12 simplified websites 1) to assess whether semantic saliency influence ocular inspection of the pages and the subsequent recollection of information; 2) to attempt to further document the role source memories may have when integrating multiple documents/pages in line with the topic of the requested information. The Web pages contained blocs of text strongly associated, weakly associated or not associated to a target topic. 31 participants had to read for collecting information about a given topic, navigating in each website composed of 4 pages and then 1) to perform a memory awareness test (R/K – Tulving, 1985) and 2) to draft a summary on the given topic. The results show that semantic relatedness affects the navigation across the webpages, contribute to gaze guidance and recollection of information. 

**Typography and individual experience in digital reading: Do readers’ eye movements adapt to poor justification?**

**Julian Jarosch**^1^**, Matthias Schlesewsky**^2^**,Stephan Füssel**^1^**, Franziska Kretzschmar**^3^****

^1^Johannes Gutenberg-University Mainz, Germany; 

^2^University of South Australia, Australia; ^3^University of Cologne, Germany jjarosch@students.uni-mainz.de 

Justification as a major typographical variable interacting with reading proficiency (Zachrisson 1965) is less well studied for digital reading – although its often poor implementation in digital media differs from print, thereby currently transforming everyday reading experience. We recorded readers’ eye movements (N=40) while they read short narratives (5–10 lines), and manipulated interword spacing with varying degrees of deviation from standard. Participants were surveyed for reading experience (time spent reading, frequency of digital reading, ART), and performed a short reading-speed pre-test. Mixed-models analysis revealed that interword spacing mainly affected saccade planning, as fixation number increased with spacing. Readers also tended to land more on wider spaces, causing shorter mean fixations. Saccade amplitude increased with the distance between words, nevertheless covering fewer characters. As for reading time measures, only first pass time was slightly increased. The frequency of regressions remained unaffected – showing a stable net outcome of different reading processes. Importantly, individual experience in digital reading did not have a measurable impact on this pattern, whereas reading speed was a strong independent predictor. These findings suggest that oculomotor processes are unaffected by prior exposure to digital typography, and that reading proficiency determines reading strategies that are robust against typographical deviations. 

**A contrastive perception study of popular-scientific texts written by journalists vs. researchers**

**Silvia Hansen-Schirra**^1^**, Jean Nitzke**^1^**, Anke Tardel**^1^**, Christoph Böhmert**^2^**, Philipp Niemann**^2^****

^1^University of Mainz, Germany; 

^2^Karlsruhe Institute of Technology, Germany hansenss@uni-mainz.de 

This paper reports on linguistic and psycholinguistic insights into the readability and comprehensibility of German popular science texts comparing two different author groups. We show how text properties influence reading behavior as well as perceived comprehensibility. To do this, we analyse a sub-corpus of 20 popular-scientific articles written by journalists and 20 texts written by researchers. On the one hand, the texts can be ascribed to the journalistic domain because they are written for a lay public and should thus be interesting and easy to read. On the other hand, the texts describe and explain scientific topics, which are often difficult to comprehend and include a certain amount of specialized language (e.g. terminology). Our results show to which extent journalists and researchers adhere to their conventionalized writing styles, respectively, when dealing with complex topics. Moreover, we reveal how these different writing tyles affect the eye-tracking results of a homogeneous lay reader group. We will show fixation durations and fixation counts for the two reading corpora – based on more than 200 reading sessions. In addition, we discuss how these findings 

interact comprehensibility ratings as well as cognitive interviews and whether the readers exhibit a preferred author group. 

**Eye Response to Blockiness Artifacts in Video**

**Deepti Pappusetty, Hari Kalva**

Florida Atlantic University, United States of America dpappuse@fau.edu

Automatic assessment of subjective quality of a video is a challenging problem. Eye tracking has the potential to enable new approaches to video quality assessment and structure analysis. We conducted an analysis of gaze and attentional responses to videos with quality variations. Experiment was designed taking 10 director-driven movie videos (10-secs clips) and encoded them into good quality (30 Mbps bitrate) & bad quality (500 kbps bitrate) videos. The lower bitrate used for bad quality video produced strong blocking artifacts. These processed videos were shown to 20 participants in 4 groups. The experiments conducted were task-free to avoid TEPR (Task-evoked pupillary response). None of the videos were repeated for any participants to avoid any expectation bias. We chose director-driven movie videos where consistent salient regions are expected across subjects. Videos with strong blockiness create spurious motion and such motion could be leading to longer fixations and exploration of scenes. Viewer’s responses show significant difference in ‘Fixations/ Saccade’ Ratio (Sum of fixation times divided by trial duration) for Good Vs Bad. Statistical significance was tested using ‘Wilcoxon Signed-Rank Test' with p-value 0.02852; p<0.05. 

**Personalization in advertising: Effects of demographic targeting on visual attention **

**Kai Kaspar, Sarah Lucia Weber, Anne-Kathrin Wilbers**

University of Cologne, Germany kkaspar@uni-koeln.de**

Internet users often avoid looking at online advertisements as they have learned to actively ignore them. This established banner blindness threatens the effectiveness of advertising and counteracts the substantial global investments in this field. In this context, personalized advertising is expected to overcome banner blindness by attracting users’ attention to more self-relevant ad content. However, only little is known about users’ actual attention allocation during the exposure to webpages that include personalized versus non-personalized ads. We aimed to further fill this empirical gap and examined whether personalization in terms of demographic targeting has a positive effect on attention allocation. Moreover, we tested subsequent effects on brand attitude and website evaluation. Overall, eye tracking data of 49 participants revealed that personalization had a mediumto-large sized effect on several eye movement parameters (dwell time, number of entry fixations, number of fixations, and the mean duration of fixations), whereby the effect was moderated by the specific visual components of the multi-element banner ads. In contrast, personalization showed no effect on brand attitude and website evaluation. We conclude that personalization of ads significantly reduces banner blindness, but increased visual attention is not sufficient to trigger positive effects on the level of subjective judgments.

**Attention to brand logos during the first exposure to advertisements affects the neural correlates of recognition memory: An eye movement – ERP study Jaana Simola**

University of Helsinki, Finland jaana.simola@helsinki.fi

We recoded eye movements (EM) and electroencephalography (EEG) to investigate how attention during the first exposure to advertisements affects recognition of brand logos. On day 1, participants read 40 editorials presented together with an ad. Participants were divided into two groups based on their EMs during reading. Compared to the “Attention-” group (n = 11), the “Attention+” group (n = 11) attended the logos more in terms of the number and the probability of fixations and the total gaze duration on logos. In the recognition task, on day 2, participants indicated whether the logo was seen during reading. A frontal negative event-related potential (ERP) at 400–600 ms post-stimulus was larger for correctly rejected new logos than for the missed old logos in the “Attention+” group. No such difference was observed in “Attention-“ group. At the parietal site, a positive response at 400– 600 ms was larger for misses than for correct rejections in the “Attention+” group, while an opposite pattern was observed in the “Attention-“ group. The ERP responses, thus, provided a marker that differentiated between the two attention groups. Importantly, the difference between the unrecognized old logos and correctly rejected lure logos indicates an implicit memory for the old logos.

**Eye Movement Markers in Perceiving of Logos **

**Adel Adiatullin**^1^**, Marina Koroleva**^1^**, Victor Anisimov**^1,2^**, Alexander Latanov**^2^**, Natliya Galkina**^1^****

^1^Joint-Stock Company ‘Neurotrend’, Moscow, Russia; 

^2^Dept. of Higher Nervous Activity, M.V. Lomonosov State University, Moscow, Russia galkina@neurotrend.ru 

Logo is an detail in marketing of any serious modern company. At the same time there are still many questions related to how do some logos get advantage over the others drawing more attention of consumers and therefore affecting to final consumer's choice. 

In the first experiment 8 subjects watched neutral video in which 20 logos were presented sequentially every minute for 6 s each. Then these 20 logos and 40 others were presented for 15 within 25 s on each of four consecutive slides, while recording subjects’ eye movements. We found that the logos seen earlier presumably draw more attention because the subjects exhibited more fixations and longer dwell time on each of them compared to previously unseen logos. The second experiment was conducted in 50 subjects in the same manner as the first experiment. After logos presentation the subject were asked which logos they remembered to verify memorization. The subjects better remembered those logos that were presented in the video. Also they exhibited more fixations and longer dwell time on remembered logos compared to unremembered logos. Based on these results we built up the statistical predictive model that described interrelation between eye movement parameters and memorization efficacy.

**Understanding use of labelling information when preparing infant formula: an eye-tracking study****

**Lenka Malek**^1^**, Hazel Fowler**^2^**, Gillian Duffy**^2^****

^1^The University of Adelaide, Australia; 

^2^Food Standards Australia New Zealand ****lenka.malek@adelaide.edu.au 

Infant products are specially formulated to meet the nutritional needs of a vulnerable population. Caregivers’ ability to understand and follow preparation and storage instructions is therefore of high importance. This study aims to increase understanding of how Australian caregivers perceive, interpret and use mandatory and voluntary “on-package” labelling information when preparing and storing infant formula. An eye-tracking task requiring caregivers (n=30) to prepare an unfamiliar infant formula product while wearing Tobi Pro 2 Glasses revealed that almost all caregivers look at the preparation instructions (93%) and feeding guide (87%); fewer look at the warning advice (43%) and storage instructions (27%); and none look at the date-marking. The same trend was observed with respect to fixation duration. Findings from retrospective think-aloud and in-depth interviews conducted immediately after the eye-tracking task, revealed that while the instructions are generally understood, they are not always adhered to, with most caregivers making modifications for efficiency or convenience. Lack of awareness and low perceived risk to the infant’s health were other reasons for non-adherence. These findings suggest that mandated food-safety elements on infant formula products need to be clearer, more comprehensible and more effective, to ensure safe preparation and storage by all caregivers.

**Visual intake of price information of organic food – a shopping task with EyeTracking Glasses**

**Manika Rödiger, Ulrich Hamm**

University of Kassel, Germany

m.roediger@uni-kassel.de

Many studies found that the price of organic food is a major barrier to purchase. This study investigates the visual intake of price information of organic food, hypothesizing that depending on individual attitudes toward organic food prices, amongst others, consumers have different patterns of searching for price information. In November/December 2016, a consumer study was conducted in a city in central Germany. Wearing SMI Eye-Tracking Glasses, consumers performed a shopping task in a laboratory test market offering Fusilli noodles (two organic and four non-organic brands) and strawberry jam (two organic and four non-organic brands) at different prices. Afterwards participants answered a structured questionnaire via computer-assisted self-interviewing and received a monetary reward for their participation. By a combination of systematic and quota sampling (according to age and gender of the German population) 255 consumers were acquired for the study. The mapping of gaze data on reference pictures with SMI BeGaze software will be finished by the end of March 2017 as this is ‘work in progress’. Results will be presented on consumer segments clustered and described according to their attitudes toward organic food prices, socio-demographic characteristics and their visual intake of price information in the shopping task.

**The ‘objectfiying gaze’ - how it is affected by information on distribution of sexting images**

**Frederike Wenzlaff, Briken Peer, Dekker Arne**

University Medical Center Hamburg-Eppendorf, Germany

 f.wenzlaff@uke.de

The exchange of intimate photos through the internet (“sexting”) has become common, despite the risk of distribution to unintended audiences. We were interested how information about the consensual or non-consensual distribution influences the perception and evaluation of such images. One participant group was informed that the men and women distributed their pictures voluntarily. The other group was informed that the images were distributed against the will of the persons shown. Both groups rated attractiveness, intimacy as well as unpleasantness of further distribution for each image while eye movements were measured. In line with Objectification Theory we defined the 'objectifying gaze' as relatively longer dwell time on the body. This pattern was most pronounced by men who assumed non-consensual distribution. Also, for higher acceptance of myth about sexual aggression and for higher objectification tendencies, the relative dwell time on the body increased. Participants in the non-consensual condition rated further distribution as more unpleaseant for the depicted persons and women in this condition perceived the images as more intimate than men did. We demonstrated that the assumed way of distribution not only affects explicit ratings of the images but viewing behavior as well. These results are discussed in light of current theories. 

**Speed transformation function as a mean of improvement of gaze-based HCI**

**Dominik -Wachtel**^1^**, Cezary Biele**^1^**, Marek Młodożeniec**^1^**, Anna Niedzielska**^1^**, Jarosław **

**Kowalski**^1^**, Paweł Kobyliński**^1^**, Krzysztof Krejtz**^1^**, Andrew T. Duchowski**^2^

^1^National Information Processing Institute, Poland; 

^2^School of Computing, Clemson University, USA dchrzastowski@opi.org.pl

One of the possible reasons for low adoption of eye-trackers for human-computer interaction may be a difference in behavior of gaze-control in comparisons to traditional controls like mouse. Mouse control uses so called pointer acceleration that causes a pointer to move with a speed proportional to mouse movement speed. In our study we tested the effectiveness of similar feature - the speed transformation function based on the distance between the character and gaze location. We hypothesized that such function would facilitate interaction and improve visual scanning by making gaze control similar to input modalities more familiar to users. To test the hypothesis we developed a simple arcade game with which we tested several different transformation functions to determine how they change the subjective game ratings and game performance. The functions were designed to allow people to scan peripheries of visual field, guide the character through the game as well as use precise, small movements to correct the character position. We found out that speed transformation function improved the experience of gaze-controlled interaction and increased performance. Wider adoption of tested speed transformation function may lead to more interest in using eye-trackers in human-computer interaction and improve the precision of eye-input.

**Investigating gaze-controlled input in a cognitive selection test**

**Katja Gayraud**^1^**, Catrin Hasse**^1^**, Hinnerk Eißfeldt**^1^**, Sebastian Pannasch**^2^

^1^German Aerospace Center (DLR), Germany; 

^2^Technische Dresden, Germany katja.gayraud@dlr.de

In the field of aviation, there is a growing interest in developing more natural forms of interaction between operators and systems to enhance safety and efficiency. These efforts also include eye gaze as an input channel for human-machine interaction. The present study investigates the application of gazecontrolled input in a cognitive selection test called Eye Movement Conflict Detection Test. The test enables eye movements to be studied as an indicator for psychological test performance and uses eye gaze as an input modality. Participants have to detect potential conflicts between aircraft and mark them using gaze input. In order to differentiate between eye movements related to the conflict detection task and fixations as commands (Midas touch problem), conflicts are first selected (preactivated) and then marked (activated). Pre-activation is indicated by a color change from the border to the middle within the respective field. Unintended pre-activation can be interrupted by a saccade to another location. Different dwell times have been tested for pre-activation and activation in order to find an appropriate configuration for the participants. First results from pilot and air traffic controller applicants will be presented. The potential contribution of eye movements in the selection of aviation staff will be discussed. 

**The effect of visual signaling when reading to do**

**Michael Meng**

Merseburg University of Applied Sciences, Germany michael.meng@hs-merseburg.de

Numerous studies have shown that visual cues such as arrows or color coding can foster learning from multimedia materials (de Koning et al., 2009; Mayer, 2009). This study examines whether readers also benefit from visual signaling in procedural texts, such as software tutorials or manuals, which are typically not read to learn, but to immediately execute series of steps in order to complete a certain task. We designed three versions of a beginner’s tutorial for an image manipulation program that included (a) pictures (screenshots) with signaling elements, (b) the same pictures without signaling elements, or (c) text only. The tutorials were presented on a monitor alongside with the program in fixed position. Eye movements were recorded while participants (N=48) worked through one tutorial version and performed the tasks described there. Results show that accuracy of task execution is higher if pictures with signaling elements are used. Dwell time spent on pictures did not differ across conditions. However, picture areas relevant for a task attracted more fixations and longer fixation times if highlighted by visual cues. The results provide evidence that visual signaling successfully guides visual attention to relevant information in “reading-to-do” situations as well, thereby supporting effective task execution.

**Eye-Tracking-Based Attention Guidance in Mobile Augmented Reality Assistance Systems**

**Patrick Renner, Thies Pfeiffer**

Bielefeld University, CITEC, Germany prenner@techfak.uni-bielefeld.de 

Mobile assistance systems are currently a focus of increased interest, since augmented reality (AR) glasses have become more lightweight and powerful in the recent years. However, their displays still have a limited field-of-view (FOV) in which the real world can be overlaid with additional information. Thus, points-of-interest (POIs) can be augmentable (when aligned with the AR FOV), visible for user but not augmentable (outside the AR FOV but within the human FOV) or not visible at all. For this reason, it is often necessary to guide the user's attention to a specific POI. Our aim is to evaluate whether eye tracking in AR glasses could be used to notify the user that he is currently fixating a POI which is not augmentable without aligning the AR FOV with it. The underlying idea is that it is possible to peripherally perceive information on the AR display while fixating the POI. We present the results of two studies evaluating the general benefit of integrating eye tracking in an AR attention guidance system as well as comparing different ways of visualizing information to be perceived in the periphery.

**Usability Heuristics for Eye-Controlled User Interfaces**

**Korok Sengupta**^1^**, Chandan Kumar**^1^**, Steffen Staab**^1,2^

^1^University of Koblenz, Germany; ^2^University of Southampton, UK koroksengupta@uni-koblenz.de

Evolution of affordable assistive technologies like eye tracking helps people with motor disabilities to communicate with computers by eye-based interaction. Eye-controlled interface environments need to be specially built for better usability and accessibility of the content and should not be on interface layouts that are conducive to conventional mouse or touch-based interfaces. In this work we argue the need of the domain specific heuristic checklist for eye-controlled interfaces, which conforms to the usability, design principles and less demanding from cognitive load perspective. It focuses on the need to understand the product in use inside the gaze based environment and apply the heuristic guidelines for design and evaluation. We revisit Nielsen’s heuristic guidelines to acclimatize it to eye-tracking environment, and infer a questionnaire for the subjective assessment of eye-controlled user interfaces.

**CrowdPupil: A crowdsourced, pupil-center annotated image dataset**

**David G. de Gómez Pérez, Roman Bednarik** University of Eastern Finland, School of Computing dgil@uef.fi

In this paper, we present a dataset of pupil images and associated hand-annotated pupil centers, obtained through the method of crowd-sourcing. Acquisition of the points is explained and the dataset is presented. We present a comparison of two state-of-the-art pupil detection algorithms as a proposal towards public benchmarking of pupil detection algorithms. We invite the eye tracking community to test their own algorithms, share the results, and thereby advance the domain systematically. Finally, we present our plans for organizing a public pupil-detection challenge. 

**Robust, real-time eye movement classification for gaze interaction using finite state machines**

**Antonio Diaz-Tula, Carlos H. Morimoto**

University of Paulo, Brazil hitoshi@ime.usp.br

Fixations and saccades are commonly used in gaze-based interfaces. State-of-the-art algorithms for eye movement segmentation work well for high speed and accurate eye trackers, which are still too expensive and bulky for most gaze interaction applications. For low-end eye trackers running at 30 to 60 Hz and with accuracy of about 1 degree, such algorithms do not perform as well. We propose a robust, real-time method to classify eye movement data into four categories: fixations, saccades, drifts, and none. The classifier is based on a finite-state machine (FSM) and is robust to missing data and blinks. The approach first filters raw gaze data to recover missing samples and smoothes the data. The current filtered sample is then classified by computing spatial dispersion and absolute eye velocity using a small number of recent gaze samples and the current state of the machine. Qualitative evaluation have shown evidence that FSM reduces latency after blinks, reduces the number of re-focusing events and improves user experience during the interaction compared with a simple fixation detector based on a running average window. 

The source code is publicly available at https://bitbucket.org/diaztula/gaze_movements_fsm/. 

**Supervised Gaze Bias Correction for Gaze Coding in Interactions **

**Rémy Siegfried**^1,2^**, Jean-Marc Odobez**^1,2^****

^1^IDIAP Research Institute, Switzerland; 

^2^Ecole Polytechnique Fédérale de Lausanne, Switzer jean-marc.odobez@epfl.ch 

Understanding role of gaze in conversations and social interactions or exploiting it for HRI applications is an ongoing research subject. In these contexts, vision based eye trackers are preferred as they are non-invasive, allow people to behave more naturally. In particular, appearance based methods (ABM) are very promising, as they can perform online gaze estimation and have the potential to be head pose and person invariant, accommodate more situations, user mobility and resulting low resolution images, and are person as well as head pose invariants. However, they may also suffer from a lack of robustness when several of these challenges are jointly present. In this work, we address gaze coding in human-human interactions, and present a simple method based on a few manually annotated frames that is able to much reduce the error of an head pose invariant ABM method, as shown on a dataset of 6 interactions.

**Schau genau! A Gaze-Controlled 3D Game for Entertainment and Education**

**Raphael Menges**^1^**, Chandan Kumar**^1^**, Ulrich Wechselberger**^1^**, Christoph Schaefer**^2^**, Tina Walber**^2^**, Steffen Staab**^1^

^1^University Koblenz-Landau, Germany; 

^2^EYEVIDO GmbH, Germany raphaelmenges@uni-koblenz.de

Eye tracking devices have become affordable. However, they are still not very much present in everyday lives. To explore the feasibility of modern low-cost hardware in terms of reliability and usability for broad user groups, we present a gaze-controlled game in a standalone arcade box with a single physical buzzer for activation. The player controls an avatar in appearance of a butterfly, which flies over a meadow towards the horizon. Goal of the game is to collect spawning flowers by hitting them with the avatar, which increases the score. Three mappings of gaze on screen to world position of the avatar, featuring different levels of intelligence, have been defined and were randomly assigned to players. Both a survey after a session and the high score distribution are considered for evaluation of these control styles. An additional serious part of the game educates the players in flower species, who are rewarded with a point-multiplier for prior knowledge. During this part, gaze data on images is collected, which can be used for saliency calculations. Nearly 3000 completed game sessions were recorded on a state horticulture show in Germany, which demonstrates the impact and acceptability of this novel input technique among lay users. 

**Social Cognition, emotion and cultural factors**

**A Framework for Exploring the Social Gaze Space**

**Arne Hartz**^1,2^**, Mathis Jording**^3^**, Björn Guth**^1,2^**, Kai Vogeley**^3,4^**, Martin Schulte-Rüther**^1,2,4^

^1^Department of Child and Adolescent Psychiatry, Psychosomatics, and Psychotherapy, University Hospital RWTH Aachen, Germany; ^2^JARA Brain Translational Medicine, Research Center Jülich, 

Germany; ^3^Neuroimaging Group - Department of Psychiatry, University Hospital Cologne, Germany;

^4^Institute for Neuroscience and Medicine – Cognitive Neuroscience, Research Center Jülich, Germany ahartz@ukaachen.de 

Introduction: communication has a high dimensional and procedural complexity and is usually produced and perceived automatically and unconsciously, but a comprehensive understanding of gaze-based nonverbal communication is lacking. We introduce a novel technical setup for investigating the Social Gaze Space, an umbrella term for different types of interactions mediated by gaze behavior in triadic interactions. Methods: We have specified and validated different agent’s states as 1. partner-oriented 2. object-oriented 3. introspective 4. initiating joint attention 5. responding joint attention These states differ in temporal duration, frequency, and responsiveness of/to gaze shifts/directions. In first empirical studies, we have determined corresponding parameters to ensure ecological validity. A Tobii TX300 eyetracker allows for a chinrest-free setup, the algorithms are written in Python using PyGaze. Research questions We are investigating social interaction across the lifespan and psychiatric conditions, focusing on Autism and age: Do temporal parameters in gaze interaction differ among participant groups? Does detectability of the agent’s state depend on the group membership of a participant? How is autonomous reflexive behavior influenced by the agent’s and participant’s “states”? Conclusion: This work is essential for the development of ecologically valid interaction platforms, facilitating the development of virtual agents for therapeutic settings. 

**Visual Exploration of Social Stimuli – Comparisons of Patients with**

**ADHD or Autism and Healthy Controls**

**Chara Ioannou**^1^**, Divya Seernani**^1^**, Holger Hill**^2^**, Giuseppe Boccignone**^3^**, Tom Foulsham**^4^**, Monica Biscaldi-Schäfer**^1^**, Christopher Saville**^5^**, Ulrich Ebner-Priemer**^2^**, Christian Fleischhaker**^1^**, Christoph **

**Klein**^1,5^

^1^Department of Child and Adolescent Psychiatry, Medical Faculty, University of Freiburg, Germany; 

^2^Institute of Sports and Sports Sciences, Karlsruhe Institute of Technology, Germany; ^3^Department of Computer Science, University of Milan, Italy; ^4^Department of Psychology, University of Essex, 

England; ^5^School of Psychology, Bangor University, North Wales, United Kingdom; ^6^Department of 

Child and Adolescent Psychiatry, Medical Faculty, University of Cologne, Germany chara.ioannou@uniklinik-freiburg.de 

While observable social deficits are among the obligatory DSM 5 criteria for diagnosing Autism Spectrum Disorder (ASD), their empirical verification through the analysis of gaze movement patterns of social attention has proven difficult. According to a recent meta-analysis, one of the main abnormalities of ASD patients is processing of social complexity (Chita-Tegmark, 2016). The present study aims to elucidate the impact of social complexity on gaze movement patterns of ASD patients in comparison with ADHD patients, supposed to share aetiological factors (Rommelse et al., 2011). Four images with two levels of social complexity – one person versus four people – are presented, for 120 sec each, to children and adolescents with ADHD, ASD and healthy controls, aged 10-13 years (N=90; all native German speakers). 

**Eye movement patterns in response to social and non-social cues**

**Claudia Bonmassar, Francesco Pavani , Wieske van Zoest**

University of Trento, Italy claudia.bonmassar@unitn.it

Gaze and arrow cues cause covert shifts of attention even when they are uninformative. We investigated to what extent oculomotor behaviour helps to explain manual response biases to social and non-social stimuli. We tracked the gaze of 20 participants while performing the cueing task with uninformative cues (gaze vs. arrow), SOA (250 vs. 750 ms) and validity (valid vs. invalid) as within-subject factors. Our results confirmed previous behavioural findings and showed participants were faster when the gaze or arrow cue was correctly directed towards the target. Analyses of initial saccades showed anticipatory movements in response to the cue which were larger in the longer compared to the short SOA condition. Once the target appeared, the eyes fixated closer to the valid target location than to the invalid target location; however, while this happened for both SOAs in gaze-cues, arrow-cues triggered this oculomotor behaviour only in the longer SOA. Moreover, both ‘cue-triggered’ and ‘target-triggered’ responses revealed a right-side bias such that eye movements were larger to cues pointing to the right than to the left. This work provides novel insight in the relation between attention and eye movements in response to social and non-social cues.

**Oculomotor control in social and non-social information processing contexts **

**Eva K. Riechelmann, Anne Böckler , Tim Raettig , Lynn Huestegge**

Universität Würzburg, Germany eva.riechelmann@uni-wuerzburg.de 

Efficient gaze control is assumed to be associated with the anticipation of its effects (ideomotor control theory), which requires the acquisition of learned associations between saccades and their visual effects. However, only few eye movement studies have addressed the underlying mechanisms of this phenomenon. While previous research predominantly focused on the investigation of non-social effect signals, the present study incorporated social (faces that respond to the participant’s gaze with either direct vs. averted gaze) and non-social targets. Two eye-tracking experiments investigated whether social information processing in the anticipation of saccadic action-effects is special, and focused on the impact of exogenously vs. endogenously triggered saccades when acquiring action-effect associations. To examine the occurrence of anticipation, both experiments included congruency manipulations to prime or interfere with any anticipated representation of the subsequent effect signal. We hypothesized to observe congruency effects for both social and non-social stimuli, with different temporal dynamics for social stimuli. The anticipated gaze type (direct vs. averted) was predicted to affect behavior in terms of a facilitating approach signal in the case of the potentially rewarding (direct gaze) stimulus. Data collection is ongoing. Our results will contribute to a better understanding of gaze control mechanisms and social gaze interaction. 

**Understanding social interaction and social presence of others using simultaneous eye tracking of two people: Behavioral Data**

**Haruka Nakamura, Seiya Kamiya , Takako Yoshida**

Tokyo of Technology, Japan nakamura.h.ao@m.titech.ac.jp 

How the sensation “a live human is looking at me” changes our eye behavior including eye movement? How about when they make eye contact via live video chat system? To answer these, we built a system wherein a pair of participants looked at each other’s faces via video chat like display, and we tracked their eye movements simultaneously. Three conditions were tested: real-time face-to face observation (RT), recorded face observations (RF), and static face picture observations (SF) for 30 sec. Participants had to watch the video and judge whether they observed “Live” or “Not Live” video. Comparison of results between RT and RF showed no significant difference in the ratio to respond “Live,” etc., while they were significantly different from SF conditions, suggesting that participants could not tell the difference between RT and RF. When data were classified based on the response types or “Live”/“Not Live,” less fixations around eyes were observed for “Live” response, and for “Live” response trials, less fixations around eyes were observed for another participant in the video. This showed that decision process to judge whether somebody is looking at me in real-time or depended on other person’s behavior related to avoiding eye contacts. 

**Gender differences in natural viewing behavior?**

**Marco Rüth, Anne-Kathrin Wilbers, Daniel Zimmermann, Kai Kaspar** University of Cologne, Germany marco.rueth@uni-koeln.de 

Previous research showed gender differences in viewing behavior on sexual stimuli and photographs of human actors. However, can we generalize such gender differences to other types of visual stimuli? This is a central but hitherto neglected question: In particular, numerous studies use complex scenes to investigate viewing behavior under “natural” conditions. Thereby, most studies use convenience samples including a strong gender bias. Critically, this might bias parameter estimation based on mean scores calculated across all participants of the sample. We investigated whether women and men differ in common eye-tracking parameters when freely observing complex scenes of seven different categories. 106 participants (57 female) observed 140 images while eye movements were recorded. Several personality traits and participants’ current emotional state were initially measured and used as covariates in the final analyses. Also, participants rated how much they liked each of the images in a separate session. Overall, we found gender differences in image evaluation concerning two categories but no differences in viewing behavior after controlling for personality traits and emotional states. However, we replicated common effects of image type on eye movement parameters. Hence, an unbalanced gender ratio seems to be no serious problem in some parts of the eye-tracking literature.

**Does our native language determine what we pay attention to? A crosslinguistic study of gaze behaviour between Korean and German speakers **

**Florian Goller**^1^**, Ulrich Ansorge**^1^**, Soonja Choi**^1,2^

^1^University of Vienna, Austria; 

^2^San Diego State University, USA florian.goller@univie.ac.at 

Languages differ in how they categorise spatial relations: While German differentiates between containment (in) and support (auf) with distinct spatial words – (a) den Kuli IN die Kappe (‘put pen in cap’); (b) die Kappe AUF den Kuli stecken (‘put cap on pen’) –, Korean uses a single spatial word (KKITA) collapsing across (a) and (b) into one semantic category, particularly when the spatial enclosure is tightfit. Korean uses a different word (e.g., NEHTA) for loose-fits (e.g., apple in bowl). In a cross-cultural study, we compared German speakers with Korean speakers. Participants rated the similarity of two videos of several scenes where two objects were joined/nested (either in a tight or loose manner). The rating data show that Korean speakers base their judgement of similarity more on tight versus loose fit, whereas German speakers base their judgements more on containment and support (in vs. auf). Throughout the experiment, we also measured participants’ eye movements. Korean speakers looked equally often at the moving figure object and the stationary ground object equally often, whereas German speakers were more biased to look at the ground object. Additionally, Korean speakers also fixated more on the region where the two objects touched than did German speakers.

**Social influence on face perception in different ethnicities – An eye tracking study in a free viewing scenario**

**Jonas D. Großekathöfer, Matthias Gamer**

Department of , University of Würzburg, Germany jonas.grossekathoefer@uni-wuerzburg.de

Do people approach faces of different ethnicities in the same way? No, at least according to research on the own-race bias (ORB). The ORB describes that recognizing and discriminating own-race faces is enhanced compared to other-race faces. In this eye-tracking study, Caucasian participants freely explored two pictures of faces at a time (Caucasian vs. Arabic-Muslim). We examined the total fixation duration per face as well as specific predefined Regions-of-Interests (ROIs: glabella, eye and mouth region). Regarding the global fixation duration, male faces were looked at shorter when they belonged to the other-race. Interestingly this effect was reversed for female faces. However, comparison of the ROIs revealed that glabella and eyes were cumulatively fixated longer in male faces. Especially the eyes were more fixated in male own-race faces, which was again reversed in female faces. Participant’s gender had no influence on the fixation duration. Neither explicit measures (e.g., life satisfaction, contact to refugees) nor implicit measures (here: negative implicit attitudes towards Muslims) correlated with gaze behavior. The implication of these findings for social attention, face perception and cultural differences are discussed. The current findings open several interesting avenues for future research on the interplay between social characteristics and attentional processes. 

**Psychopaths show a reduced tendency to look at the eyes while categorizing emotional faces**

**Nina A. Gehrer, Jonathan Scheeff, Aiste Jusyte, Michael Schönenberg**

University of Tuebingen, Germany nina.gehrer@uni-tuebingen.de

Impairments in facial emotion recognition are postulated to contribute to the development and maintenance of antisocial and psychopathic behavior in prone individuals. There is some evidence suggesting that this deficiency could be due to reduced attention to the eye region of emotional faces. The eyes have been shown to automatically attract attention and provide crucial information for decoding emotional expressions. Previous studies already linked psychopathic traits in healthy individuals to a reduced tendency to shift attention to the eye region of faces. To date, no study investigated this relationship in incarcerated psychopathic populations. In our study, psychopathic (N=20) and non-psychopathic (N=16) violent offenders were asked to categorize faces while their eye movements were recorded. The faces either expressed one of the six basic emotions: Happiness, sadness, fear, anger, disgust, and surprise, or displayed a neutral expression. In line with the previous findings in healthy samples, psychopaths showed a reduced tendency to focus on the eye region of emotional faces when compared to the non-psychopathic offenders (i.e. less frequent initial fixations on the eyes and shorter dwell time on the eyes). Implications of the current findings for existing theory are discussed along with directions for future research.****

**Perceiver’s sensitivity and lateralization bias in the detection of posed and genuine facial emotions in movie clips: eye tracking study.**

**Katerina Lukasova**^1^**, Yuri Busin**^2^**, Manish Kumar Asthana**^3^**, Elizeu Coutinho Macedo**^2^

^1^UFABC, Sao Paulo, Brazil; 

^2^Social and Cognitive Neuroscience Laboratory, Mackenzie Presbyterian University, Brazil; ^3^Department of Humanities and Social Sciences, Indian Institute of Technology Kanpur, India katerinaluka@gmail.com

The aim of this study was to assess veracity (genuine vs posed) and eye tracking pattern of basic emotions (happy, sad and fear) in human faces showed in dynamic video clips. The faces were showed from left side 45o view and inverted right side view in order to generate lateralization bias. Forty-eight participants were assessed and had the eye movements recorded with Eye Gaze Edge 1750 eye tracker (LC Technologies, Inc.) with a recording frequency of 120Hz. Each clip appeared four times, constituting a total of 96 clips presented in two blocks. A 3-way ANOVA showed a significant main effect for the judgment in emotions (F(2,34)=3.71, p=.03) more accurate in happy faces compared to sad and fear and veracity (F(1,34)=7.66, p=.01) better in genuine emotions. Less fixations were made on genuine emotions (F(1,43)=4.82, p=.03) but the mean fixation time was longer for emotion (in sad and fear compared to happy, F(2,42)=4.38, p=.02), veracity (in posed compared to genuine, F(1,43)=5.98, p=.02) and side (in right side face view compared to left side, F(1,43)=5.14, p=.03). Based on the results, in dynamic video clips, the visual processing of facial cues is differently affected by viewing side and veracity of the emotion.

**Implicit Negative Affect Predicts Attention for Sad Faces beyond Self-Reported **

**Depression – An Eye Tracking Study**

**Charlott M. Bodenschatz , Marija Skopinceva, Anette Kersting , Thomas Suslow**

University of Leipzig, Germany charlott.bodenschatz@medizin.uni-leipzig.de

Cognitive theories of depression assume biased attention towards mood-congruent information as a central vulnerability and maintaining factor. Among other symptoms, depression is characterized by excessive negative affect (NA). However, little is known about the impact of NA on the allocation of attention to emotional information. NA can be measured using implicit and explicit assessment methods, whereby implicit affect has been found to be more predictive of spontaneous physiological reactions than explicit measures. The present study examined the link between implicit and explicit measures of NA, depression and attentional biases in a sample of healthy individuals (N = 105). Attentional biases were assessed using eye tracking during a passive viewing task. Participants viewed 20 slides, each depicting sad, angry, happy and neutral facial expressions. Higher levels of depression symptoms were associated with sustained attention to sad faces as well as reduced attention to happy faces. After controlling for depression symptoms, higher levels of implicit NA, but not explicit NA, significantly predicted gaze behavior towards sad faces. The present study supports the idea that gaze allocation to emotional facial expression is associated with implicit NA. Moreover, the findings demonstrate the utility of implicit affectivity measures in studying individual differences in visual attention.

**Gender differences in eye movement patterns during facial expression recognition**

**Elizaveta **^1^**, Natalia Malysheva**^1^**, Jahan Ganizada**^2^

^1^Lomonosov Moscow State University, Russian Federation; ^2^Lomonosov Moscow State University Baku Branch, Azerbaijan eluniakova@gmail.com

Adults are experts in the recognition of basic emotional expressions, but females do it more accurately compared to males (McClure, 2000). This may occur because women and men could rely on different mechanisms of face perception, namely females are more successful using feature-based processing and extraction of second-order relations (the distances among face features) so than males. Recognition of facial expression in female and male adults was studied applying eye tracking technology. Upright, inversed and Thatcherized stimuli were used. Three sets of photos of 2 male and 2 female faces from WSEFEP (Olszanowski et al., 2015 doi: 10.3389/fpsyg.2014.01516) each displaying seven facial expressions (neutrality, anger, fear, disgust, happiness, surprise and sadness) were randomly presented to each participant. Women were more accurate than men in the expression recognition of Thatcherized images, that evidenced a better feature processing in females compared to males. The results showed some differences in fixation patterns between two participant groups. Women looked more at the eyes and shifted more fixations between internal facial features compared to men, suggesting more featurebased processing and extracting information about second-order relations. Men made more fixations on eyebrows, nose bridge and external facial features than women, suggesting more holistic processing.****

Analyzing Emotional Facial Expressions’ Neural Correlates Using

**Event-Related Potentials and Eye Fixation-Related Potentials **

**Emmanuelle Kristensen**^1,2^**, Raphaëlle, N. Roy**^4^**, Bertrand Rivet**^1,2^**, Anna Tcherkassof**^1,3^**, Anne Guérin-**

**Dugué**^1,2^

^1^Université Grenoble Alpes, France; 

^2^Gipsa-Lab, CNRS, Grenoble, France; 

^3^LIP–PC2S, , France; 4: ISAE-SUPAERO, Toulouse, France

emmanuelle.kristensen@grenoble-inp.org

The processing of emotional facial expressions (EFE) elicits specific evoked brain responses reflecting different stages of the EFE processing. Here, we focus on the Late Positive Potential (LPP; around 500ms) as a marker of an elaborative processing and conscious recognition of EFE involving the working memory. But at this latency, during visual exploration, the Event-Related Potential (ERP) at the stimuli onset and the Eye Fixation-Related Potential (EFRP) at the first fixation onset overlap. Using a General Linear Model, these potentials can be identified separately. Methods. Twenty-four participants were asked to freely empathize with the presented EFE (70 natural but standardized EFE -Neutral, Disgust, Surprise, Happiness- before categorizing them. Results. Around 200ms, early posterior neural activities of the first EFRP were modulated by EFE (Happiness vs Disgust). During the LPP latency, this modulation provided by cognitive processing from this fixation onset (mean 275ms), strengthened an activities pattern at left frontal sites -more involved for positive EFE- becoming significant across EFE. In contrast, at right frontal sites -more involved for negative EFE, another activities pattern, only elicited by the stimuli presentation, was significant across EFE. Moreover, taken together, these findings are in line with faster and facilitated perceptual processing for negative EFE. 

**Affective and Cognitive Influences of Aesthetic Appeal of Texts on Oculomotor **

**Parameters**

**Hideyuki Hoshi**

Max Planck Institute for Empirical Aesthetics, Germany hideyuki.hoshi@aesthetics.mpg.de

This study investigated the eye-tracking data patterns that reflect the aesthetic appeal of short rhetorical sentences (proverbs). Participants read German proverbs either in the original or in a modified version from which meter, rhyme, and pronounced rhetorical brevitas (shortness) were removed. During reading these one-line sentences, pupil size and eye-movement were recorded simultaneously by using eye-tracking. Individual aesthetic ratings were collected afterwards, and the relations between stimulus complexity, aesthetic rating scores and eye-tracking datasets were analysed. A factor analysis extracted two underlying factors from the rating scores, which captured affective and cognitive dimensions of the aesthetic appeal of the text. A polynomial-curve fitting of the pupillary response and following regression analysis (linear-mixed-effect model) revealed that the affective and cognitive properties modulated the oculomotor parameters (fixations and pupil size) significantly and antagonistically. Higher scores on the extracted affective factor predicted more fixations and larger pupil dilation, whereas higher scores on the cognitive factor predicted fewer fixations and smaller pupil dilation (Table S1). The study identified the correlates of the affective and cognitive responses to the texts in the oculomotor parameters, and shows a possible application of the eye tracking method for capturing the aesthetic evaluation of literature during online reading.****

**The eye movement examination on achievement emotion images**

**Chia Yueh Chang, Sunny SJ Lin**

Institute of Education, National Chiao Tung University, Taiwan rainbowchang0226@gmail.com

This study based on Pekrun’s Achievement Emotion theory (2002) to design achievement emotion images which includes emoticons of nine emotions, enjoyment, hope, pride, relief, anger, anxiety, shame, hopelessness and boredom. These emotions could be categorized according to their valance (positive-negative), levels of activation (activating-deactivating) and object-focus (activity, outcomeprospective and outcome-retrospective). For each emotion, 5 images were drawn and evaluated. We adopted eye movement technique to identify the relative importance of the four Area of Interest (eyes, mouth, gesture, and decorations) in recognizing the images. Fifteen graduate students were invited to participate. Eye Link 1000 was used to collect eye data in looking on the emotion images. Several indicators including fixation- and saccade-based data were used for the analyses. Across all images, the majority of attention was placed at the AOIs of eye and mouth; while less attention was placed on either gesture or decoration, depending on the feature of the emotions. Emotions of positive valance were less distinguishable among each other so that the decoration and gesture AOIs became the conformation clues to help judgement. As for the 3 negative emotions (shame, hopelessness and boredom), they were more distinctive; therefore the decoration and gesture AOIs were less needed for comprehension.

**Space scanning patterns in impulsive and reflective subjects**

**Anna , Irina Blinnikova**

Lomonosov Moscow State University, Russian Federation mayoran@mail.ru

In the current study we analysed the influence of cognitive style on visual search process in a modelled graphical interface environment. The participants had to find the target stimulus in a 9x9 matrix with 81 images, commonly used in web design. Search time and eye movement data were recorded. The subjects were divided into two groups according to their Matching familiar figures test score: impulsive and reflective (Kagan, 1966; Carretero-Dios et al., 2008). Impulsive subjects tended to find the stimulus faster than reflective subjects (F(2;1983)=5.1;p<0.05), demonstrated shorter mean fixation duration (F(2;1983)=3.5;p<0.05) and shorter dwell time on the areas of interest (F(2;1983)=7.1;p<0.05). Furthermore, we identified sequential and non-sequential visual search patterns, using the combination of intersaccadic angle and saccade direction measures (Amor et al., 2016; Blinnikova, Izmalkova, 2017). We opted a three cluster solution with two sequential patterns, characterized by prevalence of smaller intersaccadic angles (mostly 0°-45°), which differed in prevailing saccade direction (horizontal or vertical), and one non-sequential pattern, characterized by larger amount of intersaccadic angles with 45°-135° values. Significant distinctions were found in Impulsivity score in different patterns (F(3;1983)=4.7 (p<0.05)): impulsive subjects tended to demonstrate non-sequential pattern and reflective subjects preferred sequential patterns, especially the horizontal sequential pattern

**Correlations between eye movements and personality traits**

**Anne-Kathrin Wilbers, Kai Kaspar**

University of Cologne, Germany

a.wilbers@uni-koeln.de

Time-independent personality traits have been widely neglected in eye movement research, but recent studies indicate that inter-individual differences may be systematically associated with differences in gaze behavior. In an ongoing eye tracking study, we investigate the relation between personality factors (Big Five and Behavioral Inhibition/Activation System) and eye movements. We created a new set of stimuli with a fearful target face at the center and peripheral cues including neutral and emotionally arousing scenes. Initial results indicate that the extent of neuroticism and behavioral inhibition negatively correlate with dwelling time in general. Conscientiousness correlates negatively with the percent of dwelling time on the eye region of the target face while agreeableness correlates negatively with dwelling time on the nose and the mouth. Moreover, in linear models the Big Five explain 7% (adjusted R²) of the variance in the duration of the first fixation located at the target faces with extraversion and agreeableness being the best predictors, while BIS/BAS explain 12% in the revisits on the peripheral cues. These results show the influence of top-down processes on visual attention and suggest a connection between gaze behavior and personality traits.****

Session II - , August 22^nd^, 15:30-17:00 

**Smooth pursuit eye movements **

**Saliency coding in superior colliculus during smooth pursuit eye movements**

**Brian White, Jing Chen, Karl Gegenfurtner, Douglas Munoz**

Queen's University, Centre for Nueroscience Studies, Canada

Justus Liebig Universität Giessen, Germany brian.white@queensu.ca

Theories/models of saliency postulate that visual input is transformed into a topographic representation of visual conspicuity, whereby certain stimuli stand out from others based on low-level features. Our recent research revealed evidence of an evolutionarily old saliency map in the superficialvisual-layers of the superior colliculus (SCs), a midbrain structure associated with the control of attention/gaze. However, little is known about visual representations during smooth pursuit, so we examined saliency coding in the SC during pursuit. Rhesus monkeys smoothly pursued a moving stimulus while we presented a stationary wide-field array of task-irrelevant stimuli that extended beyond the classic-RF, and contained a salient oddball. The pursuit stimulus moved across the array orthogonal to the neuron’s receptive field (RF), which was drawn over the oddball during pursuit. We found that SCs neurons signaled the presence of the salient-but-irrelevant stimulus, as evidenced by an increase then decrease in activation as the RF moved across the oddball, relative to other items. For intermediate-layer-visuomotor SC neurons, we did not observe significant modulation from the oddball. These results extend our previous research by showing that SCs continues to encode, and dynamically update, the saliency map while gaze actively tracks moving task-relevant stimuli in complex scenes. 

**Analysis of superior colliculus receptive fields during smooth pursuit eye movements **

**Jing , Brian White, Karl Gegenfurtner, Doug Munoz** Justus-Liebig-Universität Gießen, Germany; Queen's University, Canada jing.chen@uni-giessen.de

Smooth pursuit induce anticipatory attention shift in the direction of pursuit. The present study examined its potential neural mechanisms in the superior colliculus (SC), a midbrain structure linked to the control of gaze and attention. To this end, we analyzed the receptive fields (RF) of local field potentials (LFPs) and single neurons in SC during pursuit. Monkeys followed with gaze a moving target (15deg/s), while a salient task-irrelevant peripheral stimulus remained stationary on the screen. The peripheral stimulus was positioned orthogonally to the direction of pursuit such that it was brought into, then out of, the RF by pursuit. We compared responses during the entering and exiting phases to estimate the hypothesized anticipatory RF bias. We observed a bias in the RF in the direction of pursuit in the LFPs, providing tentative support for our hypothesis. This seems to be driven by the fact that the LFP profile goes through a process of positive inflection followed by a negative inflection as the RF is drawn over the stimulus. We did not observe a significant bias in single unit spiking activities, which suggests that the anticipatory responses are computed upstream, and/or inhibited from being manifested in the spiking activity. 

**Doing Smooth Pursuit paradigms in Windows 7**

**Inge Linda Wilms**

University of Copenhagen, Denmark inge.wilms@psy.ku.dk 

Smooth pursuit eye movements are interesting to study as they reflect a subject’s ability to predict movement of external targets, keep focus and move the eyes appropriately. The process of smooth pursuit requires collaboration between several systems of the brain and the resulting action may predict strength or deficits in perception and attention. However, smooth pursuit movements have been difficult to study and very little normative data is available for smooth pursuit performance in children and adults. This talk describes the challenges in setting up a smooth pursuit paradigm in windows with live capturing of eye movements using a Tobii TX300 eye tracker. In particular, the talk will describe the challenges and limitations presented by hardware and software in creating a smooth movement to track in a windows 7 environment. Also, the talk will present one way of quantifying the resulting raw data into manageable component for later statistical comparison and analysis. Furthermore, the normative results from a study comparing smooth pursuit ability in children and adults will be presented. It will detail some of the challenges generating smooth pursuit paradigms in windows and how to quantify the results for comparison and analysis.

**Predictable motion on a Necker cube leads to micro-pursuit-like eye movements and affects the dynamics of bistability.**

**Kevin M. A. Parisot, Alan Chauvin, Anne Guérin, Ronald P.**^,^**S. Zozor**

Gipsa-lab, France; LPNC, France; Université Grenoble Alpes, France; Grenoble INP, France; CNRS,

France

kevin.parisot@gipsa-lab.fr

Multistable perception occurs when a single, but ambiguous stimulus drives perceptual alternations. Understanding its mechanisms has a direct impact on perceptual inference and decision making. A model proposed by Shpiro and colleagues explains the dynamics of bistable perception by neural adaptation and driving noise. The action of adaptation and noise on competing neuronal populations— each encoding a perceptual representation—results in perceptual reversals. Goal. To test effects of noise and adaptation on perceptual reversal speed. Methods. We manipulate noise and adaptation using predictability of the retinal projection of the stimulus. A Necker cube was presented to 16 observers instructed to gaze at a central fixation cross while reporting their perceptual changes by key press. The stimulus followed either a smooth, predictable motion; a pseudo-random motion; or no motion at all (control). Our hypotheses predicted higher (lower) reversal speeds for low (high) predictable motion w.r.t. no motion. Results. Key press analysis validated our hypothesis for unpredictable motion, but not for predictable motion. We explain the latter by quantifying correlations between stimulus and gaze positions. This shows that observers executed micro-pursuit-like movements under predictable stimulus motion, thereby increasing the effect of adaptation on reversal speed w.r.t. our hypothesis. 

**Manual & Automatic Detection of Smooth Pursuit in Dynamic Natural Scenes**

**Mikhail Startsev, Ioannis Agtzidis, Michael Dorr**

Technical University Munich, Germany mikhail.startsev@tum.de

To understand gaze behaviour, we need to abstract from the raw point-of-regard data and segment the gaze trace into eye movement (EM) types. For static stimuli, these are typically limited to fixations and saccades, but dynamic stimuli may induce smooth pursuit (SP) as well. Detecting SP on naturalistic videos is challenging because the SP targets and their trajectories are not known a priori, and SP episodes may be short (average uninterrupted episode duration in hand-labelled data is 0.41s) and have speeds not much greater than both oculomotor and tracker noise around fixations. We previously developed an algorithm that uses information from several observers to address these challenges, and a preliminary evaluation showed excellent performance compared to state-of-the-art methods. To more thoroughly evaluate its performance, we now collected a manually annotated “ground truth” for the entire GazeCom dataset (more than 4.5 viewing hours) from 2 annotators and 1 tie-breaker. Prior to any parameter tuning, our detection algorithm achieves precision and recall of 74.2% and 46.4%, respectively. As part of the pipeline, we also detect fixations, with precision and recall of 91.3% and 90.2%. A Python implementation of the classification tool and the annotated dataset are publicly available at http://www.michaeldorr.de/smoothpursuit.

**Spatiotemporal EEG Source Localization during Smooth Pursuit Eye Movement by Use of Equivalent Dipole Source Localization Method**

**Takahiro Yamanoi, Tomoko Yonemura, Hisashi Toyoshima**

Meikai Hokkai-Gakuen University

Japan Technical Software yamanoi@hgu.jp

A linear moving white full circle on a CRT display was presented to subjects. Moving patterns were downward, upward, to the right and left. Subjects were requested to trace the stimulus. Meantime, electroencephalograms (EEG) were recorded. The EEG was summed in each movement and the equivalent current dipole localization (ECDL) was done to estimate the source in the brain. As results, the dipoles were localized to the V5 at latency of approximately 143ms, and after to the intraparietal sulcus (IPS, 162ms), to precentral gyrus (PrCG, 224ms) to the frontal eye field (FEF, 236ms) and to the superior colliculus (SC, 248ms). The direction of estimated dipole corresponded with the opposite movements. And the dipole to the superior colliculus was estimated, this organ is supposed to correspond with the eye movement. Also a dorsal pathway and a ventral pathway were found. 

**Visual transient onsets decrease initial smooth pursuit velocity and inhibit the triggering of catch-up saccades**

**Antimo Buonocore**^1,2^**, Ziad M. Hafed**^1,2^****

^1^Werner Reichardt Centre for Integrative Neuroscience, Tübingen University, Germany; ^2^Hertie Institute for Clinical Brain Research, Tübingen University, Germany antimo.buonocore@cin.uni-tuebingen.de 

Smooth synergistically interacts with catch-up saccades during tracking. While pursuit and saccades share neural mechanisms, details of their interactions are not fully resolved. Here we explored the effects of visual transients on pursuit initiation and catch-up saccades. In Experiment 1, a spot moved in one of four directions at ~27 deg/s. After 44-176 ms from motion onset, a high-contrast 1-deg square appeared for ~11 ms ~8 deg in front of or behind the spot. Experiment 2 was identical except that we used step-ramp motion trajectories to obtain saccade-free pursuit initiation. During such saccade-free initiation, pursuit velocity was lower with flashes during movement preparation (up to ~60 ms after motion onset) as opposed to later. Interestingly, in Experiment 1 with catch-up saccades during pursuit initiation, the early flashes also caused strong saccadic inhibition. We extended these saccadic effects to sustained pursuit by testing two monkeys tracking a horizontally-moving spot (~14 deg/s). Similar saccadic inhibition occurred, supporting the hypothesis of a resetting mechanism time-locked to flash onset, and affecting both saccadic and smooth velocity control systems. Based on the saccade-free pursuit initiation results in particular, we hypothesize that neural loci for saccadic inhibition would also impact smooth eye movements.

**Visual Search, Scanpaths and Scene Perception**

**Searching for real objects in a natural environment: The role of contextual semantic cues and incidental encoding in older and young viewers**

**Hanane Ramzaoui, Sylvane Faure, Sara Spotorno**

Laboratoire d’Anthropologie et de Psychologie Cliniques, Cognitives et Sociales, Université Côte d'Azur, 

France; Institute of Neuroscience and Psychology, University of Glasgow, UK hanane.ramzaoui@unice.fr

In everyday life, we often search for different targets in the same environment. Our study utilised object arrays on cluttered tables in a real room, requiring older and young healthy adults to search for four targets in each table. Each target name was given only after the previous target had been found (four trials per table). We recorded oculomotor behaviour with SMI eye-tracking glasses. We examined the potential benefit from prior fixations on a target when it was a distractor in the preceding trials, and the influence of semantic relatedness between each target and its neighbouring objects. The results showed quicker search for previously fixated targets, suggesting that incidental information gathering from an object leads to a representation binding identity and position, which may be a source of guidance during subsequent search. We also found that targets were located faster when surrounded by semantically related distractors (e.g., teacup near sugar and spoon) than when surrounded by unrelated distractors. This effect was stronger in older than young viewers, suggesting that reliance upon expectations and object-to-object associations increases with age and that contextual semantic cues may be used to improve search strategies in older viewers, counteracting speed reduction typically linked to aging.

**Dwelling, Rescanning, and Skipping of Distractors Explain Search Efficiency in Difficult Search: Evidence from Large Set Sizes and Unstructured Displays** **Gernot Horstmann, Stefanie Becker, Daniel Ernst**

Bielefeld University, Germany

University of Queensland, Australia gernot.horstmann@uni-bielefeld.de

Popular models of overt and covert visual search focus on explaining search efficiency by visual guidance. Comparably little attention is given to other variables that might also influence search efficiency, such as dwelling on distractors, skipping distractors, and revisiting distractors. Here we test the relative contributions of dwelling, skipping, rescanning, and the use of visual guidance, in explaining visual search times, and in particular the similarity effect. The hallmark of the similarity effect is more efficient search for a target that is dissimilar from the distractors compared to a target that is similar to the distractors. In the present experiment, participants have to find an emotional face target among nine neutral face non-targets. In different blocks, the target is either more or less similar to the nontargets. Eye-tracking is used to separately measure selection latency, dwelling on distractors, and skipping and revisiting of distractors. Overall, the results show that with complex stimuli like faces, target-distractor similarity influences search times primarily via the time the gaze dwells on the nontargets and to a somewhat lesser degree by altering the proportion of revisited non-targets in the course of search. Measures of attentional guidance contributed relatively little to the similarity effect. 

**The effect of changing the item relevance in repeated search**

**Sebastian A. Bauch, Christof Körner, Iain D. Gilchrist, Margit Höfler** University of Graz, Austria

School of Experimental Psychology, University of Bristol, UK sebastian.bauch@uni-graz.at

When we search the same environment repeatedly, the relevance of a search item might change from one search to the next. Here we investigated whether such a change in relevance is reflected in oculomotor behaviour. Par-ticipants searched the same display, consisting of pink and blue letters, twice consecutively. Participants knew in advance that, in the first search, the target could be of any colour, whereas in the second search, the target colour was always fixed. In Experiment 1, we presented a probe during the first search at an item whose colour would become relevant or not for the second search. Participants were required to saccade to the probe and then to proceed with the search. The results showed no difference in saccadic latencies to the probe with regard to the future relevance of the items. In Experiment 2, we presented the probe at the beginning of the second search to investigate whether the change of item relevance influences search immediately. Here, saccadic latencies were longer to irrelevant as compared to relevant items. This suggests that participants could exploit knowledge of item relevance and adapt their search immediately once search items change relevance. 

**Target and distractor guidance in repeated visual search: When using memory does not improve search**

**Margit Höfler, Iain D. Gilchrist, Anja Ischebeck, Christof Körner**

University of , Austria University of Bristol, UK ma.hoefler@uni-graz.at

When the same display is searched consecutively twice for different targets, the second target is found faster if it was recently fixated in the previous search. This search benefit can be explained by a limited short-term memory buffer that operates according to a first-in first-out (FIFO) principle: Each newly inspected item enters the buffer while the least recent one exits it. Search can thus be guided to items in the buffer when they become a target. Such a model predicts no further search benefit if a search is repeated three times. In the reported experiment, participants performed three consecutive searches in which a target could be absent or present. We found the expected search benefit for targets and this benefit did not accumulate across searches, as predicted. However, we also found a similar pattern for target-absent trials. Recently fixated distractors that remained distractors in the subsequent search were less likely to be re-inspected, thus producing a search benefit in target-absent trials. This finding suggests that the information about items stored in the FIFO buffer can be used flexibly to do both, return to these items if they become a target, or avoid them if the target is not among them.

**Process Analysis of Visual Search in ADHD, Autism and Healthy Controls – Evidence from Intra- Subject Variability in Gaze Control.**

**Divya P. , Holger Hill, Giuseppe Boccignone, Tom Foulsham, Christian Fleischhaker, Monica Biscaldi, Ulrich Ebner-Priemer, Christoph Klein**

Department of Child and Adolescent Psychiatry, Medical Faculty, University of Freiburg, Germany; 

Institute of Sports and Sports Sciences, Karlsruhe Institute of Technology, Germany; Department of 

Computer Science, University of Milan, Italy; Department of Psychology, University of Essex, United 

Kingdom; School of Psychology, Bangor University, United Kingdom; Department of Child and Adolescent Psychiatry, Medical Faculty, University of Cologne, Germany divya.seernani@uniklinik-freiburg.de

Increased Intra subject variability, i.e. moment to moment fluctuations in performance, is a candidate endophenotype of Attention Defecit Hyperactivity Disorder (ADHD. In light of potential etiological overlap between ADHD and Autism Spectrum Disorder (ASD) (Biscaldi et al., 2015; Rommelse et al., 2011), it is important to study ISV, in both aforementioned disorders simultaneously. Here, we broaden the study of ISV from reaction time tasks with manual responses to the ISV of gaze control. Children and adolescents with ADHD, ASD and healthy controls, aged 10-13 years (N = 90; all native German speakers) were invited for an oculomotor testing session. Participants were presented a visual search task. The task required participants to find a Portuguese target word shown above a grid with multiple Portuguese German word pairs and indicate its position by pressing response keys matching the search array. Preliminary analysis have been calculated with moment-to-moment fluctuations in eye movements for the period of search. Preliminary results suggest increased ISV in the ADHD group. Our study extends the ISV finding to the ocular-motor domain, proposes methods to study ISV in gaze movement, and highlights its relationship with ASD. 

**When one target predicts the other: Target guidance in visual search**

**Christof , Jonas Potthoff**

University of Graz, Austria christof.koerner@uni-graz.at

Knowledge about a target feature such as its luminance can guide search efficiently. Here we investigated how a search for a target is guided when its luminance is indicated by the luminance of another target. Nineteen participants searched for two target letters (T’s) among distractors (L’s) in displays of 10 high- and 10 low-luminance items. Critically, the luminance of the first found target during search indicated the luminance of the second target (same vs. different). Hence, the luminance information could be retained or had to be updated to guide search for the second target. In a third (random) condition there was no consistent luminance relationship between the targets. We counted the number of fixations necessary to find the second target as a measure of search guidance. Compared to the random condition, participants needed substantially fewer fixations to locate the second target when the first target indicated its luminance. Interestingly, participants made an additional fixation on distractors sharing the luminance of the first target when, in fact, luminance information had to be updated. Our results suggest that the search for a target could be effectively guided by luminance information of another target but slightly less so if an update was required.

**Does context influence the low prevalence effect in visual search?** **Titus N. Ebersbach, Walter R. Boot, Ralph Radach**

Bergische Universität Wuppertal, Germany

Florida State University, Tallahassee, USA titus.ebersbach@uni-wuppertal.de

When a is rarely present in a visual search task its detection probability is drastically lowered which was coined the low prevalence effect. Wolfe et al. (2007) found that bursts of high prevalence trials intermixed with low prevalence trials can mitigate the low prevalence effect. Our present work examines whether this finding holds when high vs. low prevalence trials are distinguishable through a contextual cue within the same block. 

Participants were asked to search for a target X among distractor letters within a colored frame. The red frame indicated a high chance of a target being present (50 percent), while the white frame signaled a low chance (5 percent). We found a low prevalence effect both in a mixed high and low prevalence and a blocked control version. On white trials participants were biased towards reporting absent. Surprisingly, mixing the trials caused response times on white trials to be faster, but not at the cost of lowered accuracy, suggesting that participants did take the context into account. This benefit was partly driven by shorter fixation durations on the white trials in the mixed compared to control condition. 

**Simulation of visual hemi-neglect by spatio-topic and retino-topic manipulation of visual search displays**

**Jennifer Winter, Björn Machner, Inga Könemund, Janina von der Gablentz, Christoph Helmchen, Andreas Sprenger**

Institute of Psychology, University Luebeck, Germany Department of Neurology, University Luebeck, 

Germany

jennifer.winter@student.uni-luebeck.de

Right stroke patients frequently experience spatial neglect, a severe lack of awareness for contralesional hemispace. In hemi-spatial neglect eye movement patterns during visual search reflect not only inattention for the contralesional hemi-field, but interaction of multiple visuo-spatial functions’ deficits. In this study we simulated visual hemi-neglect by spatio-topic and retino-topic (gazecontingent) online manipulation of a visual search scene in healthy participants. Manipulation methods used a gaze-contingent gradually reduction of luminance, sharpness (i.e. blur) or color on a nonmanipulated (original) stimulus or a spatial gradual reduction of color. Data revealed main effects for target position, gaze-contingent modification, spatio-topic manipulation and for the manipulation function slope. It turned out that static more than dynamic modification increased search duration similar to neglect. A steeper slope of the modification function augmented search duration. Nevertheless, search duration was much shorter than in a cohort of neglect patients after right hemisphere stroke that we had investigated before using the same stimuli. Although we could show that spatio-topic and retino-topic manipulation affects visual search, the attentional bias in visual hemineglect is more than the pure visual attraction to the ipsi-lesional side but a massive disturbance of the entire attentional system and the visual network.

**Where can I find the Honey, Honey? Using color cues to overwrite syntactic rules in a scene-search paradigm**

**Marian Dieguez Laukamp, Lisa Völker, Sabine Öhlschläger, Melissa Le-Hoa Vo**

Scene Grammar Lab, Goethe University, Frankfurt, Germany Center for Research on Individual 

Development and Adaptive Education of Children at Risk (IDeA), Frankfurt, Germany MD.Laukamp@web.de

Our everyday-life is determined by a multitude of explicit and implicit rules. These rules, however, might be restricted to specific contexts so that we have to adapt to environments generated under different cultural conditions. In an eye-tracking study we tried to overwrite adults’ location rules in a cued scene search-task and manipulated observer’s prior knowledge about the cue contingency: The background color always predicted whether the object positioning in the search scene was consistent (pot on the stove) or inconsistent (pot anywhere but on the stove). We expected that being explicitly informed about the inconsistent placement of an object would, for instance, steer eye movements away from the consistent location. However, if at all, informed participants were only able to place their 5th fixation further away from the consistent position, indicating a very restricted influence of explicit rule knowledge on strategic eye movement control. Dwell and decision times for targets were shorter under explicit rule knowledge indicating a reduced overall uncertainty in object-scene processing. The general experience of objects not always being in place reduced the fixation probability of consistent target AOIs independent of the information provided. Thus, we seem to be better at learning by doing than being told.

**Time course of central and peripheral processing during scene viewing**

**Anke Cajar, Ralf Engbert, Jochen Laubrock**

University of Potsdam, Germany cajar@uni-potsdam.de

A key issue for understanding eye-movement control during scene viewing is to understand the roles played by central and peripheral vision. Yet, little is known about how much time is allocated to the processing of central and peripheral scene information during fixations. In two experiments, we investigated this question using the scene and mask onset delay paradigms. During critical fixations, scenes were degraded either in the central or the peripheral visual field for variable time intervals by attenuating low or high spatial frequencies or introducing a uniform gray mask. Results show that central or peripheral scene degradation at any time during fixation increased fixation durations, with weaker effects for low-pass filtering than for high-pass filtering or masking. In most conditions fixations lengthened increasingly with the duration of degradation. Thus, both central and peripheral information is processed during a large part of the fixation. When degrading the scene from the beginning of the fixation, fixation durations were consistently longer with central than with peripheral filtering, indicating that central information might be more important at the beginning of the fixation. Our results suggest that central and peripheral processing proceed largely in parallel during fixation, with a somewhat stronger weight on central processing.

**Central fixation bias: The role of sudden image onset and early gist extraction** **Lisa F. Schwetlick, Lars O. M. Rothkegel, Hans A. Trukenbrod, Ralf Engbert**

University of Potsdam, Germany lisa.schwetlick@uni-potsdam.de

Scene perception is used to study target selection of the eyes on complex but well controlled stimuli. However, target selection is dominated by a tendency of human observers to place fixations near the center of images, a phenomenon called Central Fixation Bias (CFB). Recent studies have shown that the CFB can be significantly reduced by experimentally delaying scene exploration by at least 125 ms after image onset. Here we show that this reduction is primarily caused by early information extraction from an image. In the current study we dissociated two possible factors contributing to the CFB: knowledge of the image content and sudden image onset. Participants were shown either valid, invalid, or phasescrambled previews of an image before exploration. Additionally, the images were either presented with a sudden onset or were faded in for 250 ms to prevent sudden luminance changes. Results show that the early CFB was reduced for valid preview conditions. We observed no differences between sudden onset and fade-in of images. Thus, the CFB is primarily shaped by early gist extraction with the image center as the optimal fixation location.

**Eye movements in scene perception during quiet standing**

**Daniel Backhaus, Hans A. Trukenbrod, Lars O. M. Rothkegel, Ralf Engbert**

University of Potsdam, Germany backhaus@uni-potsdam.de

Scene perception is commonly used to study overt allocation of attention. However, generalizability of theoretical implications has been questioned due to limitations of the paradigm (see Tatler et al., 2011). For example, in a typical scene perception experiment a participant is seated in front of a display with the head stabilized by a chin rest. Such static viewing is in contrast to everyday activities where participants are freely moving. Here we relax the static viewing paradigm by recording from participants standing in front of a projector screen. We run a scene perception experiment where participants were asked to memorize images for a subsequent recognition test. Each image was presented twice. Eye movements were recorded with a mobile eye-tracking device. Visual angle of the presented images was the same in both experiments. Among others, our results showed that distributions of fixation durations and saccade amplitudes were very similar across experiments while the central fixation bias (see Tatler, 2007) was reduced during standing. Our results show that restrictions of head movements influence eye movements. Systematic investigation of limitations of the sceneperception paradigm will inform eye movement control in less constrained everyday activities.****

**Gaze on a Stochastic Image.** **Miriam Mirolla, Emiliano Melchiorre**

Accademia delle Belle Arti di Roma, Italy miriam.mirolla@gmail.com

The measurement of gaze movements applied to artworks has revealed a wide range of differences emerging by comparing abstract versus representational paintings. However, what happens when we observe a stochastic image, an unpredictable universe which has no center nor edge? An experiment with a stochastic painting conceived by Italian artist and psychologist Sergio Lombardo, was conducted. The aim of this experiment was to compare 20 eye gazing paths and analyze the evocative spectrum in the beholders' descriptions.

**Eye movements and saliency for the Hollywood2 action recognition benchmark**

**Michael Dorr, Eleonora Vig**

Technical University Munich, Germany

German Aerospace Center, Oberpfaffenhofen, Germany michael.dorr@tum.de

Action recognition in videos remains a challenging computer vision task. In previous work (Vig et al., ECCV 2012), we had shown that classification performance on the Hollywood2 benchmark could be improved by a preprocessing step that mimics attention, by pruning image descriptors based on ground truth (human gaze) or predicted eye movements (saliency). We here used two large-scale gaze data sets for Hollywood2 (Mathe et al., Vig et al.) and applied our approach to the new, state-of-the-art 'Improved Dense Trajectories' pipeline, which compensates for camera and background motion and thus may achieve a similar effect as saliency-based pruning of trajectories. We first investigated whether both independently collected data sets were comparable. A Normalized Scanpath Saliency analysis showed that the two 'free-viewing' conditions were similar despite different experimental setups; however, this analysis also showed an effect of task (active action recognition). Surprisingly, classification performance did not improve for both empirical and analytical saliency measures, nor for a new measure based on smooth pursuit eye movements. Small classification performance gains (<1%) at strongly improved efficiency (>2x) were achieved with peripheral pruning, exposing the strong centre bias artefact in professionally produced and cut 'naturalistic' videos.

Cultural variation in eye movements during scene perception: replication with a Russian sample 

**Anton Gasimov, Artem Kovalev**

Lomonosov Moscow State University, Russian Federation gasimov.anton@gmail.com****

Previous cross-cultural eye-tracking studies have found that culture shapes eye movements during scene perception. These researches have been limited to the American, Chinese and African samples. However there are no evidences how Russians view photographs with focal object on a complex background. This study recruited 11 participants from the Western and 11 participants from the Eastern regions of Russia. Experimental material and the procedure were the same as Chua et al. (2005) to maintain the reliability and validity. Each experiment consisted of study phase and recognition phase. All images had only one focal object on a complex background. The study phase had 36 pictures and the recognition phase had 72 pictures. The number of fixations, first fixations times on focal objects and fixation durations were significantly differed (F=20,161, df=1, p<0,001) between groups. Subjects from Western regions fixated more on the focal objects, tended to look on them more quickly and recognized more objects. Fixation durations of subjects from Eastern regions during background viewing were smaller. Thus, subjects from Eastern and Western parts of Russia demonstrated different strategies of scene perception due to the cultural differences in these regions. This study was supported by grant RSСF №15-18-00109. 

**The of verbalization on eye movement parameters during complex scene repeated viewing**

**Veronika Prokopenya, Ekaterina Torubarova**

Saint Petersburg State University, Russian Federation

v.prokopenya@spbu.ru

We investigated how the verbal description of the previously seen complex scene influenced the eye movement parameters during its re-observation. Recent studies showed that picture processing can be divided in two stages: the ambient viewing, characterized by short fixations and long saccades, followed by focal viewing, characterized by longer fixations and shorter saccades (Fisher et al. 2013). In our eyetracking experiment 60 subjects were looking at the classic painting, and then one group was asked to compose coherent verbal description of the painting, and another group had a nonverbal distracting task. After that both groups re-observed the picture. All subjects demonstrated both stages of processing (ambient and focal) during the first viewing, while the gaze-pattern of the second viewing differed between the groups. Subjects who hadn`t verbalized the painting continued its focal processing, as if they did not interrupting in the examination of the picture. Subjects who had verbalized the painting began the second viewing with the ambient processing, and only few seconds later shifted to the focal processing, i.e. their fixation duration increased and saccadic amplitude decreased. These results demonstrated that verbalization of the picture affected oculomotor behavior during its repeated viewing.

**Clinical Research**

**EyeGrip as a for assessing dementia **

**Diako Mardanbegi, Shahram Jalaliniya, Hans Gellersen, Trevor J. Crawford, Peter Sawyer** Lancaster University, United Kingdom 

d.mardanbegi@lancaster.ac.uk

Emerging evidence reveals that eye movement deficits develop with dementia (Crawford, et al., 2005). One of the symptoms is exaggerated attentional blink during rapid serial visual presentation. A recent study has shown that people with AD have a unique form of attentional masking where they miss the first target but identify the second, depending on the number of intervening distractors (Kavcic, V., & Duffy, C. J., 2003). Other cognitive impairments that lead to different eye movements are top-down attentional process impairments and memory loss. We propose the idea of using EyeGrip technique which is an automatic method for detecting object of interest among other scrolling visual content (Jalaliniya, S., & Mardanbegi, D., 2016) as a diagnosis tool for studying people with dementia. In our study, we recruited 5 dementia patients and 5 controls. We presented scrolling images of faces including some familiar among other random faces on a screen. It is expected that the pattern of OKN eye movements (such as slow phase) to be disturbed and missed targets in dementia patients to be significantly increased compared to control subjects since dementia affects the top-down process of attention and memory. 

**Executive function processes in dementia:**

**Impairments in anti-saccadic eye movements are indicative for first disease stages**

**Lucas **^1^**, Martin Pszeida**^1^**, Mariella Panagl**^2^****

^1^JOANNEUM RESEARCH Forschungsgesellschaft mbH, Austria; 

^2^Sozialverein Deutschlandsberg, Austria lucas.paletta@joanneum.at

A key problem in developing knowledge about dementia and impacting factors is lack of data about mental processes evolving over time. Cognitive and behavioral interventions, emotional support by caregivers and physical exercise programs are beneficial to activities of daily living (Forbes et al., 2013). However, lack of exercise is a major risk factor in dementia development (Norton et al., 2014). In a serious game performed by clients, mobile eye tracking was applied for non-obtrusive sensing and daily monitoring of dementia profiles. An anti-saccade measuring paradigm was used for eye movements captured during playing the Tablet PC serious game. It is known to detect impulse control problems as they occur in executive function related neurodegenerative diseases (Crawford et al., 2005). In 4 training sessions with 15 participants eye movement data were collected from users with dementia (MMSTE = 25 avg.) and non-dementia users (MMSTE = 30 avg.). The system measured an error rate of MEAN 43.2% (STD 20.0%) for people with dementia in contrast to people without the disease (MEAN 7.7%, STD 5.0%), MMSTE correlated with error rate by r=0.632 (p=0.09). The serious game attentional diagnostic toolbox offers affordances for entertaining and analysis of behavioral parameters for longitudinal studies. 

**EYE MOVEMENT BEHAVIOR IN MCI AND AD:**

**USING AUTOMATIC CLASSIFICATION ALGORITHMS TO IDENTIFY COGNTIVE DECLINE**

**Marta L. G. F. Pereira**^1^**, Marina von Zuben de Arruda Camargo**^1^**, Ariella F. Belan**^1^**, Bernardo dos Santos**^2^**, Orestes V. Forlenza**^1^

^1^LIM27 - Neurosciences Laboratory, Institute of Psychiatry, Faculty of Medicine, University of São Paulo - Brazil; 

^2^Nursing - University of São Paulo – Brazil martafreitaspereira@gmail.com

BACKGROUND: Eye movement analysis is often based on single parameters, resulting in some degree of variability in Alzheimer Disease (AD) subjects’ oculomotor performance. Therefore, we aimed to: (1) determine which measures best distinguish between healthy controls (NC) and AD subjects; (2) identify Mild Cognitive Impairment (MCI) subjects with an oculomotor profile compatible with AD. METHODS: Machine Learning Methods were applied to classify 3 groups of subjects (AD=33; MCI=52; NC= 43). We investigated the capacity of the defined parameters to distinguish between AD and NC groups. Classification models were trained on a subsample of the 3 groups. MCI data was tested with the classifiers to verify if MCI subjects would have an pattern similar to either AD or NC group. RESULTS: We were able to distinguish AD from NC, with good levels of performance, reaching 85% of accuracy, 70% of sensitivity and 18% of error. Also, the classifiers successfully classified 18 MCI subjects with an AD oculomotor profile. CONCLUSION: Different parameters, when combined together, significantly improve the ability to distinguish between healthy and impaired subjects. Also, the model reveals the potential to early detect oculomotor deficits in MCI patients similar to AD subjects, and suggests an approach for detecting early AD. 

**Eye Movement Parameters while Executing Oculomotor Tasks in Patients with Cerebellum Tumor **

**Marina Shurupova**^1,2^**, Viktor Anisimov**^1,2^**, Alexander Latanov**^2^**, Vladimir Kasatkin**^1^****

^1^Dmitry Rogachev National Research Center of Pediatric Hematology, Oncology and Immunology, Russian Federation; ^2^Moscow State University, Russia shurupova.marina.msu@gmail.com 

Medulloblastoma is a cancerous glial cerebellum tumor localized in the area of vermis and hemispheres. The cerebellum impairments were shown to cause oculomotor and cognitive disorders related to its functions. We conducted a pilot study of three oculomotor tasks execution in patients with medulloblastoma. Five patients undergoing rehabilitation (3 girls and 2 boys, 13.4-17.9 year old) participated in the study. Eye movements were recorded with Arrington Research Eye Tracker (60 Hz). The patients executed three oculomotor tasks in two sessions during two rehabilitation courses: 1) gaze fixation test, 2) visual attention switching test (Go/NoGo paradigm), 3) visual scanning strategy test (to look at 10 targets one by one). The first test revealed significant decrease of gaze samples dispersion in four patients, presumably related to decreasing of intrusive saccades. In the second test the rate of relevant responses increased because of decrease irrelevant saccades amount that gives evidence of improved their inhibition. In the third test all patients showed significant decrease total execution time, fixations number, and scanpath length. Therefore, we revealed positive dynamics of oculomotor parameters in patients with medulloblastoma during their rehabilitation courses. Thus, eye tracking method provides an objective estimation of oculomotor and associated cognitive functions improvement.

**GENERAIN – a transgenerational eye-tracking study on attention biases in children at risk for depression**

**Anca **^1^**, Elske Salemink**^2^**, Gerd Schulte-Körne**^1^**, Belinda Platt**^1^****

^1^Department of Child and Adolescent Psychiatry, Psychosomatics and Psychotherapy, Ludwig-

Maximilians-University Munich, Germany; 

^2^Department of Psychology, University of Amsterdam, Netherlands 

Anca.Sfaerlea@med.uni-muenchen.de

Children of depressed parents have an increased risk of developing depression themselves. Implicit cognitive biases (reliably observed in depressed patients) may be one means by which depression is transmitted from parent to child, but the presence of these biases has rarely been examined in children of depressed parents. Eye-tracking paradigms are particularly useful for the investigation of implicit attention biases as they allow assessment of attention allocation patterns in more ecologically valid ways than experimental tasks relying solely on reaction times. Harrison and Gibb (2015) recorded eye-movements during a passive viewing task (in which neutral, happy, sad, and angry faces were presented simultaneously) and found depressed children (compared to non-depressed children) to spend less time fixating sad faces i.e. to show attentional avoidance of sad faces. The present study applies the same paradigm to investigate if this bias can also be found in 9-14 year old children of depressed parents. Additionally, a trans-generational aspect will examine whether parental biases predict children’s biases. We recruited parent-child-dyads from families in which at least one parent suffered from depression (n = 40) and families with no history of depression (n = 40). Data analysis is ongoing but will be completed before August 2017. 

**Saccadic inhibition and its interaction with implicit processing of emotion in Bipolar Disorder patients**

**Nathalie **^1^**, Alan Chauvin**^2^**, Lysianne Beynel**^3^**, Sylvain Harquel**^2^**, Cécilia Neige**^4^**, Mircea Polosan**^5^****

^1^GIPSA-lab, UMR 5216 CNRS, Univ. Grenoble Alpes, Grenoble INP, France; ^2^LPNC. UMR 5105 CNRS,

Univ. Grenoble Alpes, Grenoble, France; ^3^Duke University, Durham, North Carolina; ^4^Laval University, Québec; ^5^Psychiatry and Neurology,Univ., Grenoble, France nathalie.guyader@gipsa-lab.grenoble-inp.fr

Introduction: Finding specific abnormalities in bipolar disorder (BD), persisting in euthymic phase is crucial for clinicians. This study aims to assess saccadic inhibition and its link with an implicit emotional processing as a biomarker of this pathology. Method: 62 BD patients and 57 healthy subjects performed a saccadic task. Each trial starts with color displayed on a central dot (SPAN) or surrounding a face image, happy or sad, (SPANemo). Color indicates the type of saccade: antisaccade (AS), prosaccade (PS) or no saccade (NS). Then, a cue appears on the right or left and participants have to gaze at the cue (PS), its mirror position (AS) or stabilize their gaze (NS). Participants have to only focus on the color and not on the face. Saccadic reaction time (SRT) and inhibition error saccade rate are analyzed. Results: In the SPAN experiment, BD patients were faster but less accurate during AS than controls and produced more error during NS. In the SPANemo experiment the implicit processing of emotion interferes with the ability of patients to manage saccadic control. Conclusion: This study shows that a classic saccadic task or its coupling with implicit emotion processing is a reliable tool to identify euthymic bipolar disorder. ****

**Utilizing Eye-Movement Patterns for Improving ADHD Diagnosis and Malingering Detection**

**Michael Wagner, Corinne Berger, Yoram Braw, Tomer Elbaum, Tzur Chohen**

Ariel University, Ariel, 44837, Israel wag.michael@gmail.com

Continuous-Performance-Tests (CPT) are commonly implemented for "Attention-Deficit-HyperactivityDisorder" (ADHD) assessment. The MOXO-d-CPT is an online-administered CPT, reporting four indices: Attention, timing, hyperactivity and impulsivity. Participants’ task is timed-key-press-responses to target stimuli, refraining key-pressing-response to non-target-stimuli. Stimuli and visual distractors are displayed within 5 screen areas. Target stimuli are displayed with/ without visual/ auditory peripheral distractors. We measure participant’s eye-movements synchronized with performing MOXO-d-CPT, (Neurotech Ltd. MOXO-d-CPT developer collaboration), aimed to enhance adult ADHD diagnosis accuracy, and detecting ADHD malingering. Here we present preliminary data indicating distinctive eyemovement patterns, enabling significant differentiation of three types of adult participants (students): 1- diagnosed ADHD participants, 2- Healthy participants simulating ADHD (“simulants”), 3- Healthy participants. (n=12). Our eye-movements data show higher proportions of fixations and dwell time within the distractors interest areas for the diagnosed ADHD and “simulants”, than in healthy controls. While MOXO results could not differentiate Diagnosed ADHD from “simulants”, we found “distractororiented-saccades” during “distractor-trials” in Diagnosed ADHD, while “simulants” fixated peripheral areas during all trial types, including out of task areas. We could also differentiate these groups by means of saccadic amplitudes. Our results substantiate feasibility of improved ADHD diagnosis accuracy, and detection of ADHD malingering, by CPT - synchronized eye-movement measures. 

**Parafoveal processing Efficiency in Chinese developmental dyslexia: Evidence from RAN tasks**

**Wen Wang, Ke Tan, Mingzhe Zhang, Xuejun Bai**

Tianjin Normal University, China bxuejun@126.com 

Two current different forms of parafoveal dysfunction have been assumed as core deficits of dyslexic readers: reduced parafoveal preview benefits and increased parafoveal load costs. In experiment 1, we tested both hypotheses simultaneously by using a modified serial rapid automatized naming paradigm (as proposed by Silva et al., 2016) in an eye movement experiment. Three groups of children were selected as participants: developmental dyslexia (DD), chronological age control (CA), and reading level control (RL). The results showed that DD had reduced parafoveal preview benefits, but did not show more parafoveal load costs compared to the matched groups of children. In experiment 2, we further investigated which types of information DD could acquire from parafoveal preview using a combined RAN and boundary paradigm. Four preview conditions were manipulated: identical preview, orthographically similar preview, phonologically similar preview, and unrelated control preview. We found all three groups of children did not show any phonological preview benefits; DD were able to acquire orthographical preview benefits, but this effect was relatively delayed as compared to the control groups. Our findings strengthen the idea that a reduced parafoveal preview benefit is a core deficit in Chinese dyslexia that is consequent to reading problems. ****

**Investigating the effects of orthographic visual complexity on fixations in typical and dyslexic reading of English**

**Rea Marmarinou**^1^**, Jun Bao**^1^**, Richard Shillcock**^1^**, Mateo Obregón**^1^**, Hamutal Kreiner**^2^**, Matthew A.J. **

**Roberts**^1^**, Scott McDonald**^3^

^1^University of , United Kingdom; 

^2^Ruppin Academic Centre, Emeque-Hefer, Israel; 

^3^National Institute for Public Health and the Environment, Bilthoven, Netherlands s1142323@sms.ed.ac.uk

We investigated the effects of orthographic ‘perimetric complexity’ (perimeter squared over ‘ink’ area) (Pelli, Burns, Farell, Moore-Page, 2006) in the reading of English text by typical and dyslexic readers in the Edinburgh 5-Language Corpus. We tested the hypothesis that greater visual complexity would affect the size of the fixation disparity (FD) (the distance between the fixation points of the left and right eyes) and the duration of the fixation. FD represents a principled window onto the reading process, obviating the need to make assumptions about parafoveal processing or the size of the perceptual window. In addition, there have been claims of processing advantage within the FD (Obregón & Shillcock, 2012; Shillcock, Roberts, Kreiner, & Obregón, 2010) We found that orthographic perimetric complexity within the FD was not significantly correlated with size of the FD or with fixation duration in either the typical readers or the dyslexic readers. We provisionally conclude that processing in this region of the perceptual window may already be at ceiling, reflecting a processing advantage, or that the FD and fixation duration do not reflect this level of processing complexity.

**The benefit of eye tracking in the assessment and therapy of acquired dyslexia **

**Irene Ablinger**^1^**, Ralph Radach**^2^****

^1^SRH University of Applied Health Sciences Gera, Germany; 

^2^General Biological Psychology, University of Wuppertal, Germany

radach@uni-wuppertal.de 

As a consequence of brain damage, patients with acquired dyslexia often show massive impairments in reading. Traditionally, the severity of the reading disorder, the identification of the preferred reading strategy and the efficiency of reading interventions are all assessed by using psycholinguistic error analyses in reading aloud. Over the last decade we have been pursuing a research program utilizing eye movement methodology in this area. In the present study we present results from five aphasic patients with acquired dyslexia who participated in an eye movement contingent reading intervention, focussing on the remediation of lexical and segmental reading processes. Before and after intervention eye movement data were used to identify viewing patterns over the time course of word reading. Overall, therapy led to improved reading accuracy and changes in fixation patterns. Critically, data from all participants indicate discrepancies between word reading strategies suggested by linguistic error analysis and those identified by visuomotor analyses of fixation positions. These data strongly suggest a separate consideration of real-time word recognition and subsequent verbal output.Diverging receptive and expressive word processing mechanisms in a deep dyslexic reader. Neuropsychologia, 81, 12-21.

**A visuomotor analysis of multilevel therapy in pure alexia**

**Anne Friede**^1^**, Irene Ablinger**^2^**, Ralph Radach**^1^****

^1^University of , Germany; 

^2^SRH University of Applied Health Sciences, Gera, Germany

afriede@uni-wuppertal.de 

We report on HC, a patient with pure alexia following a posttraumatic stroke. His oral reading was characterized by pronounced sublexical word processing with letter-by-letter scanning and an inflated word length effect. Our individualized intervention represents the first attempt to systematically combine sequential and whole-word reading techniques in the treatment of pure alexia. The intervention was hierarchically structured in syllable and word reading tasks, supplemented by a criterion based text reading training. In addition to traditional linguistic error analyses we used eye movement methodology to characterize HC’s recovery progress (see Ablinger, Huber & Radach, 2014). Results indicated a substantial decrease of both first pass word viewing and re-reading times, for trained and untrained materials. Training effects on word reading were well maintained over a period of 5 months. Importantly, mean initial fixation positions moved from the left edge about two letter positions further into target words, indicating a substantial change in word reading strategies. Still, HC’s reading was dominated by very time-consuming serial processing routines. In conclusion, eye movements indicate that even with optimal therapy pure alexia patients do not return to normal reading. However, an individualized, multilevel intervention can help developing efficient alternative strategies of visual and linguistic processing. 

**Eye movements in text reading in a patient with incomplete Bálint`s syndrome**

**Katja Halm**^1^**, Ralph Radach**^2^**, Irene Ablinger**^3^****

^1^RWTH Aachen University Hospital, Germany; 

^2^General Biological Psychology, University of Wuppertal, Germany; ^3^SRH University of Applied Health Sciences, Gera, Germany

khalm@ukaachen.de 

The Bálint`s syndrome is associated with a combination of optic ataxia, simultanagnosia and gaze apraxia resulting from bilateral parieto-occipital lesions. Fixation and ocular exploration of space are severely impaired, including reading (Kerkhoff, 2000). In the present study we report on RM, a 21-year-old man with incomplete Balint`s syndrome following a hypoxic brain injury 8 months ago. RM showed impaired spatial orientation and severe problems in visual perception of colors and pictures. Remarkably, he was able to read and understand text. We were interested in RM`s visual processing strategy in text reading which seemed to be more successful than visual and spatial exploration in other contexts. Therefore we used eye tracking methodology to characterize the underlying reading procedures. We found substantially inflated total reading times, due to both icreased numbers of refixations and more episodes of re-reading. The patient uses as a primarily segmental reading strategy, but there is also evidence for holistic word processing. Although visual perception and spatial orientation were severely impaired, RM was able to compensate his difficulties during reading using a unique combination of processing strategies. 

**Localizing hemianopic visual field defects based on natural viewing behavior while watching movie clips**

**Birte Gestefeld**^1^**, Alessandro Grillini**^1^**, Jan-Bernard C. Marsman**^2^**, Frans W. Cornelissen**^1^****

^1^University Medical Center Groningen, Netherlands; 

^2^NeuroImaging , University Medical Center Groningen, University of Groningen, Netherlands

b.f.gestefeld@umcg.nl

Performing standard perimetry is tedious and fatiguing. Therefore, we asked whether we can identify the location of a visual field defect in an easier way using eye-movements. We tested this idea using simulated Homonymous Hemianopia (HH). In our approach, we exploited the fact that healthy observers show quite consistent gaze behavior when watching movie clips (Marsman et al, 2016). We hypothesized that in case of HH, observers will rarely direct their gaze to locations in the blind hemifield, even when these are prioritized by healthy observers. In the experiment, participants watched movie clips under different visual (simulated right or left HH and without HH), while their eye-movements were recorded. A measure for viewing priority at different locations of the visual field, in the control condition, was computed. Next, averaged over the various movie clips, we determined viewing priority for each observer in each simulation condition. We found that in the simulated HH conditions, average viewing priority in the blind half of the visual field is significantly lower than in the seeing half of the visual field. We conclude that that we can derive the location of a hemianopic visual field defect from natural viewing behavior exhibited during movie viewing.

**Visual search behaviours in dementia-related visual impairment in controlled real-world settings**

**Ayako Suzuki, Keir Yong, Ian McCarthy, Tatsuto Suzuki, Dilek Ocal, Nikolaos Papadosifos, Derrick Boampong, Nick Tyler, Sebastian Crutch**

University College London, United Kingdom

a.suzuki@ucl.ac.uk

Posterior cortical atrophy (PCA) is a neurodegenerative syndrome, most commonly caused by Alzheimer’s Disease, characterised by progressive visual dysfunction, occipito-temporal and occipitoparietal atrophy. PCA patients often experience difficulties in everyday tasks involving spatial orientation and object finding, but the relationship between such difficulties and eye movement abnormalities is little understood. This study assessed differences in eye fixation position relating to visual search characteristics as individuals with PCA, typical Alzheimer’s Disease (tAD), and controls navigated a controlled real-world setting under different conditions of lighting and visual clutter. Ten PCA patients, 9 tAD patients and 12 controls were asked to locate and reach a target destination within a controlled environment set up in the UCL Pedestrian Accessibility Movement and Environment Laboratory. Participants’ eye movements were monitored with SMI mobile eye tracking glasses. Head and shoulder positions were recorded by a 3D motion capture system. Preliminary analysis suggests inefficient visual search within the PCA group, with the time until first fixation on the target destination being longer in the PCA than control and tAD. Future investigations may reveal whether certain environmental features more strongly predict fixation position in PCA and tAD relative to control groups. 

**Playing games with your eyes: using gaze for intervention and outcome assessment in ASD**

**Leanne Chukoskie, Jeanne Townsend**

UC San Diego, United States of America lchukoskie@ucsd.edu

Fast and accurate attention shifting and eye movement control are essential for gathering sensory information in dynamic environments however, both are atypical in individuals with autism spectrum disorder (ASD). Improvements in the accuracy and timing of overt and covert attention could have corollary beneficial effects on social engagement. We designed and deployed PC-based gaze-contingent video games using the Unity game engine and a low-cost eye tracker (EyeTribe). The games were designed around training principles to engage fast and accurate orienting behavior as well as stable fixation. We demonstrated the feasibility of using gaze-contingent video games for in-home training for high functioning adolescents with ASD. We also demonstrated improvement of covert attention orienting and overt gaze behavior (SR Research) after 8 weeks of play on these games in a small group of adolescents with ASD. Finally, we developed a protocol and analyses to use with a glasses-based eye tracking system (Pupil Labs) to examine whether the attention performance improvements we observed in our screen-based tasks transfer to a natural conversation setting. In conclusion, we delivered a robust, low-cost, gaze-contingent game system for home use that improved the attention orienting and eye movement performance of adolescent participants with ASD. 

**Novel steps for online eye-gaze contingent attention training: A mouse-based moving window approach**

**Alvaro Sanchez, Jill Van Put, Ernst Koster**

Ghent University, Belgium alvaro.sanchezlopez@ugent.be

Eye-gaze contingent attention training (ECAT) delivered via eye-tracking has proven to target attention mechanisms involved in emotion regulation. We tested a variant of ECAT delivered via gaze-mouse contingencies. Forty-one undergraduates were randomly assigned to either control or active gaze/mouse-based ECAT. Participants receiving active training were instructed to allocate attention toward positive words by using gaze/mouse coordination (i.e., participants moved the mouse cursor to unhide words and coordinates were used to provide contingent feedback on their viewing behavior). Participants in the control condition performed the task without receiving contingent feedback. Eyetracking was used in parallel to establish the level of gaze/mouse coordination. Transfer to reappraisal success and state rumination was evaluated with an emotion regulation paradigm. Mouse-based attention tracking showed high levels of congruency with gaze tracking as measured with the eyetracker, R2= 0.71. The training condition led to increased attention towards positive words, as indexed via both mouse- and eye-tracking, both F’s > 16.80, p’s< .001. Furthermore, the ECAT resulted in greater reappraisal success, F(1,39)=5.99, p=.019, and larger reductions in state rumination, F(1,39)=32.16, p=.001. The use of mouse-cursor as a way to monitor/train visual attention is an innovative feature that will allow fostering large-scale online ECAT.

**Reading: -level processing **

**Sentence to image priming of gender information. Can eyetracking data shed more light on priming effects?**

**Anton Öttl**^1^**, Ute Gabriel**^1^**, Dawn Marie Behne**^1^**, Pascal Gygax**^2^**, Jukka Hyönä**^3^****

^1^Norwegian University of Science and Technology (NTNU), Norway; 

^2^University of Fribourg, Switzerland; 

^3^University of Turku, Finland anton.oettl@ntnu.no 

Employing a sentence-to-image priming paradigm we investigate the cross modal activation of gender expectations in a series of experiments. Participants read short sentences whose subject is either gendered (e.g., ‘Jane and Mary are friendly’) or neutral (e.g., ‘The sofas are comfortable’). Each sentence was immediately followed by an image of two faces that had to be categorized as male, female or both. Preliminary results with native speakers of Norwegian and Finnish show that such gender priming occurs, as evidenced by shorter response times when categorizing a face pair whose gender is consistent with the preceding prime. The aim of the current presentation is to explore whether eyetracking data (collected during the original experiments) may provide additional insights into the effects of priming. More specifically, is the gender priming effect also detectable during online processing, and if so, is the online data more robust? The earliest stage at which we would expect the effect to surface would be in first fixation durations, reflecting an early facilitation in the recognition of face gender. Other eyetracking measures of interest include number of regressions between the two images and fixation latencies. 

**How L2 instruction influences eye-movements during reading: a withinparticipant study of English learners**

**Daniel Schmidtke**^1^**, Amy-Beth Warriner**^2^**, Victor Kuperman**^2^**, Anna Moro**^2^****

^1^University of Alberta, Canada; 

^2^McMaster University, Canada schmiddf@mcmaster.ca

Reading and oral proficiency influences eye-movements during reading both in L1 and L2 (Rayner, 1998; Whitford & Titone, 2017). However, within-participants studies of oculomotor reading behavior as a function of L2 instruction are rare. We studied a group of 31 Mandarin and Cantonese university-level learners of English who were tested twice, in the first and last weeks of a six-month intensive ESL program. We asked (a) what components of reading behavior would be most affected by instruction; (b) what individual skills at t1 would predict gains in reading proficiency at t2; and (c) what language skills developed between t1 and t2 would most strongly predict individual gains. Participants completed a battery of individual differences tests, and read passages for comprehension while eye-movements were monitored. The greatest change across time-points occurred for late eye-movement measures to words (total reading time and regression rate), i.e. reading comprehension progressed more than word identification. Also, the greatest gains in reading proficiency (gauged via eye-movements) were found in individuals who had the strongest English speaking skill at t1, as well as students who acquired more vocabulary than their peers at t2. We discuss the behavioral trajectory of reading development and implications for efficient ESL instruction.

**Metaphor comprehension in English as an additional language learner (EALL): evidence from eye movements**

**Annina K. Hessel, Victoria Murphy, Kate Nation**

University of Oxford, United Kingdom annina.hessel@education.ox.ac.uk

We asked whether EALL and monolingual English-speaking peers (N=42, 7;00-9;08 years) differ in reading comprehension, specifically for texts containing metaphors. Children read texts containing two types of metaphors and tightly matched literal control phrases: verbal (hours/birds fly by) and nominal (Jane is the queen-bee/music-lover) and eye movements were monitored. Children then answered questions about the metaphor/literal phrase. Metaphors were more difficult than literal controls for both groups of children (more regressions out, longer go-past and total times on the metaphor). This finding was mirrored in the offline comprehension data. Nominal metaphors were more difficult to read than verbal (more regressions in, longer total times on metaphors). An interaction effect revealed that largely, only nominal, but not verbal metaphors were more difficult to read than literal controls (longer total times on metaphors, more regressions out of post-metaphor region). This suggests nominal metaphors drive the main effect of difficulty of metaphors over literal counterparts. There were no differences between EALL and monolinguals on metaphor reading as revealed by eye movements, beyond some differences in early processing measures. However, their understanding on the offline comprehension task was impaired. These results suggest that EALL might not monitor their reading comprehension appropriately.

**Using Eye Movements to Investigate Cross-Language Syntactic Activation During Natural Reading**

**Naomi Vingron**^1^**, Jason Gullifer**^1^**, Veronica Whitford**^2,3^**, Deanna Friesen**^4^**, Debra Jared**^4^**, Debra Titone**^1^****^1^McGill University, Canada;

^2^Harvard University; 

^3^Massachusetts Institute of Technology; 

^4^: University of Western Ontario naomi.vingron@mail.mcgill.ca

An question within the study of bilingualism is whether bilinguals activate non-target syntax during natural reading, and whether L2 experience modulates this activation. English exclusively places adjectives before nouns (the red truck), whereas French typically places adjectives after nouns (le camion rouge). Here, we monitored eye movements of 27 French-English bilinguals (French=L1) as they read English sentences with English intact adjective-noun order, or violated adjective-noun orders that were consistent with French or anomalous (The truck red was parked on the street. vs. Red the truck was parked on the street.). First pass gaze durations (FPGD) on the constructions themselves (the red truck) were similar for French-consistent violations and English intact sentences. In contrast, total reading times (TRT) for English intact sentences were shorter than French-consistent sentences, and both were shorter than anomalous sentences. Finally, anomalous sentences were processed faster as the experiment progressed. These results suggest that French-consistent violations in adjective-noun order are processed more similarly to English intact adjective-noun order, while a different processing pattern was observed when a violated word order was inconsistent with either language. Taken together, the overall pattern of data suggests that bilinguals access non-target L1 syntax to some degree during L2 reading. 

**Reading first and second language comprehension texts in Sepedi and English among senior phase learners**

**Pheladi Florina Fakude**

North-West University, South Africa

24474134@nwu.ac.za 

Reading development in African languages remains under-researched and under-theorised to date despite a cornucopia of studies showing that successful acquisition of reading is vital for general cognitive. Native speaker often have poor reading skills in their native language even after 9-10 year of being in school. We recorded reading data from 30 native speakers of Sepedi (Grade 8 and 9 with Sepedi as the subject/language) before and after 8 weeks of extended reading program targeted at increasing exposure of Sepedi reading to native speakers of Sepedi. Participants read one of two simple texts and one of two academic texts in both Sepedi and English. We then used linear mixed effects modelling to show how the improvement in reading (as measured by global reading measures) could be predicted from attitude tests, fluency tests and learning data during the extended reading program. 

**Selective Attention of Second Language Readers**

**Caleb Prichard**^1^**, Andrew Atkins**^2^****

^1^Okayama University, Japan; 

^2^Kindai University, Japan prichard@okayama-u.ac.jp 

Effective second language (L2) readers frequently utilize global strategies, including selective attention to a text’s main points. While first language (L1) research has explored selective attention using eye tracking (e.g., Hyönä & Nurminen, 2006), L2 research is lacking. This research examines Japanese university students’ (N = 55) use of selective attention when reading a text to write a summary. Selective attention was measured by comparing fixation duration on the following areas of interest (AOIs): Global AOIs (title, introduction, and topic sentences) and Support AOIs (supporting sentences). The study also evaluates whether selective attention affects the participants’ ability to recall the text’s main points. The results showed that the participants did not fixate on the Global AOIs significantly more than the Support AOIs, t(54) = .57, p = .29. However, the participants who did fixate longer on the Global AOIs scored significantly higher on the summary, t(53) = 2.01, p < .05. This study suggests that most Japanese university learners do not read selectively, but the minority who do are better at recalling the text’s main points. These findings are similar to L1 research (Hyönä & Nurminen, 2006) and suggest that learners may benefit from instruction on reading strategies. 

**Task effects reveal cognitive flexibility responding to readers' level and word frequency: Evidence from eye movements for Chinese-English bilinguals during English reading**

Xin Li, Haichao Li, Jingyao Liu, Yongsheng 316, Xuejun Bai, Guoli 206 Tianjin Normal University, China lixinpsy1983@126.com

It is well-known that word frequency affect processing time during reading, and the word frequency effect changes magnitude across tasks. Kaakinen and Hyönä (2010) and Schotter et al. (2014) compared fixation times in natural reading and proofreading. The results showed that the frequency effects was larger in proofreading than in natural reading. In this study, we examined the effect of reading tasks and the readers' level for word frequency on Chinese-English bilinguals’ eye movement behavior during English reading. The mixed experimental design was used, which was 2 (reading tasks: natural reading, proofreading) × 2 (groups: high-level, low-level) × 2 (word frequency: high, low) design. According to their College English Test scores, thirty-four Chinese college students were divided into two groups. The results showed as follows. (1) Task instructions influenced the processing time for high-frequency and low-frequency words, especially for the low-frequency words. (2) The word frequency effects were significant both for the high-level and low-level group, especially for the low-level group. (3) The readers' level effects changed magnitude across tasks, and the reading tasks effect was more significant for the high-level group. Overall, the study suggested that word frequency effects and readers' level effects were significantly modulated by task demands.

**How EFL beginners and intermediate level students read story structures along with illustrations via eye-tracking techniques**

**Grace -Yi Hsieh, Sunny San-Ju Lin **

National Chiao Tung University, Taiwan grace.myhsieh@gmail.com 

The design of story structures (prologue/exposition, conflict, and resolution) displayed in three screen affects EFL beginners and intermediate-level readers. The attention of two groups (N=14 and N=21) reading text and illustration is a concern in this study. Some eye movements indicators were used in group comparison. The results revealed (1) Beginners were less efficient in reading, having less fixated time on each screen and encountered greater difficulties in comprehension on the first two screens than that in the intermediate level readers. In story ending, intermediate level readers were better at integrating information to reach comprehension. (2) The narratives drew attention once eyes entered the screen and pulled a major proportion of attention in all the way of reading, while the adjunct illustrations attracted only marginal attention in the later stage of reading. For the text areas, intermediate level readers could extract more meaningful chunks from the passages and easily processed sentences in each fixation location than that of beginners. Regarding narrative-illustration reference, the eyes of intermediate level readers looked at the narrative passages first and then immediately referred to key elements in illustrations to seek meaning confirmation. For beginners, they mainly read the text; illustrations rarely referred. 

**The influence of location information and word frequency on Chinese poly morphemic word recognition**

**Erjia Xu, Xue Sui**

Liaoning Normal Unversity, China suixue88@163.com

We used a boundary paradigm to investigate what is the role of morphemes position during Chinese poly morphemic word recognition and whether the word frequency would influence Chinese threecharacter words recognition. Experiment 1 examined how prime word duration time influence the flexibility of morphemes position coding by presenting words normally (e.g. 大自然) , transposed characters at a word’s end (大然自)or replaced the interior character(大吅然) as prime word and giving subjects semantic categorization task for target word . The effects of these transpositions were similar to alphabetic study, suggesting similarly flexible encoding of letter positions during reading. Experiment 2 examined the flexibility of Chinese morphemes position coding by presenting words in sentences as the same conditions in experiment1. The results indicate that replaced character word is difficult than transposed characters word in sentence reading. Both experiments also included a critical target word manipulation of word frequency, providing tests of whether the effects of letter position coding and stimulus quality are modulated by lexical processing. Together the results of these experiments have important theoretical implications for the nature of Chinese word recognition.

**Literal and Figurative Language Processing: Evidence from Bilingual Sentence Reading **

**Danielle dos Santos Wisintainer**^1,2^**, Mailce Borges Mota**^1,2^****

^1^Federal University of Santa Catarina - UFSC, Brazil; 

^2^Conselho Nacional de Desenvolvimento Científico e Tecnológico - CNPq, Brazil

wisintainer.ds@gmail.com 

Research on and figurative language has demonstrated that nonnative speakers process literal meanings faster than figurative meanings, due to difficulties in interpreting and understanding sentences with this kind of content. In the present study, the contrast in the processing of figurative and literal language was instantiated by online processing of phrasal verbs in English. Eye movements of 10 advanced speakers of English as L2 were compared to that of 10 native speakers of English during the reading of sentences containing figurative phrasal verbs, literal phrasal verbs and lexical verbs in English. Late measures (total reading time) showed that, compared to native speakers of English, the nonnative speakers of English had more difficulty processing figurative phrasal verbs than lexical verbs. These results were interpreted as evidence that the nonnative speakers of English tried to analyze each component of the figurative multiword item (e.g. to figure out) and this slowed down their processing. This behavior may have led them to revisit and reanalyze the region of interest, as previously reported by the related literature. The results are discussed in the light of theories on the processing of figurative and literal language.

**Reading and topic scanning in English and Chinese: Effects of word frequency and spacing**

**Sarah J. White**^1^**, Xiaotong Wang**^2^**, Li Hua Zhang**^2^**, Xue Sui**^2^****

^1^University of Leicester, United Kingdom; 

^2^Liaoning Normal University, China

s.j.white@le.ac.uk 

Eye behavior is compared during reading for comprehension and searching for a target word for Chinese text. The study provides an examination of whether lexical processing of words occurs during search for a target word, as well as reading for comprehension, in Chinese. The design was 2 (task: reading, searching) × 2 (critical word frequency: high frequency, low frequency). Participants completed two blocks of trials, a reading block and a searching block. Experimental sentences included a critical word (high or low frequency). There were also filler sentences within each block, each of which included the search target word. The experimental sentences never included the target word. For the experimental items, sentence reading times were longer than search times. For the critical words, there were significant effects of word frequency for reading for comprehension, but not searching. The results indicate that lexical access does not usually occur during search for a target word within Chinese text. These results are in line with those of Rayner and Fischer (1996) for reading and searching in English. Together the results indicate that search for a target word may be achieved by visual form matching regardless of the type of orthography. 

**Eye movements in reading global and local syntactic ambiguity in Russian **

**Victor Anisimov**^1^**, Olga Fedorova**^2^**, Leonid Tereschenko**^1^**, Alexander Latanov**^1^****

^1^Dept. of Higher Nervous Activity, M.V. Lomonosov State University, Moscow, Russia;

^2^Dept. of Theoretical and Practical Linguistics, M.V. Lomonosov State University, Moscow 

Russiaviktoanisimov@ya.ru

We studied eye movement parameters in reading syntactically ambiguous sentences with relative clause ambiguity in native Russian speakers. They disambiguated globally ambiguous (G), locally ambiguous with early (E) and late (L) closure, and unambiguous (U) sentences. The reading times while reading L and E but not G were significantly longer than in reading U. Less fixation numbers and regression frequencies while reading U compared with those for L and E determined shorter reading time for U. The increase in total reading times indicates more mental efforts in disambiguating E and L. We did not reveal any differences between eye movement parameters in reading U and G. Consequently, disambiguating G was a little more difficult than analysis of U. Noteworthy results consisted in absence of differences between reading times while reading G and E. However, reading time in reading L was longer than reading time for G and E because of less fixation numbers and regression frequencies in reading G and E. This implies difficult disambiguating L in Russian with early closure domination. Our results in the opposite way coincide with results in English with difficult disambiguating E assuming late closure domination. Supported by Russian Foundation of Basic Research.

**Effects of counterargument construction instruction and viewpoint presentation order on reducing myside bias in reading texts regarding controversial issues**

**Miao-Hsuan Yen**^1^**, Ying-Tien Wu**^2^

^1^Graduate Institute of Science Education, National Taiwan Normal University, Taiwan; ^2^Graduate Institute of Network Learning Technology, National Central University, Taiwan myen@ntnu.edu.tw

Myside bias (i.e., longer viewing time on myside than other-side information) has been observed during reading texts regarding controversial issues. Based on previous findings of the authors that participants (college students) who could generate counterarguments against their own arguments paid more attention to other-side than myside information during reading, an instruction for counterargument construction was developed. Seventy-three college students read two controversial issues about nuclear power and genetically modified food, each with six pages presenting various aspects of the issue. Before the instruction, they read one of the issues freely; after the instruction, they read the other issue with a focus on counterarguments. Both myside and other-side information were presented sideby-side on the same page, with a between-participant manipulation of viewpoint presentation order (i.e., myside or other-side information was presented first). Both effects of the presentation order and counterargument instruction on reducing myside bias were obtained on word-based total viewing time. Those who read the other-side-first text paid more attention to other-side information before and after the instruction. Those who read the myside-first text spent more time on myside information before the instruction and spent similar amount of time on both sides after the instruction. 

**The of Tasks and Signals on Text Processing for Readers with Different Strategies**

**Shouxin Li**^1^**, Dexiang Zhang**^1^**, Zhaoxia Zhu**^1^**, Yuwei Zheng**^2^****

^1^Shandong Normal University, China; ^2^University of Jinan, China,People's Republic of Shouxinli@sdnu.edu.cn

The purpose of present study was to examine the effects of reading tasks and signals for readers with different strategies by using eye-tracking. Experiment 1, readers were instructed to read two texts either guided by a summary task or a verification task. Experiment 2, readers read two texts with or without signals which were underlines of the topic sentences. A clustering technique distinguished four groups: topic structure processors (TSPs), slow linear readers (SLRs), nonselective reviewers (NSRs), and fast linear readers (FLRs). The results showed that the TSPs could adjust their reading strategies according to reading tasks and they adopted structure strategy in summary task meanwhile they adopted linear strategy in the verification task; however, they adopted structure strategy in signal and without signal conditions. The SLRs and NSRs used structure strategy in summary task, whereas they used linear strategy in the verification task, but the FLRs used structure strategy in both tasks. Providing signals guided the SLRs, NSRs and FLRs to switch from linear strategy to structure strategy. These results suggested that the effects of tasks and signals are inconsistent for readers with different strategies.

**Eye movement correlates of absorbed literary reading**

**Moniek Kuijpers, Sebastian Wallot**

Max Institute for Empirical Aesthetics, Germany moniek.kuijpers@aesthetics.mpg.de

Absorption is an important factor in reading and enjoying literary narratives. Since one of the characteristics of absorbed reading is that a reader loses self-awareness, it is important that we not only rely on subjective measures of absorption completed after reading, but also find objective measures to capture absorption during reading. Relatively little is known, however, about the objective markers, such as eye movement characteristics, that distinguish an absorbing reading experience from a nonabsorbing one. In the present study we investigate natural reading across two different texts (low absorption/high comprehension versus high absorption/high comprehension). We will test the hypotheses that participants who feel more absorbed show on average higher pupil dilation, lower eye blink rate, fewer gaze regressions, fewer gaze durations, and shorter reading time (i.e., higher processing fluency). We will also investigate how absorption experience progresses (e.g., are readers generally more absorbed further along in the text?) and explore possible textual features related to this progression. This research is work in progress: we are currently collecting data for the first study and have planned two more studies in which we plan to distract our participants during reading to add a measure of reaction time to capture absorption. 

**The role of defaultness and personality factors in sarcasm interpretation: **

**Evidence from eye-tracking during reading**

**Ruth Filik**^1^**, Hannah Howman**^1^**, Christina Ralph-Nearman**^1^**, Rachel Giora**^2^****

^1^University of Nottingham, United Kingdom; 

^2^Tel Aviv University, Israel ruth.filik@nottingham.ac.uk

Traditionally, theorists have debated whether our ability to understand sarcasm is principally determined by the context (e.g. Gibbs, 1994; Utsumi, 2000), or by properties of the comment itself (e.g. Giora, 2003; Grice, 1975). The aim of the current research was to investigate an alternative view; that negation generates nonliteral interpretations by default (e.g. Giora, Givoni, & Fein, 2015). In Experiment 1, we monitored participants’ eye movements while they read affirmative and negative utterances, such as “He [is/isn’t] the best lawyer”. When presented in isolation during a pre-test, affirmative phrases received a literal interpretation, and negative phrases a sarcastic one, demonstrating their default interpretations. In the eye-tracking study, prior context was used to bias target utterances equally strongly towards either a literal or sarcastic interpretation. Results showed that target utterances were easier to process when they appeared in contexts supporting their default interpretations. Results from a second eye-tracking experiment suggest that a reader’s tendency to interpret negative phrases sarcastically is related to their level of indirect aggression. Our findings suggest that negation leads to certain ambiguous utterances receiving sarcastic interpretations by default (which cannot be explained by traditional theories), and that this process may also be influenced by personality factors.

**Cognition and Learning**

**Lab - Field Comparisons on Intra-Subject Variability of Eye Movements**

**Divya P. **^1^**, Holger Hill**^^[1]^^**, Chara Ioannou**^1^**, Nadine Penkalla**^1^**, Giuseppe Boccignone**^3^**, Tom**

**Foulsham**^4^**, Christian Fleischhaker**^1^**, Monica Biscaldi**^1^**, Ulrich Ebner-Priemer**^2^**, Christoph Klein**^1,5,6^

^1^Department of Child and Adolescent Psychiatry, Medical Faculty, University of Freiburg, Germany; 

^2^Institute of Sports and Sports Sciences, Karlsruhe Institute of Technology, Germany; ^3^Department of Computer Science, University of Milan, Italy; ^4^Department of Psychology, University of Essex, United 

Kingdom; ^5^School of Psychology, Bangor University, United Kingdom; 6Department of Child and Adolescent Psychiatry, Medical Faculty, University of Cologne, Germany****divya.seernani@uniklinik-freiburg.de

Research findings, particularly with clinical groups, have favored and emphasized the importance of ecological validity (Berkley, 1991). Intra-subject variability (ISV), i.e., moment to moment performance fluctuations, is the most prominent finding in ADHD literature. Research on its ecological validity has been largely neglected. We proposed a lab-field comparison on a battery of tasks with high ecological validity.Adult participants (N=30) were invited for a lab eye-tracking session using SMI RED250, and a field testing employing SMI ETG2.0. In each session, participants performed everyday tasks, such as copying something down, visual search, and viewing pictures. All lab tasks were performed in isolation on a computer. Field tasks were performed in the presence of other participants, and employed projections and objects. However, stimuli were standardized and controlled across lab and field analogues to ensure ISV testing.Preliminary analysis, shows a gradual decrease in correlation of field analogues to the lab tasks, as we move from tasks most similar to the lab (screen presentation versus a projection of stimuli), to those more ecologically valid (screen presentation versus a picture book).To our knowledge, ours is the first study to systematically study ISV using novel eye-tracking tasks, in a comparison across devices and set-ups 

**Smart Detection of Driver Distraction Events **

**William D. **^1^**, Catherine Deegan**^2^**, Charles Markham**^1^****

^1^Maynooth University, Ireland; 

**The Influence of Light-Induced Dynamics on Attention, Perception, and Driving **

**Behavior: A Real-World Driving Study**

**Markus Grüner**^1^**, Peter Hartmann**^2^**, Ulrich Ansorge**^1^**, Christian Büsel**^1^****

^1^University of Vienna, Faculty of Psychology, Austria; 

^2^ZKW Group GmbH, Austria markus.gruener@univie.ac.at

During night driving, car headlamps are the most important assistance for the driver. New technology using adaptive driving beams aims to provide optimal lighting in all driving situations. These lighting systems avoid glaring oncoming and preceding cars by adjusting the light beam, while at the same time keeping the rest of the scene highly illuminated. Although a better illumination is usually associated with higher traffic safety, this technology also introduces novel light-induced dynamics caused by the adjustment of the light beam. So far, the effects of these light-induced dynamics on attention, perception, and driving behavior are not understood. Using mobile eye tracking during real world night driving, we investigated the influence of light-induced dynamics on eye movements. The results showed that light-induced dynamics attracted fixations, even when these dynamics did not provide drivingrelevant information. Pronounced light-induced dynamics tended to attract fixations even more than subtle dynamics. In a follow-up study, we investigate how light-induced dynamics influence visual attention and driving behavior since the first results suggested that light-induced dynamics might distract drivers and thus potentially jeopardize object and hazard recognition. 

**Investigating Feedback Processing with Eye Tracking **

**Kim Dirkx , Jarodzka Halszka , Desiree Joosten-ten Brinke **Open University, Netherlands kim.dirkx@ou.nl

Feedback is one of the most effective interventions in education (Hattie & Timperley, 2009). Although there is quite some research on different types of feedback on revising written assignments by students (e.g., corrections, explanations, questions), there is little known about the effects of different types and modes (via track changes or comments in the text). This study investigates the following research question: ‘How do mode and type of feedback affect attention allocation and revision making of students?’To investigate the research question, 15 students receive feedback on their written assignment. While they processes this feedback, attention allocation is captured using eye tracking.Pilot results (n=4; Mean age = 20; 75% female) show that students process feedback initially from the top to the bottom of the assignment but then tend to focus on ‘easy to revise’ comments and tend to neglect question-based feedback. On 10, 11, 19 and 20 April 2017 – at the end of a course on questionnaire construction of the OUNL - the actual data collection is scheduled.Feedback type and mode are expected to steer attention allocation and revision making. Based on the results of this study, guidelines for type and mode of feedback will be formulated.

**Sleep deprivation systematically changes eye movement characteristics **

**Justine Winkler**^1^**, Ricardo R. Gameiro**^1^**, Peter König**^1^**, Daniel Aeschbach**^2^**, Christian Mühl**^2^

^1^Institute of Cognitive Science, University of Osnabrueck, Germany; ^2^Institute of Aerospace Medicine, German Aerospace Center (DLR), Cologne, Germany juwinkler@uos.de

During our day-to-day life, sleepiness endangers the safety of us as well as of others. Specifically, it impairs operator performance in security-related working environments, for example in aviation. To reduce the number and impact of sleepiness-related accidents, easy to handle monitoring methods are needed. Here, we investigate the relationship between gaze as a measure of visual attention and sleepiness. After two days and two nights of adaptation in the sleep laboratory, we randomly assigned subjects to the treatment group with 24 hours of sleep deprivation. Control subjects were allowed to sleep normally. The day before and the day after the treatment night we measured their eye movements during free viewing of natural images. The results indicated a decrease of visual exploration behavior for sleep-deprived subjects, mirrored by a reduction in the entropy of the spatial distribution of fixation locations. Likewise, sleep deprivation led to shorter saccade amplitudes. On the other hand, we did not find significant effects on the number of fixations. In contrast to control subjects, the median single fixation duration decreased in sleep-deprived subjects. Overall, our findings suggested that sleep deprivation leads to systematic changes in eye movement characteristics that can be distinguished from normal viewing behavior.

**Applying head-mounted eye-tracking to investigate cultural differences in realworld face scanning **

**Jennifer X. Haensel , Tim J. Smith , Atsushi Senju **

 Birkbeck, University of London jhaens01@mail.bbk.ac.uk

Previous eye-tracking studies investigating cultural differences in face scanning have been restricted to screen-based paradigms. To examine whether findings generalise to naturalistic settings, this study used head-mounted eye-tracking in 20 Eastern Asian (EA) and 20 Western Caucasian (WC) dyads to compare their scanning strategies while they introduced themselves and played story-telling games. We developed semi-automatic tools that dynamically track regions of interest (upper/lower face) and classify gaze points accordingly. Both groups showed significantly more face gaze when listening than when speaking. Cultural differences were observed during speaking, with increased face gaze at the listening partner in EA compared to WC participants. Contrary to predictions, no group differences were found for duration of upper face scanning or mutual gaze, questioning the notion of gaze avoidance in EA individuals (Argyle et al., 1986). We also employed a data-driven approach whereby face regions and gaze points are mapped into a normalised space to generate difference maps of gaze density. Initial qualitative results revealed that EA observers showed more localised eye scanning, whereas WC observers exhibited greater gaze distribution. This replicates screen-based studies using emotionally expressive faces (Jack et al., 2009) and demonstrates cultural differences in naturalistic face scanning for the first time.

Presentation Parameters Affecting Effects in the Visual World Paradigm

**Marie-Anne Morand , Constanze Vorwerg **

 of Bern, Switzerland marie-anne.morand@students.unibe.ch 

One question addressed by means of the visual world paradigm is lexical competition during spoken word recognition, as determined by eye movements to concurrent visual objects that are phonological competitors to the target object, i.e., whose names have an initial segmental overlap with the presented object name. The detection of a parallel activation of lexical items may, however, be limited by finding the right presentation parameters.A methodological experiment tested the dependency on presentation conditions of competitor fixations. Participants (N=20) were presented with displays of four visual objects, one of which was specified by an auditory (Swiss) Standard German object name, and clicked on this target object. In critical items, there was a phonological competitor. In control trials, there was a control object instead (no phonological onset overlap). A systematic manipulation of preview time (1000 … 4000 ms, spaced at 500 ms intervals), picture size (170 x 170, 240 x 240, 350 x 350 pixels), picture location (outer, middle, inner position) (all within participants), and familiarization (yes/no, between participants) revealed a significant influence of all three picture presentation factors. The results suggest that sensitivity to presentation parameters needs to be taken into account in design and interpretation. 

**Predicting Information Context Processing from Eye movements **

**Saurin Sharadkumar Parikh**^1^**, Hari Kalva**^2^

^1^Florida Atlantic University, United States of America, Nirma University, India; ^2^Florida Atlantic University, United States of America 

sparikh2014@fau.edu 

Word is critical for learning a concept. A cognitive response to a novel word stimuli is information context processing, which may impact eye movements. For predicting information context processing behavior in real time, we have analyzed seven eye movement features: (i) second pass time (SPT) for novel term and informative context, (ii) total no of regressions (RC) into novel term / informative context (iii) Laminarity fine detail (LFD), (iv) Laminarity Re-Glance (LRG), (v) Determinism 

(Dm), (vi) mean pupil diameter (RA-Pupil-Diameter), measured during reanalysis of a word and (vii) Gaze duration. A basic predictive model developed on basis of this theory, shows a prediction accuracy of 71%.This approach gives 15% improvement over previous published methods. ANOVA result for quantitative features such as: SPT and "RA-Pupil-Diameter" shows p-value < 0.05 and chi square test of Independence for categorical features such as :LFD and RC is > 3.62 and its corresponding p-value is < 0.05. These results indicate a strong correlation of features such as RC, LFD, SPT, "RA-PupilDiameter" with Learning difficulty and these features are used to predict information context processing behavior.

**Confidence in perceptual judgments preceding eye movements **

**Monique Michl, Wolfgang Einhäuser**

Chemnitz University of Technology, Germany monique.michl@s2012.tu-chemnitz.de

Perceptual performance at a saccade's target improves prior to saccade execution, indicating presaccadic allocation of covert attention to the target (Deubel & Schneider, 1996). We investigated whether observers are aware of this attentional benefit; that is, whether observers' confidence adjusts to performance or relies on stimulus properties. Observers performed saccades to one of two locations located equidistant to initial central fixation. At each location a Gabor wavelet was briefly presented and masked prior to the saccade. After the saccade, observers reported the orientation of one of the Gabors. In some conditions, observers were cued after the saccade on which Gabor to report ("forced report"), in other conditions, they were free to choose either ("free choice"). For Gabors of the same contrast, performance was better at the saccade target, in line with Deubel & Schneider’s classical finding. In free-choice trials, however, observers tended to select the Gabor of higher contrast, even if this choice was suboptimal with respect to performance. Only if contrasts at both sides were nearly identical, the target location was preferred. This suggests that observers are not fully aware of their pre-saccadic attentional benefit: they base confidence in perceptual judgements on stimulus strength rather than on expected performance.

**The relationship between performance in the anti-saccade task and memory for paintings **

**Tobiasz Trawinski**^1^**, Natalie Mestry**^2^**, Simon P. Liversedge**^1^**, Nick Donnelly**^1^****

^1^University of Southampton, United Kingdom; 

^2^Bournemouth University, United Kingdom 

T.Trawinski@southampton.ac.uk

We explored discrimination of previously viewed paintings from new foils in participants who were art novices. In the first phase participants were presented with 100 paintings, twenty from each of five different motif categories. Participants saw a presentation order that was fully randomized, grouped by motif, or grouped by motif with additional information. In a second phase performed 30 minutes later, participants completed an old/new discrimination task where half of the paintings shown in the first phase were presented along with 50 new paintings from the same motif categories. Eye movements were measured in both phases of the experiment. Participants also completed a set of individual difference measures, including measures of working memory capacity and the anti-saccade task. The results showed excellent discrimination of paintings from 4 of the 5 motif categories. Sensitivity was positively associated with performance on the n-back task consistent with sensitivity being related to larger working memory capacity. Reduced inhibitory control (as measured by the antisaccade task) was associated with increased sensitivity and an increased number of fixations at encoding. We suggest that reduced inhibitory control of eye movements facilitates recall by increasing sampling of paintings at encoding.

**A closer look at numbers in simultaneous interpreting: An eye-tracking study **

**Pawel Korpal**^1^**, Katarzyna Stachowiak**^2^

^1^Adam Mickiewicz University in Poznań, Poland; 

^2^University of Warsaw pkorpal@wa.amu.edu.pl

Why study numbers in simultaneous interpreting? Interpreting is a challenging task, where several activities are conducted concurrently: listening, speaking, self-monitoring (Gile 2009). At the same time, numbers are difficult to process because numerical data cannot be derived from the context and they need to be rendered in a word-to-word manner (Mazza 2001). Number processing is dependent on modalities (auditory vs. visual) and codes (verbal vs. Arabic). The difficulty in number interpreting lies in the necessity of constantly transforming the content from one code to another and manipulating it across modalities.

We aimed to verify whether seeing numbers, apart from hearing them, affected the process of simultaneous interpreting performed by professional interpreters (N=25) and interpreting trainees (N=25). In addition, we manipulated the speaker’s delivery rate. Data were recorded with EyeLink 1000+ eye-tracker. Our results show that interpreters look at numbers on the screen which is reflected in high fixation count and rate. Having access to numbers in two modalities improved interpreting accuracy (p<.001). We also observed that high delivery rate negatively affected interpreting accuracy (p<.001). Finally, there were significant inter-group differences in fixation count (p=.047) and interpreting accuracy (p=.004), i.e. trainees provided less accurate interpretations and they looked more at numbers. 

**Is parallel language activation modulated by simultaneous interpreting expertise? **

**Laura Keller**

University of Geneva, Switzerland laura.keller@unige.ch

Parallel language activation in bilinguals has been widely observed. Recent findings indicate that the degree of non-task-relevant-language activation is conditioned by individuals’ executive functions and modulated by their language use. As professional simultaneous interpreters possess largely unexplored expertise in an extreme form of language use, this study aims at investigating whether their parallel activation patterns differ from those of naïve multilinguals. Furthermore, it sets out to exploit the diglossic dichotomy characterizing Swiss speakers of German, to extend the parallel activation observations to varieties of the same language, i.e. Swiss German in a Standard German setting. For this purpose, four participant profiles were recruited for a visual world eye-tracking experiment (diglossic interpreters, non-diglossic interpreters, diglossic non-interpreters and nondiglossic non-interpreters; testing ongoing, tested n=60; L1=DE, L2=EN). Participants are instructed to click on a target object image presented on a screen with a phonological competitor and two fillers (three fillers in the baseline condition). The phonological competitors belong to the task-relevant language variety (Standard German) or to the task-irrelevant variety (Swiss German). A time course analysis of fixation distributions together with the analysis of target identification times are expected to allow for conclusions in terms of interpreting expertise potentially influencing competition resolution.

Can you see what I’m saying? Eye movements and bilingual spoken language processing in conference interpreting

**Katarzyna Stachowiak**

University of Warsaw, Institute of Applied Linguistics, Poland km.stachowiak@uw.edu.pl

Eye movements were shown to reflect spoken language processing and/or mental imagery (Richardson and Spivey 2000, Spivey and Geng 2001, Johansson 2013). However, there is still a paucity of studies on eye movements in bilingual spoken language pro-cessing.

The study aimed to demonstrate that eye movements can act as markers of integrated visual and auditory processing during language perception and production in consecutive and simultaneous interpreting. Two groups of participants: professional interpreters and interpreting students were asked to interpret short speeches including “high-imagery” items (Just et al. 2004), i.e. sentences referring to four cardinal directions: north, south, east and west (e.g. “For instance, in the north of Poland the technology of generating energy from sea waves is being developed”). At the same time, the participants were looking at pictures congruent with the auditory input, incongruent with it or – at a blank screen. Mean fixation rate in response to congruent vs. incongruent cross-modal input (and in the blank screen condition) was calculated and compared in two groups, which showed big withinsubject and between-group differences. Apart from this, the results indicate that the participants’ eyes follow the imaginary north-south, east-west path while listening to and speaking in the bilingual context. 

**Evidencing the emergence of sensorimotor structures underlying proportional reasoning **

**Shakila Shayan**^1^**, Loes Boven**^1^**, Arthur Bakker**^1^**, Marieke van der Schaaf**^1^**, Dor Abrahamson**^2^

^ 1^Utrecht University, Netherlands; 

^2^University of California at Berkeley, United States of America 

s.shayan@uu.nl

Eye tracking bears exciting new methodological affordances for mathematics education research. When combined with other data, such as hand-movement logging and audio–video recording of multimodal utterance, eye tracking can assist in inferring the sensorimotor schemes students develop and deploy as they engage in manipulation problems. From an enactivist perspective, these schemes constitute the cognitive substance for building more formal understanding of mathematical concepts. Over the past several years, we have been investigating an empirical context centered on a design architecture called the Mathematical Imagery Trainer so as to understand systemic factors leading to students developing task-effective bimanual coordinations presumed to underlie proportional reasoning. During the presentation, we will report on findings from a succession of empirical studies centered specifically on an educational tablet application. Analyzing the emergence of bimanual kinesthetic patterns, we demonstrate that students achieved the enactment of these task-effective movements by way of inventing attentional anchors—perceptual structures organizing goal-oriented motor action. With reference to a database of over 100 students in primary and prevocational education, we show how students come to focus their attention on mathematically relevant areas of interest, and how they progress from incorrect, low-level strategies to high-level strategies when solving the proportional tasks.

**From lenses movement to cognitive processes: What new insight may eye tracking provide **

**Gustavo Gasaneo**^1^**, Maria L. Freije**^1^**, Juan I. Specht**^1^**, Adrian A. J. Gandica**^1^**, Claudio A. Delrioux**^2^**, Borko Stosic**^3^**, Tatijana Stosic**^3^

^1^NEUFISUR - Universidad Nacional del Sur, Argentine Republic; 

^2^DIEC - Universidad Nacional del Sur, Bahía Blanca, Buenos Aires, Argentina; ^3^Department for Statistics and Informatics, Federal Rural University of Pernambuco, Brazil ggasaneo@gmail.com

The registration of the eye movements is turning into common practice in games, business, marketing, science, and other areas. It is also turning into an important tool for diagnosis of diverse medical or psychological conditions like AAD, Autism, Alzheimer desease, etc. In this work we analyze the high frequency information obtained when measuring the eye movements of persons performing different cognitive tasks. First, from the data record we identify the relevant information about the dynamic of the eyeball and the eye lens. A model for the dynamic is presented, which includes parameters associated with the damping of the system and the elasticity of the eye muscles and lenses. In addition, using Multifractal Detrended Fluctuation Analysis and autocorrelation function, we identify the characteristics associated with the different cognitive paradigms. References T. Stosic and B. D. Stosic, IEEE TRANSACTIONS ON MEDICAL IMAGING 25, 1101 (2006) 

**Methods, and innovative Technology **

**Statistical analysis of eye movement sequences using spatial point processes **

**Anna-K. Ylitalo **

 University of Jyväskylä, Finland anna-kaisa.ylitalo@jyu.fi

Spatial point process statistics are applied for analysing data consisting of a set of events located on a space, which is typically a two-dimensional region. Sequential spatial point processes are models for sets of spatial events provided with information of their order. In the case of eye movement data, fixations can be considered as such kind of events when measured with an eye tracker. Moreover, eye movement data include information on the occurrence times and durations of fixations, which can be attached as additional information to the events in the process. By taking into account the order of fixations together with their locations, we present a sequential point process model in order to decompose features in the eye movement process. As an empirical example, the model is fitted to an eye movement sequence collected when a subject has been looking at a picture of painting for three minutes. We describe the evolution of the process by functional summary statistics, such as convex hull coverage, and show how the model can be used to assess the statistical uncertainty related to the summary statistics. The uncertainty envelopes can be further used in comparing fixation processes between the individuals.

**Study of and saccades when viewing holograms, stereo images, and **

**2D images **

**Taina M. Lehtimäki**^1^**, Mikko Niemelä**^2^**, Risto Näsänen**^3^**, Ronan G. Reilly**^1^**, Thomas J. Naughton**^1^

^1^Department of Computer Science, Maynooth University-National University of Ireland Maynooth, Ireland; 

^2^Former affiliation: Oulu Southern Institute, University of Oulu, Nivala, Finland; ^3^Institute of Behavioural Sciences, University of Helsinki, Helsinki, Finland taina@cs.nuim.ie

Digital holographic three-dimensional (3D) displays have been demonstrated that allow realistic 3D perception of a scene. Our long term aim is to contribute to the understanding of the visual perception requirements for this new generation of 3D holographic displays. The visual properties of glass-plate holograms allows them to act as idealized digital holographic displays for still images. They provide all depth cues including defocus blur, accommodation, convergence, motion parallax, and binocular parallax; features not available with traditional stereoscopic displays. We conducted a binocular eye movement study using an EyeLink II eye tracker. We had five observers, seven glass-plate holograms, and three tasks in our study. We investigated how eye movement parameters differ between viewing holograms and conventional stereo and 2D images. Stereo and 2D image stimuli were created by taking photographs of the holograms along parallel optical axes and were displayed with a polarised stereoscopic display. We present the experimental eye tracking laboratory set-up for glass-plate holograms and the results of the study including fixation duration, saccade amplitude, and blink rate analysis. We found that the fixation durations, and participants’ perception of depth, were greater when viewing hologram stimuli compared with equivalent stereo or 2D stimuli. 

**Using gaze data to evaluate text readability: a multi task learning approach **

**Ana Gonzalez-Garduño**

 University of Copenhagen, Denmark fcm220@alumni.ku.dk

Predicting the level of difficulty of a text, or its readability, has many potential applications such as assisting second language learners or individuals with reading problems, aiding technologies to perform better text summarizations or translations and information retrieval. In this study, a multi task learning approach is presented for predicting text readability. Multi task learning (Caruana 1998) allows to improve the performance of multiple classification tasks by learning them in parallel. As longer reading times are known to correlate with less readable texts (Lapata 2006) , the goal of this study is to use the Dundee eye tracking corpus which includes measures such as first pass duration, total fixation duration and regression duration, in addition to several syntactic features known to affect text complexity, to improve the induction of Long Short Term Memory models (LSTMs) for the task of text readability. Preliminary results show improvements from the baseline (competitive to state of the art) when predicting whether a sentence belongs to a simple versus a normal wikipedia english article. Further experiments aim to extend these results to show that this method can be succesful for other datasets, including common core U.S. state standards and weebit(Vajjala et al 2012).

**Parsing Pupil and Eyeball Movement in Camera-based Eye-tracker Output **

**Jun Bao , Richard Shillcock**

 University of Edinburgh, United Kingdom 

844480945@qq.com

Postsaccadic (PSO) is the wobbling of pupil and lens at saccade end (Nyström&Holmqvist, 

2010). Lenswobblinglasts for 30–100ms before fading to 5% of initial amplitude (Tabernero&Artal, 2014). To understand PSO and inform a new classification of saccade, PSO and fixation, we developed a model of the movement of eyeball and pupil. In the model, the eyeball movement imitates muscle activity during a typical point-to-point eye-movement, plus a damping force; the pupil movement follows the eyeball movement by an elastic force based on how far it deviates from the centre of the iris, and a damping force. A full mathematical description of the model was obtained by solving second-order differential equations. Using gradient descent, we fitted the model to the saccade-tofixation trajectory (saccade length = 3 degrees) extracted from the reading data of the left eye of one person, which yielded a damping ratio of 0.40 and a natural frequency of 27.0Hz for pupil PSO. The model allows us to separate eyeball and pupil movement in real time. It can be applied to eye events detection algorithms, and more importantly to the calibration of an eye-tracker to yield more accurate eye position data. 

**Extracting Saccade-to-fixation Trajectory From Eye Movement Data in Reading **

**Jun Bao , Richard Shillcock**

 University of Edinburgh, United Kingdom 

844480945@qq.com

Postsaccadic oscillation (PSO) is the wobbling of pupil and lens at the end of each saccade 

(Nyström&Holmqvist, 2010). wobblinglasts 30–100ms before fading to 5% of initial amplitude (Tabernero&Artal, 2014). To study PSO, we need to extract and summarize eye movement trajectories from eyetracker output. We developed a set of algorithms to automatically mark and extract binocular saccade-to-fixation trajectories from the raw sample points in ASC files recorded by an Eyelink2eyetracker. First, a Python DataFrame collects the samples in all ASC files. Second, we marked peaks of velocity as saccades using an adaptive threshold (Nyström&Holmqvist, 2010).Third, we collected trajectories of each saccade with its following fixation, and calculated its properties; we selected a number of saccade-to-fixation trajectories with a saccade length of 3 degrees from one person. Fourth, an algorithm was applied to these trajectories to align them on the time axis. Fifth, outliers of these trajectories were removed by an algorithm adapted from TROAD (Lee, Han, & Li, 2008). The resulting saccade-to-fixation trajectoriessuggest that glissades are artifacts of event detection algorithms; they are the first peak of the PSO.

**Data-driven Gaze Event Classification for the Analysis of Eye and Head Coordination By Natural Task. **

**Gabriel J. Diaz**^1^**, Reynold Bailey**^2^**, Chris Kanan1, Mychal Lipson**^2^**, Jeff Pelz**^1^**, Rakshit Kothari**^1^

^ 1^The Carlson Center for Imaging Science, Rochester Institute of Technology, United States of America; 

^2^Computer Science, Rochester Institute of Technology, United States of America gabriel.diaz@rit.edu

Gaze is studied using computer screens that occupy only a portion of the observer’s field of view. However, in the real world, humans often make coordinated shifts of the eyes and head that extend beyond this narrow portion of the visual field. In this study, we seek to measure the relative prevalence of gaze events such as fixations, saccades, VOR, and smooth pursuit when movements of the eyes and head are unrestrained and coordinated by a task. Several subjects performed natural tasks while the angular velocity of head rotation was recorded using a head-worn inertial measurement unit, and rotational eye velocity was recorded using a 120 Hz SMI Wireless ETG2. A small group of experts manually annotated a subset of the data as periods of fixation, saccade, smooth pursuit, vestibulo-ocular reflex, and “object tracking,” (eye+head pursuit, with catch-up saccades). Inter-labeler reliability was measured to generate a confidence metric and a cost function for misclassifications. These statistics were then used to train a machine learning classifier of gaze events. In this poster, we present a preliminary analysis of our results, including statistics on the coordination of eye and head movements during natural tasks. 

**Assessment of Two Low Cost Eye Trackers**

**Shanmugaraj Madasamy**

ITB, Dublin, Ireland innoraj@gmail.com 

Eye tracking technology is becoming cheaper and used across diverse fields including psychology, cognitive science, market analysis, medical research, human computer interaction and vision research. Several researches have been conducted on exploring eye-tracking technologies but there is no enough research on comparing eye trackers. This paper provides various characteristic features by comparing two low cost eye trackers. We have designed a simple experiment to compare both Eyetribe and GP3 Gazepoint eye tracker. Some of the characteristic features used to evaluate the eye trackers: accuracy, reliability, precision, visual angle, preparation task, distance, system latency, calibration, physical dimension and API capabilities. The results from our study reveal Eyetribe is more efficient and flexible for driving simulator experiments.

**Mobile eye tracking: Reliability in assessing saccadic eye movements in reading **

**Alexander Leube**^1^**, Katharina Rifai**^1,2^**, Siegfried Wahl**^1,2^****

^1^Eberhard Karls University Tuebingen, Germany; 

^2^Carl Zeiss Vision International GmbH, Aalen, Germany alexander.leube@uni-tuebingen.de

Eye tracker (ET) with low sampling rates leave small saccades undetected in standard velocity-based detection algorithms. This study evaluates the influence of the sampling rate of a mobile ET (SMI ETG, 60 Hz and 120 Hz) on the reliability of fixation and saccade detection in a reading task. Gaze of 11 participants was recorded by the mobile ET’s simultaneously with a highly sampled remote ET (EyeLink, 1000 Hz) as reference. Analysis was performed with a standard velocity based algorithm and a saccade threshold of 60 °/sec. Higher sampling indeed lead to higher detection rate of saccades (Δ = +12.25 %, p = 0.011, t-test) and a more reliable estimation of saccade durations (Δ = 5.91 ms, p = 0.033, t-test). No significant difference was found in the detection rate of fixations (p = 0.110, Wilcoxon). Although, the 60 Hz mobile ET reveals an underestimation of fixation durations (Δ = 10.55 ms ± 10.13, p = 0.006, ttest), and the assessment in the 120 Hz tracker is more reliable (Δ = 4.30 ms ± 14.33 ms, p = 0.59, Wilcoxon). Thus, a higher sampling rate in mobile tracking leads to more reliable estimation of saccade and fixation statistics in reading. 

Is There a “Paperback” Option in the Domain of Eye Trackers? A New Approach for Comparing Devices 

**Agnes Scholz**^1^**, Johannes Titz**^2^**, Peter Sedlmeier**^2^

^1^University of Zurich, Switzerland; 

^2^Technische Universität Chemnitz, Germany agnes.scholz@psychologie.uzh.ch 

The popularity of the eye tracking method is steadily increasing as is the number of low-cost devices for measuring eye movements. At the same time hardly any methods exist that would allow to test if different eye trackers actually measure the same gaze behaviour. We propose a simple approach for comparing two eye trackers by adopting a method well-known to psychologists: correlating constructs to show reliability and validity. In a laboratory study, we ran the low-cost EyeTribe eye tracker and an established SensoMotoric Instruments eye tracker at the same time, positioned one above the other. This design allowed us to correlate the eye-tracking metrics of the two devices over time. The experiment was embedded in a research project on memory where 26 participants viewed pictures or words and had to make cognitive judgments afterwards. The outputs of both trackers, that is, the pupil size and point of regard, were highly correlated, as estimated in a mixed effects model. Furthermore, calibration quality explained a substantial amount of individual differences for gaze, but not pupil size. We conclude that devices can be compared by correlating their outputs and that low-cost eye trackers may be perfect “pocket editions” of their “hardcover” siblings.

**What to expect from your remote eye-tracker when participants are unrestrained**

Diederick C. ^1,2^, Tim H. W. Cornelissen^3^, Kenneth Holmqvist^1,4^, Ignace T.C. Hooge^5^, Roy S. Hessels^5^

^1^Lund University, Lund, Sweden; 

^2^University of Muenster, Muenster, Germany; 

^3^Goethe University Frankfurt, Frankfurt, Germany; 

^4^North-West University (Vaal Triangle Campus), Vanderbijlpark, South Africa; ^5^Utrecht University, Utrecht, Netherlands diederick_c.niehorster@humlab.lu.se 

The marketing materials of remote eye-trackers suggest that data quality is invariant to the position and orientation of the participant as long as the eyes of the participant are within the eye-tracker’s headbox. As such, remote eye-trackers are marketed as allowing the reliable recording of gaze from participant groups that cannot be restrained, such as infants, schoolchildren and patients. Practical experience and previous research however tells us that eye-tracking data quality, e.g. accuracy and data loss of the recorded gaze position, deteriorates when the participant is unrestrained and assumes a non-optimal pose in front of the eye-tracker. How then can researchers working with unrestrained participants choose an eye-tracker? Here we investigated the performance of five popular remote eye-trackers from EyeTribe, SMI, SR Research and Tobii in a series of tasks where participants took on non-optimal poses. We report that the tested systems varied in the amount of data loss and systematic offsets observed during our tasks. The EyeLink and EyeTribe in particular had large problems. Furthermore, the Tobii eyetrackers reported data for two eyes when only one eye was visible to the eye-tracker. This study provides practical insight into how popular remote eye-trackers perform when recording from unrestrained participants. 

**Gaussian Mixture Models for Information Integration: **

**Toward Gaze-Informed Information Foraging Models for Imagery Analysis **

**Maximillian , Kristin Divis, Laura McNamara, J. Daniel Morrow, David Perkins **Sandia National Laboratories, United States of America mgchen@sandia.gov 

As eyetracking data moves from laboratory to naturalistic domains, researchers have the opportunity to develop rich ecological models of human-information interaction. Doing so, however, requires developing new data collection and analysis frameworks that facilitate reliable integration of eyetracking data with complementary indicators of human work behaviors, in the context of computersupported visual workflows. This paper describes exploratory work in applying Gaussian mixture models (GMMs) to the analysis of eyetracking data collected during a dynamic search task, in which participants were directed to look for specific features in a Synthetic Aperture Radar (SAR) image. Because SAR image products are geospatially registered, each pixel can be assigned a unique locational coordinate that reliably persists across subsequent SAR products collected at that location. We leveraging this property of SAR images to build a data collection and analysis system that enables the association of gaze events with content features in a SAR visual search workflow. Gaussian mixture models may provide an efficient way to associate gaze events with geospatial content in dynamic, user driven workflows. If we are successful, we envision developing gaze-informed foraging models to understand how imagery analysts become efficient in navigating and detecting key event signatures in large, noisy geospatial datasets.

**Moving from low level eye movement data to meaningful content in dynamic environments **

**Kristin M. Divis, Maximillian Chen, Laura McNamara, J. Dan Morrow, David Perkins **Sandia National Laboratories, United States of America kmdivis@sandia.gov 

Eye movement datasets provide a wealth of detailed information on user behavior; however, mapping that abstract eye movement data onto meaningful content in the display is a significant challenge in dynamic, user-controlled environments. How do we take low level data (e.g., velocity at an x, y coordinate on the black box of screen space) and map it onto meaningful content (e.g., she viewed the car)? For large workflows, hand-coding all areas of interest is not feasible. We are developing a set of tools to automatically map low level eye movement data onto meaningful content on the screen in dynamic, user-controlled settings. By utilizing the rich metadata in synthetic aperture radar (SAR) images and taking a systematic, controlled approach, we have created a dataset that allows us to build and validate these algorithms. Our work imposes probabilistic Gaussian distributions on the eye movement data, connecting them to activated superpixels in the SAR images, and then spatially and temporally grouping those activations to understand the meaningful sequence of steps each participant took. This work is the first step in a line of research designed to tackle the formidable challenge of interactive, dynamic environments in eye movement research. 

**Measuring dynamic and static vergence using an autostereoscopic display **

**Wolfgang Jaschinski**

Leibniz Research Centre for Working Environment and Human Factors, Germany jaschinski@ifado.de

Binocular eye movement research often requires different images presented to the two eyes for disparity-induced depth perception, for monocular calibrations or for using dichoptic nonius lines. Traditional instrumentation includes the mirror stereoscope and the separation of images with different filters. These optical parts near the eyes may hamper an unobstructed view of the stimulus. Autostereoscopic displays allow for a free-view image separation, but have disadvantages as lower resolution and residual cross-talk between the two eyes in larger visual fields. We tried to circumvent these limitations by using a low contrast and spatially limited stimuli on a 24’’ Tridelity SL2400 monitor at 70 cm viewing distance. Vergence step responses were induced by disparity offsets of 0.25, 1, 2, and 4 deg in the crossed and uncrossed direction. Vergence velocitiy of the 13 subjects was in the range of earlier studies and could be measured reliably (median r=0.8), even for step stimuli of only 0.25 deg. The objectively measured fixation disparity ranged between -30 to 60 minarc, was also reliable with (r=0.96) and agreed with the heterophoria, as measured with a Maddox rod procedure. Thus, when choosing appropriate stimulus conditions, an autostereocope can be used in vergence eye movement research.

**Objective measurement of variability of fixation disparity – is it possible? **

**Dawid Dominiak**^1^**, Alicja Brenk-Krakowska**^1,2^**, Wolfgang Jaschinski**^3^****

^1^Laboratory of Vision Science and Optometry, Faculty of Physics, Adam Mickiewicz University, Poznan, Poland; 

^2^Laboratory of Vision and Neuroscience, Nanobiomedical Centre, Adam Mickiewicz University, Poznan, Poland; 

^3^Leibniz Research Centre for Working Environment and Human Factors, Dortmund, Germany 

 dominiakidawid@gmail.com

The variability in fixation disparity (FD) is a clinically relevant indicator of binocular instability. The aim of present study was to identify a new method of objective measurement of instability of FD. Ten young adult volunteers participated in two sessions. The Eye Link II eye-tracking system was used at 20 and 40 cm viewing distance in three different test conditions: during observation two monocular nonius lines with and without a central fusion target, or while reading a paragraph. After eliminating artefacts from pupil fluctuations, a measure of FD variability was deduced, i.e. the inter-quartile range of the individual distribution of objective fixation disparity. These IQR-values were reliable between sessions with R2=0.81 at 20 cm. At 40 cm, the inter-individual range of IQR was smaller, so that IQR was less reliable, but larger in the group mean. Thus, the variability of FD differed between these viewing distances: on the average, smaller variability occurred surprisingly for closer distance. This is in contrast to previous studies of FD. However some recent studies showed that objective FD may behave differently than subjective FD as a function of viewing distance; thus, this may also refer to variability. 

**Sturmian-Wavelets as a tool to analyze eye tracking data**

**Jessica Adriana Del Punta**^1,2^**, Gustavo Gasaneo**^1^**, María Lujan Freije**^1^**, Marcos Meo**^1^**, Lorenzo Ugo Ancarani**^3^****

^1^Neufisur - Departamento de Física, Universidad Nacional del Sur - CONICET, 8000 Bahía Blanca, Argentina.; 

^2^Departamento de Matemática, Universidad Nacional del Sur, 8000 Bahía Blanca, Argentina; ^3^Equipe TMS, SRSMC, UMR CNRS 7565, Université de Lorraine, 57078 Metz, France 

jedelpunta@gmail.com 

Eye tracking a person who is performing a cognitive task allows one having an insight of how the brain works. However, the data provided by the eye-tracker contains many features of very different type that need to be identified and separated out. First of all there is the inevitable noise associated to the device itself. On the other hand, the saccadic and micro-saccadic movements add to the data characteristic shapes as overshoot and damping associated to the mechanical properties of the eye. In addition, the cognitive information is related to the way the eye performs the movements, the number of saccades, the way of going back and forth from one place to the other on the scene the person is watching. In this contribution we present a tool that we developed to extract the information based on the use of wavelets. We constructed a wavelet transformation in which the shape of the employed waves contains the physical information corresponding to the physical characteristics of the eye. This choice allows us to treat the system and to extract the cognitive information in an efficient way. 

Study on eye movement dynamics during the ‘jumping point’ experiment

Katarzyna , Pawel Kasprowski

Silesian University of Technology, Poland katarzyna.harezlak@polsl.pl 

The oculomotor system is one of the biological systems for which underlying characteristics are searched for intensively. It is a relatively simple task when state equations describing a system are known. However, if this is not the case knowledge of the system has to be built on the basis of observation of its properties. The aim of the presented research was to apply nonlinear systems analysis to eye movement signal examination. For this purpose, an experiment based on the wellknown ‘jumping point’ stimulus was utilized. A set of points was distributed over a screen. The points’ layout was designed in such a way to ensure both covering a screen area evenly and obtaining varying lengths of saccadic movements. The set of fixations related to different stimulus positions was defined for each experiment’s participant. These fixations were explored independently by means of methods for time series analysis. Time series were formed based on horizontal and vertical eye movement velocity and were analyzed in terms of dynamic patterns dependent on a stimulus position and eye movement direction. The studies presented that time series created for both movement directions provided similar representation of the underlying dynamics, however, varying for different saccade types. 

**An Update to the EYE-EEG Toolbox for Combined Eye-Tracking and EEG**

**Olaf Dimigen **

Humboldt Universität zu Berlin, Germany olaf.dimigen@hu-berlin.de 

Simultaneous recordings of eye movement and EEG data are useful in various research contexts. One major application is to time-lock the EEG to saccade or fixation onsets in unconstrained viewing situations (Dimigen et al, 2011, JEP:Gen). Other applications include fixation control, detection of EEG distortions from microsaccades, or enhancement of ocular artifact correction algorithms. Here I present an updated version of EYE-EEG (Dimigen & Reinacher, 2012, ECVP), an open-source MATLAB toolbox for the integration and analysis of combined ET/EEG recordings (www2.huberlin.de/eyetracking-eeg). New features include support for "Tobii" eye trackers, new options for data visualization, a cross-correlation-based method to assess the temporal alignment of the recordings, and an automated procedure to determine thresholds for the eye tracker-informed selection of ocular ICA components (see Plöchl et al., 2012, Dimigen et al., 2011). The poster will also present (1) synchronization accuracies for different combinations of ET (SMI/SR Research/Tobii) and EEG hardware (BrainProducts/EGI/Biosemi) and (2) an evaluation of the performance of the updated ICA procedure with different preprocessing pipelines using data from scene viewing and natural reading experiments. 

**Accuracy and precision test for a remote visible spectrum eye tracker **

**Chia-Ning Liao, Ming-Da Wu, Yen-Hua Chang, Wen-Chung Kao, Yi-Chin Chiu, Yao-Ting Sung **National Taiwan Normal University, Taiwan 

artning0905@gmail.com 

Most eye tracking systems need to operate with an infrared ray (IR) illuminant, although using IR might lead to discomfort and potentially damage the observers' eyes. Hence, a new generation of eye tracking system that operates using only visible light is highly anticipated. The aim of this study was to test a developed remote visible spectrum eye tracker's properties, e.g. accuracy and precision, and to investigate the effects of the dominant eye of observer, the color setting of foreground/background, and the stimulus point position on accuracy and precision. The design involved one between-subject factor: dominate eye (left, right), two within-subject factors: color setting (white point on black background, black point on white background) and stimulus point position (26 different positions). Twenty adults were recruited for the study. Their mean age was 28.5 years old. Eight observers were left-eye dominant, and the others were right-eye dominant. The overall result demonstrated that the mean accuracy and precision of the left dominant eye group were significantly better than the right dominant eye group. Meanwhile, point position also significantly affected the accuracy.

**Study on Directional Eye Movements in Non-frontal Face Images for Eyecontrolled Interaction **

**Min Lin **

Shanghai University of Medicine & Health Sciences, China 

 luc77sna@163.com

Directional eye movements based eye-controlled interaction focuses on interpreting the horizontal, vertical, diagonal eye movements or their combinations as inputs to design user interfaces for people who suffer with severe mobility disabilities. In this paper, we take into consideration the inherent eye jitter and evaluate the accuracy of dynamic tracking of horizontal, vertical, diagonal and rectangular eye movements prior to using them. We observe that the rectangular eye gesture composed of short horizontal and vertical eye movements has the best tracking accuracy in the presence of jitter. Finally, we present methods for identifying horizontal and vertical eye movements based on the trajectory of eye pupil centers from non-frontal face images. We find that the methods are robust and effective within ±20°deflective azimuths of non-frontal faces. This effectiveness is demonstrated by using the rectangular eye gesture as an interface to perform a painting task.

**Eye-movement in the dark for the exploration of virtual scenes encoded by sounds **

**Sylvain Huet**^1^**, Julien Doré**^2^**, Zélie Buquet**^2^**, Denis Pellerin**^1^**, Christian Graff**^2^****

^1^Univ. Grenoble Alpes, GIPSA-Lab, F-38000 Grenoble, France; 

^2^Univ. Grenoble Alpes, LPNC, F-38000 Grenoble, France

christian.graff@univ-grenoble-alpes.fr

Sensory consists in artificially replacing an impaired sensory channel by another, intact one. For instance, in its basic configuration, our Adaptive Visual Substitution system (AdViS) converts depth images issued from a head-mounted Kinect into a patterned sound. Substitute stimuli must find somehow their way up to associative cortical areas that usually do not process proprioceptive and acoustic data together. However colliculi and other primary brain structures that normally combine head and eye movements with retinal and cochlear input changes are largely left untapped. We improved the spatial perception through AdViS by including the visuo-motor system into it. A focal region within the converted visual scene is selected to prioritize information from the camera. The position of this focus within the scene is controlled by the user’s eye movements captured with an eye tracker. In their normal use, ocular saccades are directed towards salient parts of the visual field. We tested normal participants’ abilities to direct gaze movements in the dark, and to relate eye position with heterogeneities in a virtual scene through audio feedback. They succeeded in locating objects among four different places and in evaluating between two possible sizes.

**OT Eye: A tool to guide intervention and monitor progress during occupational therapy **

**Pieter Blignaut, Elize Janse van Rensburg, Marsha Oberholzer**University of the Free State, South Africa blignautpj@ufs.ac.za

Eye movements play a vital role in the development of functions such as visual form and space perception, reading, motor planning and eye-hand coordination. Occupational therapists assess clients’ eye movements as part of their routine assessment procedures. Fluidity and accuracy of eye movements give an indication of how well eye movements are coordinated, the sensory integration of vestibular and visual information and dissociation of the movements of the head and eyes among others. However, observations can be subjective and are hard to quantify in a reliable and valid manner if based solely on observations. A software tool, OTEye, was developed to track a client’s eye movements with good accuracy and precision. It is envisioned that this tool may enhance occupational therapists’ clinical observations of eye movements and give more accurate information that can be used to guide intervention and monitor progress. A valid and reliable assessment of eye movements would also enable further research into the factors that cause or contribute to poor eye movements, the effects thereof in clients’ daily lives, the efficacy of interventions targeting eye movements and related functions, and understanding how eye movements can influence other areas of functioning. 

**GazeCode: an open-source toolbox for mobile eye-tracking data analysis **

**Jeroen S. Benjamins, Roy S. Hessels, Ignace T.C. Hooge**

Utrecht University, Netherlands

j.s.benjamins@uu.nl

Software that comes with the mobile eye-tracker Tobii Pro Glasses 2 allows for manual classification of automatically detected fixations. Here, we present GazeCode and compare this in-house developed open-source alternative to the manufacturer software. Eye movements were measured during a 330 seconds card game amongst three players. One of the players wore a mobile eye tracker, the Tobii Pro Glasses 2. Experienced researchers subsequently categorized automatically detected fixations in the resulting dataset with both coding methods, Tobii and GazeCode. Inter-rater reliability was determined to be satisfactory for both methods (Cohen’s Kappa for coding fixation data with Tobii method: 0.92; for coding fixation data using GazeCode: 0.90). Moreover, average time to complete coding was shorter when using GazeCode (997.5 seconds) compared to coding fixation data using the Tobii method (2288 seconds). The longer time to code fixation data using the Tobii software could be due to the fact that as the number of already coded fixations increases, this software becomes slower and it becomes harder to keep track of already coded events, suggesting GazeCode to be a faster, more intuitive open-source alternative.

Session III - Wednesday, August 23^rd^, 15.30 - 17.00 

**Visual and ocolomotor control**

**Exploring the temporal dynamics of trans-saccadic perceptual re-calibration **

**Matteo Valsecchi**^1^**, Carlos R. Cassanello**^2^**, Arvid Herwig**^3^**, Martin Rolfs**^2^**, Karl R. Gegenfurtner**^1^

^1^Department of General Psychology, Justus-Liebig-Universität Giessen, Germany;

^2^Department of Psychology and Bernstein Center for Computational Neuroscience, Humboldt Universität zu Berlin, Germany; 

^3^Department of Psychology, Universität Bielefeld, Germany matteo.valsecchi@psychol.uni-giessen.de

Trans-saccadic re-calibration contributes to the perceptual uniformity of visual attributes, such as size, spatial frequency and shape, across the visual field. For example, if observers experience repeatedly that an object shrinks as they look towards it, in time they will report the same object as smaller when viewed peripherally. Here we introduced a novel paradigm where in each trial observers adjusted the size of a foveal disc to match the size of a peripheral (20°) one, which then they looked at. The transsaccadic size change was modulated as a sinusoidal function of trial number (1 cycle/100 trials, between 15% decrease and 15% increase). After linear detrending, we modelled the time series of adjusted sizes as a sinusoidal function of trial number, fixing the frequency of oscillation to 1/100 and leaving the time lag and amplitude as fit parameters. The average time shift for the 14 observers was 9.1 trials (±4.4 SD) and the average modulation was 2.98±1.7%, corresponding to a 20% gain relative to the physical modulation. These results show that perceptual trans-saccadic re-calibration rapidly tracks timevarying changes in the visual input. Its temporal dynamics are comparable to those observed for saccadic adaptation under similar conditions (Cassanello et al., 2016). 

**Selective of the luminance visual pathway by postsaccadic target blanking **

**Kazumichi Matsumiya**^1^**, Masayuki Sato**^2^**, Satoshi Shioiri**^1^

^1^Tohoku University, Japan; 

^2^University of Kitakyushu, Japan kmat@riec.tohoku.ac.jp

Previous studies have reported two phenomena affecting the perception of displacement during saccades: the blanking effect and landmark effect. In the blanking effect, temporarily blanking the target after a saccade improves displacement judgments. In the landmark effect, illusory target displacement occurs when a continuously presented landmark is displaced during a saccade and the target is temporarily blanked after the saccade without displacement. Since these effects involve a transient change in luminance after a saccade, this postsaccadic blanking may activate luminance transient-sensitive systems. We investigated the influence of luminance contrast on the blanking and landmark effects. In the blanking effect, target displacement detection rate increased with luminance contrast of the target. In the landmark effect, illusory target displacement decreased with luminance contrast of the target. The landmark effect was also found even for equiluminant color stimuli, while the blanking effect disappeared. Furthermore, the data analysis based on signal detection theory showed that both effects depend on a common process for target displacement detection, and that the landmark effect can be regarded as a bias in the decision criterion. These results suggest that changes in luminance, or transient signals, play a critical role in visual stability across saccades. 

**Transsaccadic prediction of real-world objects**

**Arvid Herwig**

Bielefeld , Germany aherwig@uni-bielefeld.de

With each saccade, internal object representations change their retinal position and spatial resolution. Recently, we suggested that the visual system deals with these saccade induced changes by predicting visual features across saccades (Herwig & Schneider, 2014, JEPG). In particular, peripheral perception should be biased toward previously associated foveal input. Up to now, effects of transsaccadic feature prediction on peripheral perception have been exclusively reported for simple visual features (e.g., spatial frequency, size and shape). The present study tested whether also complex visual features constituting real-world objects (fruits and balls) are predicted across saccades. Twenty-four participants in an eyetracking experiment first underwent a learning phase, where six out of twelve objects systematically changed their object category (from fruit to ball or vice versa) during saccades. In the following test phase, participants had to recognize briefly presented peripheral saccade target objects. Previously swapped objects were more often perceived as belonging to a different category compared to objects which did not change during acquisition. These category errors were mainly due to confusing the actual presented object with its previously associated foveal exemplar. This result indicates that transsaccadic prediction is object specific and not restricted to a small set of simple visual features.

**Visual perception of intrasaccadic information: A response priming experiment** **Charlotte Schwedes, Elodie Banse, Lorena Hell, Dirk Wentura**

Saarland University, Germany

c.schwedes@mx.uni-saarland.de

To keep stability of the visual impression during saccades, perception is reduced during saccade execution (saccadic suppression). However, only the magnocellular path of the visual stream is suppressed. To examine whether everyday stimuli (numbers 1 to 9) can nonetheless be perceived during a saccade, we implemented a response priming experiment. The prime number was presented during a voluntary saccade from a fixation cross to a predefined target location whereas the target appeared when the saccade reached the target location. Participants had to categorize the target as lower or greater than 5. In a subsequent direct test, the procedure was the same as in the priming task but the participants had to categorize the prime number while the target number was always set to 5. We found no priming effect (i.e. shorter reaction times in compatible compared to incompatible trials) but performance was above chance in the direct test. The results showed that everyday stimuli can be perceived during saccades. However, explaining the missing priming effect, attention allocation might be a relevant factor for intrasaccadic perception. In line with this, a follow-up experiment with the same prime presentation times but central prime and target presentation did show a priming effect.

**Visual working memory aids trans-saccadic integration**

**Emma E. M. Stewart, Alexander C. Schütz**

Philipps-Universität Marburg, Germany emma.e.m.stewart@gmail.com

With each saccade to an object, the visual system must reconcile the low-resolution pre-saccadic information with the high-resolution post-saccadic information. Humans achieve this trans-saccadic integration in a near-optimal manner, however it is unclear what mechanisms may underpin this process. This study aimed to determine whether visual working memory supports trans-saccadic integration, by introducing memory load during the saccade on a similar or a dissimilar feature. Participants completed a trans-saccadic integration task with colour or orientation. To measure integration performance, stimuli were presented in either peripheral or foveal vision, or both, and participants gave an angular judgment of perceived colour or orientation. In an additional memory task, a coloured or oriented memory item was shown at the beginning of the trial and memory was probed after the integration task was complete. We compared trans-saccadic integration performance with and without the memory tasks. For orientation integration, both colour and orientation memory load impaired integration performance compared to no memory task. For colour integration, only orientation memory load impaired integration performance. Memory performance was not affected by completing either integration task. This suggests that general memory resources are used to facilitate trans-saccadic integration, and these are not feature-specific.

**ERP potentials at the stage of saccadic preparation**

**Victoria Moiseeva**^1^**, Maria Slavutskaya**^2^**, Natalia Fonsova**^1,2^**, Valery Shulgovskiy**^2^

^1^National Research University Higher School of Economics, Centre for Cognition and Decision making, Russian Federation; 

^2^Lomonosov Moscow State University, biological faculty, Russia vikmoi@mail.ru

The oculomotor system is a good model to study competition between different elements in visual space. The goal of this study was to analyse spatial-temporal parameters of saccades and presaccadic ERP-potentials at the monocular presentation of the target and distracting stimuli. The study shows the dependence of saccade latency and ERP parameters from the conditions of stimulation. Saccadic latency depends on the brain hemisphere where the primary visual information of the stimulus projects. If it’s only one hemisphere as in the case of presentation of target and distracting stimuli in one visual semyfield, LP of correct saccades was shorter but in the same time the quantity of errors was bigger. But in the same time the LP were shorter when stimuli were presented to the dominant eye from the left side of visual field in comparison with undominant eye. Parameters and topography of the early ERP components N1 and P2 can reflect processes of visual sensory processing and attention processes at the same time. The potential N2 has complex nature and can show both the processes of motor preparation to the movement and inhibition of the distracter at the high level of subcortical structures activation.

**The effect of distractor processing on the target-related P300: Evidence from fixation-related potentials**

**Hannah Hiebel**^1^**, Joe Miller**^1^**, Clemens Brunner**^1^**, Andrey R. Nikolaev**^2^**, Margit Höfler**^1^**, Anja Ischebeck**^1^**, Christof Körner**^1^

^1^University of Graz, Austria; 

^2^University of Leuven, Belgium hannah.hiebel@uni-graz.at

Co-registration of electroencephalogram (EEG) and eye movements is a promising technique to study cognitive processes in overt visual search. Previous studies have shown that the detection of a target in visual search is associated with a P300. However, factors influencing this component in free viewing remain to be explored in more depth. We investigated the influence of preceding distractor fixations on the neural response to target detection. To this end, we recorded EEG and eye movements simultaneously while participants overtly performed visual search, presented with displays containing either one or two targets among distractors. Set size was varied systematically between 10, 22, and 30 items. Eye movement analysis showed that the average number of distractor fixations preceding the first target detection (fixation rank) increased as a function of set size. Fixation-related potentials (FRPs) revealed a larger target-related P300 amplitude for set sizes 22 and 30 than for set size 10, indicating a modulation of the P300 by fixation rank. Matching fixation durations and saccade amplitudes between conditions ruled out confounding effects of eye movements. Our results suggest that the number of inspected distractors defines the extent to which the target is perceived as a deviant, thereby modulating the P300 amplitude.

**Asymmetrical effects of saccade training on express saccade proportion in the nasal and temporal hemifields**

**Arni Kristjansson**^1^**, Jay Edelman**^2^**, Bjarki D. Sigurþorsson**^1^**, Ómar I. Johannesson**^1^

^1^Faculty of Psychology, University of Iceland; 

^2^Department of Biology, City College of New York, United States of America ak@hi.is

Recent evidence suggests that express saccade (ES) generation in humans increases with saccade training, and that this training benefit is independent of the actual saccade vector. Here we investigated whether such training-induced increases in ES proportion transfer between the nasal and temporal hemifields. Notable processing differences have been found between the two hemifields in attentional response and saccadic peak velocity, while any differences in saccade latency between the hemifields appear small. We trained 9 observers in making monocular saccades over 12 sessions of 280 trials. We found an asymmetric effect of training upon ES proportion. While before training ES proportion was overall low, it was slightly higher towards targets in the nasal hemifield. Following saccade training, however, this reversed, with a higher ES proportion towards temporal hemifield targets (in addition to the overall training induced ES increase). The express saccade proportion tripled for saccades to the temporal hemifield, but only doubled for saccades into the nasal hemifield. This raises the intriguing possibility that mechanisms responsible for saccade generation into the temporal hemifield are more amenable to training, and capable of faster saccades than they typically exhibit.

**Saccade training increases peak velocities and express saccade proportion for both trained and untrained eyes**

**Ómar I. **^1^**, Jay A. Edelman**^2^**, Bjarki D. Sigurþórsson**^1^**, Árni Krisjánsson**^1^

^1^University of Iceland, Iceland; ^2^The City College of New York, New York, NY, United States of America oij1@hi.is

Saccades are fast eye movements from one location to another in the visual field. The peak velocity (PV) of the saccades can be as high as 500°/sec and has a close relationship with their amplitude (Bahill et al. 1975). Peak velocities are higher towards the temporal, than the nasal visual field (Jóhannesson and Kristjánsson, 2013). Another parameter is saccadic latency and saccades have been classified by latency into express saccades (>70 – <130 ms) and regular saccades (>130 ms). Recent evidence suggests that training can increase express saccades generation (Bibi and Edelman, 2009). To test whether training effects carry over from the dominant to the non-dominant eye, we initially measured the latency and PVs of the saccades of 9 participants. We subsequently trained their dominant eye in 6 sessions of 280 trials, again measuring the latencies and peak velocities of both eyes, trained again for 6 session and compared the parameters again. Our results show for the first time that peak velocities increased with training and were higher into the temporal than the nasal hemifield. There was also a clear leftward shift of the latency distribution that transferred from the dominant to the non-dominant eye.

**Age-related changes in modulation of saccadic control by salience and value**

**Jing Huang**^1^**, Karl R. Gegenfurtner**^1^**, Alexander C. Schütz**^2^**, Jutta Billino**^1^

^1^Abteilung Psychologie, Justus-Liebig-Universität Gießen, Gießen, Germany; ^2^Allgemeine und Biologische Psychologie, Philipps-Universität Marburg, Marburg, Germany jing.huang@psychol.uni-giessen.de

Saccadic control has been shown to dynamically depend on salience and expected value. Whereas endpoints of short-latency saccades are determined mainly by target salience, endpoints of long-latency saccades reflect more pronounced top-down modulation (Schütz et al., 2012). Ageing could challenge this pattern by the well-documented increase of saccadic latencies as well as by changes in value processing. We explored the trade-off between salience and expected value in 21 young (22-39 years) and 19 senior (62-81 years) adults. Participants were required to make saccades to target patches with subregions differing in salience and associated reward. Dynamic modulation of saccade direction was found congruent with previous findings in both age groups. However, senior adults achieved overall lower scores than young adults (t(38)=2.36, p=.023). We determined a significant interaction effect between value manipulation and age group on saccade direction (F(2, 76)=4.56, p=.023). Expected value was less effective in senior adults. However, decreased modulation by value was associated with an agespecific modulation of saccade latency. Value manipulation triggered an average latency decrease in senior adults, but not in young adults (F(2, 76)=5.74, p=.005). We suggest that a facilitating effect of value expectation on latencies might counteract top-down modulation of saccade direction in senior adults.

**An age-dependent saccadic saliency model**

**Antoine Coutrot**^1^**, Olivier Le Meur**^2^

^1^University College London, United Kingdom;

^2^IRISA, University of Rennes, France

a.coutrot@ucl.ac.uk

The way we look at the world depends on who we are. For instance, an elderly Chinese woman might not look at a given image the same way as a young French man. However, most previous visual attention models do not take into account the profile of the observers. Here, we use the eye data from 101 observers split in 5 age groups (adults, 8-10 y.o., 6- 8 y.o., 4-6 y.o. and 2 y.o.) and evaluate 8 bottom-up saliency models from the literature with 7 different metrics. We show that depending on the metric, all models perform better with age, with two clear groups: adults and 6-10 yo on one side, 2 y.o. and 4-6 y.o. on the other. To take into account these age-related differences, we propose to use saccadic models, a flexible framework that can be tailored to emulate observer’s viewing patterns. We show that the joint distribution of saccade amplitude and orientation is a visual signature specific to each age group, and can be used to generate age-specific scanpaths. Our age-dependent saccadic model not only outputs human-like, age-specific visual scanpath, but also significantly outperforms other state-of-theart saliency models.

**Can the cortical magnification factor account for the latency increase in the remote distractor effect when the distractor is less eccentric than the target?**

**Soazig Casteau**^1^**, Françoise Vitu**^2^**, Robin Walker**^3^

^1^Durham University, United Kingdom; 

^2^Aix-Marseille Université, France; 

^3^Royal Holloway University of London, United Kingdom soazig.casteau@durham.ac.uk

The increase in saccade latency (SL) that is observed when a distractor appears simultaneously with a target is known as the Remote Distractor Effect (RDE). It has been found to depend mainly on the relative eccentricity of target and distractor stimuli rather than on their spatial separation, becoming smaller as the ratio of distractor-to-target eccentricity increases. One common account for this relationship is in terms of a competition between a fixation and a move system. However, this relationship could alternatively be explained by the cortical magnification factor (CMF), assuming that overrepresentation of stimuli closer to the fovea, in the visual cortex, enhances the negative influence of a distractor that is less eccentric than the target. To test this assumption, we used a distractor paradigm, and manipulated both the distractor-to-target-eccentricity ratio and the angular separation between target and distractor, but with the stimuli being either scaled according to the CMF or of comparable size. Results showed that the distractor systematically delayed saccade onset (RDE), with this effect decreasing as the distractor-to-target-eccentricity ratio increased. Yet, there was no difference between scaled and non-scaled stimuli, suggesting that previous findings cannot be accounted for by the CMF.

**The optokinetic nystagmus dynamic reflects the vection illusion perception**

**Artem Kovalev**

Lomonosov Moscow State University, Russian Federation artem.kovalev.msu@mail.ru

Vection describes the sensation of ego-motion induced by moving visual stimuli that cover a large part of the visual field. One of the main problems in vection research is to identify the vection perception periods using objective indicators. In the present study we used the eye tracking to examine parameters of optokinetic nystagmus (OKN) during vection perception. Vection was evoked by the rotating optokinetic drum with black and white stripes in CAVE virtual reality system. Rotational velocity was 20, 40 or 60 deg/s. 16 participants with healthy vestibular systems took part in this study. Subjects passively observed rotating stimulation and pressed the button to indicate the vection appearance. We analyzed durations of OKN slow phases in period of 10 seconds after pressing a button. It was found that for all stimuli velocities OKN slow phases were longer in these 10 seconds periods compared to other time periods of eye movement recording (F=44,5, p<0,01). It is suggested that the increase in OKN slow phases durations reveals the attention shift from moving stimuli perception to self-motion illusion perception. Therefore the OKN dynamics may be used as objective indicator for vection periods identification. This work was supported by grant RFH №17-36-01101.

**The use of eye tracking in fMRI study: differences in adults and children **

**predictive saccades**

**Katerina Lukasova**^1,2^**, Edson Amaro**^2^

^1^UFABC, Sao Paulo, Brazil; 

^2^NIF/LIM44, FMUSP, Sao Paulo,Brazil katerinaluka@gmail.com

Combined functional magnetic resonance imaging (fMRI) and eye-tracking measurements were performed in 21 adults (age 24 years, SD = 4) and 15 children (age 11 years, SD = 1). Subjects visually tracked a point target on a horizontal line in four conditions: time and position predictable task (PRED), position predictable (pPRED), time predictable (tPRED) and visually guided saccades (SAC). The eye tracking was done with MRI-compatible eye tracker (Mag Design and Engineering, sampling frequency 60Hz) and processed by ViewPoint software (Arrington Research, EUA). Both groups in the PRED task but not in pPRED, tPRED and SAC produced predictive saccades with latency below 80 ms. In task versus group comparisons, children’s showed less efficient learning compared to adults for predictive saccades (adults = 48%, children = 34%, p = 0.05). Group–task interaction was found in the supplementary eye field and visual cortex in the PRED task, and the frontal cortex including the right frontal eye field and left frontal pole, in the pPRED condition. These results indicate that, the basic visuomotor circuitry is present in both adults and children, but fine-tuning of the activation according to the task temporal and spatial demand mature late in child development.

**Microsaccade and blink rates index subjective states during audiobook listening**

**Elke B. Lange, Moniek Kuijpers**

Max-Planck-Institute for Empirical Aesthetics, Germany elke.lange@aesthetics.mpg.de

Microsaccade and blink rates as well as pupil dilation provide indices of cognitive processing unrelated to visual input. In the present study we investigate such cross-modal coupling between eye parameters and subjective states during listening to audiobooks. We selected excerpts of a wide range of authors (e.g., Tolkien, Goethe, Follett), literary genres (e.g., novels, fairy tales, mystery stories, drama, poetry, dada), and text-types (dialogue versus descriptions) from well-known books (e.g., bestsellers, published surveys), with a length of 37 to 60 sec. During audio presentation participants were asked to fixate a central fixational stimulus. After each excerpt participants rated their subjective states for absorption, imagery, felt valence, felt arousal, concentration level, being immersed in the sound of the voice or semantics, liking, and familiarity. Results were analyzed using linear mixed effect models. Reduced blink rate predicted absorption. Both reduced blink rate and increased microsaccade rate predicted imagery. No other models yielded significance, even though subjective ratings were highly correlated. This indicates highly specific relations between eye parameters and subjective states. The effect of increased microsaccade rate was unexpected, as decreased microsaccade rate is known to index cognitive load. Hence, an increased rate might relate to processing visual aspects of imagery.

**Fixation in EOG studies with eyes closed**

**Tanina Rolf, Niels Galley**

HerzNeuroZentrumBodensee, Switzerland taninarolf@yahoo.com

Aims: Fixation durations and blinks are expected to have less variation without a visible picture. Subjects usually perform eye movements with closed eyes. Voluntary saccades can be performed with eyes closed. Methods: Eye movements in 133 subjects with eyes closed were registered. They had to solve different tasks: to point to cracking noises; to count backwards; to find words beginning with “U”; to imagine their flat; acoustic running point; relax; interview. Results: We registered very many very short fixation durations (80-120ms). This seems to represent the searching behavior of human beings in the dark. There is a variation of fixation duration by different tasks with eyes closed. The following saccade may be delayed by mental stress. The longest fixation duration was measured in the acoustic running point, the shortest one in the relax task. Conclusions: Concentration might be measured in by fixation duration in each task. The subjects confirmed that the acoustic running point required the strongest concentration, the relax task required minor concentration.

**Separate resource pools for effector systems? Evidence from manualoculomotor dual tasks**

**Aleks Pieczykolan, Lynn Huestegge**

University of , Germany aleksandra.pieczykolan@uni-wuerzburg.de

Performing two tasks simultaneously usually yields dual-task costs, that is elevated response times compared to single-task performance. According to major dual-task frameworks, dual-task costs arise because limited processing resources need to be shared. Although these frameworks regard the oculomotor system rather as a provider of visual input than as an “ordinary” action modality, previous studies showed that executing oculomotor actions (i.e., saccades) exhibits resource sharing phenomena comparable to those in dual-task situations utilizing manual or vocal actions. Interestingly, only one dual-task framework explicitly focuses on the role of response modalities by assuming distinct resource pools, predicting that dual tasks involving different response modalities should be more efficient than tasks involving the same modality. In 2 experiments, we examined dual-task costs for the same manual action when accompanied by another manual action (intra-modal action) or by an oculomotor action (cross-modal action). While in Experiment 1, responses were spatially compatible to each other, they were incompatible in Experiment 2 to study effects of response congruence. Our results contradict the assumption of separate resource pools for response modalities by showing larger dual-task costs in cross-modal conditions irrespective of response congruence, likely due to additional cognitive demands resulting from response modality selection or activation.

**Influence of background illumination on horizontal and vertical objective fixation disparity**

**Remo , Joëlle Joss, Roland Joos**

FHNW Institute of optometry, Switzerland remo.poffa@fhnw.ch

We investigated the influence of background illumination on fixation disparity using two different tasks: 21 participants read two lines of text or fixated a sequence of dots (viewing distance: 60 cm). Text/dots were presented as bright targets on a dark background or as dark targets against a bright background, respectively. We further varied calibration, running all experiments with associated and dissociated calibration. Binocular eye movements were registered (SR Eyelink II) and horizontal and vertical fixation disparities calculated. Generally, for bright backgrounds fixation disparities became significantly more eso. When comparing reading against the dot fixation task, average horizontal fixation disparity changed significantly by 25 minarc, showing a crossed (eso) fixation for reading and an uncrossed fixation (exo) for dot fixations. No interaction was observed. As expected from previous research, vertical disparity did not change due to experimental conditions and variations in calibration did not affect fixation disparities. Uncrossed/exo fixation disparities for dark backgrounds in both tasks nicely relate to previous results and can be explained by lesser fusional demand and weaker stimulation of convergence. We further discuss weather more uncrossed/exo fixation disparities in dot fixations relate to target or presentation characteristics.

**Interactive and group eye tracking**

**Explore the effectiveness of online dynamic video-text vs. static image-text multimedia learning on students' science performance: An Eye movement study**

**Ya-Chi Lin, Hsiao-Ching She**

National Tung University, Taiwan hcshe@mail.nctu.edu.tw

This study investigated the effectiveness of online dynamic video-text vs. static image-text multimedia science learning on students’ performance of science concepts and scientific explanations. Forty students were randomly assigned into the dynamic video-text group who learned the concept through visualizing the dynamic videos of the events with text, whereas the static image-text group who learned the concept through visualizing the four critical static images of the events with text which were captured from the video of each event. Students’ eye movement behaviors were recorded during their learning with the use of EYELINK 1000. All students received pre-and post-scientific concepts and scientific explanation tests before and immediately after learning. The ANCOVA results showed that the dynamic video-text group’s students outperformed than to the static image-text group’s students, regardless of scientific concepts (F=13.17, p<0.001) and scientific explanations (F=11.30, p<0.002). Additionally, students in the dynamic video-text group allocated greater attention than to the static image-text group, regardless of the mean fixation duration and mean regression duration in the whole area, picture area, and picture-text area. This study demonstrated that dynamic video-text group’s students allocated greater attention than to the static image-text group’s students thus resulted in better performance of science learning. ****

**Using eye-tracking to provide dynamic assistance on the reading skills of beginner readers on desktop or mobile devices**

**Rykie Van der Westhuizen, Pieter Blignaut**

University of the Free State, South Africa blignautpj@ufs.ac.za

The ability to read fluently and with comprehension is closely related to eye movement behaviour of an individual. A multitude of commercial reading software applications exists and are developed with the aim to improve reading skills of beginner readers. In most of these applications, reading progress is measured subjectively through comprehension tests and an instructor’s observation. Accordingly, there is a need to address the lack of quantifiable metrics associated with a reader’s progress in existing reading applications, and eye-tracking provides a solution to this shortcoming. The immediate objective of this research was to develop an application for a desktop or mobile device that aims to i) provide tracked eye movement exercises to practice relevant eye movement skills, and ii) utilize existing techniques for providing guided reading. The system logs eye movement data and provide feedback on eye movement exercises and guided reading exercises. Ultimately, the logged data is used to dynamically adjust reading progress and highlight areas of difficulties experienced by the reader. The system can also recommend specific exercises based on the reader’s experienced difficulties. In conclusion, the application aims to improve reading skills of beginner readers by addressing the individual needs of each user.

**Using Tracking Data to Assist Identifying Wayfinding Strategies in the Virtual Maze**

**Tsuei-Ju Hsieh, Jun-Kai Niu**

Chinese Culture University, Taiwan tracy.tjhsieh@gmail.com

Three-dimensional virtual space is one of the essential elements for creating immersive experience of human-computer interaction. There is significant individual difference among users for finding way accurately and quickly in an interactive virtual space, such as a maze. This study aims at using eye movement data together with other wayfinding behaviors to reveal wayfinding strategies in the virtual maze. We designed six 3D mazes as stimuli which vary in three levels of spatial complexity and two types of navigation landmarks (feature in colors or shapes). The participants’ task was finding way out of the maze as soon as possible. During the wayfinding task, an eye tracker recorded each participant’s fixation times within areas of landmarks. Also, the time for finding way out, the number of times of hesitation, and the number of times of error were recorded as behavioral indicators for representing the wayfinding performance. Preliminary results show the landmark type affect participants’ wayfinding accuracy, efficiency, and navigation strategy, as they seemed to utilize those colored landmarks for distinguishing whether a path has been passed. However, as the complexity of maze increases, the participants tended to use try-error strategy.

**Real-time visualisation of student attention in a computer laboratory**

**Pieter Blignaut**

University of Free State, South Africa blignautpj@ufs.ac.za

A prototype system was developed to capture the gaze behaviour of students in a computer laboratory with 24 workstations. Each PC was equipped with a MyGaze eye tracker. Gaze behaviour on the PC as well as on the projection screen at the front of the room was captured on the individual student PCs and transmitted in real-time to the instructor PC. The instructor, having two displays, could duplicate one display with lecture content on the data projector while inspecting students’ attention on the other display. For the latter, the instructor could choose between any one of the student PCs or the projection screen as stimulus. The students’ real-time gaze behaviour could be overlaid as a bee swarm on the selected stimulus. While the technology exists and works, it is still unclear as to how to make the best use of it in real-time. Some modes of usage are better suited for use in a classroom than others. Arguments are offered in favour of usage during actual teaching and against. Challenges to be addresses by teachers and students as well as limitations with respect to the physical installation were identified.

**Detecting collaboration in a real classroom mathematics problem solving session from visual scan-paths**

**Enrique G. M.-Esteva, Jessica S.-Saari, Miika Toivanen, Markku S. Hannula**

University of Helsinki, Finland; enrique.garciamoreno-esteva@helsinki.fi

Our research question is to find out to what extent there is a common gazing strategy (as a measure of collaboration) during a real mathematics problem solving session. Our study involves three students in a live class wearing eye-tracking glasses during the students’ interaction. In our case study (the first of many such studies that we are conducting), we approach this problem by means of extracting longest common sequences (LCS) of fixations on areas of interest (AOI’s) from pairs of AOI fixation sequences obtained from the three students wearing mobile eye-tracking glasses. The LCS’s can be visualized using a dot plot approach similar to the one discussed by by Goldberg and Helfman (2010). Our technique allows us to compare the length of the extracted LCS with the lengths of thousands of LCS’s extracted from randomly generated pairs of AOI sequences and get some statistical validation on whether the students are in the same task mode (when the LCS length is longer than in the random experiments), moderately in the same task mode (when the length is close to the random average), or in totally different task modes during the activity (when the length is under the random average).

**Preservice teachers’ professional vision of own classroom management: combining mobile eye tracking in the classroom with retrospective reporting**

**Sharisse van Driel**^1^**, Halskza Jarodzka**^1,2^**, Frank Crasborn**^3^**, Saskia B.-Gruwel**^1^

^1^Open of the Netherlands, Netherlands; 

^2^Lund University, Sweden; 

^3^Fontys University of Applied Sciences Sittard, Netherlands sharisse.vandriel@ou.nl

Classrooms full of pupils are information-dense and dynamic real-world environments. Managing such classrooms is challenging for beginning teachers, yet crucial for pupils’ learning. Important for classroom management is teachers’ professional vision, including noticing and interpreting of relevant events. This study centred on the question: How do preservice teachers notice and interpret own classroom management events during teaching? 10 preservice teachers (3 female; 18-32 years old) conducted a 1hour lesson while wearing eye tracking glasses and participated in retrospective interviews afterwards based on these mobile eye tracking recordings. All gaze data recorded during teaching is manually transferred to a schematic coding of the classroom (via semantic gaze mapping) in which meaningful areas are represented, including pupils’ faces, body posture, feet or tables. These areas are based on prior exploratory research of teachers’ professional vision of classroom videos (Wolff et al., 2016). Moreover, eye tracking data will be also analyzed on fixation dispersion, transitions between pupils and revisits. Verbal data will be analyzed on interpretations based on a validated coding scheme (Wolff et al., 2015). The data analysis is in progress and results will be presented at the conference.

**“Look who's reading now!” - Evaluating the benefit of interactive eye tracking in chat**

**Christian Schlösser, Carsten Friedrich, Linda Cedli, Andrea Kienle**

FH Dortmund - University of Applied Sciences and Arts, Germany christian.schloesser@fh-dortmund.de

Coherence is a well-known property of comprehensible text. Chat – due to parallel text production and therefore missing turn-taking – is usually not able to provide such coherence and thus aggravates the interaction management with re-reading, revisions and withdrawals of messages. In a recent study, we explored the usage of interactive eye tracking to improve the awareness in chats and induce a more face-to-face like feeling, without altering the well-established chat functionality. In a counterbalanced within-subjects design, ten dyads used a standard and a gaze-enabled chat to discuss two given topics. Using a new reading detection algorithm that deals with the volatile patterns in chat, the following gazebased awareness features were implemented: (1) message seen, (2) reading message, (3) read message and (4) re-reading message. Additionally, highlighting of unseen messages and an accompanying notification was implemented. Early findings show that the gaze-enabled chat delivers a more “direct feeling”, motivates to “wait for the partner” and “helps to anticipate the course of the conversation”. However, the acceptance for using those features highly depends on context, role and topic. As this is research in progress, the analysis is still ongoing and further work will focus on acceptance factors and expansion to triads.

**Using eye-tracking techniques to explore students’ reading behaviors when using e-books with different role-playing mechanisms**

**Gloria Yi-Ming Kao**^1^**, Xin-Zhi Chiang**^1^**, Tom Foulsham**^2^

^1^Graduate Institute of Digital Learning and Education, National Taiwan University of Science and Technology, Taiwan; 

^2^Department of Psychology, University of Essex, United Kingdom gloriakao@gmail.com

Traditional e-books lack mechanisms to engage readers. In this study, we embedded role-playing in the reading activity to enhance students’ reading experience. Eye-tracking technology was used to analyze the students’ visual attention distributions during the reading process to understand the effects of roleplaying on their reading behaviors. Participants were 65 college students in Taiwan randomly assigned to one of three groups: Emotive Portraits, Fixed Portrait, or No Portrait. The result showed that all three groups spent more time fixated on the text since they still needed to read the text to know the storyline. Compared to the other two groups, the Emotive Portraits group fixated less on the text and more on the graphics and the main character, and had more saccades between the text and graphics. The Emotive Portraits group showed a higher reading motivation and higher reading engagement. These results indicate that the students were more interested in the self-portraits which could change emotions according to the development of the story. The emotive self-portraits helped the students engage in the role they were playing and enabled them to think about how to play the role better.

**What does simultaneous eye tracking of two people tell us about the social interaction and social presence of others? -A recurrence analysis-** **Seiya Kamiya, Haruka Nakamura, Takako Yoshida**

TokyoTech, Japan kamiya.s.ac@m.titech.ac.jp

It is known that eye movements have two roles, perceiving and signaling. For humans, this dual function makes the eyes a remarkable tool for social interaction. However, the data analysis methods of the eye movements for this type of social interaction have not been well-established yet. In this study, we proposed recurrence analysis to investigate this interaction. We applied this analysis, which has been proposed as a successful method to describe complex dynamic systems, and tested whether we could extract the synchronized gaze and blink behavior from the eye movement data obtained from the pair of participants looking at each other through a video chat-like display. Two conditions of video observation were tested: real-time face-to-face observation for both the participants (RT) and static face picture observation (SF) for one participant while the other participant observed this participant’s live face image. The results showed that the number of nearly synchronized blinks in RT condition was larger than the ones in the SF condition. Our results suggest that looking at a face is not only to see the face but also to have some eye behavior interaction.

**Teacher pair and group work in English as a foreign language lessons: insights from an eye-tracking study**

**Eva Minarikova**^1^**, Zuzana Smidekova**^2^**, Miroslav Janik**^1^

^1^Masaryk University, Institute for Research in School Education, Czech Republic; ^2^Masaryk University, HUME Lab, Czech Republic minarikova@ped.muni.cz

In the last two decades, considerable research has focused on what teachers find important when observing classroom situations. Many terms have been used (noticing, ability to notice, professional vision) and many research methods have been employed. Recently, teacher’s professional vision has been explored with the use of eye-tracking. Teachers’ gaze has been investigated e.g. when observing a classroom video or during instruction in simplified settings (Stürmer et al., 2017). In our research, we focus on teachers’ gaze in natural classroom settings. In this poster we address how a teacher distributes his gaze when monitoring pair and group work in English as a foreign language (EFL) lessons and present the results of a pilot study. Data was collected using SMI wireless eye-tracking glasses (60Hz) in three 7th grade EFL lessons of a male EFL teacher. As additional context data, we asked the teacher to comment selected sequences of the gaze replay to gain a deeper insight. The data is being analysed using BeGaze software focussing on the frequency and duration of fixations on selected areas of interest (students, instructional materials). The results will be available at the time of presentation and discussed together with introduction of the subsequent research.

**Detecting collaboration in a real classroom mathematics problem solving session from visual scan-paths**

**Enrique G. M.-Esteva, Jessica S.-Saari, Miika Toivanen, Markku S. Hannula**

University of , Finland; enrique.garciamoreno-esteva@helsinki.fi

Our research question is to find out to what extent there is a common gazing strategy (as a measure of collaboration) during a real mathematics problem solving session. Our study involves three students in a live class wearing eye-tracking glasses during the students’ interaction. In our case study (the first of many such studies that we are conducting), we approach this problem by means of extracting longest common sequences (LCS) of fixations on areas of interest (AOI’s) from pairs of AOI fixation sequences obtained from the three students wearing mobile eye-tracking glasses. The LCS’s can be visualized using a dot plot approach similar to the one discussed by by Goldberg and Helfman (2010). Our technique allows us to compare the length of the extracted LCS with the lengths of thousands of LCS’s extracted from randomly generated pairs of AOI sequences and get some statistical validation on whether the students are in the same task mode (when the LCS length is longer than in the random experiments), moderately in the same task mode (when the length is close to the random average), or in totally different task modes during the activity (when the length is under the random average).

**Facing challenges in groups – An exploratory eye tracking and EDA study**

**Michelle L. Nugteren**^2^**, Eetu Haataja**^3^**, Halszka Jarodzka**^1^**, Jonna Malmberg**^3^**, Sanna Järvelä**^3^

^1^Open of the Netherlands, Netherlands; 

^2^Department of Education and Pedagogy – Education, Faculty of Social and Behavioral Sciences, Utrecht University; 

^3^Learning and Educational Technology Research Unit, University of Oulu, Finland

Halszka.Jarodzka@OU.nl

During collaborative problem solving, challenges can interfere with the problem-solving process. It is not yet clear how students regulate and control these challenges. Therefore, this exploratory study investigated how people react to challenging situations when solving problems in groups. We used a mobile eye tracker to measure changes in the allocation of their visual attention and an Empatica E3 to measure their electro dermal activity (EDA). Twenty-four participants (Mage = 17.33, SD = 0.62 years, 11 females) worked in groups of 3 to 4 participants. Participants were asked to compose meal plans using available food packages, an electronic learning environment, and online search engines. In three groups, one participant wore a mobile eye tracker (SMI ETG 2w). Their gazes were coded with semantic gaze mapping to different AOIS (e.g., food package, tablet, other group members, etc.). Preliminary results show an interaction between type of situation (challenging vs. not) and AOI, indicating that participants allocated their attention differently when they faced a challenge. For instance, when participants faced a challenge, they looked less at the electronic tools and attend more to social interaction. Before the conference, additional analyses will be run to explore the EDA data. 

**Infrastructure Methodology for Group Studies in Multiple Eye Trackers Laboratory**

**Martin Konopka, Robert Moro, Peter Demcak, Patrik Hlavac, Jozef Tvarozek, Jakub Simko, Eduard Kuric, Pavol Navrat, Maria Bielikova**

Slovak University of Technology in Bratislava, Slovak Republic martin_konopka@stuba.sk

Laboratory with multiple eye trackers enables researchers to save time and effort with conducting experiments in parallel and opens possibilities for a novel research, such as observing a group of participants at once. Consequently, it requires us to tailor methodologies and software infrastructure used for recording and evaluating data. For our UX laboratory with 20 workstations, each equipped with Tobii X2-60 eye tracker (http://uxi.sk), we developed a software infrastructure to simplify process for everyone involved – conductors and participants. The infrastructure provides means of scheduling experiments, recording data on each workstation and collecting data on a single server for further processing or export. The only requirement put on a participant is to calibrate the eye tracker before an experiment. The infrastructure allows its integration with other, e.g., educational systems; it exposes eye tracking data in real time and collects custom events that occur based on the participant’s gaze or other actions. We designed a methodology for conducting experiments in the group setting, which is based on results of several group studies following this methodology focusing on the analysis of student behavior during an interactive lecture or researching individual differences between participants in usability studies.

**Robust Recording of Program Comprehension Studies with Eye Tracking for **

**Repeatable Analysis and Replay**

**Jozef Tvarozek, Martin Konopka, Jakub Hucko, Pavol Navrat, Maria Bielikova**

Slovak University of Technology in Bratislava, Slovak Republic jozef.tvarozek@stuba.sk

Source comprehension is a fundamental task of software development and its learning. Since source code is not just a free-flowing text, programmers apply various comprehension and reading strategies to understand the problem and its implemented solution, identify bugs and maintain the code. Eye tracking lets us study this process in a detailed manner, but since programmers modify and interact with source code in a code editor - making it a very dynamic stimulus spanned across numbers of documents of different sizes - we monitor fine-grained interactions in a code editor, so the programming session may be fully reconstructed afterwards, replayed and any data processing or analysis method reevaluated. We do so to avoid introduction of any possible bias by online preprocessing methods, e.g., incorrect online fixation filtering or mapping fixations to code elements, that could affect results of our method for automatic comprehension patterns approximate matching. In this poster, we present our methodology and results of program comprehension studies in more detailed and higher scale, when compared to existing works, with recording fine-grained interactions and raw gaze data in source code editors and our learning environment Turing, which is used in programming classes in our multiple eye trackers laboratory.

**Visual processing in the real world**

**Analyze the gaze behavior of drivers of semi-autonomous vehicles**

**Holger Schmidt, Rahel Milla**

Stuttgart Media University, Germany schmidtho@hdm-stuttgart.de

Actual transportation trucks are able to stay within the lane and to follow another vehicle autonomous. In the near future the portion of automation in these vehicle increase. Until trucks are able to drive fully automated, a driver is still needed to be able to regain control of the vehicle in difficult situations. In this context we conduct studies to analyze the drivers gaze behavior in semi-autonomous vehicles by using our stationary driving simulator.

Our goal is to develop a gaze based tool that measures the driver’s behavior in real-time an outputs the drivers "behavior state" based on gaze patterns. This enables further automation in order to improve the overall awareness of the driver and the security of the driving in real traffic with regard to the increase of automation.

As we stand at the beginning of the process we are about to conduct studies to measure the drivers gaze behavior in different automation setups and in manual driving situations in order to gain reaction times for different driving situations and gaze behavior patterns of the driver. We also aim toward the “automation trust” factor in our studies. The poster shows the planned process of research.

**Adding mirror information to the traditional Hazard perception test discriminates between novice and experienced drivers**

**Petya Ventsislavova, David Crundall**

Nottingham Trent University, United Kingdom petya.petrova@ntu.ac.uk

Driving is a visuomotor task which involves a division of attention, as the driver constantly explores the environment for potential hazards. The hazard detection process is studied by measuring driver’s fixations which has helped to identify several oculomotor strategies employed by drivers of differing experience. Inexperienced drivers have a narrower search pattern, accompanied by longer fixations while more experienced drivers tend to search a wider area of the visual scene. Without knowledge of where to look to spot potential hazards, novice drivers are less likely to employ the most appropriate visual strategies.

In order to study whether a mirror information would be better discriminator in terms of hazard detection, short driving video clips including front-view footage synchronised with the rear-view, right and left mirrors footage were recorded and edited into a hazard perception test. Sixty participants were eye tracked while watching each video clip. All video clips contained a variety of hazardous situations, including overtaking hazards which required the use of the added mirror information.

The contribution of the results to our current understanding of the hazard perception test and to models of eye movement behaviour will be discussed.

**Age-related changes in gaze dynamics during real-world navigation**

**Marcia Bécu, Guillaume Tatur, Annis-Rayan Bourefis, Luca L. Bologna, Denis Sheynikhovich, Angelo Arleo**

Sorbonne Universités, UPMC Univ Paris 06, INSERM, CNRS, Institut de la Vision, Paris, France marcia.becu@inserm.fr

Healthy aging is associated with changes in the way people navigate in space. However, little is known about the impact of aging on eye movements mediating the exploration and acquisition of spatial information. This study investigated how aging shapes the oculomotor dynamics during goal-oriented navigation in real environments.

Young old subjects solved a spatial navigation task by finding an invisible goal in a real environment (street-like setup made of realistic relief sceneries). After training, the configuration of landmarks was rotated, creating a conflict between the landmarks and the geometry of the environment.

During training, older subjects were longer to reach the goal, due to a larger decision time during reorientation and suboptimal trajectories. Gaze fixation characteristics (frequency, duration) as well as eyes/head coordination did not differ with age. However, in older adults fixations were significantly more exploitative (looking at the same landmark) than explorative (searching for a new landmark). Interestingly, compared to young subjects, a greater proportion of older adults reoriented according to a geometry-based strategy during probe tests, neglecting landmarks rotation.

In conclusion, analyzing the time course of eye movement signatures in real-world spatial tasks helped unveiling and understanding age-related differences in spatial coding and goal-oriented navigation strategies. 

**Potentials of eye-tracker use for wind turbine maintenance workers**

**Berna Ulutas**^1^**, Stefan Bracke**^2^

^1^Eskisehir Osmangazi University, Turkey; 

^2^Wuppertal University, Germany bernaulutas@gmail.com

Renewable sustainable energy sources are gaining more importance due to the increase in energy consumption. Among the renewable resources (i.e., solar, wind, ocean, hydropower, biomass, geothermal, biofuels, and hydrogen) that are utilized to generate electricity and heat; wind energy is known as the leading source of new capacity for several countries such as Europe, USA, and Canada. The wind power industry is rapidly developing based on better wind turbine aerodynamics, leaf blade designs and fault diagnosis technologies. On the other hand, a cost-efficient system depends on the reliability, availability, and longevity of the wind turbines. Therefore, planning and performing corrective and time-based maintenance are very critical in wind farms. There are several studies concerning maintenance, safety, and accident risks in the energy sector. However, limited number of studies focusing on the electrical and mechanical aspects of wind turbines are currently far from discussing human factors issues. This paper aims to attract attention to qualified technicians’ possible cognitive workload during performing maintenance and operation activities along with environmental ergonomic factors. To optimize maintenance schedules and reduce workplace fatalities, the possibility to utilize data obtained from mobile eye-tracker to identify and cluster the priorities and features of the duties are discussed.

**How individual differences in visual learning process are reflected by eye movements.**

**Aleksandra Kroll, Monika Mak**

Pomeranian Medical University, Poland kroll.at@gmail.com

Many cognitive functions are involved in the complex process of visual learning. Basing on the training task different functions are playing major role. To find eye-movements’ indicators of this differences we prepared two slightly different visual trainings based on a choice-reaction time task. The expected influence of visuospatial memory on the learning process was the differentiating factor between these two trainings.

Two groups of participants were asked to perform one of the trainings. We controlled the level of cognitive functions using Ruff figural fluency test (RFFT) which allows to measure the degree of visuospatial mode of fluency together with a test to measure the visual attention. Eye movements were recorded using an IROG eye-tracker.

The level of changes differed due to the type of visual task. In both groups the results differed also due to the level of executive functions measured by psychological tests. Also the preferred strategy of solving RFFT was differentiating subjects depending on learning achievements. Differences were also observed in eye movements’ patterns.

**The challenge of learning histology: a longitudinal observational study with medical freshman students.**

**Alan Brecht, Gertrud Klauer, Frank Nürnberger**

Goethe University Frankfurt, Germany alan.br@gmx.de

Learning histology is a challenging task: the student has to recognize characteristic visual features of the microscopic image and ascribe them to new cognitive categories, terms and biomedical concepts. This is an essential scaffold to further understand histopathological alterations, and thus we became interested in the learning steps of histology.

We ‘eye-tracking’ (Tobii RT120™) combined with ‘retrospective think aloud’ to analyze learning steps along six successive observation points of a cohort of 34 medical students. Items presented were digital images of microscopic course material used in classes. Think aloud protocols were categorized and statistically described and further analyzed with two-way variance analysis (ANOVA) with repeated measures and post hoc comparisons.

Participants acquire domain specific terminology very rapidly. But they are not successful in linking tissue features with terms and cognitive knowledge. Post hoc comparisons of fixation duration and numbers show no significant evidence for changes. While most fixations of participants are shown to be trapped on local salience spots, experienced students also observe relevant but less apparent structures. We recommend more time for training particularly when beginning to learn histology. Effective visual perceptive learning should be accompanied by rapid individual feedback.

**The decision making on radiologists: A joint effect of experience and authority** **Xuejun Bai, Meixiang Chen**

Tianjin Normal University, China baixuejun@mail.tjnu.edu.cn

Two types of factors have been hypothesized to affect radiologists’ decision-making: experience-based and authority-based. We tested both the hypotheses in a single experiment by using eye-movement technique. In experiment 1, we asked two groups of radiologists (expert and novice) to make a decision of detecting pulmonary nodule under three conditions which with different levels of authority clue (high, low vs. no). The result showed that the decision making of novice was highly affected by external clues irrespectively of which with high- or low- level of authority, while such effect on experts was only limited to high-level of authority cue. It appears that novice, with less experience, is more dependent on external clue (like authority) to make decision than expert. In experiment 2, we further investigated whether the consistency/inconsistency of the authority clue showed different effects on radiologists’ decision making. We found that the false negative rate was remarkably higher for novice than experts when their own decision making was inconsistent with the judgment from authority. We conclude that experience and authority play a joint role in radiologists’ decision-makings. The models in terms of expert-novice on decision-making will be discussed. 

**An Eye Gaze-Based Approach for Labeling Regions in Fundus Retinal Images**

**Nilima Kulkarni**^1^**, Amudha J.**^2^

^1^Dept of Computer Science & Engineering Amrita School of Engineering, Bengaluru Amrita Vishwa Vidyapeetham Amrita University, India; 

^2^Dept of Computer Science & Engineering School of Engineering, Bengaluru Amrita Vishwa 

Vidyapeetham Amrita University, India kulkarninilima@gmail.com

Objective: An image labeling is a tedious task. This becomes even more cumbersome when regions are to be annotated in the medical images. In this work, a novel method for identification/ labeling of the target (attractor’s regions or ROI) and distractor’s regions in fundus retinal images is proposed. Methods: The algorithm proposed here uses participant eye gaze fixation data for region labeling. The images used were fundus retinal images and target is an optic disc (OD). Here along with the target regions, the distractor’s regions were also identified. The eye gaze data is used which is collected from optometrists experts and non-experts group using the SMI eye tracker. The proposed algorithm identifies an attractor’s region with the expert fixations and distractor’s region with the non-expert’s data. The results are encouraging and the method provides a new edge for target and distractor labeling. Result: The optic disc detected by this algorithm with a 100% success rate for the fundus images taken from DRIVE and STARE datasets. The expert optometrist analyzed the results of the proposed algorithm and confirmed the validity and reliability of the method.

Novelty: The novel approach for labeling regions in fundus retinal image is proposed in this work. 

**No link between eye movements and reported eating behaviour in a non-clinical population**

**Frouke Hermens**^1^**, Leanne Caie**^2^

^1^University of Lincoln, United Kingdom; 

^2^University of Aberdeen, United Kingdom frouke.hermens@gmail.com

There is an increasing interest in the development of diagnostic tools to detect psychiatric disorders based on eye tracking. Studies comparing people with schizophrenia and controls have shown reliable differences in their eye movement patterns in various tasks. For treatment to be more effective, it may be desirable to detect people at risk rather than diagnose people at the clinical stage. To examine whether eye movements can aid in such risk detection, we examined the link between eye movements and reported eating behaviour (measured using the EAT-26 scale; experiments 1 and 2), schizotypy (O-LIFE scale, experiment 1), locus of control (experiment 2) and impulsivity (experiment 2). Eye movement tasks included smooth pursuit, fixation control and the anti-saccade task (experiment 1), free viewing of scenes with and without food items, low and high weight bodies, and a dot-probe task (experiment 2). Comparison of the eye movement metrics (e.g., the number of catch-up saccades, time spent looking at food, and pro- and anti-saccade reaction times and error rates) with the scores on the various scales did not reveal any significant correlations. These results suggest that the use of eye movements for diagnostics may be restricted to clinical populations only.

**Using Tracking to Evaluate Survey Questions**

**Cornelia E. Neuert**

GESIS - Leibniz Institute for the Social Sciences, Germany cornelia.neuert@gesis.org

Questions asked in surveys should produce data that are valid, reliable, and unbiased. A critical step to this end is designing questions that minimize the respondents’ burden by reducing the difficulty and cognitive effort required to comprehend and answer them.

Eye tracking appears to be a promising technique for identifying problematic survey questions. Eye tracking enables the researcher to see where and for how long respondents look when reading and answering survey questions. This feature can be used to detect questions that are difficult to understand or otherwise flawed (Galesic & Yan 2011).

By investigating their effectiveness in identifying difficult questions, this paper examines the potential of eye movements as indicators for evaluating survey questions. In particular, the presentation focuses on the following research question: Can eye-tracking be used to distinguish between easy and difficult questions?

In a laboratory experiment (N=100), eye-tracking was used to evaluate survey questions that had previously been tested in f2f-cognitive interviewing to classify them as either easy or difficult. The questions were compared by analyzing respondents’ attention processes and the cognitive effort respondents spend while answering the questions (operationalized by fixation times and fixation counts). Practical implications of the findings are discussed.

**Identifying in translation from scratch and post-editing with keylogging and eyetracking data**

**Jean Nitzke**

University of Mainz, Germany nitzke@uni-mainz.de

Translation processes often include problem-solving activity. When the translation of the source unit is not obvious to the translator on first sight (there is a barrier between the source item and the target item, cf. Dörner 1987), the translation process can be considered problematic. Using MT output for translation tasks should provide advantages in efficiency and reduce problem-solving effort in the postediting task (PE). However, if the quality of the MT output is not acceptable, new problematic translation units may arise.

24 translators (twelve professionals and twelve semi-professionals) produced translations from scratch, post-edited and monolingually post-edited MT output, which was produced by Google Translate. Altogether, the translators had to handle six texts (two texts per task). The translation and PE sessions were recorded with an eye-tracker (Tobii TX300) and a keylogging program (Translog II).

Earlier approaches to identify translation problems used think-aloud protocols and screen recordings (e.g. Krings 1986 or Kubiak 2009), which assessment is effortful and time consuming. Keylogging and eyetracking data were analysed for different problem indicators in the translation and PE tasks. Further, a method was developed to identify problems in translation research data with mere keylogging and eyetracking data. ****

**Evaluating the Comprehensibility of Graphical Business Process Models – An Eye **

**Tracking Study**

**Michael Zimoch**^1^**, Rüdiger Pryss**^1^**, Thomas Probst**^1^**, Winfried Schlee**^2^**, Georg Layher**^3^**, Heiko Neumann**^3^**, Manfred Reichert**^1^

^1^Institute of and Information Systems, Ulm University, Germany;

^2^Department of Psychiatry and Psychotherapy, Regensburg University, Germany; ^3^Institute of Neural Information Processing, Ulm University, Germany michael.zimoch@uni-ulm.de

Process models provide detailed information about tasks, decisions, and actors involved in various business processes. Graphical representations provide tangible benefits regarding process model comprehension compared to textual documentations. Many unresolved issues regarding the factors thwarting the understanding of process models, e.g., process model quality, exist. In this context, we use eye tracking to monitor selective attention shifts and serial groupings of semantically meaningful chunks in process model comprehension.

36 subjects (23 male) had to study 12 different process models expressed in BPMN, eGantt, EPC, and Petri Net by conducting a reading comprehension task. Further, subjects answered a questionnaire with questions related to the process described in the models. Subjects' scanning saccade patterns and relative fixation durations were recorded with SMI iView X Hi-Speed system at 240 Hz.

We observed specific eye-movement patterns (e.g., targeted search, back-and-forth saccade jumps) as well as unique strategies for reading different process model representations. Additionally, scan path pattern and fixation time variabilities indicate different levels of cognitive load and reveal potential stumbling blocks in the context of graphical business process model comprehension. The results, in turn, enrich the development of a conceptual framework, targeting at the comprehension of business process models. 

**Eye movements while perceiving images of natural and built environments**

**Jan Petružálek, Denis Šefara, Marek Franěk, Jiří Cabal**

University of Hradec Králové, Czech Republic jan.petruzalek@uhk.cz

The eye tracking methodology was used to compare eye movement behavior while viewing various types of outdoor scenes with their subjective perceived level of fascination and restorativeness in terms of the Attention Restoration Theory. Sixty undergraduates viewed thirteen ordinary urban scenes, thirteen old city scenes, and thirteen natural scenes. Eye-movements were recorded by means of the eye-tracker Tobii X2-60. The analysis of eye movements revealed that the mean number of fixations was the highest in ordinary urban scenes, the lower in old city scenes and the lowest in nature scenes. Significant differences in the mean number of fixations were between ordinary urban and natural scenes and between old city scenes and natural scenes. The mean fixation durations were the highest in nature scenes, the lower in old city scenes and the lowest in ordinary urban scenes. Significant differences in the mean number of fixations were between ordinary urban and natural scenes and between old city scenes and natural scenes. The study supports the recently discussed idea that nature scenes are processed easily than urban scenes. Moreover, the study also showed that perception of images of old scenic cities required lower activity of eye movements than perception of ordinary urban images. 

**Eye movements are linked to sexual preference in a real world preferential looking paradigm**

**Frouke Hermens, Oliver Baldry**

University of Lincoln, United Kingdom frouke.hermens@gmail.com

Forensic studies have examined whether eye movements can be used to measure a person’s sexual interest, for example, when treating sex offenders. Typically, participants are asked to look at images of naked models on a computer screen. Such images, however, reveal the purpose of the study, and may therefore be less effective in forensic settings. We here examine whether eye movements are linked to sexual preference in a real world preferential looking paradigm. Participants were informed that they were going to engage with other people in a separate room via a webcam link while wearing a mobile eye tracker. In fact, a video clip was played in which pairs of actors stepped in and out of view of a webcam, with each pair containing one female and one male actor. Overall, people’s tended to look at the leftmost actor first. When compensating for this leftward bias, we found that the person first fixated was significantly more often the actor of the participant’s reported preferred sex. These results suggest that sexual preference may be linked to real world viewing, but further work will be required to test the specificity and sensitivity of a measure of this viewing behaviour. 

**Language and Cognition **

**GAZE-SPEECH WHEN LISTENING TO L1 AND L2 SPEECH**

**Agnieszka Konopka**^1^**, Emily Lawrence**^1^**, Sara Spotorno**^1^

^1^University of Aberdeen, United Kingdom;

^2^University of Glasgow, United Kingdom agnieszka.konopka@abdn.ac.uk

How do listeners attend to native (L1) and non-native (L2) speech? Eye-tracked L1 and L2 listeners viewed naturalistic scenes and listened to English descriptions recorded by an L1 or L2 English speaker (n=16 per group) for a later memory test. The descriptions listed five of the eight objects present in the scene (e.g., “This is a baby’s room: there is a crib, a rocking horse, a toy train, an elephant, and a yoyo”, without mention of a doll, abacus, and picture in peripheral locations of the scene). At test, participants had to identify the studied scene among two alternatives (a studied scene and a modified scene that included a new object, substituted either for the 4th mentioned object or for an unmentioned object in the original scene). Analyses compared gaze shifts during study to assess the degree of L1 and L2 speechgaze coordination. All listeners showed stronger speech-gaze coordination when listening to L2 than L1 descriptions, but L2 listeners outperformed L1 listeners in memory for mentioned and unmentioned objects (both when listening to L1 and L2 descriptions). The results present new evidence of how online scene processing can vary as a function of listeners’ linguistic proficiency and the input language.

**When tones constrain segmental activation-competition in Chinese spoken word recognition: evidence from eye movements**

**Chung-I Su, Guan-Huei Li, Jie-Li Tsai**

National Chengchi University, Taiwan ericasuci@gmail.com

Eye fixations were tracked as listeners looked at a display of four printed words on a computer screen while following a spoken instruction to click on a target. The visual display comprised a target (e.g., 胎 /tai1/), a segmental competitor, and two phonologically unrelated distractors. The segmental competitor was either a cohort competitor with the same tone (e.g., 湯 /tang1/), a rhyme competitor with the same tone (e.g., 拍 /pai1/), a cohort competitor with a different tone (e.g., 唐 /tang2/), or a rhyme competitor with a different tone (e.g., 排 /pai2/). The results showed that words shared onset segments compete for recognition regardless whether they had the same or different tones, but the competition effects were larger and sustained longer for cohorts shared the same tone than those with different tones. In contrast, words shared offset segments compete for recognition only when they shared the same tone but not when they differed in tones. These results, not only replicated earlier/stronger competition effects for onset segmental overlap and later/weaker effects for rhymes (e.g., Allopenna, Magnuson, & Tanenhous, 1998), but also demonstrated that tones constrain segmental activation-competition in Chinese spoken words recognition. 

**Reading Music. How Tonality and Notation Influence Music Reading Experts' Eye Movements and Information Processing.**

**Lucas Lörch, Benedict Fehringer, Stefan Münzer**

Universität Mannheim, Germany lloerch@mail.uni-mannheim.de

Scholars in field of music psychology assume that regularities in musical notation regarding their visual display and tonal structures support the grouping of multiple notes into perceptual units (visual chunking), and the automatic activation of abstract musical information (cognitive chunking). In this study, the eye movements of music students were tracked while they performed a silent reading pattern-matching task with sequentially presented melodies. The melodies varied on two factors, tonality (tonal vs. atonal) and notation (regular vs. irregular). We analyzed differences in behavioural measures (reaction time, sensitivity, response bias) and eye tracking measures (number and duration of fixations, number and distance of saccades) between the different types of melodies. Both tonality and notation had a significant influence on reaction time and sensitivity. Eye movements were weakly influenced by the notation of the melodies, but not by tonality. We conclude that future studies should investigate the crucial aspects of both regularities and the exact conditions of the underlying mechanisms.

**Characteristics of sight-reading performance of pianists depending on texture of musical pieces** **Leonid V. Tereshchenko, Lyubov’ A. Boyko, Dar’ya K. Ivanchenko, Galina V. Zadneprovskaya, Alexander V. Latanov**

Lomonosov Moscow State University, Russian Federation lter@mail.ru

We studied eye movements in pianists at sight-reading of the musical text. The musicians of similar level of proficiency were asked to sight-read facing pages of two-line musical selections of three classical music pieces of various complexities. While sight-reading eye movements of musicians were recorded in conditions close to natural (with free head and torso motions).

We measured an eye-hand span (EHS) i.e. delay between the gaze position on a note and the performed music measured by musical signs number. The EHS varied significantly both in relation to each musician as well as between musicians from -3 to 14 symbols. We revealed a direct correlation between an EHS and the tempo stability and also a reliable invert correlation between an EHS and the number of errors at sight reading - an objective criterion of sight-reading ability. When performing the more difficult pieces the pianists demonstrated the shorter visual fixations separated by saccades of low amplitudes. While sight-reading the easiest musical piece the EHS is maximal and on average constitutes 4-5 symbols, for the most difficult piece – 2-3 symbols. Therefore, EHS is indicative of the difficulty of a music piece for sight-reading. (Supported by the Russian Foundation for Humanities №16-08-01082)

**Eye-movements during the encoding of object locations provide new insights into the processing and integration of spatial information**

**Anne-Kathrin Bestgen**^1^**, Dennis Edler**^1^**, Frank Dickmann**^1^**, Lars Kuchinke**^2^

^1^Ruhr-Universität Bochum, Germany; 

^2^IPU Berlin, Germany

Anne-Kathrin.Bestgen@rub.de

Maps provide a huge amount of spatial information, including spatial objects, the relations and distances between them. This variety of spatial information has to be perceived and filtered to build a mental representations of the environment, a so-called cognitive map. Map complexity and structuring map elements such as grids have different effects on cognitive maps formation. Yet, little is known about how spatial information is perceived and processed during the construction of a cognitive map, which may be the basis for these different effects. Eye-tracking data enable the examination of how different map information is processed during encoding, thus during the construction of a cognitive map. Moreover, a correlation of these eye-tracking data with results of a recognition memory paradigm can relate these measures to the actual spatial memory performance (d’; signal detection theory measure). Eye-tracking results reveal a lower number of fixations and longer fixation durations with increasing map complexity and in case of additional grid lines. Correlation analysis points to positive correlations between memory performance and fixation durations that are only observed in maps with higher map complexity. Additional heat-map analyses provide a more complex insight in the processing of spatial information from maps. 

**Automatic identification of cognitive processes in the context of spatial thinking** **Anna Klingauf, Benedict C.O.F. Fehringer**

University of Mannheim, Germany

aklingau@mail.uni-mannheim.de

Cognitive processes to solve spatial visualization tasks can be divided into three phases (search, transform, confirm, Just and Carpenter, 1976). Usually, these phases will be established by manual rating of eye movement fixations. The present study compares this rating with three algorithms for an automatic phase detection using the duration of the fixations (Dur), their positions (Pos), and both measures combined (PosDur) with a visualization test. In each task, participants have to decide whether two simultaneously presented Rubik’s Cubes are equal except for single rotated elements. Eye movements of the participants (N = 28) were recorded during performance of the test. The three algorithms were validated with respect to their distributions of the relative fixation durations and the relative numbers of saccades over all phases, as well as their relative deviation from the manual rating regarding the thresholds between the phases. Both expected distributions could only be found for the PosDur-Algorithm. The comparisons with the manual rating showed significant lower deviations for the Pos-, respectively PosDur-algorithm at both thresholds. Generally, the results show an advantage of the PosDur-algorithm. It is concluded that detection of cognitive phases in visualization tasks based on eye tracking measures can be computed automatically. 

**Rotate It! – What eye movements reveal about solution strategies of spatial problems**

**Stefanie Wetzel**^1,2^**, Veronika Krauß**^2^**, Sven Bertel**^1,2^

^1^Hochschule , Germany; 

^2^Bauhaus-Universität Weimar, Germany stefanie.wetzel@hs-flensburg.de

We report on results of a study in which university students solved mental and physical rotation tasks with three different levels of difficulty using our iPad app Rotate It!. The tasks follow the classical mental rotation test paradigm developed by Vandenberg and Kuse (1978). In the physical rotation condition, the 3D objects can be rotated using an Arcball interaction on the iPad. Times-on-task, answers given, touch events on the iPad, as well as users’ eye movements were captured during problem solving. In this contribution, we reflect on methods and methodology for the analysis of different time course data channels for the extraction of rotation strategies. Measures derived from eye movements, such as fixations rates and saccade amplitudes, were combined with measures derived from touch data, such as the angle or the direction of object rotations. Based on these measures, we derived several general rotation strategies. We found differences in the frequency of use of strategies between levels of task difficulty, as well as between individuals. Furthermore, the individual student’s pattern of strategy use can be a predictor for success and can indicate individual problem solver type.

**Fixation time as a predictor of the improvement of the test performance during a chronometric mental-rotation test**

**Martina Rahe, Claudia Quaiser-Pohl**

University of Koblenz-Landau, Germany rahe@uni-koblenz.de

Different strategies of solving mental-rotation tests could be a possible explanation for the gender differences in these tasks. To examine the influence of different strategies on mental-rotation performance we administered a computer based mental-rotation test to 60 females and 50 males (age: M=22.36; SD=2.45). Eye movements were recorded and the fixation-time spent on one of the cube figures before the participant switched to the other one was calculated. On the basis of fixation-times of the first half, the sample was divided into subjects with long and short fixation-times (median split). The improvements from the first half of the test on the second one were calculated for reaction time, rotational speed, and error rate. For reaction time and rotational speed, significant improvements and significant interactions of gender and fixation-time on the improvement were found. Females with initially long fixation-times had stronger improvements than males while females with short fixationtimes had fewer improvements. Overall, participants with shorter fixation-times reacted and rotated faster. Males seem to benefit more from shorter fixation-times while females with initially shorter fixation-times may be changing to less effective strategies. Or maybe faster switching females get worn out under stress more quickly during the test. 

**Eye movements during abductive reasoning process**

**Li-Yu Huang, Hsiao-Ching She**

National Chiao Tung University, Taiwan, ROC tlp.edu@gmail.com

The aims of this study were to explore the associations among the eye movement behaviors, task difficulties and task performance during the abductive reasoning tasks in different difficulties involving genetics. Fifty-five college students were recruited to participate in this study. They were asked to determine the following two tasks: whether the genetic disease is color blindness and whether the Xlinked disease is dominance or recessiveness according to the pedigree charts in 15s (task difficulty: task 2>task 1). Each task consisted of 50 trials.

The results showed the mean accuracy in task 1 was significantly higher than in task 2 (F(1,2749)=54.21, p<.001). The fixation number (F(1,5187)=11.77, p=.001), total fixation duration (F(1,5187) =11.78, p=.001) and re-reading number (F(1,5187)=3.90, p=.048) within the areas of interest (AOI) for the correct responses were significantly greater than those for the incorrect responses across task 1 and task 2. The mean re-reading time within the AOI for task 2 was greater than that for task 1 across the correct and incorrect responses (F(1,5187)=6.13, p=.013). To summary, this study demonstrated that subjects allocated greater attention in the high difficulty task than in the low difficulty task, and greater attention for the correct responses than for the incorrect responses.

**A tool to visualize the complete problem solving scenario**

**John J. H. Lin**^1^**, Sunny S. J. Lin **^1^National Central University, Taiwan;

^2^National Chiao Tung University, Taiwan john.jrhunglin@gmail.com

Eye tracking was integrated with hand writing to tackle the difficulties while solving problems. However, handwriting is difficult to be analyzed from a quantitative perspective. To qualitatively address the issue, a tool that visually represented the process was developed. This software is implemented in visual c++, which support for real-time data stream. In addition, Software Development Kit provided by main manufactory of eye trackers and a comprehensive GUI helped to efficiently access eye movement data. The tool could demonstrate three basic behaviors of handwriting, including writing, erasing, and moving. Records of eye movement and handwriting need to be imported, along with the corresponding timestamps. Given eye movement were recorded in a fixed frequency, whereas handwriting was varied. An algorithm was developed to chronologically synchronize coordinates of eye and handwriting. Afterwards, the process of problem-solving was showed and users could pause, move backwards or forwards to a specific moment. Dynamically presenting eye movement, as well as hand writing helped to get deeper insight into the reasons that led to difficulty. 

**The effects of symbolic and social cues on gaze behaviour.**

**Flora Ioannidou, Frouke Hermens, Timothy Hodgson** School of Psychology, University of Lincoln, United Kingdom ioannnidouflora@gmail.com

An important research question in social attention is whether social cues (i.e., cues provided by other people) are ‘special’ in that they elicit exogenous shifts of attention in the observer. Whereas the majority of studies have focused on cues presented in isolation and at fixation, more recent studies have started to examine the influence of social cues embedded in natural scenes. This latter work, however, has relied on small numbers of images, particularly when comparing social and symbolic (arrow) cues. As image features, such as saliency, can vary widely across images, results from these studies could be biased. to the particular set of images used. In this contribution, we will present the results of two experiments based on a much larger set of image. We directly compare the influence of gaze cues, pointing gestures, arrows and no-cue conditions, either when cues are presented alone inside a natural scene, or when placed in direct competition. To measure the influence of the cues, we measure observers’ eye movements and analyse these for the time spent looking at the cue, the cued object and the direction of saccades leaving the cue.

**Tonic and Phasic Changes in Pupil Size Are Associated with Different Aspects of Cognitive Control**

**Péter Pajkossy**^1,2^**, Ágnes Szőllősi**^1^**, Gyula Demeter**^1,2^**, Mihály Racsmány**^1,2^

^1^Hungarian of Sciences, Hungary; 

^2^Budapest University of Technology and Economics, Hungary ppajkossy@cogsci.bme.hu

There is accumulating evidence that the size of the pupil reflects activity of the brainstem nucleus Locus Coeruleus (LC), which innervates large parts of the cortex through noradrenergic (NA) projections (e.g. Joshi et al, 2016). This LC/NA system plays an important role in organizing information processing and behavioral regulation (Aston-Jones & Cohen, 2005). In a series of studies, we investigated how different aspects of NA transmission, reflected in pupil size, are related to attentional processes and controlled, effortful information processing. We assessed phasic noradrenergic activity by measuring task-evoked pupil dilation and tonic noradrenergic activity by measuring pretrial, baseline pupil dilation. We used several different cognitive tasks assessing attentional set shifting, verbal fluency, lexical decision. We found that tonic and phasic changes of pupil size are related to different aspects of cognitive control. These results support theories of the LC/NA system, which also suggest that tonic and phasic changes in NA level underlie different functions. 

**Pupil dilation and conflict processing: probability of occurrence of conflict trials influences pupil size**

**Michael A. Kursawe**^1^**, Franca Schwesinger**^2^**, Jochen Müsseler**^1^

^1^RWTH Aachen University, Germany; 

^2^Universität zu , Germany michael.kursawe@psych.rwth-aachen.de

In the literature it is debated whether spatial conflict effects are reflected by pupil dilations. We therefore examined, if conflict processing in the Simon task can become apparent by changes in pupil diameter. Analogous to increased reaction times and error rates we expected an increase in pupil diameter during incongruent trials compared to congruent trials. Additionally we aimed to show an increasing Simon effect when the probability of occurrence of a conflict trial is low. To test this hypothesis an eye tracking study was conducted with 39 participants doing a Simon task while measuring reaction times, errors and pupil dilation. To manipulate the probability of occurrence of conflict trials we varied the percentage of incongruent trials to either 80 or 20 percent. As expected we found a significant Simon effect in reaction times and error rates that increases with decreasing percentage of conflict trials. In addition we could show a Simon effect in pupil dilation in the condition with a low amount of incongruent trials. Considering these results it seems very likely that pupil diameter is able to reflect conflict processing in a Simon task especially when the occurrence of incongruent trials is not highly expected.

**Location Trumps Color: Determinants Of Free-Choice Eye Movement Control Towards Arbitrary Targets**

**Lynn Huestegge, Oliver Herbort, Nora Gosch, Wilfried Kunde, Aleks Pieczykolan**

Würzburg University, Germany lynn.huestegge@uni-wuerzburg.de

Models of movement control distinguish between different control levels, ranging from automatic 

(bottom-up, stimulus-driven selection) and automatized (based on well-learned routines) to voluntary (top-down, goal-driven selection, e.g., based on instructions). However, one type of voluntary control has yet only been examined in the manual, not in the oculomotor domain, namely free-choice selection among arbitrary targets, that is, targets that are of equal interest both from a bottom-up and top-down processing perspective. Here, we ask which features of targets (identity-related or location-related) are used to determine such oculomotor free-choice behavior. In two experiments, participants executed a saccade to one of four peripheral targets in three different choice conditions: free choice (unconstrained), constrained choice based on target identity (color), and constrained choice based on target location. A Bayesian analysis of choice frequencies revealed that free-choice selection closely resembled constrained choice based on target location. The results suggest that free-choice oculomotor control is mainly guided by spatial target characteristics. We explain these results by assuming that participants avoid less parsimonious re-coding of target-identity representations into spatial codes, the latter being a necessary prerequisite to configure oculomotor commands. 

**A cross-cultural investigation of the Positive Effect in Older and Younger Adults: An Eye movement study**

**Jingxin Wang**^1^**, Fang Xie**^1^**, Liyuan He**^1^**, Katie L. Meadmore**^2^**, Valerie Benson**^2^

^1^Academy of Psychology and Behavior,Tianjin Normal University,Tianjin China; ^2^School of Psychology, University of Southampton, United Kingdom wjxpsy@126.com

The 'Positive Effect' is defined as the phenomenon of preferential cognitive processing of positive affective information, and avoidance or dismissal of negative affective information in the social environment. Recentstudieshave investigated the ‘Positive Effect’ in different groups and cultures. There is evidence to suggest that older adults will develop a bias to focus and process positive emotional stimuli.However, there are very few studies investigating cross-cultural differences in emotion and aging in relation to the ‘Positive Effect’.To explore whether different cultures modulate emotional information in a similar pattern, in the current study we used eye tracking technology to investigate the ‘Positive Effect’ in English and Chinese participants when they looked at displays of pictures that included Pleasant, Neutral and Unpleasant pictures in the same display. The results suggested that both Chinese and English older and younger adults showed similar patterns of a ‘Positive Effect’ for emotional pictures. An interaction between emotion valence and culture was observed in all of the eye movement measures and the memory accuracy for pictures presented in the experiment.These interactions indicate that there are differences in inspection strategies between the two cultures, whichmay be underpinned by collectivist or individualistic cultural norms.

**Time-dependency of the SNARC effect on number words: Evidence from saccadic responses**

**Alexandra Pressigout, Agnès Charvillat, Karima Mersad, Alexandra Fayel, Karine Doré-Mazars**

Laboratoire Vision Action Cognition EA 7326, Institut de Psychologie, Institut Neurosciences et Cognition, Université Paris Descartes, Sorbonne Paris Cité, Boulogne-Billancourt, France alexandra.pressigout@gmail.com

A large number of studies have reported a robust SNARC effect (spatial-numerical association of response codes) on manual responses implying numerical quantity for Arabic digits and number words; this association is modulated by task demands for number words, but not for digits. Moreover, a recent study revealed an effector specificity : the SNARC strength does not correlate between manual and saccadic responses (Hesse, Fiehler & Bremmer, 2015). Based on saccadic responses, we compared the SNARC strength for Arabic digits and number words (from zero to nine). Twenty-eight participants made a parity judgment, a task assumed to activate numerical information automatically. Preliminary results show the expected SNARC effect for Arabic numbers (faster gaze durations to leftward/rightward responses with small/large numbers, respectively) but surprisingly, a different pattern was found for number words. Two distinct profiles emerged from individual differences: only half of our sample shows the classical SNARC effect that seems to correlate with response latency. Our results will be discussed in terms of time-dependency that only impacts the lexical representation of numbers (not their symbolic one). Based on saccadic latencies, the SNARC effect thus turns out to be less automatic than expected. 

**Empirical and Perceived Task Difficulty Predict Eye Movements during the **

**Reading of Mathematical Word Problems**

**Anselm R. Strohmaier, Matthias C. Lehner, Jana T. Beitlich, Kristina M. Reiss**

Technical University of Munich, Germany anselm.strohmaier@tum.de

Reading is the process of extracting textual information. We used methods from research on eye movements during silent reading to investigate the decoding of textual information in mathematical word problems. Based on the assumption that features of the text and features of the reader both influence eye movements during reading, we conducted two experiments. We tested if (a) on the tasklevel, the mean perceived task difficulty (PTD) and the empirical task difficulty (ETD) of a mathematical word problem predict eye movement parameters during reading and if (b) PTD and ETD can also predict reading parameters on the individual level. We found (a) a strong correlation between PTD and characteristic parameters of eye movements during reading. Experiment 2 showed that (b) on the individual level, PTD predicted these parameters similar to the ETD, but not better. On the betweensubjects level, significant variances for these effects suggest that the relationship between task difficulty and eye movements during reading largely differs between individuals. The results show that, in the context of mathematical word problems, features of the text and the reader influence eye movements during reading and that these eye movements might be an indicator for underlying cognitive processes.

**Cognitive strategies for solving graphically presented chemical tasks** **Yulia Ishmuratova, Irina Blinnikova**

Moscow State University, Russian Federation ishmuratova08@gmail.com

The purpose of our work was to identify differences in the way information is processed in visually presented graphical chemistry tasks in experts and novices. It was assumed that experts solve problems faster and make fewer errors, using fundamentally different cognitive strategies. Cognitive strategies were identified through the analysis of eye movement patterns. In total, 19 people took part in the experiment (7 of them were undergraduate chemistry students and 12 – specialists in chemistry). The study consisted of two stages. First, the subjects were asked to read a text describing a chemical process, then using information from the text they had to solve the problems in the form of graphs: Fill empty cells in the circuits; Indicate errors; Swap individual elements to maintain the correct structure of the chemical process. Perfomance time and eye movement characteristics were recorded with SMI iView X Hi-Speed equipment. Our hypotheses were confirmed. It was found that eye movements of the experts were characterized by longer fixation duration and shorter saccadic amplitude, which indicates deeper cognitive processing. Novices solve problems slower using less effective strategies, which is manifested in shorter fixations and high-amplitude saccades. Also the differences, when performing particular tasks, were figured out. 

**Reading: word-level processing**

**The availability of low spatial frequency information affects the effect of word predictability **

**Stefan Hawelka**^1^**, Tim Jordan**^2^

^1^University of Salzburg, Austria; 

^2^Zayed University, Dubai stefan.hawelka@sbg.ac.at

It has been hypothesized that during parafoveal preprocessing a reader mainly makes use of the low spatial frequencies (i.e., the coarse shape) of words. The present study investigated this assumption with a moving window paradigm. The text inside the moving window was presented normally. The parafoveal text (i.e., the text outside the window) was either unaltered or spatially filtered in such a way that it displayed the low spatial or the high spatial frequencies of the words. The stimulus material was the Potsdam corpus which provides predictability norms for each word of its sentences. The main finding was that the effect of word predictability was substantially reduced when the parafoveal information contained only the high spatial frequency information, but lacked low spatial frequency information. This findings indicate that the effect of predictability depends – at least partially – on the availability of low spatial frequency information about upcoming words.

**Cross-Frequency : Correlates of Predictability in Natural Reading**

**Nicole Alexandra Himmelstoß**^1^**, Sarah Schuster**^1^**, Lorenzo Vignali**^1^**, Stefan Hawelka**^1^**, Florian Hutzler**^1^**, Rosalyn Moran**^2^

^1^University of Salzburg, Austria; 

^2^University of Bristol, United Kingdom nicolealexandra.himmelstoss@sbg.ac.at

There is growing consensus that in line with predictive coding theories of perception, reading entails matching of linguistic input and predictions of upcoming words inferred by previous knowledge and context-based semantic information. A recent framework for language comprehension links predictive coding and oscillatory network dynamics gating hierarchical information processing. Within the language-network, it has been hypothesized that beta oscillations transmit ‘top-down’ predictions while gamma oscillations may indicate the ‘matching’ of predictions and input as well as ‘bottom-up’ driven prediction errors. We evaluated these hypotheses by manipulating both close probability and semantic congruency during natural reading by means of simultaneous eye-tracking and EEG. Employing dynamic causal modeling for induced responses we sought to identify effects of predictability on brain connectivity within the language-network. Applying DCM to source-reconstructed data from the inferior frontal gyrus and the ventral occipital-temporal cortex we found significant main effects of both global and local predictability on top-down cross-frequency coupling. Particularly, we observed an alteration in theta/alpha to gamma coupling from left inferior frontal to left occipito-temporal regions for low cloze and incongruent sentence finals. Our data provide evidence that reading networks adjust to the semantic predictability of sentences and to local mismatches using specific frequency-frequency interactions. 

**Predictability effects and preview processing for one- and two- character word in Chinese reading**

**Lei Cui**^1^**, Jue Wang**^1^**, Huizhong Zhao**^1^**, Simon Liversedge**^2^

^1^Shandong University, China; 

^2^University of Southampton, UK cuilei_cn@163.com

We report a boundary paradigm eye movement experiment to investigate whether the predictability that a character will be a word on its own, or the second character of a two character word affects how it is processed prior to direct fixation during reading. The invisible boundary was positioned prior to the one-character word or the second character of the two-character word. We also manipulated whether the target character was or was not predictable. The preview was either a pseudo-character or an identity preview. We obtained clear preview effects in all conditions, but also found a parafoveal-onfoveal effect. This effect only occurred when the target word was highly predictable from the preceding context. Moreover, the preview effects were larger when the target character was the second character of a two-character word than when it was a one-character word, indicating that preview processing of the second character of a two-character word was not only influenced by the preceding context but also by constraints deriving from the first character. We conclude that information about both a word’s constituent likelihood, as well as its likelihood based on preceding context is used on-line to moderate the extent to which upcoming characters are processed for meaning. 

Reading words in context: Effects of predictability in children’s and adults’ eye movements 

**Simon , Sascha Schroeder**

Max Planck Institute for Human Development, Germany tiffin-richards@mpib-berlin.mpg.de

We present a study of the effect of cloze predictability on beginning readers’ eye movements to investigate their use of context to generate candidates for upcoming words. The sample of 20 children, M(age) = 10.5 years, and 16 adults, M(age) = 24 years, read stories with embedded target nouns while their eye movements were recorded. Each story was presented with a title (e.g., “At the zoo”). Target words were either thematically related to the title (e.g., “animals”) or unrelated (e.g., “car”). Cloze predictability scores were collected from a separate sample of children and adults and used to validate the categorization of predictable (thematically related) and unpredictable (thematically unrelated) targets. Target words were further manipulated in their length and frequency. We present three main results. First, predictability was reliably manipulated by the relation of a word to the title theme of the story. Second, the predictability of words influenced readers’ eye movements independently of word length and frequency. Finally, the effect of predictability was greater for children than adults, suggesting that inexperienced readers use contextual information to facilitate their reading of continuous text. Results are discussed in relation to current models of eye movement control and reading development. 

**The two sides of prediction error in reading: on the relationship between eye **

**movements and the N400 in sentence processing**

**Franziska Kretzschmar**^1^**, Phillip M. Alday**^2,3^

^1^University of Cologne, Cologne, Germany; 

^2^University of South Australia, Adelaide, Australia; 

^3^MPI for Psycholinguistics, Nijmegen, Netherlands franziska.kretzschmar@uni-koeln.de

There is that domain-general principles like predictive coding explain sentence processing better than linguistic accounts. Instead of linking N400 and fixation measures via linguistic subdomains, the domain-general approach postulates that N400 and eye movements result from prediction error (Bornkessel-Schlesewsky et al. 2016; Friston et al. 2012). This predicts that both measures correlate only if they follow the same prediction error, without targeting a particular eye movement measure. To test this hypothesis, we re-analysed data from two eye-movement studies (N=116) and one ERP study (N=37) that investigated animacy-based prediction errors of actor prototypicality with identical stimuli. The experiments replicated previous results for unpredicted atypical actors by revealing larger N400 amplitudes, longer go-past time and nil effects for first-pass time. To assess whether reading times and N400 correlate, we aggregated one measure across participants to provide a numeric predictor for the other with mixed-effects models. Overall, the "design" models with experimental manipulations provided the best model fit. For the ERP data, aggregate go-past time provided a better fit than firstpass time. For the eyetracking data, aggregate N400 responses provided a better fit for go-past time than first-pass time. This supports the domain-general approach and emphasises the feasibility of crossmethods statistical modelling.

**Understanding word predictability using Natural Language Processing algorithms**

**Bruno Bianchi**^1^**, Gastón B. Monzón**^1^**, Diego F. Slezak**^1,2^**, Luciana Ferrer**^1^**, Juan Esteban Kamienkowski**^1,3^**, Diego E. Shalóm**^3,4^

^1^Laboratory of Aplied Artificial Intelligence (Computer Science Institute, CONICET-UBA, Argentina); 

^2^Computer Science Dept. (FCEyN-UBA, Argentina); 

^3^Physics Dept. (FCEyN-UBA, Argentina); ^4^IFIBA (CONICET-UBA, Argentina) brunobian@gmail.com

During reading our brain predicts upcoming words. Predictability (probability of guessing the next word) is currently estimated by performing cloze experiments, where participants read incomplete statements and have to complete them with one word. During the task, the only information subject can use is the preceding context. To estimate the predictability of one word, it is necessary to ask several participants, and then calculate the proportion of correct answers. Cloze-task is then an expensive experiment, and results are only valid for those words in the analyzed texts. During the last years, different approaches have been taken to automatically estimate this human predictability. Here we analyzed different ways of predicting words, using Natural Language Processing algorithms (LSA, word2vec, n-grams), and explore different aspects of the human predictability (semantic, syntactic, mnemonic). We evaluated the incorporation of these computational measures, both by themselves or combined on Linear Mixed Models with eye movements as dependent variables. Results show that these computational estimations of the word predictability have very good performance and can be used to replace the human predictability in the used models. Further, this is a step forward in understanding and separating the contribution of the different cues we use to predict words.

**Working memory capacity affects eye movement behavior during Chinese reading**

**Xingshan Li, Ya Lou**

Institute of Psychology, Chinese Academy of Sciences, China lixs@psych.ac.cn

In this study, we studied how the size of working memory capacity affects eye movement behavior during Chinese reading. Chinese reader with high working memory capacity and low working memory capacity read sentences including overlapping ambiguous strings while their eyes were monitored. In the consistent condition, disambiguating material compatible with the readers’ initial analysis of preceding material. In the inconsistent condition, the disambiguating material incompatible with the readers’ initial tendency of segmentation. For overlapping ambiguous stings, the middle character constitutes a word with the first character of the string, and it constitute another word with the third character of the string. Results showed that readers with high working memory capacity made shorter fixations and made longer saccades than readers with low working memory capacity. Interestingly, first fixation duration, last fixation duration, and outgoing saccade length in the overlapping ambiguous strings were all affected by the sentences condition only for the high-span individual. These results indicate that high-span individual can extract semantic information from parafoveal and such semantic information affect the decision where to fixate next. During the talk, we will discuss the possible reasons that working memory capacity affect eye movements. ****

**Reading and searching in Chinese: The role of lexical processing**

**Sarah J. White**^1^**, Xiaotong Wang**^2^**, Li Hua Zhang**^2^**, Xue Sui**^2^

^1^University of Leicester, United Kingdom; 

^2^Liaoning Normal University, China 

s.j.white@le.ac.uk

Eye movement behavior is compared during reading for comprehension and searching for a target word for Chinese text. The study provides an examination of whether lexical processing of words occurs during search for a target word, as well as reading for comprehension, in Chinese. The design was 2 (task: reading, searching) × 2 (critical word frequency: high frequency, low frequency). Participants completed two blocks of trials, a reading block and a searching block. Experimental sentences included a critical word (high or low frequency). There were also filler sentences within each block, each of which included the search target word. The experimental sentences never included the target word. For the experimental items, sentence reading times were longer than search times. For the critical words, there were significant effects of word frequency for reading for comprehension, but not searching. The results indicate that lexical access does not usually occur during search for a target word within Chinese text. These results are in line with those of Rayner and Fischer (1996) for reading and searching in English. Together the results indicate that search for a target word may be achieved by visual form matching regardless of the type of orthography.

**Orthographic and Root Frequency Effects in Arabic: Evidence from Eye **

**Movements and Lexical Decision **

**Ehab W. Hermena**^1^**, Simon P. Liversedge**^2^**, Sana Bouamama**^2^**, Denis Drieghe**^2^****

^1^Zayed University, Dubai, United Arab Emirates; 

^2^University of Southampton, Southampton, United Kingdom ehab.hermena@zu.ac.ae

One of the most studied effects in the reading literature is that of word frequency. Semitic words (e.g., in Arabic or Hebrew) contain roots that indicate the core meaning to which the word belongs. The effects of the frequency of these roots on reading as measured by eye movements is much less understood. In a series of experiments, we investigated and replicated word frequency effects in Arabic: Eye movement measures showed the expected facilitation for high- over low-frequency target words embedded in sentences (Experiment 1). The same was found in response time and accuracy in a lexical decision task (Experiment 3a). Using target words that were matched on overall orthographic frequency and other important variables, but that contained either high or low frequency roots, we found no significant influence of root frequency on eye movement measures during sentence reading (Experiment 2). Using the same target words in a lexical decision task (Experiment 3b), we did obtain a significant effect for root frequency, but it was qualified by a significant interaction with letter string lexicality. The results suggest that compared to overall word orthographic frequency, the frequency of Semitic roots has a more subtle, albeit important, influence on word processing measures.

**Information Acquisition from Left of the Current Fixation**：** Evidence from Chinese Reading. **

**Lin Li**^ 1^**, Xue Sui**^1^** & Ralph Radach**^2^

^1^Department of , Liaoning Normal University, Dalian, China; 

^2^General and Biological Psychology, University of Wuppertal, Germany

Prior research has shown that readers obtain information from locations left of the current fixation position (Binder, Pollatsek & Rayner, 1999; Inhoff & Radach, 2000). In a recent study, we addressed the issue using a postview paradigm with display changes implemented after a progressive saccade left the critical pre-target word (Radach, Reilly, Vorstius & Inhoff, 2015). Results indicated both an increased frequency of regressions and increased viewing times on the target word after orthographic and semantic changes were made on the pre-target. The present work used a similar methodology with Chinese sentences.

An invisible boundary was implemented within a well-matched attributive structure, so that an adjective was replaced either by a non-word mask (experiment 1: similar vs. dissimilar characters) or an alternative word (experiment 2: from high to low vs. from low to high frequency). 

When reader crossed the boundary, the adjective to the left was replaced. As a result, more regressions were made back to the pre-target adjective and viewing times on the target increased. This replicates our similar findings with sentence reading in German. We take this as solid evidence for the parallel acquisition of linguistic information from multiple locations with the perceptual span during reading in Chinese.

**Interword spacing effect on Chinese developmental dyslexia: A comparison in oral and silent sentence reading**

**Mingzhe , Ke Tan, Wen Wang, Xuejun Bai **

Tianjin Normal University, China bxuejun@126.com 

Chinese dyslexic children were far less efficient in phonologic processing and tone classification perception (Gao et al., 2016). Given the fact that oral reading is more dependent on phonological processing than silent reading, two experiments were conducted to explore whether interword space played more important role in oral than silent reading. In experiment 1, we recorded three groups of children’s eye movements when they read age-appropriated sentences: dyslexic children, age-matched group and reading level-matched group. Each sentence included four types of presentations: unspaced, word spaced, character spaced, and non-word spaced. In experiment 2, we adopted highlighting to create analogous conditions in order to keep the sentence length as the same in the four conditions. The results both in the two experiments showed that dyslexic children made shorter reading time in word spaced condition than that in normal unspaced condition when they read sentence aloud; however, this pattern did not occur in silent reading. These data indicates that the facilitatory effect of word spacing occurred in oral, but not in silent reading for dyslexic children. We argue that word spacing, as one kind of visual word segmentation cue, is helpful for Chinese dyslexic children in oral reading. (supported by NSFC:81471629).

**Transposed Letter Effects in Persian: Evidence from a Semantic Categorization Task**

**Ehab W. Hermena**^1^**, Hajar Aman-Key-Yekani**^1^**, Ascensión Pagán**^2^**, Mercedes Sheen**^1^**, Timothy R.**

**Jordan**^1^****

^1^Zayed University, United Arab Emirates; ^2^University of Oxford, UK

ehab.hermena@zu.ac.ae 

Persian belongs to the Indo-Iranian branch of Indo-European languages and is read from right to left. Using a multi-word display paradigm that resembled the procedure developed by Brysbaert (1995), we presented native Persian readers with three context-setting words (e.g., drink containers: پیاله cup, بطری bottle, پارچ pitcher). On the same line, a fourth (target) word (e.g., لیوان mug) appeared on the far left. Target words were presented either spelled correctly, or with transposed (لیاون) or substituted (لیلرن) internal letters. The context-setting words either belonged to the same semantic category as the target (the example above), or did not (e.g., clothes: دستکش gloves, شلوار pants, جوراب socks). All words were masked prior and subsequent to being fixated (e.g., once the reader’s eye moved on to the next word). Controlling the display and masking of all words was done using an eye tracker, with programed invisible boundaries triggering the display changes. The readers’ task was to determine if the target word is semantically related to the preceding three words. The results from response time, accuracy, and fixation duration measures, indicate that Persian readers show transposed letter effects, particularly when the target string is preceded by a semantically-congruent context.

**Word skipping in Chinese reading: The role of high-frequency preview and syntactic felicity **

**Chuanli Zang**^1^**, Hong Du**^1^**, Simon P. Liversedge**^2^****

^1^Tianjin Normal University, China; 

^2^University of Southampton, United Kingdom 

c.zang@soton.ac.uk 

Previous evidence demonstrates that Chinese readers skip an extremely high frequency parafoveal word de (的) automatically without taking the syntactic sentence context into account (Zang et al., 2017). An obvious follow-on question is whether the effects are limited to that word alone, or they are associated with a broader range of other high frequency words? In the present study, we manipulated target word frequency (high or low) and preview (identical, pseudocharacter, or syntactically infelicitous low or high frequency preview) using the boundary paradigm (Rayner, 1975). Results showed that for the high frequency target, skipping rates were higher for identical previews compared to the other two previews, however for the low frequency target, skipping rates were higher for high frequency previews compared to the other two previews. Furthermore, readers were likely to skip the target when they had a high frequency, syntactically felicitous preview compared to a high frequency, syntactically infelicitous preview. These effects were robust when readers’ eyes were launched from a near position, suggesting that decisions to skip a word in Chinese reading are based mainly on parafoveal word familiarity, but that syntactic felicity of the parafoveal word may also play a limited role. 

**Semantic Transparency Modulates the Emotional Words in Chinese Reading: **

**Evidence from Eye Movements**

**Kuo Zhang**^1^**, Jingxin Wang**^2^**, Lin Li**^2^**, Shasha Pan**^2^**, Simon Liversedge**^3^****

^1^Department of Social Psychology, Nankai University, Tianjin,China; 

^2^Academy of Psychology and Behavior, Tianjin Normal University, China; ^3^School of Psychology, University of Southampton, United Kingdom zhkuo@126.com 

Recently, studies have found the emotionality of a word influences lexical processes during both English and Chinese reading (Scott et al., 2012; Knickerbocker et al., 2014; Yan et al., 2015). However, to date it remains unclear as to how positive and negative words differentially influence eye movements during reading. Furthermore, semantic transparency is an important factor which affects eye movement behavior in Chinese reading, but it is still unknown whether transparency and emotionality interactively influence word identification in Chinese reading. In the present study, we manipulated emotionality and semantic transparency of two character target words to investigate how eye movements were affected during Chinese reading. Target words were matched for frequency and predictability across conditions, and embedded into the same sentence frames. Typical transparency effects and the interaction between emotionality and transparency were found on the measure of total reading time. In the transparent condition, the positive target words were read more quickly compared to the negative ones. For the opaque condition, however, the benefit for positive emotionality was not found. The results suggest that semantic transparency modulates the processing of emotional words during Chinese reading, which is consistent with the automatic vigilance model during processing emotion words. ****

**General Linear Model to isolate higher-level cognitive components from oculomotor factors in natural reading by using EEG and eye-tracking data coregistration **

**Anne Guérin-Dugué**^1^**, Benoît Lemaire**^2^**, Aline Frey**^3^****

^1^Laboratory GIPSA-lab, University Grenoble Alpes, CNRS; ^2^Laboratory LPNC, University Grenoble Alpes, CNRS; ^3^Laboratory CHArt, ESPE of the Créteil Academy, University of East-Paris Créteil anne.guerin@gipsa-lab.grenoble-inp.fr 

The Event-Fixation Related Potential (EFRP) technique allows free viewing conditions in ecological reading or visual scene exploration tasks. However, in those paradigms, saccadic potentials (i.e., presaccadic potential, “spike potential”, and sub-component of the “lambda response” at the saccade onset) overlap with the potentials elicited by fixations, producing confounding effects [Nikolaev et al., 2016]. Here, the confounded effects are isolated by regression using the General Linear Model with both fixation and saccade onset timestamps as predictors, whereas they are usually considered separately [Dandekar et al., 2012; Kristensen, et al., 2017]. Each fixation was tagged according to different reading strategies, relatively to the previous read words (forward progression with or without skipped words, refixation, rereading, and other situations). Moreover, each corresponding saccade was tagged relatively to its amplitude and orientation into six classes: five classes with an increasing amplitude in forward direction, and one class for the return sweeps (large amplitude in backward direction). To that end, the two sub-components of the “lambda response” have been distinguished. More generally, higher-level cognitive components of interest in reading (EFRP) can be separated from oculomotor factors thanks to the estimation of Event-Saccade Related potentials, in a same integrated model.****

**The use of pupillary response as an indicator of reading task complexity in Irish**

**Patrick M. Hynes**^1^**, Ronan G. Reilly**^1^**, Raúl Cabestrero**^2^****

^1^Maynooth University Department of Computer Science (Maynooth, Ireland); ^2^Department of Basic Psychology II. Faculty of Psychology. Universidad Nacional de Educación a

Distancia phynes@nuim.ie 

Twenty two native speakers of Irish participated in the study. Readers were presented with an Irish version of the English sentences used by Radach et al. (2008) in their study of top-down effects on reading. The stimuli comprised 80 sentences divided into two blocks of 40. The block order was counterbalanced across subjects. Comprehension questions targeted either simple (location, object) or complex semantic relations within the target sentence. Multiple-choice questions regarding which of four words had been in the sentence or passage just read were used to check comprehension. Our study investigated if the pupillary response during both tasks reflected reading complexity in a similar way to that reported by Radach et al (2008). The latter study found that fixation durations were significantly longer for the comprehension task as compared to the verification task. Therefore, our study aimed to investigate if there is pattern of pupillary response linked to those oculomotor variables and if there is temporal relation with them. Our findings are in line with the idea of the pupillary response being an indicator of cognitive load during reading with an overall increase in average pupil diameter for sentences read under the complex task condition.

**Dynamic properties of return sweep saccades during reading**

**Rostislav Belyaev**^1^**, Vladimir Kolesov**^1^**, Galina Menshikova**^2^**, Alexander Popov**^1^**, Victor Ryabenkov**^1^

^1^Lomonosov MSU, Moscow, Russian Federation;

^2^Institute of Radioengineering & Electronics RAS, Moscow, Russian Federation gmenshikova@gmail.com

The aim of our investigation was to analyze characteristics of return sweep saccades (RSS) which voluntary were executed on return sweeps from the end of one line to the beginning of the next during reading. 12 texts were constructed consisting of 60-64 words organizing in 6 lines, each subtended 15 degree of visual angle. 14 participants were tested. Their gaze positions during reading were recorded with SMI Hi-Speed technique. Saccade characteristics were analyzed including the distribution of saccade amplitudes, peak velocities and trajectory curvatures. The significant individual differences were revealed in all analyzed values. Using the RSS characteristics the dynamic model of the eye was developed which predicted approximate estimates of eye rotational dynamics. According to the model the equation was proposed linking the forces rotating the eye with the trajectory of eye movements. Our results may allow a better understanding of the eye dynamics in reading particularly in individual 

strategies of movements when performing RS saccades.

**Taking typography to experimental testing: On the influence of serifs, fonts and justification on eye movements in text reading **

**Julian Jarosch**^1^**, Matthias Schlesewsky**^2^**, Stephan Füssel**^1^**, Franziska Kretzschmar**^3 ^****

^1^Johannes Gutenberg-University Mainz; 

^2^University of South Australia; 

^3^University of Cologne jjarosch@students.uni-mainz.de 

Typography is assumed to extensively influence reading ease. Yet, some typographic variables such as serifs do not influence eye movements in reading (Perea 2013). One possible confound in previous studies may be the use of single sentences or short paragraphs, since many typographic variables only bring their influence to bear in longer texts. We tested this assumption using 12 short stories distributed on two pages, each with 24 lines, and varied font (Compatil vs. Lucida), serifs (serif vs. sans-serif), and justification (justified vs. flush left). Participants (N=32) read the stories for comprehension. Mixedmodels analysis replicated that serifs do not impact on fixation durations or saccades. Font did not influence reading. Justification, however, increased the number of fixations on a page, especially where spaces were extraordinarily wide. Moreover, readers tended to fixate on spaces more often with increasing space width, while fixation duration decreased in such cases. This suggests that readers used these fixations to plan saccades to upcoming words that, otherwise, fell outside of parafoveal preview. Overall, our findings suggest that typographic variables influence eye movements when they have a direct influence on word identification (justification). Whenever they do not impede word identification, they do not influence reading ease (serifs).

**Translation assessment: eye movement evidence **

**Alena Konina, Tatiana Chernigovskaya **

Saint Petersburg State University, Russian Federation alena.konina@gmail.com 

Translation theory focuses mainly on the translation product and translation process, less on the translator, and even more rarely on the figure of the translation quality expert. This study makes an attempt to identify their role in the translation theory paradigm through eye movement research and identify main assessment strategies by comparing intuitive and scale-assisted assessment methods. In the first experiment, participants were asked to intuitively assess student translations on the scale from one to five (Cronbach’s alpha=0,88). We were able to conclude that experts read the source text longer than the translation (F=85,842, p<0,001) compared to professional translators (Jakobsen&Jensen, 2008), following the same pattern as student translators. They also make longer fixations (F=21,334, p<0,001) than participants with simple reading task. In the second experiment, another group of experts was asked to assess the same student translations via a modified error typology (MeLLANGE). The group followed two strategies: “strict” and “mellow” ones showing little consistency and no statistically significant correlation with their experience. These results allow us to conclude that TQA experts follow a different reading pattern than native speakers and translators and become much less consistent when asked to decompose their skill transitioning from intuitive to error typology assessment. 

**What does the rhino do with the rose? Predicting gaze duration to validate an adult version of the Salzburger Lese-Screening (SLS-B) **

**Jana Lüdtke, Eva Fröhlich, Arthur M. Jacobs **

Freie Universität Berlin, Germany jana.luedtke@fu-berlin.de 

The Lese-Screening for adults (SLS) is a standardized sentence reading test which had been developed to ecologically assess reading fluency in normal adult readers for scientific research. Here we report results of a validation study of a computerized version of the SLS (SLS-B). For this purpose, 34 adults (M = 28.2 years) completed the SLS-B, named a list of words and pseudowords (SLRT-II; Moll & Landerl, 2010) and read silently two texts with varying levels of complexity for which we obtained eye tracking data (EyeLink 1000; SR Research). To assess the validity of the SLS-B we regressed its test scores, as well as those of pseudoword reading (PWR) on gaze durations of texts 1 and 2, respectively. Comparing the corrected coefficients of determination (r2) showed PWR to explain 24.8 and 27.9% of variance (text 1/2), while the SLS-B explained 29.7 and 25.9%. However, for participants with an above average reading time, r2 of PWR dropped to .17 and .20 while the SLS-B accounted for 49.6 and 35.5% of the variance. The results led us to conclude, that the SLS-B is particularly suited for differentiating within the lower range of reading fluency. 

## Author Index

**A **

Aagten-Murphy, David 160 

Ablinger, Irene 247, 248 

Abrahamson, Dor 265 

Acarturk, Cengiz 103 

Achtziger, 167 

Adiatullin, Adel 214 

Adini, Yael 121 

Aeschbach, Daniel 102, 261 

Agtzidis, Ioannis 90, 233 

Ahmed, Zaheer 116 

Ahn, Jieun 140 

Aizenman, Avi M. 149 

Akehurst, Lucy 165 

Akhutina, Tatyana 207 

Al , Noor Z. 69 

Alahyane, Nadia 

Alday, Phillip M. 311 

Alexa, Marc 116 

Alexander, David 124 

Alexeeva, Svetlana 83, 142 

AlJassmi, Maryam A. 84, 155 

Aluani, Fernando O. 79 

Aman-Key-Yekani, Hajar 315 

Amaro, Edson 284,315 

Ancarani, U. 273 

Andersson, Richard 149 

Andrews, Sally 117 

Angelaki, Dora 111 

Angele, Bernhard 83, 144 

Anisimov, Victor 184, 214, 244, 256 

Ansorge, Ulrich 224, 260 

Aponte, Eduardo A. 66, 159 

Archibald, Neil 112 

Arkesteijn, Kiki 79 

Arleo, 189, 294 

Arne, Dekker 216 

Asthana, Manish K. 226 

Atkins, Andrew 253 

**B **

Baccino, Thierry 144, 211 

Backhaus, Daniel 240 

Bagdasaryan, Kristine 142 

Bailey, Reynold 269 

Bailly, 92 

Bakker, Arthur 265 

Baldry, Oliver 300 

Ballenghein, Ugo 144 

Banse, Elodie 279 

Bao, Jun 247, 268 

Barsingerhorn, Annemiek D. 117 

Bartolozzi, Chiara 150 

Batten, Jonathan P. 73, 108 

Bauch, Sebastian A. 236 

Bays, M. 160 

Becker, Stefanie 235 

Bécu, Marcia 189, 294 

Bednarik, Roman 219 

Beelders, Tanya 179 

Behne, Dawn M. 251 

Beitlich, Jana T. 308 

Belan, Ariella F. 244 

Belopolsky, Artem V. 77, 79, 123 

Belyaev, Rostislav 318 

Bendixen, 185 

Benedek, Mathias 186 

Benedetto, Simone 152 

Benjamins, Jeroen S. 277 

Ben-Shakhar, Gershon 113 

Benson, Valerie 175, 307 

Berger, Corinne 246 

Bertel, Sven 303 

Bertram, Raymond 198 

Bestgen, Anne-Kathrin 302 

Beynel, 245 

Bhandari, Pratik 114 

Bianchi, Bruno 312 

Biele, Cezary 121, 216 

Bielecki, Maksymilian 103 

Bielikova, Maria 292, 293 Biemann, Chris 118 

Billino, Jutta 282 

Biscaldi, Monica 222, 237, 259 

Bisley, James 58 

Blignaut, Pieter 126, 276, 287, 288 

Blinnikova, 229, 309 

Blythe, Hazel I. 71, 119, 157 

Boampong, Derrick 249 

Boccignone, Giuseppe 222, 237, 259 

Böckler, Anne 223 

Bodenschatz, Charlott M. 226 

Böhmert, Christoph 212 

Bologna, Luca L. 294 

Bompas, Aline 125, 161, 189 

Bonmassar, Claudia 222 

Bonneh, S. 121 Boon, Paul J. 123 

Boonstra, Nienke 117 

Boot, Walter R. 238 

Borji, Ali 173 

Bornkessel-Schlesewsky, Ina 200 

Bouamama, Sana 313 

Bourefis, Annis-Rayan 294 

Boven, Loes 265 

Boyko, Lyubov’ A. 302 

Bracke, Stefan 295 

Bradford, E. F. 166 

Brandt, Silke 140 

Braw, Yoram 246 

Brecht, Alan 296 

Bremmer, Frank 110, 190 

Brenk-Krakowska, Alicja 273 

Brien, Donald C. 69 

Brocher, Andreas 136, 188 

Bruder, Carmen 115 

Brunner, Clemens 280 

Brunsdon, E. A. 166 

Buonocore, Antimo 234 

Buquet, Zélie 276 

Burke, Melanie R. 64 

Burke, Michael 98 

Büsel, Christian 260 

Busin, Yuri 226 

**C **

Cabal, Jiří 299 

Cabestrero, 318 

Caie, Leanne 297 

Cajar, Anke 239 

Caldato, Christian 152 

Cambuzat, Remi 92 

Campbell, Annie 161 

Carrasco, Marisa 54 

Caspi, Avi 177 

Cassanello, Carlos R. 277 

Casteau, Soazig 112, 283 

Catrysse, 128 

Cedli, Linda 289 

Chandra, Johan 155, 192 

Chang, Chia Yueh 229 

Chang, Yen-Hua 275 

Charvillat, Agnès 308 

Chau-Morris, Ashley 176 

Chauvin, Alan 233, 245 

Chen, Jing 231, 232 

Chen, Maximillian 271, 272 

Chen, 296 

Chernigovskaya, Tatiana 319 

Chiang, Xin-Zhi 290 

Chicca, Elisabetta 150 

Chiu, Yi-Chin 275 

Chohen, Tzur 246 

Choi, Soonja 224 

Chrząstowski-Wachtel, Dominik 216 

Chukoskie, Leanne 68, 250 

Chumachenko, Dmitry V. 187, 188, 204 

Churan, 110 

Clackson, Kaili 63 

Clarke, Alasdair D.F. 100 

Clarke, Sophie 201 

Clausel, Marianne 145 

Clayden, Adam C. 106 

Clemens, Ivar 109 

Clifford, William D. 259 

Cohen, Noga 138 

Cornelissen, Frans W. 249 

Cornelissen, H. W. 90, 97, 146, 271 

Coutrot, Antoine 178, 282 

Crasborn, Frank 289 

Crawford, Trevor J. 80, 243 

Crundall, David 164, 165, 294 

Crutch, Sebastian 249 

Cui, Lei 310 

Curran, Tim 108 

**D **

Dalmaijer, S. 93 Damsma, Atser 73 de Dieuleveult, Alix 189 Deegan, Catherine 259 

Del Punta, Jessica A. 273 

Delrioux, Claudio A. 266 

Demcak, Peter 292 

Demeter, Gyula 306 

Deubel, Heiner 78, 111, 113, 163, 185 

Devillez, Hélène 108 

Diaz, Gabriel J. 269 

Diaz-Tula, Antonio 79, 219 

Dickmann, Frank 302 

Dienes, 140 

Dimigen, Olaf 274 

Dini, Amir 92 

Dirkx, Kim 260 

Divis, Kristin M. 152, 180, 271, 271 

Dobrego, Aleksandra 83 

Domagalik, Aleksandra 64 

Dominiak, Dawid 273 

Donche, Vincent 128 

Donk, Mieke 79 

Donnelly, 263 

Donovan, Tim 201 

Doré, Julien 276 

Doré-Mazars, Karine 161, 203, 308 

Dorn, Jessy D. 177 (fert Dorr, Michael 88, 90, 107, 233, 241 dos Santos Rodrigues, 139 

dos Santos Wisintainer, Danielle 255 dos Santos, Bernardo 57 Dowiasch, Stefan 190 

Drai-Zerbib, Véronique 211 

Draschkow, Dejan 97, 134 

Dreiser, Marc 91 

Dreneva, Anna A. 187, 188, 204 

Drieghe, 143, 153, 313 

Du, Hong 315 

Duchowski, Andrew T. 121, 122 

Duffy, Gillian 215 

Dunn, Kirsty 201 

Durand, Jean-Baptiste 145 

**E **

Ebersbach, Titus N. 238 

Ebner-Priemer, Ulrich 222, 237, 259259 

Edelman, A. 281 

Eder, Thérése F. 127 

Edler, Dennis 302 

Eggert, Thomas 191 

Ehinger, Benedikt V. 94 

Ehlers, Jan 67, 81 

Eilers, Sarah 87, 131 

Einhäuser, Wolfgang 263 

Eißfeldt, Hinnerk 217 

Elbaum, Tomer 246 

Elisei, 92 

Elmadjian, Carlos E.L. 79 

Engbert, Ralf 95, 120, 155, 185, 192, 

239, 240 

Erica, Chung-I Su 301 

Ernst, Daniel 235 

Eskola, Eeva 201 

Essig, Kai 135 

Ettinger, Ulrich 158, 160 

Everling, Stefan 159, 162 **F **

Fabius, H. 124 

Faiola, Eliana 160 

Fakude, Pheladi F. 253 

Fasshauer, Teresa 88, 90 

Faure, Sylvane 235 

Fayel, Alexandra 308 

Fedorova, Olga 256 

Fehringer, Benedict 114, 301, 303 

Fei Ngan, Meng 185 

Ferguson, Heather 166 

Ferrara, 208, 209, 210 

Ferrari, Clara 203 

Ferrer, Luciana 312 

Ferris, Daniel P. 137 

Feuerstack, Sebastian 191191 

Fiebach, Christian J. 119, 194 

Filik, Ruth 258 

Fink, Lauren K. 74 

Finke, Andrea 91 

Fisher, Robert B. 106 

Fitzsimmons, 153 

Fleischhaker, Christian 222, 237, 259 

Foerster, Rebecca M. 101 

Fonsova, Natalia 280 

Forlenza, Orestes V. 244 

Foulsham, Tom 174, 222, 237, 259, 290 

Fowler, Hazel 215 

Fracasso, Alessio 124 

Franěk, Marek 299 

Frankenstein, Julia 135 

Freije, L. 266, 273 

Freitas Pereira Toassi, Pâmela 170 

Frey, Aline 317 

Freytag, Sarah-Christin 68 

Friede, Anne 248 

Friedrich, Carsten 289 

Friesen, Deanna 120, 252 

Fröhlich, Eva 320 

Fu, Ying 197 

Füssel, Stephan 212, 319 

**G **

Gabriel, Ute 251 

Gagl, Benjamin 119, 194 

Galkina, Natliya 214 

Gallagher-Mitchell, Thomas 147 

Galley, Niels 285 

Gambino, Renata 97 

Gameiro, Ricardo R. 89, 261 

Gamer, Matthias 225 

Ganizada, Jahan 227 

Gao, Qi 198 

Gao, Ying 125 

García-Blanco, Ana 176 

Garzorz, Isabelle 110 

Gasaneo, Gustavo 266, 273 

Gaschler, Robert 211 

Gasimov, Anton 242 

Gayet, Surya 76 

Gayraud, Katja 217 

Gegenfurtner, Karl R. 59, 231, 232, 277, 

282 

Gehrer, Nina 122, 225 

Gehsmann, Kristin 208, 208 

Gellersen, Hans 67, 69, 80, 243 

Geng, Joy J. 74 

Georgieva, Stani 63 

Gerber, Stephan 133 

Gerjets, Peter 137 

Gestefeld, Birte 324 

Geyer, Thomas 98 

Giannini, 124 

Gijbels, David 128 

Gil de Gómez Pérez, David 

Gilchrist, Iain D. 174, 236 

Gillies, Anna 201 

Giora, Rachel 258 

Giordano, Daniela 97 

Glover, Arren 150 

Gochna, Michał 103 

Godfroid, Aline 140 

Golch, 194 

Goldinger, Stephen D. 100, 102 

Goller, Florian 224 

Gonnerman, Laura 120 

Gonzalez, Claudia C. 267 

Gonzalez-Garduño, Ana V. 267 

Goossens, Jeroen 267 

Gosch, Nora 307 

Graf, Tim 188 

Graff, Christian 276 

Graham, 165 

Greenberg, Robert J. 177 

Greenwald, Scott 93 

Gregory, Hossein A. 156 

Grenzebach, Jan 162 

Grillini, Alessandro 249 

Großekathöfer, Jonas D. 225 

Grüner, Markus 260 

Grzeczkowski, Lukasz 78 

Guérin, Anne 145, 228, 233, 317 

Guest, 164, 165 

Gullifer, Jason 252 

Günther, Franziska 98 

Günther, Thomas 85 

Guth, Björn 221 

Guy, Nitzan 89 

Guyader, Nathalie 245 

Gygax, Pascal 251 

**H **

Haass, 152, 180 

Haataja, Eetu 292 

Hadwin, Julie A. 205 

Haensel, Jennifer X. 108, 261 

Hafed, Ziad M. 234 

Häikiö, Tuomo 180, 201, 202 

Halm, Katja 248 

Halszka, Jarodzka 129, 148, 260, 292 

Hämäläinen, Jarmo A. 70 

Hamm, Ulrich 215 

Hamon, 203 

Hand, Christopher J. 181 

Hanning, Nina M. 113 

Hannula, Markku S. 104, 288, 291 

Hansen, Dan W. 116 

Hansen-Schirra, Silvia 212 

Harbecke, Raphael 136 

Harezlak, Katarzyna 178, 274 

Harquel, Sylvain 245 

Harrison, Neil 154 

Hartmann, 260 

Hartung, Franziska 260 

Hartz, Arne 221 

Haslacher, Constanze 177 

Hasse, Catrin 115, 217 

Hauperich, Anna-Katharina 187 

Hautala, Jarkko 70187 

Hawelka, Stefan 70, 177, 194, 309, 310 

Hayhoe, Mary 109 

He, Liyuan 198, 307 

Hebert, P. 102 

Heeman, Jessica 190 

Heikkilä, Timo 168 

Hein, Oliver 151 

Heinzle, Jakob 66, 159 

Hell, Lorena 279 

Helmchen, Christoph 87, 238 

Henik, Avishai 138 

Henry, Regina 132 

Heon Yoo, Seung 62 Herbik, Anne 89 

Herbort, 307 

Hermena, Ehab W. 84, 313, 315 

Hermens, Frouke 297, 300, 305 

Hershman, Ronen 138 

Herwig, Arvid 277, 278 

Hessel, Annina K. 252 

Hessels, Roy S. 90, 149, 271, 277 

Heuer, Anna 66 

Hiebel, Hannah 280 

Hill, Holger 222, 237, 259 

Himmelstoß, A. 70, 310 

Hlavac, Patrik 292 

Hodgson, Timothy 305 

Hoffmann, Mareike A. 65 

Hoffmann, Michael 89 

Höfler, Margit 236, 280 

Hofmann, Florian 150 

Hofmann, Markus J. 118 

Hohenstein, Sven 82, 143, 193 

Holleman, Gijs A. 90 

Holmberg, 153 

Holmqvist, Kenneth 93, 128, 151, 179, 

271 

Hooge, Ignace T.C. 90, 146, 149, 271, 

277 

Hoppe, David 135 

Horbach, Josefine 85 

Horstmann, Gernot 235 

Hoshi, Hideyuki 228 

Howard, Christina 164, 165 

Howard, L. 175 

Howman, Hannah 258 

Hsiao, Yi-ting 72, 199 

Hsieh, Tsuei-Ju 64, 300 

Huang, Jing 282 

Huang, Li-Yu 304 

Huckauf, Anke 67, 81, 138 

Hucko, Jakub 293 

Huestegge, Lynn 65, 223, 285, 307 

Huet, Sylvain 276 

Hunt, R. 100 

Huovinen, Erkki 74 

Hurlemann, René 158 

Hurley, Brian K. 74 

Husain, Masud 93 

Hutson, John P. 142 

136, Stefanie 40 

Hutzler, Florian 70, 177, 310 

Hynes, Patrick M. 318 

Hyönä, Jukka 143, 168, 201, 202, 251 

**I **

Ingram, Joanne 168, 181 

Ioannidou, Flora 305 

Ioannou, Chara 222, 259 

Ischebeck, Anja 236, 280 

Ishmuratova, Yulia 309 

Ison, Matias J. 101 

Istance, Howell 105 

Ivanchenko, Dar’ya K. 302 

Ivanov, 136 

Izmalkova, Anna 229

**J **

Jacobs, Arthur M. 97, 99, 320 

Jainta, Stephanie 71 

Janata, Petr 74 

Janik, Miroslav 291 

Jared, Debra 120, 252 

Jarosch, Julian 212, 319 

Järvelä, 292 

Jaschinski, Wolfgang 115, 272, 273 

Jaudas, Alexander 167 

Jayes, Lewis T. 119 

Jian, Yu-Cin 207 

Johannesson, Ómar I. 281 

Johnston, Kevin 162 

Joos, Roland 286 

Joosten-ten Brinke, Desiree 260 

Jordan, Timothy R. 84, 155, 309, 315 

Jording, 221 

Jörg, Sophie 123 

Joss, Joëlle 286 

Juhasz, Barbara J. 170 

Juma, Eida J. 84 

Jünemann, Kristin 89 

Jusyte, Aiste 225 

**K **

Kaakinen, Johanna K. 144, 175 

Kacian, 98 

Kalva, Hari 213, 262 

Kamienkowski, Juan E. 101, 312 

Kamiya, Seiya 223, 290 

Kanan, Chris 269 

Kao, Wen-Chung 275 

Karlsson, Hasse 202 

Karlsson, Linnea 202 

Kasatkin, Vladimir 244 

Kaspar, Kai 213, 224, 230 

Kasparbauer, 158, 160 

Kasprowski, Pawel 178, 274 

Kataja, Eeva-Leena 201, 202 

Kaufhold, Lilli 94 

Keller, Laura 264 

Kemner, Chantal 90 

Kersting, Anette 226 

Kienle, Andrea 289 

Kilic, Ozkan 103 

Kim, Young-Suk G. Kim 86, 99 

Kirby, R. 69 

Kirkby, Julie A. 83, 144 

Kirkorian, Heather L. 62 

Kirtley, Clare 141 

Klauer, Gertrud 296 

Klein, Christoph 222, 237, 259 

Kliegl, Reinhold 82 

Klingauf, Anna 303 

Kobyliński, Paweł 216 

Koester, Dirk 33 

Kok, M. 149 

Kolesov, Vladimir 318 

Könemund, Inga 238 

König, Peter 89, 94, 137, 261 

Konina, Alena 83, 319 

Konopka, Agnieszka 300 

Konopka, Martin 292, 293 

Konstantinova, Maria 184 

Korja, Riikka 201 

Korneev, Aleksei 207 

Körner, 186, 236, 237, 280 

Koroleva, Marina 214 

Korpal, Pawel 264 

Koster, Ernst 250 

Kothari, Rakshit 269 

Kovalev, Artem 242, 283 

Kowalski, Jarosław 216 

Krauß, Veronika 303 

Krebs, Marie-Christin 127, 148 

Kreiner, Hamutal 72, 199, 247 

Krejtz, 121 

Krejtz, Krzysztof 121, 122, 123 

Kretzschmar, Franziska 200, 212, 311, 

319 

Krichevets, Anatoly N. 104, 187, 188 

Krisjánsson, Árni 281 

Kristensen, Emmanuelle 228 Kristjansson, Arni 281 

Kroll, Aleksandra 295 

Kronbichler, Martin 70 

Krügel, André 155, 192 

Kuchinke, 302 

Kuijpers, Moniek 258, 284 

Kumar, Chandan 218, 220 

Kunde, Wilfried 307 

Kuniecki, Michał 64 

Kuperman, Victor 130, 131, 132, 193 

Kuric, Eduard 292 

Kursawe, Michael A. 306 

Kurtev, Stoyan 196, 197 

**L **

Lakshminarasimhan, Kaushik J. 111 

Lancry, Oryah 89, 113 

Lange, Elke B. 284 

Lappe, Markus 110 

Lasrich, Annika 87 

Latanov, Alexander V. 184, 214, 244, 

256, 302 

Laubrock, Jochen 86, 143, 193, 239 

Laukamp, Marian D. 239 

Laurinavichyute, 141, 142 

Lawrence, Emily 300 

Laxton, Victoria 164 Layher, Georg 299 

Le Meur, Olivier 282 

Lehner, Matthias C. 308 

Lehtimäki, Taina M. 267 

Leinenger, Mallorie 129, 157 

Leitner, Michael C. 177 

Lemaire, Benoît 317 

Lemoine-Lardennois, Christelle 161, 

203 

Leong, Victoria 63 

Leppänen, Jukka 202 

Leppänen, Paavo H.T. 70 

Leube, Alexander 270 

Li, Guan-Huei 301 

Li, Haichao 254 

Li, Lin 182, 195 

Li, Sainan 206 

Li, Sha 195, 196 

Li, 257 

Li, Xin 254 

Li, Xingshan 312 

Liang, Feifei 198 

Liao, Chia-Ning 275 Lieven, Elena 275 

Lin, John J. H. 305 

Lin, Min 275 

Lin, Sunny S. J. 210, 229, 254, 305 

Lin, Ya-Chi 286 

Lipson, Mychal 269 

Litchfield, 147 

Liu, Jingyao 254 

Liu, Min 206 

Liu, Yanping 156 

Liu, Zhong-Xu 126 

Liversedge, Simon P. 71, 119, 143, 144, 

157, 175, 196, 197, 198, 205, 263, 310, 

313, 315, 316 

Loberg, Otto 70 

Lonigan, Christopher J. 85, 130 

López-Orozco, 179 

Lopukhina, Anastasiya 141 

Lörch, Lucas 301 

Loschky, Lester C. 142 

Lou, Ya 312 

Lüdtke, Jana 97, 99, 320 

Ludwig, Jonas 167 

Ludwig, Karin 183 

Lukas, Greiter 138 

Lukasova, Katerina 226, 284 

Luniakova, 227 

**M **

Ma, Jie 198 

Macdonald, Ross G. 140 

Macedo, Elizeu C. 226 

Machner, Björn 238

Mack, David J. 150 

Mackenzie, Andrew K. 165, 226 

MacNeilage, Paul 110 

Madasamy, 269 

Maertens, Marianne 116 

Magliano, Joseph P. 142 

Major, Alex J. 159 

Mak, Monika 295 

Malek, Lenka 215 

Malmberg, Jonna 292 

Malysheva, Natalia 227 

Manca, Giulia 163 

Manoli, Athina 205 

Marchesotti, 189 

Mardanbegi, Diako 80, 243 

Markham, Charles 259 

Marmarinou, Rea 247 

Marsman, Jan-Bernard C. 249 

Mason, Luke 60 

Mathôt, Sebastiaan 136 

Matsumiya, Kazumichi 278 

Matthis, Jonathan S. 109 

Matuschek, Hannes 82 

Matveeva, 207 

Matzen, Laura 152, 180 

Mayo, David 93 

McCarthy, Ian 249 

McDonald, Scott 72, 199, 247 

McGowan, Victoria A. 118, 182, 195 

McNamara, Laura 271, 272 

Meadmore, Katie L. 307 

Medendorp, Pieter 109 

Megardon, Geoffrey 65 125 

Meghanathan, N. 96, 124 

Meijer, Liesbeth 129 

Meixner, Johannes M. 86 

Melchiorre, Emiliano 241 

Meng, Michael 217 

Menges, Raphael 220 

Menshikova, Galina 318 

Meo, Marcos 273 

Mercer, Olivia 201 

Mersad, Karima 308 

Mestry, 263 

Metzler, Ralf 120 

Mey, Shirley 91 

Meyhöfer, Inga 158, 160 

Michl, Monique 263 

Mikulski, Jacek 103 

Milla, Rahel 293 

Miller, Joe 280 

Minarikova, Eva 291 

Ming-Yi Hsieh, Grace 210, 254 

Mirolla, 241 

Mishra, Ramesh 114 

Missal, Marcus 158 

Młodożeniec, Marek 216 

Moiseeva, Victoria 280 

Monzón, Gastón B. 312 

Moran, Rosalyn 310 

Morand, Marie-Anne 262 

Moreno-Esteva, Enrique G. 288, 291 

Morimoto, Carlos H. 79, 219 

Moro, 251 

Moro, Robert 292 

Morrow, J. Dan 271, 272 

Mota, Mailce B. 170, 255 

Mousikou, Betty 169 

Mühl, Christian 102, 261 

Mulder, Kees 76 

Müller, Hermann J. 98 

Müller, Mathias 134 

Munoz, Douglas P. 69, 231, 232 

Münzer, 301 

Murko, Cornelia 92 

Murphy, Victoria 252 

Murray, Christopher 141 

Musiolek, Lea 99 

Müsseler, Jochen 306 

**N **

Nagels, Arne 200 

Nahari, Tal 113 

Nakamura, 223, 

Narcizo, Fabricio B. 116 

Näsänen, Risto 267 

Nation, Kate 171, 252 

Naughton, Thomas J. 267 

Navrat, Pavol 292, 293 

Neesgaard, Nadia 62 

Neige, Cécilia 245 

Neuert, Cornelia E. 298 

Neumann, Heiko 299 

Nguyen, 68 

Niedzielska, Anna 121, 216 

Niehorster, Diederick C. 93, 105, 146, 

149, 151, 271 

Niemelä, Mikko 267 

Nikolaev, Andrey R. 96, 124, 280 

Nikolova, Mirela 71 

Nitzke, Jean 212, 298 

Niu, Jun-Kai 287 

Nowakowska, Anna 100 

Nugteren, L. 292 

Nürnberger, Frank 296 

Nuthmann, Antje 106, 173 

Nyström, Marcus 128, 149 

**O **

O’Reilly, Randall C. 108 

Oberholzer, Marsha 276 

Obersteiner, Andreas 127 

Obregón, Mateo 72, 199, 247 

Ocal, 

Odobez, Jean-Marc 220 

O'Driscoll, Gillian 176 

Öhlschläger, Sabine 106, 239 

Olivier, Brice 145 

Olsen, Rosanna K. 126 

Ost, James 165 

Öttl, Anton 251 

**P **

Pagán, 84, 171, 315 

Pajkossy, Péter 306 

Paletta, Lucas 92, 243 

Pan, Shasha 316 

Panagl, Mariella 243 

Pannasch, Sebastian 217 

Papadosifos, Nikolaos 249 

Pappusett, Deepti 213 Parikh, Saurin S. 262 

Parsons, Christine 202 

Pascoe, Jeffrey 208, 209, 210 

Paterson, B. 118, 155, 181, 182, 

195, 196 

Pauli, Paul 134 

Pavani, Francesco 222 

Peer, Briken 216 

Pellerin, Denis 276276 

Pelz, Jeff B. 172, 269 

Penkalla, Nadine 259 

Perea, Manuel 176 

Pereira, Marta L. G. F. 244 

Perkins, 271, 272 

Pertzov, Yoni 89, 113 

Pesonen, Henri 201, 202 

Petrova, Tatiana 208 

Petrovsky, Nadine 160 

Petružálek, Jan 299 

Pfeiffer, Thies 150, 218 

Pieczykolan, 65, 285, 307 

Pierides, Stella 98 

Pilarczyk, Joanna 64 

Pipa, 133 

Pisella, Laure 55 

Pitkow, Xaq 111 

Platt, Belinda 245 

Poeppel, David 194 

Poffa, Remo 286 

Polat, Uri 121 

Polosan, Mircea 245 

Pomante, Antonella 109 Popov, Alexander 318 Portugal, Ana M.

Potęga vel Żabik, Katarzyna 103 

Potgieter, 126 

Poth, Christian H. 78 Potthoff, Jonas 237 Pouget, Pierre 158

Pressigout, Alexandra 308 

Prichard, Caleb 253 

Probst, Thomas 299 

Prokopenya, Veronika 242 

Pryss, Rüdiger 299 

Pszeida, Martin 243 

Pulvirenti, Grazia 97 

Puurtinen, Marjaana 74 

**Q **

Quaiser-Pohl, Claudia 304 

Quintana-Nevárez, Carlos-Alberto 179 

**R **

Racsmány, Mihály 306 

Radach, Ralph 85, 86, 99, 118, 130, 

205, 238, 247, 248, 314 

Raettig, 223 

Rahe, Martina 304 

Rahmanian, Sadaf 193 

Räihä, Kari-Jouko 105 

Raijmakers, Maartje 61, 96, 203 

Ralph-Nearman, Christina 258 

Ramirez-Gomez, Argenis 69, 80 

Ramzaoui, Hanane 235 

Rebuschat, Patrick 140 

Reich, Sebastian 95 

Reichert, 299 

Reichle, Erik D. 156 

Reid, Vincent 201 

Reilly, Ronan G. 267, 318 

Reiss, Kristina M. 308 

Remus, Steffen 118 Ren, Guiqin 206 

Renner, Patrick 218 

Richlan, Fabio 70 

Richter, Eike 143 

Richter, Eike M. 193 

Riechelmann, K. 223 

Rifai, Katharina 270 

Rigoll, Gerhard 91 

Risse, Sarah 81, 82 

Ritter, Helge 91 

Rivet, Bertrand 228 

Roberts, Matthew A. J. 72 

Rödiger, Manika 215 

Rolf, Tanina 285 

Rolfs, Martin 277 

Rothkegel, O. M. 95, 240 

Rothkopf, Constantin A. 135 

Rowan, Daniel 144 

Roy, Arup 177 

Roy, Raphaëlle N. 228 

Ruff, Stefan 68 

Rukzio, Enrico 81 

Rüth, Marco 224 

Ryabenkov, Victor 318 

Ryan, Jennifer D. 126 

**S **

Sabel, Bernhard 125 

Salemink, Elske 245 

Salmerón, Ladislao 176 

Sanchez, Alvaro 250 

Sassenhagen, Jona 97, 194 

Sato, Masayuki 278 

Sauer, James 165 

Saville, Christopher 222 

Sawyer, 80, 243 

Schack, Thomas 91, 135 

Schaefer, Christoph 220 

Scharinger, Christian 137 

Scharke, Wolfgang 85 

Scheeff, Jonathan 225 

Scheiter, Katharina 127, 148 

Schenk, Simon 91 

Schenk, Thomas 183 

Schlachter, Tina A. 192 

Schlee, 299 

Schlesewsky, Matthias 200, 212, 319 

Schlösser, Christian 289 

Schmid, Doris 183 

Schmidt, Holger 293 

Schmidtke, Daniel 131, 251 

Schneider, Werner X. 78, 101 

Schnotz, Wolfgang 211 

Schöbi, Dario 159 

Scholz, Agnes 270 

Schönenberg, 122, 225 

Schotter, E. R. Sc 157 

Schroeder, Sascha 87, 131, 169, 311 

Schröger, Erich 185 

Schubert, Torsten 186 

Schubö, Anna 66 

Schueler, Anne 127, 148 

Schulte-Körne, Gerd 245 

Schulte-Rüther, Martin 221 

Schuster, Sarah 70, 177, 310 

Schut, J. 77 

Schütt, Heiko H. 95 

Schütz, Alexander C. 66, 279, 282 

Schwedes, Charlotte 279 

Schwesinger, Franca 306 

Schwetlick, Lisa F. 240 

Scott, Graham 168 

Scott-Brown, Kenneth C. 154 

Sedlmeier, Peter 270 

Seelig, Stefan 82 

Seernani, P. 222, 237, 259 

Šefara, Denis 299 

Seidkhani, Hossein 124 

Sekerina, Irina 142 

Selen, Luc 109 

Sengupta, Korok 218 

Senju, Atsushi 62, 261 

Serratrice, Ludovica 140 

Sfärlea, Anca 245 

Shalóm, Diego E. 312 

Shayan, 265 

She, Hsiao-Ching 286, 304 

Sheen, Mercedes 84, 155, 315 

Shen, Kelly 126 

Sheridan, Heather 170 

Sheynikhovich, Denis 189, 294 Shillcock, Richard 72, 199, 247, 268 

Shioiri, Satoshi 278 

Shulgovskiy, Valery 280 

Shurupova, Marina 244 

Shvarts, Anna 104, 187, 188, 204 

Sicuro , Letícia M. 139 

Siegfried, Rémy 220 

Sigurþórsson, Bjarki D. 281 

Silberg, Johanna E. 88, 90 

Silling, Karen 88, 90 

Simko, Jakub 292 

Simms, Victoria 147 

Simola, Jaana 214 

Skopinceva, Marija 226 

Skuballa, Irene T. 105 

Slattery, J. 83 

Slavutskaya, Maria 280 

Slezak, Diego F. 312 

Sligte, Ilja 76 

Smeets, Jeroen B. J. 79 

Smidekova, Zuzana 291 

Smith, Daniel T. 112 

Smith, Jenny 165 

Smith, Tim J. 62, 73, 261 

Smithson, Hannah E. 187 

Snefjella, 130 

Söhnchen, Bastian 134 

Sonuga-Barke, Edmund 205 

Spakov, Oleg 105 

Spampinato, Concetto 97 

Specht, Juan I. 266 

Spichtig, Alexandra 208, 209, 210 

Spotorno, Sara 107, 235, 300 

Sprenger, Andreas 87, 88, 90, 238 

Staab, Steffen 218, 220 

Stachowiak, 264, 265 

Stainer, Matthew J. 154 

Startsev, Mikhail 90, 107, 233 

Steffens, Maria 158, 160 

Stephan, Klaas E. 66 Stosic, Borko 266 

Stosic, Tatijana 266 

Strauch, Christoph 67, 138 

Strohmaier, Anselm R. 308 

Strukelj, Alexander 128 

Su, Juan 196, 197 

Sui, 255, 256, 313, 314 

Sumner, Petroc 65, 161 

Sun, Yan 205 

Sun, Yuanyuan 206 

Sung, Yao-Ting 275 

Suslow, Thomas 226 

Suzuki, Ayako 249 

Suzuki, Tatsuto 249 

Szinte, Martin 78, 111, 185 

Szőllősi, Ágnes 306 

**T **

Tagu, Jérôme 161 

Tajaddini, Mani 103 

Tan, Ke 246, 314 

Tardel, Anke 212 

Tatler, Ben W. 56, 107, 141, 154 

Tatur, Guillaume 189, 294 

Tcherkassof, Anna 228 

Teixeira, Elisângela N. 139, 170 

Tereshchenko, V. 184, 302 

Theakston, Anna 140 

Theeuwes, Jan 123, 190 

Tiefenbacher, Philipp 91 

Tiffin-Richards, Simon 87, 311 

Tiffin-Richards, Simon P. 131 

Tissier, Geoffrey 144 

Titone, Debra 57, 120, 176, 252 

Titz, Johannes 270 

Tiv, Mehrgol 120 

Toivanen, 104, 288, 291 

Torubarova, Ekaterina 242 Tousley, Elias 209 

Townsend, Jeanne 68, 250 

Toyoshima, Hisashi 234 

Trawinski, Tobiasz 263 

Trukenbrod, Hans A. 95, 162, 240 

Tsai, Jie-Li 301 

Tsodyks, Misha 121 

Tudge, Luke 186 

Tune, Sarah 200 

Tvarozek, 292, 293 

Tyler, Nick 249 

**U **

Ulutas, Berna 295 

**V **

Vainio, Seppo 168, 180 Valsecchi, Matteo 277 van Aswegen, Janne 190 van den Hoven, Emiel 98 van der Schaaf, Marieke 265 Van der Stigchel, Stefan 76, 77, 124, 

190 

Van der Stoep, Nathan 77 Van der Westhuizen, Rykie 287 van Driel, Sharisse Driel 129, 289 Van Dyke, Julie A. 132 van Gog, Tamara 147, 148 van Leeuwen, Cees 96, 124 van Leeuwen, Jonathan 96, 124 

van , Tim 148 

Van Put, Jill 250 van Rensburg, Elize J. 276 van Renswoude, Daan 61, 96, 203 

van Rijn, Hedderik 73 van Wermeskerken, Margot 147, 148 van Zoest, Wieske 222 Varatharajah, Alexander 101 

Vasilev, Martin R. 81, 83, 144 

Vaughan, Phillip B. 141 

Veldre, Aaron 117 

Venjakob, Antje C. 68 

Ventsislavova, Petya 294 

Vergilino-Perez, Dorine 161 

Vergne, Judith 161 

Vig, 241 

Vignali, Lorenzo 177, 310 

Vijayraghavan, Susheel 159 

Vine, Sam 154 

Vingron, Naomi 252 

Visser, Ingmar 61, 96, 203 

Vitu, Françoise 156, 283 

Võ, Melissa L.-H. 134 

Vogeley, Kai 221 Völker, Lisa 239 von der Gablentz, Janina 238 von der Malsburg, Titus 157 von Suchodoletz, Antje 105 von Zuben de Arruda Camargo, Marina 

244 

Voorvaart, 203 

Vorstius, Christian 85, 86, 99, 130, 205 

Vorwerg, Constanze 262 

Vosseler, Anne 88 

**W **

Wade, Nicholas 172 

Wadehn, Federico 150 

Wagner, Inga 211 

Wagner, Michael 246 

Wahl, 270 

Wahn, Basil 137 

Walber, Tina 220 

Walcher, Sonja 186 

Walenchok, Stephen C. 100, 100 

Walker, Robin 283 

Wallot, Sebastian 258 

Wan, Peng 206 

Wang, Jingxin 155, 182, 195, 196, 307, 

316 

Wang, 310 

Wang, Mengsi 316 

Wang, Suxia 316 

Wang, Wen 246, 314 

Wang, Xi 316 

Wang, Xiaotong 256, 313 

Wang, Yali 206 

Warriner, Amy-Beth 251 

Warrington, Kayleigh L. 118, 155, 181, 

182, 195 

Wass, V. 63 

Weal, Mark J. 153 

Weber, Sarah L. 213 

Wechselberger, Ulrich 220 

Weiß, Anna F. 200 

Wentura, Dirk 279 

Wenzlaff, Frederike 216 

Wetze, Nicole 185 

Wetzel, Stefanie 303 

Weutelen, Bertram 334 

White, 231, 232 

White, Sarah J. 118, 155, 182, 195, 256, 

313 

Whitford, Veronica 120, 176, 252 

Wichmann, Felix A. 95, 

Widmann, Andreas 185 

Wilbers, Anne-Kathrin 213, 224, 230 

Wilcockson, Thomas 80 

Willems, Roel M. 98 

Wilms, Inge L. 232 

Wilson, 154 

Winkler, Justine 261 

Winter, Jennifer 238 

Wobrock, Dennis 91 

Wolf, Christian 66 

Wolfe, Jeremy M. 149 

Wolff, Anika 89 

Wollenberg, Luca 185, 185 

Wołoszyn, Kinga 64 

Wood, Greg 154 

Woolhouse, 75 

Wu, Changmin 189 

Wu, Hao 257 

Wu, Ming-Da 257 

Wu, Ying-Tien 257 

**X **

Xie, Fang 195, 307 

Xu, Erjia 255 

Xue, Shuwei 97 

Xuejun, Bai 143, 196, 197, 198, 246, 

296, 314 

**Y **

Yamanoi, Takahiro 234 

Yan, Guoli 143, 196, 197, 206 

Yang, Lizhu 205 

Yao, Yuxiang 195 

Yen, Miao-Hsuan 257 

Yi-Ming , Gloria 290 Yin, Guoen 196, 197 

Ylitalo, Anna-Kaisa 266 

Yonemura, Tomoko 234 

Yong, Keir 249 

Yoshida, Takako 223, 290 

Yu, Chen 63 

**Z **

Zadneprovskaya, Galina V. 302 

Zang, Chuanli 197, 197 

Zeiser, 88 

Zelinsky, J. Ze 156 

Zemblys, Raimondas 151, 179 

Zeni, Silvia 123 

Zerr, Paul 76 

Zhang, Dexiang 257 

Zhang, Kuo 195 

Zhang, Mingzhe 246, 314 

Zhang, Yingying 182, 195, 196 

Zhao, Fang 211 

Zhao, 135 

Zhao, Huizhong 310 

Zheng, Yuwei 257 

Zhou, Helen 93 

Zhu, Zhaoxia 257 

Zimmermann, Daniel 224 

Zimoch, Michael 299 

Zozor, Steeve 233 

Żurawska, Justyna 122 

